# Activity-Directed Synthesis with Intermolecular Reactions: Development of a Fragment into a Range of Androgen Receptor Agonists

**DOI:** 10.1002/anie.201506944

**Published:** 2015-09-11

**Authors:** George Karageorgis, Mark Dow, Anthony Aimon, Stuart Warriner, Adam Nelson

**Affiliations:** School of Chemistry and Astbury Centre for Structural Molecular Biology, University of Leeds Leeds, LS2 9JT (UK) E-mail: s.l.warriner@leeds.ac.uk a.s.nelson@leeds.ac.uk

**Keywords:** activity-directed synthesis, agonists, bioactive molecules, carbenoids, reaction discovery

## Abstract

Activity-directed synthesis (ADS), a novel discovery approach in which bioactive molecules emerge in parallel with associated syntheses, was exploited to develop a weakly binding fragment into novel androgen receptor agonists. Harnessing promiscuous intermolecular reactions of carbenoid compounds enabled highly efficient exploration of chemical space. Four substrates were prepared, yet exploited in 326 reactions to explore diverse chemical space; guided by bioactivity alone, the products of just nine of the reactions were purified to reveal diverse novel agonists with up to 125-fold improved activity. Remarkably, one agonist stemmed from a novel enantioselective transformation; this is the first time that an asymmetric reaction has been discovered solely on the basis of the biological activity of the product. It was shown that ADS is a significant addition to the lead generation toolkit, enabling the efficient and rapid discovery of novel, yet synthetically accessible, bioactive chemotypes.

The discovery of biologically active molecules typically involves the synthesis and testing of many compounds, each individually crafted to optimize the arrangement of functionality. Such workflows tend to induce scientists to exploit a limited palette[1–3] of reliable chemical transformations. As a direct consequence, designed arrays comprise compounds that are readily prepared, tending to limit diversity and, potentially, to focus on unproductive areas of chemical space.

We recently introduced activity-directed synthesis (ADS),[4] a novel discovery approach in which bioactive small molecules emerge together with associated syntheses. ADS is iterative, borrowing concepts from biosynthetic pathway evolution.[5] In each round, the components of diverse reaction arrays are widely varied; by exploiting reactions with many possible outcomes, diverse chemical space is explored. After catalyst removal, the crude product mixtures are screened, and reactions that yield active products inform subsequent reaction array design. Only reactions that yield bioactive product mixtures are ever scaled up to enable the characterization and identification of the responsible products. ADS is function-driven,[6] focusing resources on reactions that yield bioactive products.

We recently harnessed intramolecular metal-catalyzed carbenoid reactions in the ADS of androgen receptor (AR) agonists. ADS drove the discovery of both novel ligands—based on scaffolds with no annotated activity against the receptor—and associated high-yielding syntheses (Figure 1 A). However, in this validation work, reliance on intramolecular reactions meant that the structural diversity of possible products was largely encoded by the substrates used. We envisaged that ADS would be enhanced by exploiting intermolecular reactions as the range of possible reaction outcomes—and thus the chemical space explored—would be dramatically increased. It was proposed to exploit ADS to drive the productive elaboration of the 4-cyano-3-trifluoromethylphenylacetamide fragment found in many modulators of AR.[7] Although the core motif displayed only modest agonism (**1**; EC_50_=92±13 μm; EC_50_=concentration of ligand needed to induce the half-maximal observed effect), we reasoned that intermolecular reactions of related diazo acetamides could help identify productive strategies for fragment growth (Figure 1 C).[8–10]

**Figure 1:**
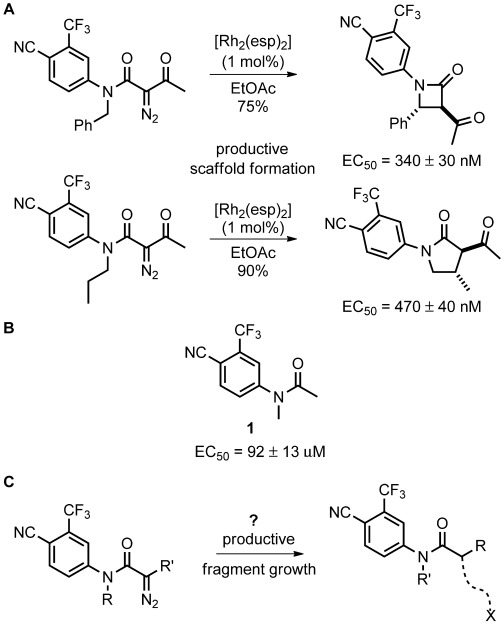
Discovery of novel androgen receptor agonists by ADS. A) Discovery of novel chemotypes enabled by intramolecular metal-catalyzed carbenoid reactions.[4] B) Core fragment for elaboration. C) Envisaged exploitation of intermolecular metal-catalyzed reactions to drive productive fragment elaboration.

The α-diazo amides **2**–**5** incorporate the 4-cyano-3-trifluoromethylphenyl fragment and bear a group (*N*-methyl or *N*-cyclopropyl) expected to suppress intramolecular reactions.[11] In round one, we performed an array of 192 reactions randomly chosen from 480 possible combinations of four substrates (**2**–**5**), ten co-substrates (**6 a**–**6 i** or no co-substrate), six catalysts, and two solvents (CH_2_Cl_2_ or toluene; Figure 2 A). The co-substrates were selected on the basis of diversity of possible intermolecular reactions with metal carbenoids[12–14] and the catalysts on the basis of their diverse reactivity. We initially showed that the diazo substrates and co-substrates were all inactive in our assay (see the Supporting Information). The array was performed in 96-well plates with reactions involving a diazo substrate (100 mm), a co-substrate (1.0 m), and a catalyst (1 mm). To demonstrate significant exploration of chemical space, we showed that a random selection of reactions yielded several products, including, in general, those of both inter- and intramolecular reactions (see the Supporting Information). After 48 h, the crude reaction mixtures were scavenged to remove metal contaminants, evaporated, and assayed for agonism of AR (total concentration of products: 10 μm in 1 % DMSO in pH 7.5 aqueous buffer) using an established assay.[15]

**Figure 2:**
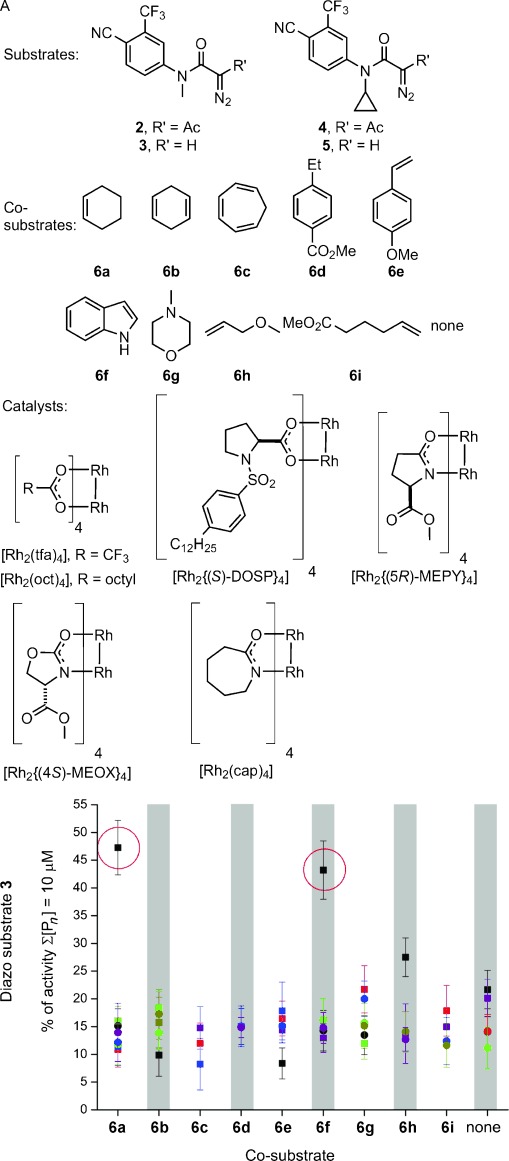
Round one of ADS. A) Substrates, co-substrates, and catalysts used. B) Activities of product mixtures derived from substrate 3 relative to 5 μm testosterone. Reactions involved combinations of substrates, co-substrates, catalysts (represented by different colors; [Rh_2_{(*S*)-DOSP}_4_] black), and solvents (CH_2_Cl_2_ squares; toluene circles). Experiments were performed in duplicate. See the Supporting Information for the activities of product mixtures derived from substrates 2, 4, and 5.

Remarkably, only two of the 192 reactions yielded products that were significantly active (Figure 2 B; see also the Supporting Information): both reactions involved diazo substrate **3**, [Rh_2_{(*S*)-DOSP}_4_], and CH_2_Cl_2_, together with either cyclohexene (**6 a**) or indole (**6 f**) as the co-substrate. These data suggest that the fate of **3**—both with **6 a** and **6 f**—depends critically on the specific catalyst and solvent used, which determine whether the substrate is steered towards active products. The lack of activity without a co-substrate strongly suggests that the active products are derived from intermolecular reactions. At this stage, no products were isolated or identified; instead, the most promising reactions informed subsequent reaction array design.

Round two focused on *N-*methyl diazo substrate **3** and its closely-related *N-*cyclopropyl variant **5**, the co-substrates cyclohexene (**6 a**) and indole (**6 f**) as well as structurally related compounds, and rhodium carboxylate catalysts. The 86 reactions were randomly chosen from 360 possible combinations of the two substrates, 18 co-substrates, five catalysts, and two solvents (CH_2_Cl_2_ or toluene; Figure 3 A). To drive the development of efficient activity-directed syntheses, the crude product mixtures were assayed at two-fold lower total product concentration (5 μm). Five promising combinations of substrate and co-substrate were identified: *N-*methyl diazo substrate **3** with dihydronaphthalene (**6 m**), dihydropyran (**6 o**), or indene (**6 r**), and *N-*cyclopropyl diazo substrate **5** with indole (**6 f**) or 7-azaindole (**6 n**; see Figure 3 B and the Supporting Information). All of these combinations were superior to the most promising combinations from round one.

**Figure 3:**
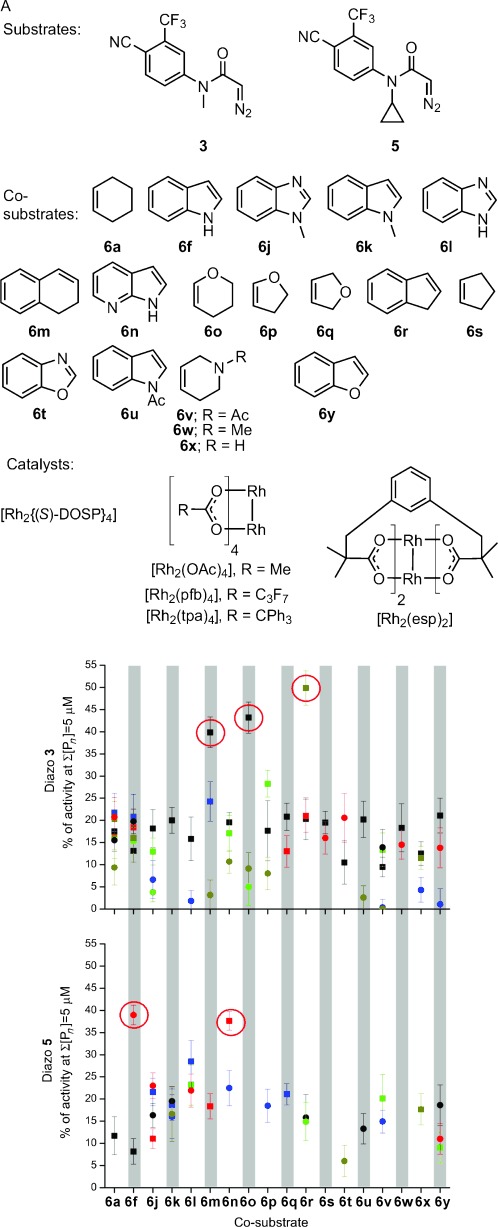
Round two of ADS. A) Substrates, co-substrates, and catalysts used. B) Activities of product mixtures derived from substrates 3 (top) and 5 (bottom) relative to 5 μm testosterone for combinations of co-substrates, catalysts (represented by different colors: [Rh_2_{(*S*)-DOSP}_4_] black; [Rh_2_(esp)_2_] dark yellow; [Rh_2_(OAc)_4_] red), and solvents (CH_2_Cl_2_ squares; toluene circles). Experiments were performed in duplicate.

In round three, substrate **3** was combined with all possible combinations of 12 co-substrates (**6 a, 6 f**, **6 m**, **6 n**, **6 o**, **6 r**, and six structurally related compounds) and four catalysts in CH_2_Cl_2_ (Figure 4 A). After scavenging and evaporating, the crude product mixtures were assayed at five-fold lower total product concentration (1 μm) to increase the selection pressure (Figure 4 B). With co-substrate **6 e′**, the substrate yielded active product mixtures with all four catalysts, with the highest activity for [Rh_2_(OAc)_4_]. However, with dihydropyran **6 f′**, significant activity was only observed when [Rh_2_{(*R*)-DOSP}_4_] had been used; remarkably, no significant activity was observed with the enantiomeric catalyst, suggesting that the most active product is chiral and that [Rh_2_{(*R*)-DOSP}_4_] catalyzes the formation of the more active enantiomer.

**Figure 4:**
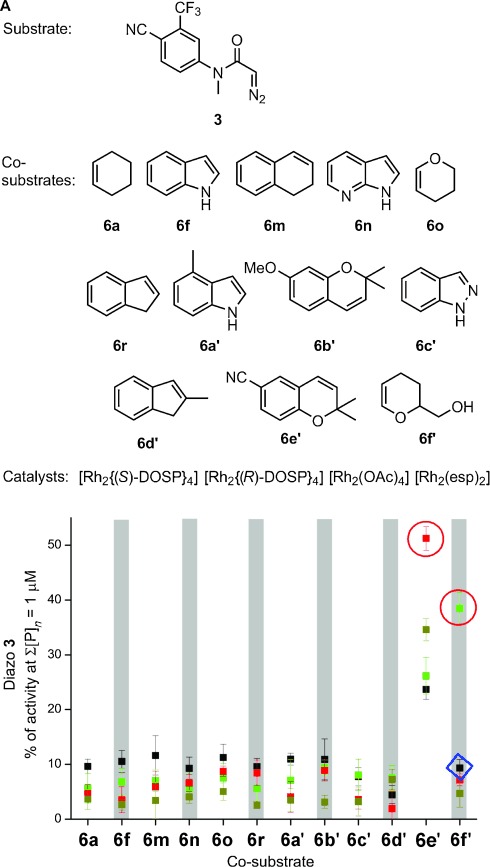
Round three of ADS. A) Substrate, co-substrates, and catalysts used. B) Activities of product mixtures relative to 5 μm testosterone for combinations of co-substrates and catalysts (represented by different colors: [Rh_2_{(*R*)-DOSP}_4_] green; [Rh_2_{(*S*)-DOSP}_4_] black; [Rh_2_(OAc)_4_] red; [Rh_2_(esp)_2_] dark yellow). The blue diamond highlights the activity obtained with the enantiomer of the most promising catalyst with co-substrate 6 f′. Experiments were performed in duplicate.

To understand the basis for the emergence of bioactive structures and associated synthetic routes, prioritized reactions were scaled up from all three rounds of ADS. The reactions were performed on fifty-fold larger scale; the products were purified by column chromatography and their dose-dependent activities determined (Table 1). In all but one case, a product whose activity accounted for that observed in the original array was obtained in good (71–82 %) yield. However, the combination of **5**, **6 n**, and [Rh_2_(OAc)_4_] (from round two) gave the α,β-unsaturated γ-lactam **9** in low (18 %) yield together with recovered starting material **5** (67 %): here, the observed activity must have stemmed from γ-lactam **9** (EC_50_=790±60 nm) rather than the recovered starting material **5** (EC_50_ >500 μm). Crucially, in each case, we were confident that the product that was responsible for the observed activity had been identified.

**Table 1:** Yields and activities of the purified products of reactions that were scaled up

Round^[a]^	Reaction conditions^[b]^	Product (yield)^[c]^	EC_50_^[d]^
1	**3, 6 f**, [Rh_2_{(*S*)-DOSP}_4_]	**7** (80 %)^[e]^	8.8±0.7 μm
1	**3, 6 a**, [Rh_2_{(*S*)-DOSP}_4_]	**8** (71 %)^[e]^	7.3±0.2 μm
			
2^[f]^	**5**, **6 n**, [Rh_2_(OAc)_4_]	**9** (18 %)^[g]^	790±60 nm^[h]^
2	**3, 6 o**, [Rh_2_{(*S*)-DOSP}_4_]	**10** (82 %)^[e]^	4.7±0.1 μm
2	**3**, **6 m**, [Rh_2_{(*S*)-DOSP}_4_]	**11** (76 %)^[e]^	4.9±0.1 μm
2	**3, 6 r**, [Rh_2_(esp)_2_]	**12** (73 %)^[e]^	3.8±0.2 μm
			
3	**3, 6 f′**, [Rh_2_{(*R*)-DOSP}_4_]	(*S*)-**13** (73 %; 56 % *ee*)^[e]^	1.1±0.1 μm^[i]^
3	**3, 6 e′**, Rh_2_(OAc)_4_	**14** (75 %)^[e]^	730±30 nm

[a] Round of ADS. [b] Co-substrate **6** (10 equiv), catalyst (1 mol %), CH_2_Cl_2_. [c] Yield of purified product (see Figure 5 for the structures). [d] Dose-dependent activity of the purified product. [e] Additional products were also isolated whose activity was not significant (see the Supporting Information). [f] See the Supporting Information for the products obtained with **6 f** in place of **6 n**. [g] Substrate **5** was recovered in 67 % yield. [h] Partial agonist (see the Supporting Information). [i] Activity of the purified reaction product that had 56 % *ee*.

In round one, two reactions had yielded product mixtures with significant activity (Figure 5). In each case, the bioactive product was formed in an intermolecular reaction. Amide **7** was the product of C–H insertion into the 3-position of indole (**6 f**), whereas amide **8** was formed by cyclopropanation of cyclohexene (**6 a**). In the other 190 reactions, the product mixtures did not have biological activity that was detectable in the assay. Analysis of a selection of reactions in round one had revealed that a wide range of products had been produced: thus, significant chemical space had been explored—yet discarded—in the search for bioactive products.

**Figure 5:**
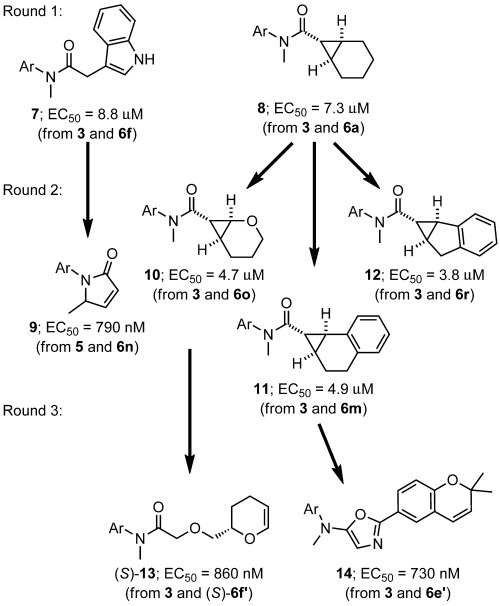
Evolution of bioactive structures driven by ADS. The arrows indicate the relationship between the bioactive compounds formed in prioritized reactions in consecutive rounds. Ar=4-cyano-3-trifluoromethylphenyl.

In round two, the reaction array had been informed by the reactions that yielded amides **7** and **8**. Thus, success with cyclohexene (**6 a**) led to the inclusion of dihydronaphthalene (**6 m**), dihydropyran (**6 o**), and indene (**6 r**) as alternative co-substrates. In each case, screening at lower total product concentrations (5 μm) had enabled the identification of alternative cyclopropanation products (**10**–**12**) with significantly higher biological activities than **8**. Interestingly, with substrate **5**, the introduction of indole (**6 f**) or 7-azaindole (**6 n**) changed the outcome of the reaction. Rather than acting as co-substrates, these additives steered the reaction towards the rearranged product (**9**)[16, 17] of an intramolecular C–H insertion: These reactions were discovered because product **9** is a potent partial agonist.

In round three, dihydropyran **6 f′** and benzopyran **6 e′** had been included as co-substrates on the basis of their similarity to dihydropyran (**6 o**) and dihydronaphthalene (**6 m**). The additional functional groups in both **6 e′** and **6 f′** offer opportunities for different types of intermolecular reactions. In both cases, these new possibilities were exploited in the formation of more bioactive products: amide **13**, which is the product of an O–H insertion,[18, 19] and oxazole **14**, which is formed by reaction with the nitrile moiety of **6 e′**.[20]

Our retrospective analysis revealed two mechanisms by which activity-directed syntheses can develop. First, by varying the specific catalyst and solvent used, the yield of the bioactive products may be optimized. As an example, in round three, the activity of the product mixtures derived from substrate **3** and co-substrate **6 e′** varied widely. With [Rh_2_(OAc)_4_] as the catalyst, oxazole **14** (EC_50_=730±30 nm) was formed in 75 % yield; however, with other catalysts, the observed activity was lower, presumably as a result of **14** being produced in a lower yield. The choice of total product concentration in the assay is crucial as, to optimize the yield of a bioactive product, it is important to screen at concentrations that do not saturate the target protein.

Second, ADS can be used to optimize the structure of bioactive products. For example, the cyclopropanes **10**, **11**, and **12**, which were formed in round two, all had significantly higher activities than cyclopropane **8**, which was formed in round one. Furthermore, introducing reactants with new functional groups can offer new opportunities for forming more active products. For example, amide **13** and oxazole **14** were both formed in types of reaction that were not possible in round two. Such possibilities can expand the chemical space that is explored during the optimization process, enabling the emergence of new synthetically accessible bioactive chemotypes. The emergence of oxazole **14** was particularly interesting: Such heterocycles are widely exploited as peptidomimetics in medicinal chemistry.[21–24] In future ADS campaigns, the inclusion of more functionalized substrates in round one may increase the diversity of the explored chemical space from the outset. It remains to be seen whether other promiscuous reaction classes can be configured to support the discovery of novel bioactive chemotypes using ADS.

In round three, the activity of the product mixture derived from substrate **3** and racemic dihydropyran **6 f′** was dependent upon the enantiomer of the [Rh_2_(DOSP)_4_] catalyst used. HPLC analysis on a chiral stationary phase showed that kinetic resolution of the dihydropyran had occurred to give product **13** in 56 % *ee*. To determine the absolute configuration and activity of both enantiomers, we prepared[25, 26] samples of both enantiomers of **6 f′** and hence **13**: It was found that (*S*)-**13** was approximately five-fold more active than (*R*)-**13** [(*S*)-**13**: EC_50_=860±40 nm; (*R*)-**13**: EC_50_=4.5±0.2 μm] and had indeed been selectively formed in the kinetic resolution of dihydropyran **6 f′** catalyzed by [Rh_2_{(*R*)-DOSP}_4_]. To the best of our knowledge, enantioselective O–H insertion reactions of rhodium carbenoids have not previously been described:[27] Hence, ADS has enabled a novel asymmetric transformation to be identified for the first time solely on the basis of the biological activity of a product.

To demonstrate the value of ADS in lead generation, we performed a limited SAR study inspired by amide **13** and oxazole **14**. A range of 17 analogues was prepared by Rh-catalyzed reactions of α-diazo amide **3** with either alcohols or nitriles. The activity of the analogues spanned well over two orders of magnitude in both series, allowing key structural features to be identified (Figure 6; see also the Supporting Information, Table S2). Therefore, ADS can enable the discovery of bioactive chemotypes that provide options for subsequent lead optimization.

**Figure 6:**
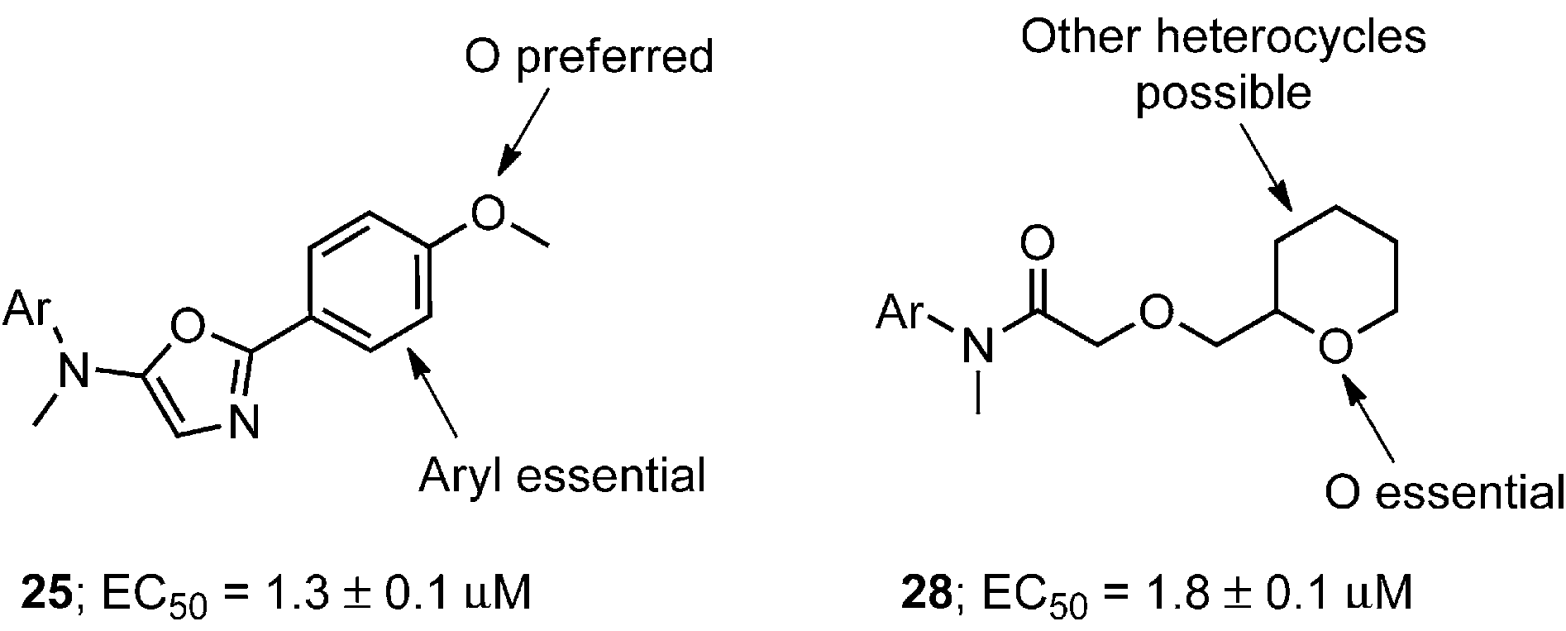
Structures of the most active analogues and summary of the limited SAR study. See the Supporting Information for information on analogues 21–37. Ar=4-cyano-3-trifluoromethylphenyl.

In conclusion, intermolecular reactions add significant value to ADS by dramatically expanding the range of reaction outcomes. By exploiting intermolecular reactions of diazo acetamides, ADS enabled the rapid elaboration of a fragment (**1**) to produce sub-micromolar agonists, all based on chemotypes with no previously reported activity against AR. In total, just four substrates were prepared, yet exploited in a total of 326 reactions to explore diverse chemical space; however, the products of just nine reactions were purified to reveal a range of novel bioactive ligands. In just three rounds of ADS, the activity of the fragment was improved by up to a factor of 125. Retrospective analysis showed that ADS had enabled the efficient exploration of chemical space and facilitated the identification of increasingly active AR agonists, together with associated syntheses. Remarkably, the approach also led to the discovery of a novel asymmetric transformation on the basis of the biological activity of the product alone. Overall, ADS is a significant addition to the lead generation toolkit, enabling the rapid discovery of novel, yet synthetically accessible, bioactive small molecules.

## Supporting Information

As a service to our authors and readers, this journal provides supporting information supplied by the authors. Such materials are peer reviewed and may be re-organized for online delivery, but are not copy-edited or typeset. Technical support issues arising from supporting information (other than missing files) should be addressed to the authors.

miscellaneous_information
