# Supplementary material for: Activity-Directed Synthesis with Intermolecular Reactions: Development of a Fragment into a Range of Androgen Receptor Agonists
Source: Angew Chem Int Ed Engl. 2015 Sep 11;54(46):13538–44. doi: 10.1002/anie.201506944 (PMC4648041; doi:10.1002/anie.201506944)
Supplement: Supplementary file 1 — miscellaneous_information [file anie0054-13538-sd1.pdf]

Angewandte Chemie
Eine Zeitschrift der Gesellschaft Deutscher Chemiker

Supporting Information

Activity-Directed Synthesis with Intermolecular Reactions: Development of a Fragment into a Range of Androgen Receptor Agonists

George Karageorgis, Mark Dow, Anthony Aimon, Stuart Warriner, and Adam Nelson**

anie_201506944_sm_miscellaneous_information.pdf

Supporting Information:..2
1. General Information: 2
2. Experimental procedures 3
2.1. Experimental procedures for compounds 3
2.2. Experimental procedures for assaying individual compounds or product mixtures from diazo-arrays rounds 1-3. 5
3. Experimental details for individual compounds. 8
3.1. Preparation of enantiomerically enriched N-[4-cyano-3-(trifluoromethyl)phenyl]-2-(3,4-dihydro-2H-pyran-2-ylmethoxy)-N-methylacetamide, **S-13** and **R-13** 16
3.2. Chiral HPLC analysis for

3,4-Dihydro-2H-pyran-2-ylmethyl acetate, **19** 27
3.3. Chiral HPLC analysis for N-[4-cyano-3-(trifluoromethyl)phenyl]-

(3,4-dihydro-2H-pyran-2-ylmethoxy)-N-methylacetamide, **13** 28
4. Extended Table 1: Reaction conditions and reaction outcomes for the selected scaled-up reactions 29
5. Limited SAR study……………………………………………………….30
5.1 Extended Table 2: Structure Activity Data for oxazole and ether series identified by ADS………………………………………………………..30
5.2 Figure: Activity and molecular properties of the analogues prepared..32
6. Dose-response curves for individual molecules: 32
7. Reaction array Data: 51
8. Activity of individual co-substrates: 53
9. Reaction array tables 55
10. LCMS analysis of product mixtures from round 1 66
11. NMR Spectra 87
12. References: 125

Supporting Information:

1 General Information:

All non-aqueous reactions were carried out under an atmosphere of nitrogen. Water-sensitive reactions were performed in oven- or flame-dried glassware cooled under nitrogen before use. Solvents were removed under reduced pressure using a Büchi rotary evaporator and a Vacuubrand PC2001 Vario diaphragm pump.

Ether refers to diethyl ether and petrol refers to petroleum spirit (b.p. 40-60 °C) unless otherwise stated. All other solvents and reagents were of analytical grade and used as supplied. Commercially available starting materials were obtained from Sigma–Aldrich and Alfa Aesar.

Flash column chromatography was carried out using silica (35-70 μm particles). Thin layer chromatography was carried out on commercially available pre-coated glass or aluminium plates (Merck silica 2 8 8 0 Kieselgel 60F254).

Purification by Mass Directed Liquid chromatography was performed on an Agilent 1260 preparative LC system with a small fraction of the eluent continuously analysed by electrospray ionization using an Agilent 6120 mass spectrometer. Detection of the target ion in the mass spectrometer triggered automatic collection of the fraction. The system used an XBridge Prep C18 5µm OBD column (19×100mm) and elution was effected with a binary gradient of MeOH and H_2O containing 0.1% formic acid.

Analytical LC-MS was performed using a system comprising of a Bruker HCT Ultra ion trap mass spectrometer equipped with electrospray ionization and an Agilent 1200 series LC made up of, a high vacuum degasser, a binary pump, a high performance autosampler, an autosampler thermostat, a thermostated column compartment and diode array detector. The system used a Phenomenex Luna C18 50 × 2 mm 5 micron column and elution was effected with a binary gradient of two solvent systems: MeCN/H_2O + 0.1% Formic acid or MeCN/H_2O.

Proton and carbon NMR spectra were recorded on a Bruker Avance DPX 300, Avance 500, AV-3 400 or DRX 500 or JEOL ECA600II spectrometer using an internal deuterium lock. Carbon NMR spectra were recorded with composite pulse decoupling using the watts 16 pulse sequence. DEPT, COSY, HMQC and HMBC pulse sequences were routinely used to aid the assignment of spectra. Chemical shifts are quoted in parts per million downfield of tetramethylsilane, and coupling constants (*J*) are given in Hz. NMR spectra were recorded at 300 K unless otherwise stated.

Melting points were determined on a Reichert hot stage microscope and are uncorrected.

Infrared spectra were recorded on a Bruker alpha FT-IR spectrometer using a "platinum ATR" accessory and are reported in wavenumbers (cm^{-1}).

Optical rotation measurements were carried out at the sodium D-line (589 nm) on a Schmidt & Haensch Polatronic H532 polarimeter; concentrations are g/100 mL, temperatures given in °C, optical rotations are given in

10^{-1}degcm^{2}g^{-1} (units are omitted). Chiral HPLC was carried out on an Agilent Infinity 1290 series HPLC system. Racemic standards were obtained by preparing samples of both enantiomers and then combining in an approx. 1:1 ratio.

Nominal mass spectrometry was routinely performed on a Bruker HCT Ultra spectrometer using electrospray (+) ionization. Nominal and accurate mass spectrometry using electrospray ionisation was carried in the School of Chemistry at the University of Leeds, using a Bruker MaXis Impact spectrometer.

EC_{50} values were obtained by performing logistic fits to the appropriate dose-response curves using the OriginPro v.8.6 software.

2 Experimental procedures

2.1 Experimental procedures for compounds

General procedure A: Amination of 4-iodo-2(trifluoro)methylbenzonitrile with primary amines.

The amine (3 equiv.) was added to a stirred solution of 4-iodo-2(trifluoro)methylbenzonitrile (1 equiv.), CuI (10 mol%), L-proline (20 mol%) and K_2CO_3 (3 equiv.) in DMSO (0.5 M) and the reaction mixture was heated to 80 °C. After 20 h the reaction mixture was allowed to cool to rt. Water (10 mL) was added and the resulting mixture was extracted with EtOAc (4 × 10 mL). The combined organic layers were washed with brine (sat. aq. sol 20 mL), dried (Na_2SO_4), filtered and concentrated *in vacuo* to give a crude product.

General procedure B: Acylation of secondary anilines with 2,2,6-trimethyl-4*H*-1,3-dioxin-4-one *via* microwave irradiation and diazotisation of the crude reaction mixture.

2,2,6-Trimethyl-4*H*-1,3-dioxin-4-one (1.5 equiv.) was added to a solution of the appropriate secondary aniline (1 equiv.) in toluene (4.3 M). The reaction mixture was irradiated under microwave conditions (100 Watts, 50 psi) at 110 °C for 30 min. The reaction mixture was concentrated *in vacuo* to give a crude product. Et_3N (1.1 equiv.) was added to a stirred solution of the crude product in MeCN (0.5 M) at rt. *p*-ABSA (1.1 equiv.) was added portion-wise to the reaction mixture. After 2 h the reaction mixture was concentrated *in vacuo* to give a crude product.

General Procedure C: Deacylation of diazo-butanamides.

NaOMe (1.1 equiv.) was added portion-wise to a stirred solution of the appropriate diazo-butanamide (1 equiv.) in MeOH (1.0 M) at 0 °C, and the reaction mixture was stirred at 0 °C. After 4 h the reaction mixture was poured into ice-water (10 mL) and layers separated. The aqueous layer was saturated with solid NaCl and extracted with Et_2O (4 × 10 mL). The combined organic layers were dried (Na_2SO_4), filtered and concentrated *in vacuo* to give a crude product.

General Procedure D: Implementation of reaction arrays

The reaction arrays were carried out in 96-well plates (8 × 12) custom made out of PTFE (well volume ~300 µl). Diazo-substrates were dissolved in CH_2Cl_2, to give a final solution of 1.25 M. Co-substrates were dissolved in CH_2Cl_2, to give a final solution of 12.5 M. $Rh^{(II)}$ catalysts were dissolved in THF to give a 25 mM solution. 8 µL of the diazo-substrate solution were added to the appropriate well followed by 80 µL of the appropriate reaction

solvent, 8 μL of the appropriate co-substrate and finally, 4 μL of the appropriate catalyst solution. The final volume of the reaction mixture was 100 μL; the final concentration of diazo-substrate was 100 mM, the final concentration of the co-substrate was 1 M and the final concentration of catalyst was 1 mM. The reaction wells were sealed using disposable sealing caps (Qiagen, Part No. 1051163) and left to react for 48 h at rt and mixed occasionally (*ca*. 2 h) by pipetting. The product mixtures were transferred to scavenging wells pre-loaded with QuadraPure TU resin (30 mg). Each reaction well was washed with the reaction solvent (3 × 50 μL) and the washes transferred to the corresponding scavenging well. The wells were sealed and left for 24 h. The scavenged reaction mixtures were transferred into Eppendorf tubes (500 μL). Each well was washed with the reaction solvent (3 × 100 μL) and the washes transferred to the corresponding Eppendorf tube. The product mixtures were left to evaporate at rt for 48 h. After evaporation the tube rack was placed in a desiccator and placed under vacuum for a further 24 h. The crude was dissolved in 100 μL of DMSO to give a product mixture solution of $\Sigma[P_n]$ = 100 mM.

General Procedure E: Reaction Scale-up process

The scale-up process was identical to the reaction array method (General Procedure D), performed on a 50-fold larger scale. Diazo-substrates were dissolved in CH_2Cl_2, to give a final solution of 1.25 M. Co-substrates were dissolved in CH_2Cl_2, to give a final solution of 12.5 M. $Rh^{(II)}$ catalysts were dissolved in THF to give a 25 mM solution. 400 μL of the diazo-substrate solution were added to a round-bottom flask followed by 4.0 mL of the appropriate reaction solvent, 400 μL of the co-substrate solution and finally, 200 μL of the appropriate catalyst solution and the reaction mixture was stirred at rt. The final volume of the reaction mixture was 5.0 mL; the final concentration of the diazo-substrate was 100 mM, the final concentration of co-substrate was 1 M and the final concentration of catalyst was 1 mM. After 48 h QuadraPure TU resin (500 mg) was added and the mixture stirred at rt. After 24 h the mixture was filtered washing with the reaction solvent (3 × 10 mL) and concentrated *in vacuo* to give a crude product.

2.2 Experimental procedures for assaying individual compounds or product mixtures from diazo-arrays rounds 1-3.

General Procedure F: Assaying individual compounds using the Invitrogen TR-FRET Androgen Receptor co-activator assay kit – agonist mode.

The assay kit was purchased from Invitrogen and used as instructed. The kit contained the protein: AR LBD-His-GST, the fluorescein-tagged peptide, the Tb^{3+}-anti-GST-Ab, co-regulator buffer and DDT.

The biochemical assay was performed in black 384-well plates from Corning (#3676). The plate type was found to be important for high quality reproducible assay results.

Reagents:

Fluorescein-tagged peptide: 100 μM in 50 mM HEPES buffer, pH 7.5, sequence: VESGSSRFMQLFMANDLLT.

AR LBD GST: Rat AR LBD in a buffer pH 7.5, containing protein, stabilising reagents and glycerol (batches were typically supplied at a concentration ranging between 450-550 μmol).

Tb^{3+}-anti-GST antibody: 3.7 μM in 10 mM HEPES buffered saline 137 mM NaCl, 2.7 mM KCl, pH 7.5.

TR-FRET Coregulator buffer: proprietary buffer, pH 7.5, 20% glycerol.

DTT: 1 M in water.

Complete co-regulator buffer A: A solution of 5 mM DTT in TR-FRET co-regulator buffer was prepared freshly before each assay. This solution was then used as the dilution buffer for all materials.

The assay includes three additions per well, in which AR-LBD is added to agonist dilutions or controls, followed by the addition of a pre-mixed fluorescein-tagged peptide/Tb^{3+}-anti-GST antibody solution.

A solution of compound in DMSO at 100× the desired maximum assay concentration was prepared (*e.g.*for testosterone the 100× concentration was 500 µM giving a maximum assay concentration of 5 µM).

A 12-step, 4-fold serial dilution of the compound stock was prepared in DMSO and 1 μL of each dilution point was added to 49 μL of complete co-regulator buffer A. 10 μL of the resulting solution were added to each well in rows A to C, columns 1-12 of a 384 well plate.

Positive Control: A 10 µM solution of testosterone was prepared by adding 2 μL of 500 µM of testosterone in DMSO to 98 μL of complete co-regulator buffer A and 5 μL of this solution were added to each well in row D columns 5 to 8 of the 384 well plate.

Negative Control: A solution of 2% DMSO in complete co-regulator buffer A was prepared and 5 μL of this solution were added to each well in row D, columns 1 to 4 and 9 to 12 of the 384 well plate.

Diffusion TR-FRET baseline control: 10 µL of complete co-regulator buffer A were added to each well in row D, columns 9 to 12 of the 384 well plate.

A 5 nM solution of the AR-LBD in complete co-regulator buffer A was prepared. 5 µL of this solution were added to each well rows A to C, columns 1 to 12 and row D columns 1 to 8, of the 384 well plate.

A solution with a final concentration of 500 nM of the fluorescein-tagged peptide and 5 nM of the Tb^{3+}-anti-GST antibody respectively in complete co-regulator buffer A was prepared. 5 µL of this solution were added to each well rows A to D, columns 1 to 12 of the 384 well plate.

Representative 384-well plate layout:

	1	2	3	4	5	6	7	8	9	10	11	12
A	Agonist in serial dilution in triplicate											
B												
C												
D	Negative Control				Positive Control				Diffusion TR-FRET Control			

The plate was left to equilibrate for 2-4 h and read using a Perkin-Elmer Envision 2103 Multilabel Reader with a 340 nm excitation filter (14 nm bandwidth for the Tb^{3+}) and 495nm (14 nm bandwidth, for the Tb^{3+}) and 520 nm (10 nm bandwidth, for the fluorescein) emission filters and a 400 nm dichroic mirror, with a delay window of 100 µs and an integration window of 200 µs. 5 repeat measurements were obtained and the results of each measurement were averaged during data processing. Each assay was performed at least twice with a z' factor > 0.85.

The results were normalised by dividing the TR-FRET ratio of each well (emission at 520 nm / emission at 495 nm) by the average baseline TR-FRET ratio.

General Procedure G: Assaying reaction mixtures from the microreaction arrays using the Invitrogen TR-FRET Androgen Receptor co-activator assay kit – agonist mode. Stock solutions were prepared as indicated in General Procedure F.

Each microreaction stock solution of $\Sigma[P_n]$ = 100 mM in DMSO was diluted to give a stock assay solutions of $\Sigma[P_n]$ = 1mM, 0.1 mM and 0.01 mM in DMSO, appropriately, for reaction arrays one, two and three, respectively. This solution was used as the 100× agonist solution.

1µL of each 100× agonist solution was added to 49 µL of complete co-regulator buffer A. 10 µL of the resulting solution were added to duplicate wells in a 384 well plate

Positive Control: A 5 µM solution of testosterone was prepared by adding 2 µL of 500 µM of testosterone in DMSO to 98 µL of complete co-regulator buffer A and 5 µL of this solution were added to each well in row D columns 5 to 8 of the 384 well plate.

Negative Control: A solution of 2% DMSO in complete co-regulator buffer A was prepared and 5 μL of this solution were added to each well in row A, columns 1 to 4 and 9 to 12 of the 384 well plate.

Diffusion TR-FRET baseline control: 10 μL of complete co-regulator buffer A were added were added to each well in row A, columns 9 to 12 of the 384 well plate.

A 5 nM solution of the AR-LBD in complete co-regulator buffer A was prepared. 5 μL of this solution were added to each well of the plate with the exception of row A, columns 9 to 12 (Diffusion TR-FRET baseline control).

A solution with a final concentration of 500 nM of the fluorescein-tagged peptide and 5 nM of the Tb^{3+}-anti-GST antibody respectively in complete co-regulator buffer A was prepared. 5 μL of this solution were added to each well of the 384 well plate.

For the first reaction array the final assay concentration was $\Sigma[P_n]$= 10 μM.
For the second reaction array the final assay concentration were $\Sigma[P_n]$ = 10 μM and 5 μM.
For the third reaction array the final assay concentrations were $\Sigma[P_n]$ = 5 μM and 1 μM.

The plate was left to equilibrate for 2-4 h and read using a Perkin-Elmer Envision 2103 Multilabel Reader (see General Method F for optical configuration). 5 repeat measurements were obtained and the results of each measurement were averaged during data processing. Each assay was performed at least twice with a z' factor > 0.85.

The results were normalised by dividing the TR-FRET ratio of each well (emission at 520 nm / emission at 495 nm) by the average baseline TR-FRET ratio. These results were transformed into an activity percentage, by setting the normalised TR-FRET ratio of the positive control as 100% and the normalised TR-FRET ratio of the negative control as 0%.

3 Experimental details for individual compounds.

4-(Methylamino)-2-(trifluoromethyl)benzonitrile, 15

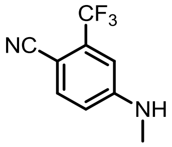

By general procedure A using methylamine HCl salt with two additional equiv. of K_2CO_3 followed by purification by flash chromatography eluting with 50:50 Petrol—EtOAc to yield the *aniline* **15** (860 mg, 86%) as a yellow solid. R_f: 0.53 (50:50, Petrol—EtOAc); δ_H (500 MHz; $CDCl_3$) 7.56 (1H, d, *J* 8.5, Ar 6-H), 6.84 (1H, d, *J* 2.1, Ar 3-H), 6.68 (1H, dd, *J* 8.5 and 2.1, Ar 5-H), 4.54 (1H, s, b, NH), 2.93 (3H, d, *J* 5.5, Me); δ_C (125 MHz; $CDCl_3$) 151.8 (Ar C-4), 136.1 (Ar C-6), 134.0 (q, $^2J_{FC}$ 31, Ar C-2), 122.0 (q, $^1J_{FC}$ 274, *C*F_3), 117.1 (*C*N), 113.5 (Ar C-3), 109.7 (Ar C-5), 95.0 (Ar C-1), 29.9 (Me); ν_{max}/cm^{-1} (film) 3357, 2218; *m/z* (ES) [MH^+] 201.1 (100%, MH^+); HRMS Found: 201.0630 ($C_9H_8F_3N_2$ requires *MH* 201.0634).

***N*-[4-Cyano-3-(trifluoromethyl)phenyl]-*N*-methylacetamide, 1**

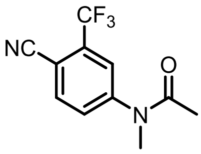

Triethylamine (1.0 mL, 7.5 mmol) was added dropwise over 5 min to a stirred solution of the amine **15** (330 mg, 1.65 mmol), chloromethylpyridinium iodide (575 mg, 2.25 mmol) and acetic acid (86 µL, 1.5 mmol) in CH_2Cl_2 (5.5 mL) at 0 °C. The reaction mixture was allowed to warm to rt and stirred. After 22 h HCl (10% aq. sol., 5.5 mL) was added and layers separated. The organic layer was washed with sat. aq. sol. of $NaHCO_3$ (5 mL) and brine (5 mL), dried, filtered and concentrated *in vacuo*. Purification by flash chromatography, eluting with 50:50 EtOAc—Hexane, afforded the amide **1** as a colourless amorphous solid (139 mg, 35%); δ_H (500 MHz; $CDCl_3$) 7.88 (1H, d, *J* 8.2, Ar 5-H), 7.68 (1H, d, *J* 2.1, Ar 2-H), 7.57 (1H, dd, *J* 8.2 and 2.1, Ar 6-H), 3.38 (3H, s, *N*-Me), 2.11 (3H, s, b, Ac); δ_C (300 MHz; $CDCl_3$) 169.6 (Acetamide C-1 verified *via* HMBC), 148.3 (Ar C-1 verified *via* HMBC), 136.1 (Ar C-5), 134.7 (Ar C-3 verified *via* HMBC), 129.7 (Ar C-6), 124.5 (Ar C-2), 114.9 (*C*N), 107.6 (Ar C-4 verified *via* HMBC), 37.5 (N-Me), 22.8 (Acetamide C-2), *C*F_3 not observed; ν_{max}/cm^{-1} (film) 3049, 2210, 1669, 1510; *m/z* (ES) [MH^+] 243.1 (100%, MH^+); HRMS Found: 243.0702 ($C_{23}H_{19}F_3NO_2$ requires *MH* 243.0701).

***N*-[4-Cyano-3-(trifluoromethyl)phenyl]-2-diazo-3-oxo-*N*-methylbutanamide, 2**

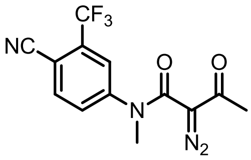

By general procedure B, using aniline **15**, followed by purification by flash chromatography, eluting with 90:10 CH_2Cl_2—Et_2O, to yield the butanamide **2** (1.15 g, 88%), as a yellow amorphous solid, R_f: 0.46 (90:10 CH_2Cl_2—Et_2O); δ_H (500 MHz; $CDCl_3$) 7.86 (1H, d, *J* 8.4, Ar 5-H), 7.65 (1H, d, *J* 2.2, Ar 2-H), 7.54 (1H, dd, *J* 8.4 and 2.2, Ar 6-H), 3.45 (3H, s, N*Me*), 2.35 (3H, s, butanamide 4-H); δ_C (125 MHz; $CDCl_3$) 187.9 (butanamide C-3), 161.5 (butanamide C-1), 147.7 (Ar C-1), 136.1 (Ar C-5), 134.4 (q, $^2J_{CF}$ 32, Ar C-3), 128.0 (Ar C-6), 122.9 (Ar C-2), 121.8 (q, $^1J_{CF}$ 274, *C*F_3), 114.7 (*C*N), 107.6 (Ar C-4), 75.9 (butanamide C-2), 38.3 (N*Me*), 27.7 (butanamide C-4); ν_{max}/cm^{-1}(film) 2231, 2115, 1643, 1606; *m/z* (ES) [MNa^+] 333.1 (100%, MNa^+); HRMS Found: 333.0573, ($C_{13}H_9F_3N_4NaO_2$ requires *MNa* 333.0570).

***N*-[4-Cyano-3-(trifluoromethyl)phenyl]-2-diazo-*N*-(methyl)acetamide, 3**

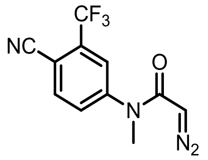

By general procedure C using amide **2**, followed by purification by flash chromatography eluting with 90:10 CH_2Cl_2—Et_2O, to yield the acetamide **3**, (276 mg, 64%) as a pale yellow solid, R_f: 0.40 (90:10 CH_2Cl_2—Et_2O); δ_H (500 MHz; $CDCl_3$) 7.87 (1H, d, *J* 8.4, Ar 5-H), 7.71 (1H, d, *J* 2.4, Ar 2-H), 7.60 (1H, dd, *J* 8.4 and 2.4, Ar 6-H), 4.80 (1H, s, acetamide 2-H), 3.37 (3H, s, N*Me*); δ_C (125 MHz; $CDCl_3$) 165.0 (acetamide C-1), 147.4 (Ar C-1), 135.8 (Ar C-5), 134.0 (q, $^2J_{CF}$ 33, Ar C-3), 129.2 (Ar C-6), 124.2 (Ar C-2), 122.0 (q, $^1J_{CF}$ 288, *C*F_3), 114.9 (*C*N), 108.0 (Ar C-4), 448.3 (acetamide C-2), 28.6 (N*Me*); ν_{max}/cm^{-1} (film) 2235, 2115, 1604; *m/z* (ES) [MNa^+] 291.0 (100%, MNa^+); HRMS Found: 291.0465, ($C_{11}H_7F_3N_4NaO$ requires *MNa* 291.0464).

4-(Cyclopropylamino)-2-(trifluoromethyl)benzonitrile, 16

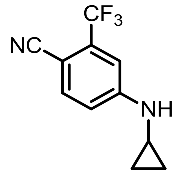

By general procedure A, using cyclopropyl amine, followed by purification by flash chromatography, eluting with 70:30 Petrol—EtOAc, gave the *aniline* **16** (910 mg, 81%) as a pale yellow solid, m.p. 80-84 °C; R_f: 0.75 (50:50 Petrol—EtOAc); δ_H (500 MHz; $CDCl_3$) 7.56 (1H, d, *J* 8.7, Ar 6-H), 7.03 (1H, d, *J* 2.1, Ar 3-H), 6.89 (1H, dd, *J* 8.7 and 2.1, Ar 5-H), 4.96 (1H, s, b, NH), 2.51 (1H, ttd, *J* 6.8, 3.6 and 1.4, cp 1-H), 0.87 (2H, td, J 6.8 and 4.9, cp 2-Ha), 0.58 (2H, td, *J* 4.9 and 3.6, cp 2-Hb); δ_C (125 MHz; $CDCl_3$) 151.8 (Ar C-4), 135.9 (Ar C-6), 134.0 (q, $^2J_{CF}$ 34, Ar C-2), 122.7 (q, $^1J_{CF}$ 284, *C*F_3), 117.2 (*C*N), 114.6 (Ar C-5), 110.6 (Ar C-3), 96.1 (Ar C-1), 24.6 (cp C-1), 7.7 (cp C-2);ν_{max}/cm^{-1} (film) 3351, 2213; *m/z* (ES) [MH^+] 227.1 (100%, MH^+); HRMS Found: 227.0795, ($C_{11}H_{10}F_3N_2$ requires *MH* 227.0790).

***N*-(Cyclopropyl)-*N*-[4-cyano-3-(trifluoromethyl)phenyl]-2-diazo-3-oxobutanamide, 4**

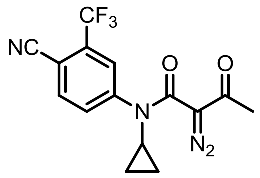

By general procedure B, using aniline **16**, followed by purification by flash chromatography, eluting with 70:30 Petrol—EtOAc, to yield the *butanamide* **4** (560 mg, 83%), as a pale yellow solid; R_f: 0.34 (50:50 Petrol—EtOAc); δ_H (500 MHz; $CDCl_3$) 7.91 (1H, d, *J* 1.9, Ar 2-H), 7.83 (1H, d, *J* 8.6, Ar 5-H), 7.77 (1H, dd, *J* 8.6 and 1.9, Ar 6-H), 3.09 (1H, tt, *J* 7.0 and 3.8, cp 1-H), 2.46 (3H, s, butanamide 4-H), 1.15 (2H, td, *J* 7.0 and 1.4, cp 2-Ha), 0.80 (2H, td, *J* 3.8 and 1.4, cp 2-Hb); δ_C (125 MHz; $CDCl_3$) 189.7 (butanamide C-3), 162.7 (butanamide C-1), 146.4 (Ar C-1), 135.3 (Ar C-5), 133.5 (q, $^2J_{CF}$ 32, Ar C-3), 127.4 (Ar C-6), 122.4 (Ar C-2), 121.6 (q, $^1J_{CF}$ 286, *C*F_3), 115.1 (*C*N), 106.6 (Ar C-4), 75.9 (butanamide C-2), 31.9 (cp C-1), 28.2 (butanamide C-4), 11.1 (cp C-2); ν_{max}/cm^{-1} (film) 2228, 2114, 1642; *m/z* (ES) [MNa^+] 359.1 (100%, MNa^+); HRMS Found: 359.0729 ($C_{15}H_{11}F_3N_4NaO_2$ requires *MNa* 359.0726).

N-(Cyclopropyl)-N-[4-cyano-3-(trifluoromethyl)phenyl]-2-diazo-acetamide, 5

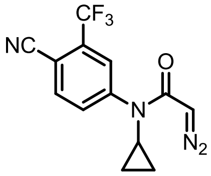

By general procedure C using amide **4**, followed by purification by flash chromatography eluting with 60:40 Petrol—EtOAc, to yield the acetamide **5**, (232 mg, 48%) as a pale yellow solid, R_f: 0.30 (60:40 Petrol—EtOAc); δ_H (500 MHz; $CDCl_3$) 7.85 (1H, d, *J* 1.9, Ar 2-H), 7.79 (1H, d, *J* 8.4, Ar 6-H), 7.68 (1H, dd, *J* 8.4 and 1.9, Ar 5-H), 5.64 (1H, s, acetamide 2-H), 2.97 (1H, tt, *J* 6.9 and 3.8, cp 1-H), 1.13 (2H, td, *J* 6.9 and 1.2, cp 2-Ha), 0.71 (2H, td, *J* 3.8 and 1.2, cp 2-Hb); δ_C (125 MHz; $CDCl_3$) 167.6 (acetamide C-1), 146.1 (Ar C-1), 134.7 (Ar C-5), 133.1 (q, $^2J_{CF}$ 32, Ar C-3), 127.9 (Ar C-6), 123.1 (Ar C-2), 122.1 (q, $^1J_{CF}$ 276, *C*F_3), 115.5 (*C*N), 105.5 (Ar C-4), 49.0 (acetamide C-2), 29.4 (cp C-1), 11.4 (cp C2); ν_{max}/cm^{-1} (film) 2230, 2111, 1636; *m/z* (ES) [MNa^+] 317.2 (100%, MNa^+); HRMS Found: 295.0805 ($C_{13}H_{10}F_3N_4O$ requires *MH* 295.0801).

1-(1,2,3,6-Tetrahydropyridin-1-yl)ethan-1-one, 6v[1]

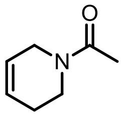

By the method of Stille *et al*.,[1] acetic anhydride (340 μL, 3.6 mmol) was added dropwise to a solution of 1,2,3,6-tetrahydropyridine (274 μL, 3.00 mmol) and Et_3N (830 μL, 6.00 mmol) in CH_2Cl_2 (6 mL, 0.5 M) at 0 °C over ten min. After the addition was completed the mixture was allowed to warm to rt and stirred. After 30 min, sat. aq. NH_4Cl solution (1 mL) was added and layers separated. The organic layer was washed with sat. aq. $NaHCO_3$ solution (2 × 3 mL), dried (Na_2SO_4), filtered and concentrated *in vacuo*. Purification by flash chromatography eluting with 95:5 CH_2Cl_2—MeOH gave the ethanone **6v**[1] (223 mg, 60%) as a pale yellow oil, R_f: 0.45 (95:5 CH_2Cl_2—MeOH); δ_H (500 MHz; $CDCl_3$ observed as a mixture of rotamers) 5.93-5.76 (1H, m, 5-H), 5.73-5.59 (1H, m, 4-H), 4.03 (1H, q, *J* 2.7, 6-H rot A), 3.92 (1 H, q, *J* 2.7, 6-H rot B), 3.65 (1 H, t, *J* 5.7, 2-H rot A), 3.50 (1 H, t, *J* 5.7, 2-H rot B), 2.22-2.11 (2H, m, 3-H), 2.10 (1.6H, s, N-acetyl), 2.08 (1.4H, s, N-acetyl); δ_C (125 MHz; $CDCl_3$) 169.4 (C=O rot A), 169.2 (C=O rot B), 126.5 (C-5 rot A), 124.8 (C-5 rot B), 124.5 (C-4 rot A), 123.2 (C-4 rot B), 45.6 (C-6 rot A), 43.2 (C-2 rot A), 41.8 (C-6 rot B), 37.9 (C-2 rot B), 25.7 (C-3 rot A), 24.8 (C-3 rot B), 21.9 (N-acetyl rot A), 21.4 (N-acetyl rot B); *m/z* (ES) [MNa^+] 148.4 (100%, MNa^+).

Benzyl 1,2,3,6-tetrahydropyridine-1-carboxylate, 6z[2]

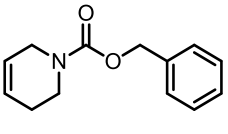

By the method of Larock *et al*.,[2] benzyl chloroformate (1.14 mL, 8.00 mmol) was added dropwise over ten min to a stirred solution of 1,2,3,6-tetrahydropyridine (457 μL, 5.00 mmol) in CH_2Cl_2 (50 mL, 0.1 M) and aqueous $NaHCO_3$ (10.5 mL, 4 M) at 0 °C. After the addition was completed the mixture was allowed to warm to rt and stirred. After 2 h, H_2O (40 mL) was added and layers separated. The aqueous layer was extracted with CH_2Cl_2 (3 × 40 mL) and the combined organic layers were dried (Na_2SO_4), filtered and concentrated *in vacuo*, to yield the carbamate **6z**,[2] as a colourless oil which required no further purification. R_f: 0.42 (80:20 Petrol—EtOAc); δ_H (500 MHz; $CDCl_3$) 7.40-7.37 (2H, m, Ph), 7.36-7.30 (3H, m, Ph), 5.84 (1H, ap. s, b, 5-H), 5.67 (1H, ap. d, b, *J* 21.0, 4-H), 5.16 (2H, s, benzyl-H), 3.97 (2H, ap. quint, *J* 3.2, 6-H), 3.58 (2H, t, *J* 5.9, 2-H), 2.16 (2H, s, b, 3-H); δ_C (125

MHz; $CDCl_3$) 155.6 (C=O), 136.9 (Ph), 135.2 (C-5), 128.7 (Ph), 127.6 (Ph), 126.9 (Ph), 125.4 (C-4), 67.0 (C-6), 40.3 (C-2), 24.9 (C-3); *m/z* (ES) [MH^+] 218.3 (100%, MH^+).

1-Methyl-1,2,3,6-tetrahydropyridine hydrochloride, 6w[3]

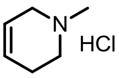

The carbamate **6z** (650 mg, 3.00 mmol) in THF (1 mL) was added dropwise, over 30 min, to a solution of $LiAlH_4$ in THF (6 mL, 1M) at 0 °C. The reaction mixture was allowed to warm to rt and then heated to reflux. After 2 h, the reaction mixture was cooled to 0 °C and H_2O (0.5 mL), NaOH (aq, sol., 15% w/v, 0.5 mL) and H_2O (0.5 mL) were sequentially added dropwise. The mixture was allowed to warm to rt and after 15 min filtered through a pad of Celite eluting with Et_2O (30 mL). The organic layer was extracted with 1 M HCl aqueous solution (2 × 10mL) and H_2O (2 × 10mL) and the combined aqueous layers were concentrated *in vacuo* to give the amine HCl salt **6w**,[3] as a crystalline solid which required no further purification. δ_H (500 MHz; MeOD) 5.91-5.83 (1H, m, 5-H), 5.70-5.62 (1H, m, 4-H), 3.81-3.73 (1H, m, 6Ha), 3.60-3.53 (1H, m, 6-Hb), 3.49-3.42 (1H, m 2-Ha), 3.15 (1H, td, *J* 11.5 and 5.0, 2-Hb), 2.86 (3H, s, N*Me*), 2.56-2.47 (1H, m, 3-Ha), 2.33-2.27 (1H, m, 3-Hb); δ_C (125 MHz; MeOD) 126.3 (C-5), 121.0 (C-4), 52.9 (C-6), 51.6 (C-2), 43.2 (N*Me*), 23.5 (C-3); *m/z* (ES) [MNa^+] 120.2 (100%, MNa^+).

***N*-[4-Cyano-3-(trifluoromethyl)phenyl]-2-(1H-indol-3-yl)-*N*-methylacetamide, 7**

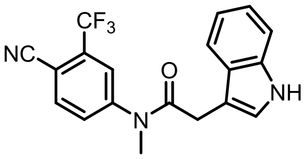

By general procedure E using the diazo-substrate **3**and co-substrate **6f** with Rh_2(*S*-DOSP)$_4$ in CH_2Cl_2 followed by flash chromatography, eluting with 90:10 CH_2Cl_2—Et_2O gave the *acetamide* **7** as a brown viscous oil (143 mg, 80%). R_f: 0.35 (90:10 CH_2Cl_2—Et_2O); δ_H (500 MHz; $CDCl_3$) 8.39 (1H, s, b, N*H*), 7.71 (1H, d, *J* 8.2, Ar 5-H), 7.48 (1H, s, b, Ar 2-H), 7.40 (2H, ap. d, *J* 8.1, Indole 4-H and Ar 6-H), 7.32 (1H, d, *J* 8.1, Indole 7-H), 7.19 (1H, dt, *J* 8.2 and 1.0, Indole 6-H), 7.09 (1H, dt, *J* 7.5 and 1.0, Indole 5-H), 6.83 (1H, s, b, Indole 2-H), 3.79 (2H, s, CH_2), 3.35 (3H, s, N*Me*); δ_C (125 MHz; $CDCl_3$) 171.3 (C=O), 148.0 (Ar C-1), 136.1 (Indole C-7a), 135.7 (Ar C-5), 133.8 (q, $^2J_{CF}$ 33, Ar C-3), 130.1 (Ar C-6), 126.7 (Indole C-3a), 125.0 (Ar C-6), 122.9 (Indole C-2), 122.4 (Indole C-6), 121.6 (q, $^1J_{CF}$ 276, *C*F_3), 119.8 (Indole C-5), 118.3 (Indole C-4), 114.9 (*C*N), 111.4 (Indole C-7), 108.0 (Indole C-3), 107.9 (Ar C-4), 37.7 (N*Me*), 32.3 (*C*H_2); ν_{max}/cm^{-1} (film) 3466, 3055, 2234, 1664, 1609; *m/z* (ES) [MH^+] 358.1 (100%, MH^+); HRMS Found: 358.1175 ($C_{19}H_{14}F_3N_3O$ requires *MH* 358.1162). The regioselectivity was determined by the quaternary nature of C-3 of the indole.

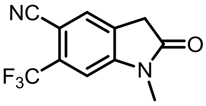

Also obtained was 1-(methyl)-2-oxo-6-(trifluoromethyl)-2,3-dihydro-1*H*-indole-5-carbonitrile **17** as a colourless amorphous solid (13.1 mg, 11 %). R_f: 0.62 (90:10 CH_2Cl_2—Et_2O); δ_H (500 MHz; $CDCl_3$) 7.66 (1H, s, 4-H), 7.16 (1H, s, 7-H), 3.64 (2H, s, 3-H), 3.29 (3H, s, N*Me*); δ_C (125 MHz; $CDCl_3$) 174.0 (C-2), 150.0 (C-7a), 129.8 (C-3a), 135.0 (C-4), 133.0 (q, $^2J_{CF}$ 34 C-6), 124.0 (C-7), 121.0 (q, $^1J_{CF}$ 274, *C*F_3), 116.0 (*C*N), 106.0 (C-5), 34.9 (C-3), 26.6 (N*Me*); ν_{max}/cm^{-1} (film) 2227, 1719; *m/z* (ES) [MH^+] 146.0 (100%, MH^+); HRMS Found: 137.0025, ($C_{11}H_6N_2Na_4O$ requires *MH* 137.0029). Compound **17** was also isolated as a side product in the reactions of diazo-substrate **3** and the following individual co-substrates: **6a**, **6o**, **6m**, **6r**, **6f'**, **6e'** (see Extended Table 1 for details).

N-[4-Cyano-3-(trifluoromethyl)phenyl]-N-methylbicyclo[4.1.0]heptane-7-carboxamide, 8

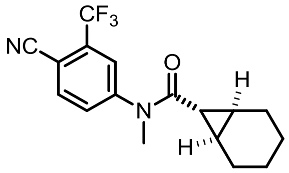

By general procedure E, using the diazo-substrate **3** and co-substrate **6a** with Rh_2(*S*-DOSP)$_4$ in CH_2Cl_2 followed by flash chromatography, eluting with 60:40 Petrol—EtOAc gave the carboxamide **8** (114 mg, 71%). R_f: 0.51 (60:40 Petrol—EtOAc); δ_H (500 MHz; $CDCl_3$) 7.89 (1H, d, *J* 8.2, Ar 5-H), 7.70 (1H, d, *J* 2.2, Ar 2-H), 7.57 (1H, dd, *J* 8.2 and 2.2, Ar 6-H), 3.40 (3H, s, N*Me*), 1.92 (2H, ap. q, 2-Ha and 5-Ha), 1.77-1.71 (2H, m, 1-H and 6-H), 1.54 (2H, ap. quint, 2-Hb and 5-Hb), 1.34-1.20 (2H, m, 3-Ha and 4-Ha), 1.16 (1H, t, *J* 4.2, 7-H), 1.07-0.96 (2H, m, 3-Hb and 4-Hb); δ_c (125 MHz; $CDCl_3$) 173.7 (C=O), 148.6 (Ar C-1), 135.7 (Aryl C-5), 133.8 (q, $^1J_{CF}$ 32, Ar C-3), 129.2 (Ar C-6), 124.6 (Ar C-2), 121.9 (q, $^1J_{CF}$ 276 *C*F_3), 115.0 (*C*N), 107.0 (Ar C-4), 37.0 (N*Me*); 26.5 (C-7), 22.8 (C-1 and C-6), 22.4 (C-2 and C-5), 20.9 (C-3 and C-4); ν_{max}/cm^{-1} (film) 2928, 2855, 2230; *m/z* (ES) [MH^+] 323.2 (100%, MH^+); HRMS Found: 323.1373 ($C_{17}H_{18}F_3N_2O$ requires *MH* 323.1366).

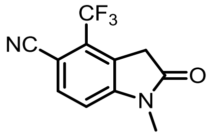

Also obtained was 1-methyl-2-oxo-4-(trifluoromethyl)-2,3-dihydro-1*H*-indole-5-carbonitrile **18** as a yellow solid (14.2 mg, 12 %). R_f: 0.28 (60:40 Petrol—EtOAc); δ_H (500 MHz; $CDCl_3$) 7.81 (1H, d, *J* 8.2, 6-H), 7.04 (1H, d, *J* 8.2, 7-H), 3.76 (2H, s, 3-H), 3.28 (3H, s, N*Me*); δ_C (125 MHz; $CDCl_3$) 173.2 (C-2 verified *via* HMBC), 149.9 (C-7a), 136.2 (C-6), 128.2 (q, $^2J_{CF}$ 30, C-4), 124.3 (C-3a), 115.8 (CN), 110.5 (C-7), 102.8 (C-5), 35.1 (C-3), 26.5 (N*Me*) (*C*F_3 not observed); ν_{max}/cm^{-1} (film) 2921, 2229, 1752; *m/z* (ES) [MH^+] 145.1 (100%, MH^+); HRMS Found: 145.1331 ($C_7H_{17}F_3N_2O$ requires *MH* 145.1335). Compound **18** was also isolated as a side product in the reactions of diazo-substrate **3** and the following co-substrates: **6m**, **6r**, **6f'**, **6e'** (see Extended Table 1 for details).

4-(2-Methyl-5-oxo-2,5-dihydro-1H-pyrrol-1-yl)-2-(trifluoromethyl)benzonitrile, 9

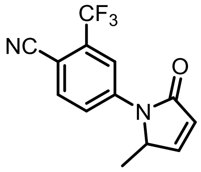

By general procedure E, using the diazo-substrate **5** and co-substrate **6f** with $Rh_2(OAc)_4$ in CH_2Cl_2 followed by flash chromatography, eluting with 95:5 CH_2Cl_2—Et_2O gave the benzonitrile **9** (25 mg, 19%) as a colourless oil. R_f: 0.82 (50:50 Petrol—EtOAc); δ_H (500 MHz; $CDCl_3$) 8.09 (1H, d, *J* 2.2, Ar 3-H), 7.92 (1H, dd, *J* 8.4 and 2.2, Ar 5-H), 7.86 (1H, d, *J* 8.4, Ar 6-H), 7.27 (1H, dd, *J* 6.1 and 1.6, pyrol 3-H), 6.27 (1H, dd, *J* 6.1 and 1.6, pyrol 4-H), 4.90-4.94 (1H, m, pyrol 2-H), 1.39 (3H, d, *J* 6.8, 2-Me); δ_C (125 MHz; $CDCl_3$) 169.7 (C=O), 150.1 (pyrol C-3), 141.2 (Ar C-4), 135.7 (Ar C-6), 133.9 (q, $^2J_{CF}$ 33, Ar C-2), 126.7 (pyrol C-4), 122.7 (Ar C-5), 122.2 (q, $^1J_{CF}$ 274, *C*F_3), 117.9 (Ar C-3), 115.5 (*C*N), 104.3 (Ar C-1), 58.2 (pyrol C-2), 16.9 (2-Me); ν_{max}/cm^{-1}(film) 2236, 1703; *m/z* (ES) [MH^+] 267.0 (100%, MH^+); HRMS Found: 289.0558 ($C_{13}H_9F_3N_2ONa$ requires *MNa* 289.0559).

N-[4-Cyano-3-(trifluoromethyl)phenyl]-N-methyl-2-oxabicyclo[4.1.0]heptane-7-carboxamide, 10

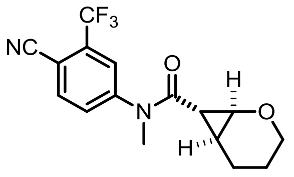

By general procedure E, using the diazo-substrate **3** and co-substrate **6o** with Rh_2(S-DOSP)$_4$ in CH_2Cl_2 followed by flash chromatography, eluting with 1:1 Petrol—EtOAc gave the carboxamide **10** (133 mg, 82%) as a pale yellow amorphous solid. R_f: 0.15 (60:40 Petrol—EtOAc); δ_H (500 MHz; $CDCl_3$) 7.89 (1H, d, J 8.5, Ar 5-H), 7.71 (1H, d, J 2.3, Ar 2-H), 7.60 (1H, dd, J 8.5 and 2.3, Ar 6-H), 3.96 (1H, dd, J 7.2 and 1.7, Heptane 1-H), 3.53 (1H, dtd, J 11.0, 3.4, 1.2, Heptane 3-Ha), 3.40 (3H, s, N*Me*), 3.35 (1H, ddd, J 11.8, 10.9, 1.8, Heptane 3-Hb), 2.04-1.85 (3H, m, Heptane 5-Ha and 5-Hb and Heptane 6-H), 1.58 (1H, d, b, J 4.7, Heptane 7-H), 1.53-1.46 (1H, m, Heptane 4-Ha), 1.29-1.20 (1H, m, Heptane 4-Hb); δ_C (125 MHz; $CDCl_3$) 171.0 (C=O), 148.2 (Ar C-1), 135.7 (Ar C-5), 133.9 (q, $^2J_{CF}$ 32, Ar C-3), 129.4 (Ar C-6), 124.4 (Ar C-2), 121.6 (q, $^2J_{CF}$ 289, *C*F_3), 114.9 (*C*N), 107.2 (Ar C-4), 64.4 (Heptane C-3), 60.8 (Heptane C-1), 36.9 (N*Me*), 27.1 (Heptane C-7), 22.8 (Heptane C-6), 21.9 (Heptane C-4), 18.6 (Heptane C-5); ν_{max}/cm^{-1} (film) 2933, 2859, 2231, 1655, 1608; *m/z* (ES) [MH^+] 325.3 (100%, MH^+); HRMS Found: 325.1166 ($C_{16}H_{16}F_3N_2O_2$ requires *MH* 325.1166).

N-[4-Cyano-3-(trifluoromethyl)phenyl]-N-methyl-*1H,1aH,2H,3H,7bH*-cyclopropa[α]naphthalene-1-carboxamide, 11

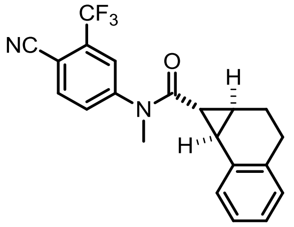

By general procedure E using the diazo-substrate **3** and co-substrate **6m** with Rh_2(S-DOSP)$_4$ in CH_2Cl_2 followed by flash chromatography, eluting with 6:4 Petrol—EtOAc gave the carboxamide **11** (141 mg, 76%) as a colourless amorphous solid. R_f: 0.39 (60:40 Petrol—EtOAc); δ_H (500 MHz; $CDCl_3$) 7.79 (1H, d, J 8.3, Ar 5-H), 7.66 (1H, d, J 2.0, Ar 2-H), 7.52 (1H, dd, J 8.3 and 2.0, Ar 6-H), 7.28 (1H, dd, J 7.5 and 1.4, Ph 7-H), 7.15 (1H, td, J 7.5 and 1.4, Ph 6-H), 7.10 (1H, td, J 7.5 and 1.4, Ph 5-H), 6.97 (1H, d, J 7.5, Ph 4-H), 3.42 (3H, s, N*Me*), 2.72 (1H, dd, J 8.7 and 3.5, 7b-H), 2.63 (1H, dt, J 14.5 and 4.5, 3-Ha), 2.35-2.29 (1H, m, 1a-H or 1-H), 2.18-2.10 (2H, m, 2-Ha and 3-Hb), 1.93-1.80 (2H, m, 2-Hb and 1-H or 1a-H); δ_C (125 MHz; $CDCl_3$) 171.6 (C=O), 148.1 (Ar C-1), 135.7 (Ar C-5), 134.6 (Ph C-3a), 133.9 (q, $^2J_{CF}$ 33, Ar C-3), 133.4 (Ph C-7a), 129.1 (Ar C-6), 128.7 (Ph C-7 and C-4), 126.5 (Ph C-6), 126.2 (Ph C-5), 124.4 (Ar C-2), 121.8 (q, $^1J_{CF}$ 274, *C*F_3), 114.9 (*C*N), 107.2 (Ar C-4), 37.0 (N*Me*), 27.1 (C-7b), 25.3 (C-3 and C-1a or C-1), 24.1 (C-1 or C-1a), 18.3 (C-2); ν_{max}/cm^{-1} (film) 3022, 2924, 2231, 1661, 1608; *m/z* (ES) [MH^+] 371.0 (100%, MH^+); HRMS Found: 371.1368 ($C_{21}H_{18}F_3N_2O$ requires *MH* 371.1366).

N-[4-Cyano-3-(trifluoromethyl)phenyl]-N-methyl-1H,1aH,6H,6aH-cyclopropa[α]indene-1-carboxamide, 12

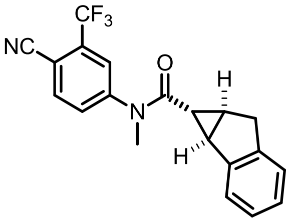

By general procedure E, using the diazo-substrate **3** and co-substrate **6r** with $Rh_2(esp)_2$ in CH_2Cl_2 followed by flash chromatography, eluting with 60:40 Petrol—EtOAc gave the carboxamide **12** (130.1 mg, 73%) as a colourless amorphous solid. R_f: 0.35 (60:40 Petrol—EtOAc); δ_H (500 MHz; $CDCl_3$) 7.81 (1H, d, J 8.3, Ar 5-H), 7.69 (1H, d, J 2.2, Ar 2-H), 7.55 (1H, dd, J 8.3 and 2.2, Ar 6-H), 7.34-7.30 (1H, m, Ph), 7.16-7.09 (3H, m, Ph), 3.41 (3H, s, N*Me*), 3.31 (1H, dd, J 17.6 and 6.4, 6-Ha), 3.12 (1H, ddd, J 6.4, 2.5 and 1.3, 1a-H), 2.90 (1H, d, J 17.6, 6-Hb), 2.59 (1H, tdd, J 6.5, 3.3 and 1.3, 6a-H), 1.00 (1H, s, 1-H); δ_C (125 MHz; $CDCl_3$) 174.2 (C=O), 148.1 (Ar C-1), 143.4 (Ph), 141.8 (Ph), 135.8 (Ar C-5), 133.9 (q, $^2J_{CF}$ 34, Ar C-3), 129.2 (Ar C-6), 126.5 (Ph), 125.4 (Ph), 124.4 (Ar C-2), 123.8 (Ph), 121.8 (q, $^1J_{CF}$ 289, *C*F_3), 114.9 (*C*N), 107.4 (Ar C-4), 37.2 (N*Me*), 35.5 (C-1a), 35.1 (C-6), 31.8 (C-1), 27.5 (C-6a); v_{max}/cm^{-1} (film) 2921, 2230, 1665, 1609; *m/z* (ES) [MH^+] 357.4 (100%, MH^+); HRMS Found: 379.1039 ($C_{20}H_{15}F_3N_2NaO$ requires *MNa* 379.1029).

N-[4-Cyano-3-(trifluoromethyl)phenyl]-2-(3,4-dihydro-2H-pyran-2-ylmethoxy)-N-methylacetamide, 13

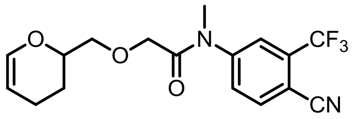

By general procedure E using the diazo-substrate **3** and co-substrate **6f'** with Rh_2(S-DOSP$)_4$ in CH_2Cl_2 followed by flash chromatography, eluting with 95:5 CH_2Cl_2—Et_2O gave the carboxamide **13** (129.3 mg, 73%) as a colourless oil. R_f: 0.18 (50:50 Petrol—EtOAc); δ_H (500 MHz; $CDCl_3$) 7.89 (1H, d, J 8.3, Ar 5-H), 7.75 (1H, d, J 1.7, Ar 2-H), 7.64 (1H, dd, J 8.3 and 1.7, Ar 6-H), 6.33 (1H, d, J 6.2, pyran 6-H), 4.69 (1H, ddd, J 6.2, 3.2 and 1.4, pyran 5-H), 4.20 (1H, d, J 14.2, acetamide 2-Ha), 4.15 (1H, d, J 14.2, acetamide 2-Hb) 3.93 (1H, dddd, J 10.1, 6.2, 4.1 and 2.3, pyran 2-H), 3.58 (2H, ap. d, J 5.6, methoxy C*H_2*), 3.40 (3H, s, N*Me*), 2.14-2.04 (1H, m, pyran 4-Ha), 1.96 (1H, ap. d, J 17.4, pyran 4-Hb), 1.77 (1H, ap. dd, J 13.5 and 5.8, pyran 3-Ha), 1.67-1.56 (1H, m, pyran 3-Hb); δ_C (125 MHz; $CDCl_3$) 168.9 (acetamide C-1), 147.2 (Ar C-1), 143.2 (pyran C-6), 135.8 (Ar C-5), 134.1 (q, $^2J_{CF}$ 34, Ar C-3), 129.3 (Ar C-6), 124.3 (Ar C-2), 121.9 (q, $^1J_{CF}$ 274, *C*F_3), 114.9 (*C*N), 107.9 (Ar C-4), 100.6 (pyran C-5), 74.0 (methoxy *C*H_2), 73.9 (pyran C-2), 70.6 (acetamide C-2), 37.0 (N*Me*), 24.2 (pyran C-3), 19.3 (pyran C-4); v_{max}/cm^{-1} (film) 3060, 2952, 2231, 1675, 1609; *m/z* (ES) [MNa^+] 377.0 (100%, MNa^+); HRMS Found: 377.1094 ($C_{17}H_{17}F_3N_2NaO_3$ requires *MNa* 377.1083).

4-[(2-(2,2-Dimethyl-2H-chromen-6-yl)-1,3-oxazol-5-yl)(methyl)amino]-2-(trifluoromethyl)benzonitrile, 14

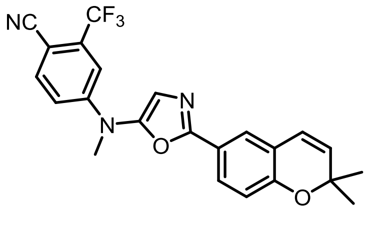

By general procedure E, using the diazo-substrate **3** and co-substrate **6e'** with $Rh_2(OAc)_4$ in CH_2Cl_2 followed by flash chromatography, eluting with 50:50 Petrol—EtOAc gave the oxazole **14** (159 mg, 75%) as a bright yellow oil.

R$_f$: 0.62 (50:50 Petrol—EtOAc); δ_H (500 MHz; $CDCl_3$) 7.74 (1H, dd, *J* 8.5 and 2.1, chromene 7-H), 7.67 (1H, d, *J* 8.7, Ar 6-H), 7.62 (1H, d, *J* 2.1, chromene 5-H), 7.18 (1H, d, *J* 2.6, Ar 3-H), 7.02 (1H, dd, *J* 8.7 and 2.6, Ar 5-H), 6.93 (1H, s, oxazole 4-H), 6.84 (1H, d, *J* 8.5, chromene 8-H), 6.36 (1H, d, *J* 9.8, chromene 4-H), 5.69 (1H, d, *J* 9.8, chromene 3-H), 3.43 (3H, s, NMe), 1.46 (6H, s, chromene 2-Me_2); δ_C (125 MHz; $CDCl_3$) 159.4 (oxazole C-2), 155.4 (chromene C-8a), 150.1 (Ar C-4), 148.7 (oxazole C-5), 135.9 (Ar C-6), 134.3 (q, $^2J_{CF}$ 33, Ar C-2), 131.6 (chromene C-3), 127.3 (chromene C-7), 124.3 (chromene C-5), 122.2 (q, $^1J_{CF}$ 276, *C*F_3), 121.6 (chromene C-4), 121.5 (chromene C-4a), 120.1 (oxazole C-4), 116.8 (chromene C-8), 116.5 (Ar C-5), 116.2 (*C*N), 111.7 (q, $^3J_{CF}$ 5, Ar C-3), 105.0 (chromene C-6), 99.6 (Ar C-1), 77.2 (chromene C-2), 39.6 (N*Me*), 28.2 (chromene 2×2-*Me*); ν_{max}/cm^{-1} (film) 2976, 2923, 2224, 1602; *m/z* (ES) [MH$^+$] 426.0 (100%, MH$^+$); HRMS Found: 426.1428 ($C_{23}H_{19}F_3NO_2$ requires *MH* 426.1424).

N-[4-Cyano-3-(trifluoromethyl)phenyl]-2-(1H-indol-3-yl)-N-cyclopropylacetamide, 20

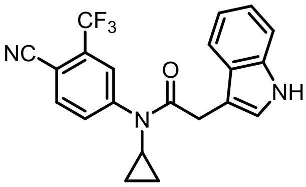

By general procedure E using the diazo-substrate **5** and co-substrate **6f** with $Rh_2(OAc)_4$ in CH_2Cl_2 followed by flash chromatography, eluting with 95:5 CH_2Cl_2—Et_2O gave the carboxamides **20** (103 mg, 54%) as a colourless oil. R$_f$: 0.75 (50:50 Petrol—EtOAc); δ_H (500 MHz; $CDCl_3$) 8.18 (1H, br s, N-*H*), 7.74 (1H, d, *J* 8.4, Ar 5-H), 7.68 (1H, d, *J* 2.0, Ar 2-H), 7.56 (1H, m, Indole 4-H), 7.54 (1H, dd, *J* 8.4 and 2.0, Ar 6-H), 7.35 (1H, ap. d, *J* 8.1, Indole 7-H), 7.20 (1H, ddd, *J* 8.1, 7.0 and 1.0, Indole 6-H), 7.12 (1H, ddd, *J* 8.1, 7.0 and 1.0, Indole 5-H), 7.11-7.08 (1H, m, Indole 2-H), 4.17 (2H, s, C*H_2*), 3.12 (1H, tt, *J* 6.8 and 3.8, cyclopropyl 1H), 1.15-1.09 (2H, m, cyclopropyl 2-Ha), 0.71-0.65 (2H, m, cyclopropyl 2-Hb); δ_C (125 MHz; $CDCl_3$) 173.4 (C=O), 146.6 (Ar C-1), 136.1 (Indole C-7a), 134.8 (Ar C-5), 133.5 (q, $^2J_{CF}$ 34, Ar C-3), 129.3 (Ar C-6), 127.2 (Indole C-3a), 124.4 (q, $^3J_{CF}$ 5, Ar C-2), 122.9 (Indole C-2), 122.4 (Indole C-6), 122.1 (q, $^1J_{CF}$ 274, *C*F_3), 119.9 (Indole C-5), 118.5 (Indole C-4), 115.4 (*C*N), 111.4 (Indole C-7), 108.2 (Indole C-3), 106.3 (Ar C-4), 32.3 (*C*H_2), 31.1 (cyclopropyl C-1), 11.3 (cyclopropyl C-2) ν_{max}/cm^{-1} (film) 3058, 2939, 2221, 1671; *m/z* (ES) [MH$^+$] 384.1 (100%, MH$^+$); HRMS Found: 384.1339 ($C_{21}H_{17}F_3N_3O$ requires *MH* 384.1338). The benzonitrile **9** was also isolated as a side product of this reaction (see Extended Table 1 for details).

3.1 Preparation of enantiomerically enriched *N*-[4-cyano-3-(trifluoromethyl)phenyl]-2-(3,4-dihydro-2*H*-pyran-2-ylmethoxy)-*N*-methylacetamide, *S*-13 and *R*-13

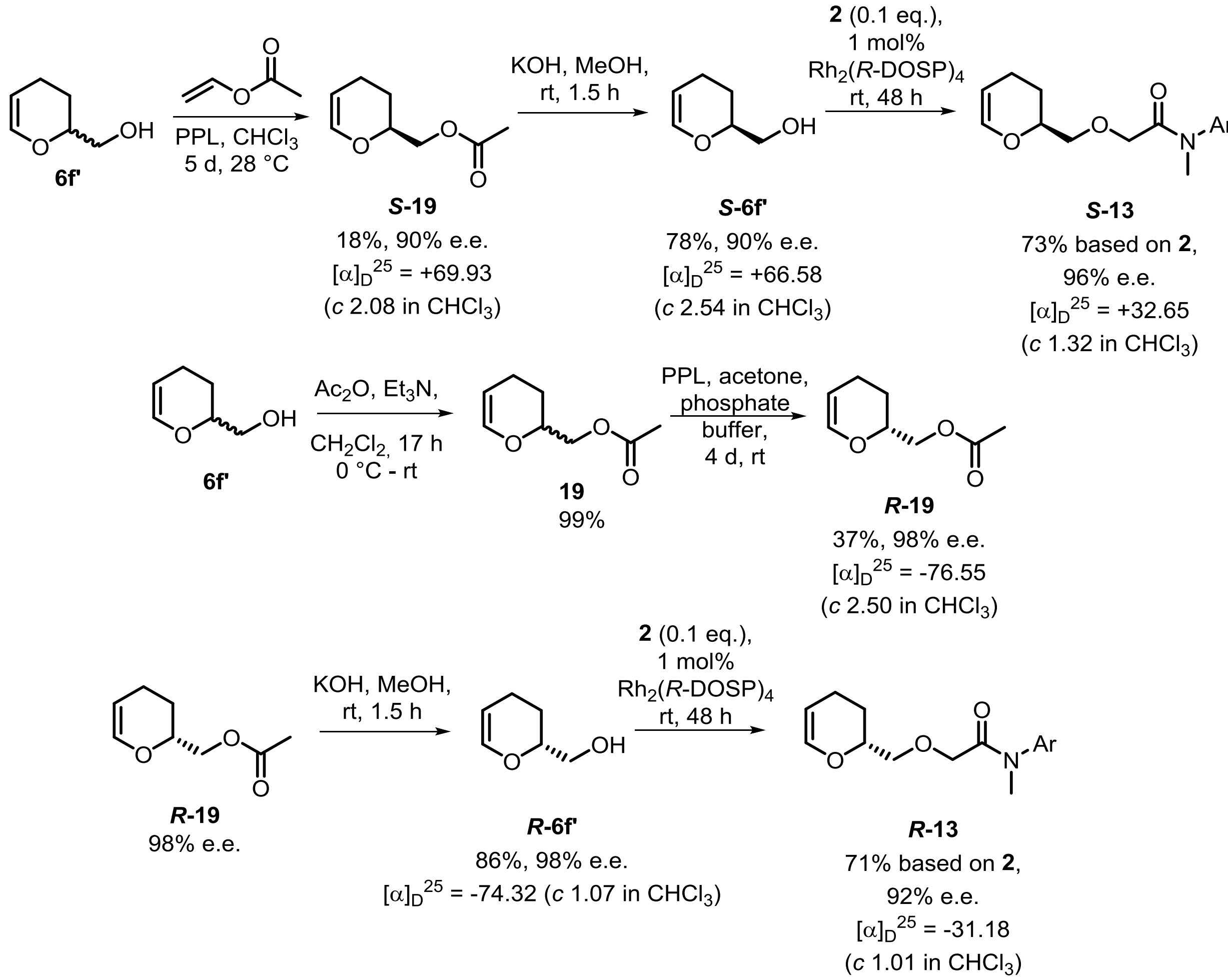

3,4-Dihydro-*2H*-pyran-2-ylmethyl acetate, 19[4]

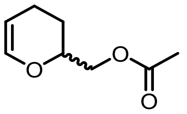

Acetic anhydride (1.13 mL, 12.0 mmol) was added dropwise over 10 min to a stirred solution of 3,4-dihydro-*2H*-pyran-2-yl methanol **6f'** (1.30 mL, 10.0 mmol) and triethylamine (2.75 mL, 20.0 mmol) in CH_2Cl_2 (20 mL, 0.5 M) at 0 °C. The reaction mixture was allowed to warm to rt. After 1 h an extra 6 mmol of acetic anhydride was added and the reaction stirred at rt. After 16 h sat. aq. sol. NH_4Cl (10 mL) was added and the layers were separated. The organic layer was washed with sat. aq. sol. NH_4Cl (2 × 10 mL), dried (Na_2SO_4), filtered and concentrated *in vacuo*. Purification by flash chromatography eluting with 9:1 CH_2Cl_2—Et_2O afforded **19** as a colourless oil (1.55 g, quant.) with a pleasant fruity odor. R_f: 0.58 (90:10 CH_2Cl_2—Et_2O). Spectroscopic data identical to those reported in literature.[4] δ_H (500 MHz; $CDCl_3$) 6.40-6.36 (1H, m), 4.73-4.69 (1H, m), 4.22-4.13 (2H, m), 4.07-4.02 (1H, m), 2.16-2.08 (4H, m), 2.04-1.97 (1H, m), 1.88-1.82 (1H, m), 1.73-1.65 (1H, m); δ_C (125 MHz; $CDCl_3$) 170.9, 143.3, 100.5, 72.7, 66.2, 24.2, 20.8, 19.1.

(2*S*)-3,4-Dihydro-*2H*-pyran-2-ylmethyl acetate, *S*-19[5]

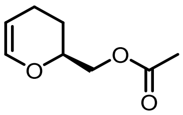

Vinyl acetate (3.1 mL, 67 mmol) was added to a mildly agitated suspension of 3,4-dihydro-*2H*-pyran-2-yl methanol **6f'** (2.1 mL, 20 mmol) and PPL (Sigma, 82 mg, specific activity 27.4 U) in $CHCl_3$ (32 mL, 0.62 M) at 28 °C. After 120 h the reaction mixture was filtered eluting with $CHCl_3$ and concentrated *in vacuo*. Purification by flash chromatography eluting with 9:1 CH_2Cl_2—Et_2O afforded acetate ***S*-19** as a colourless oil (281 mg, 18%, 90% e.e.), $[\alpha]_D^{25}$ = +69.93 (*c* 2.08 in $CHCl_3$) (lit.[5] $[\alpha]_D^{25}$ = +65.7, *c* 0.25, $CHCl_3$) with a pleasant fruity odor with spectroscopic data identical to those obtained for the racemate.

(2*R*)-3,4-Dihydro-*2H*-pyran-2-ylmethyl acetate, *R*-19[4]

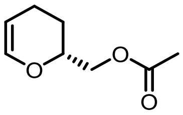

3,4-Dihydro-*2H*-pyran-2-ylmethyl acetate **19** (1.45 g, 10.0 mmol) as a solution in acetone (4 mL) was added to a mildly agitated phosphate buffer aq. solution (K_3PO_4, 20 mM, 1 L, pH = 7.6). The syringe was washed with acetone (2 × 1 mL) and the washes added to the reaction mixture. PPL (Sigma, 10 mg, specific activity 27.4 U) was added to the reaction mixture and stirred at rt. After 18 h additional PPL was added (5 mg). After 96 h the reaction mixture was extracted with Et_2O (4 × 100 mL) and the combined organic layers were dried (Na_2SO_4), filtered and concentrated *in vacuo*. Purification by flash chromatography eluting with 9:1 CH_2Cl_2—Et_2O afforded ***R*-19** as a colourless oil (296 mg, 37%, 98% e.e.), $[\alpha]_D^{25}$ = –76.55 (*c* 2.50 in $CHCl_3$) (lit.[4] $[\alpha]_D^{25}$ = – 76.67, *c* 2.58 in $CHCl_3$) with spectroscopic data identical to those obtained for the racemate.

(2*S*)-3,4-Dihydro-*2H*-pyran-2-ylmethanol, *S*-6f'[5]

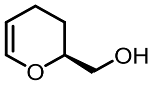

(2*S*)-3,4-Dihydro-*2H*-pyran-2-ylmethyl acetate, ***S*-19** (280 mg 1.80 mmol), was added to a stirred mixture of aq. sol. KOH (30 mg, 1 M) in MeOH (4 mL) at 0 °C. The reaction mixture was allowed to warm to rt and stirred. After 1.5 h the reaction mixture was extracted with Et_2O (5 × 4 mL) and the combined organic layers were allowed to evaporate at rt to give the alcohol ***S*-6f'** as a colourless oil (160 mg, 78%, 90% e.e.) $[\alpha]_D^{25}$ = +66.58 (*c* 2.54 in $CHCl_3$) (lit.[5] $[\alpha]_D^{25}$ = +66.1, *c* 1.7, $CHCl_3$) which required no further purification. Spectroscopic data were identical to those reported in literature[5] δ_H (500 MHz; $CDCl_3$) 6.39 (1H, dt, *J* 6.2 and 1.8), 4.71 (1H, ddt, *J* 6.2, 1.8 and 1.1), 3.94-3.89 (1H, m), 3.73-3.63 (2H, m), 2.33 (1H, ap. t, *J* 5.9), 2.15-2.07 (1H, m), 2.02-1.96 (1H, m), 1.83-1.77 (1H, m), 1.74-1.65 (1H, m); δ_C (125 MHz; $CDCl_3$) 143.3, 100.8, 75.6, 65.4, 23.9, 19.4; ν_{max}/cm^{-1} (film) 3602, 1652.

(2*R*)-3,4-Dihydro-*2H*-pyran-2-ylmethanol, *R*-6f'[4]

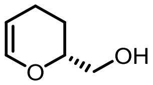

(2*R*)-3,4-Dihydro-*2H*-pyran-2-ylmethyl acetate, ***R*-19** (296 mg, 1.9 mmol), was added to a stirred mixture of aq. sol. KOH (36 mg, 1 M) in MeOH (5 mL) at 0 °C. The reaction mixture was allowed to warm to rt and stirred. After 1.5 h the reaction mixture was extracted with Et_2O (5 × 5 mL) and the combined organic layers were allowed to evaporate at rt to give the alcohol ***R*-6f'** as a colourless oil (254 mg, 86%, 98% e.e.) $[\alpha]_D^{25}$ = –74.32 (*c*1.07 in

$CHCl_3$) (lit.[4][α]$_D^{25}$ = –74.61, *c* 2.52 in $CHCl_3$) which required no further purification with spectroscopic data identical to those reported in literature[4]

N-[4-Cyano-3-(trifluoromethyl)phenyl]-2S-(3,4-dihydro-2H-pyran-2-ylmethoxy)-N-methylacetamide, S-13

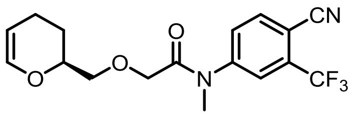

The diazo substrate **3** was dissolved in CH_2Cl_2, to give a final solution of 1.25 M. (2*S*)-3,4-Dihydro-*2H*-pyran-2-ylmethanol, ***S*-6f'** was dissolved in CH_2Cl_2, to give a final solution of 12.5 M. Rh_2(*R*-DOSP)$_4$ was dissolved in THF to give a 25 mM solution. 80 µL of the diazo acetamide solution were added to a round-bottom flask followed by 800 µL of CH_2Cl_2, 80µL of the co-substrate solution and finally, 40 µL of the appropriate catalyst solution and the reaction mixture was stirred at rt. The final volume of the reaction mixture was 1.0 mL; the final concentration of the diazo acetamide was 100 mM, the final concentration of the alcohol was 1 M, and the final concentration of catalyst was 1 mM. After 48 h QuadraPure TU resin (200 mg) was added and the mixture stirred at rt. After 24 h the mixture was filtered washing with the reaction solvent (3 × 10 mL) and concentrated *in vacuo* to give a crude product**.** Purification by flash chromatography, eluting with 95:5 CH_2Cl_2—Et_2O gave the carboxamide ***S*-13** (26 mg, 73%, 96% e.e.) [α]$_D^{25}$ = +32.65 (*c*1.32 in $CHCl_3$) as a colourless oil, with spectroscopic data identical to those obtained previously.

N-[4-Cyano-3-(trifluoromethyl)phenyl]-2R-(3,4-dihydro-2H-pyran-2-ylmethoxy)-N-methylacetamide, R-13

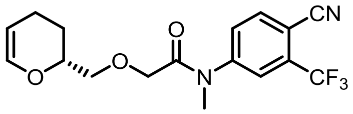

The diazo substrate **3** was dissolved in CH_2Cl_2, to give a final solution of 1.25 M. (2*R*)-3,4-Dihydro-*2H*-pyran-2-ylmethanol, ***R*-6f'** was dissolved in CH_2Cl_2, to give a final solution of 12.5 M. Rh_2(*R*-DOSP)$_4$ was dissolved in THF to give a 25 mM solution. 80 µL of the diazo acetamide solution were added to a round-bottom flask followed by 800 µL of CH_2Cl_2, 80µL of the co-substrate solution and finally, 40 µL of the appropriate catalyst solution and the reaction mixture was stirred at rt. The final volume of the reaction mixture was 1.0 mL; the final concentration of the diazo acetamide was 100 mM, the final concentration of the alcohol was 1 M, and the final concentration of catalyst was 1 mM. After 48 h QuadraPure TU resin (200 mg) was added and the mixture stirred at rt. After 24 h the mixture was filtered washing with the reaction solvent (3 × 10 mL) and concentrated *in vacuo* to give a crude product**.** Purification by flash chromatography, eluting with 95:5 CH_2Cl_2—Et_2O gave the carboxamide ***R*-13** (25 mg, 71%, 92% e.e.) [α]$_D^{25}$ = –31.18 (*c*1.01 in $CHCl_3$) as a colourless oil with spectroscopic data identical to those obtained previously.

4-[(2-(2-Fluorophenyl)-1,3-oxazol-5-yl)(methyl)amino]-2-(trifluoromethyl)benzonitrile 21

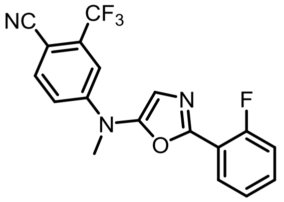

By general procedure E, using the diazo-substrate **3** and 2-fluorobenzonitrile with $Rh_2(OAc)_4$ in CH_2Cl_2 followed by flash chromatography, eluting with 80:20 Hexane—EtOAc gave the desired oxazole (13.8 mg, 20%) as a colourless oil. R$_f$: 0.41 (80:20 Hexane—EtOAc); δ_H (400 MHz; $CDCl_3$) 8.00 (1H, td, *J* 7.6, 1.8, fluorophenyl 6-H), 7.69 (1H, d,

J 8.7, Ar 6-H), 7.50-7.43 (1H, m, fluorophenyl 4-H), 7.29-7.18 (3H, m, Ar 3-H, fluorophenyl 2-H and 5-H), 7.08 (1H, dd, *J* 8.7 and 2.6, Ar 5-H), 7.01 (1H, s, oxazole 4-H), 3.46 (3H, s, NMe); δ_C (101 MHz; $CDCl_3$) 160.1 (d, $^1J_{CF}$ 257, fluorophenyl C-2), 155.3 (d, $^3J_{CF}$ 5, oxazole C-2), 149.9 (d, $^5J_{CF}$ 2, oxazole C-5), 149.8 (Ar C-4), 136.1 (Ar C-6), 134.5 (q, $^2J_{CF}$ 33, Ar C-2), 132.5 (d, $^4J_{CF}$ 9, fluorophenyl C-4), 129.3 (d, $^5J_{CF}$ 2, fluorophenyl C-5), 124.6 (d, $^4J_{CF}$ 4, fluorophenyl C-6), 122.4 (q, $^1J_{CF}$ 274, CF_3), 119.2 (oxazole C-4), 117.1 (d, $^2J_{CF}$ 21, fluorophenyl C-3), 116.9 (Ar C-5), 116.2 (*C*N), 115.4 (d, $^2J_{CF}$ 11, fluorophenyl C-1), 112.3 (q, $^3J_{CF}$ 5, Ar C-3), 100.1 (q, $^3J_{CF}$ 2, Ar C-1), 39.6 (N*Me*); ν_{max}/cm^{-1} (film) 3127, 2923, 2225, 1586, 1493, 1447, 1357, 1222, 1176, 1129, 1044; *m/z* (ES) [MH^+] 362.8 (100%, MH^+); HRMS Found: 362.0907 ($C_{18}H_{12}F_4N_3O$ requires *MH* 362.0911).

4-[(2-Cyclohexyl-1,3-oxazol-5-yl)(methyl)amino]-2-(trifluoromethyl)benzonitrile 22

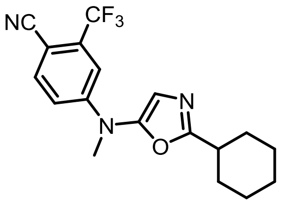

By general procedure E, using the diazo-substrate **3** and cyclohexanecarbonitrile with $Rh_2(OAc)_4$ in CH_2Cl_2 followed by flash chromatography, eluting with 80:20 Hexane—EtOAc gave the desired oxazole (12.0 mg, 20%) as a colourless oil. R_f: 0.58 (80:20 Hexane—EtOAc); δ_H (400 MHz; $CDCl_3$) 7.66 (1H, d, *J* 8.7, Ar 6-H), 7.07 (1H, d, *J* 2.4, Ar 3-H), 6.92 (1H, dd, *J* 8.7 and 2.4, Ar 5-H), 6.75 (1H, s, oxazole 4-H), 3.36 (3H, s, NMe), 2.77 (1H, tt, *J* 11.3, 3.6, cyclohexyl C*H*), 2.10-2.03 (2H, m), 1.86-1.78 (2H, m), 1.74-1.67 (1H, m), 1.64-1.52 (2H, m), 1.45-1.24 (3H, m); δ_C (101 MHz; $CDCl_3$) 166.1 (oxazole C-2), 150.3 (Ar C-4), 148.9 (oxazole C-5), 136.1 (Ar C-6), 134.3 (q, $^2J_{CF}$ 32, Ar C-2), 122.5 (q, $^1J_{CF}$ 274, CF_3), 118.6 (oxazole C-4), 116.4 (*C*N), 116.3 (Ar C-5), 111.8 (q, $^3J_{CF}$ 5, Ar C-3), 99.4 (q, $^3J_{CF}$ 2, Ar C-1), 39.7 (N*Me*), 37.9 (cyclohexane C-CH), 30.5 (cyclohexane 2×C-CH_2), 25.8 (cyclohexane C-CH_2), 25.6 (cyclohexane 2×C-CH_2); ν_{max}/cm^{-1} (film) 2930, 2855, 2225, 1603, 1558, 1505, 1448, 1319, 1270, 1174, 1130, 1105; *m/z* (ES) [MH^+] 350.6 (100%, MH^+); HRMS Found: 350.1478 ($C_{18}H_{19}F_3N_3O$ requires *MH* 350.1474).

4-[(2-(2,4-Dichlorophenyl)-1,3-oxazol-5-yl)(methyl)amino]-2-(trifluoromethyl)benzonitrile 23

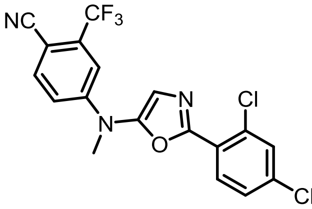

By general procedure E, using the diazo-substrate **3** and 2,4-dichlorobenzonitrile with $Rh_2(OAc)_4$ in CH_2Cl_2 followed by flash chromatography, eluting with 80:20 Hexane—EtOAc gave the desired oxazole (16.6 mg, 22%) as a colourless oil. R_f: 0.43 (80:20 Hexane—EtOAc); δ_H (400 MHz; $CDCl_3$) 7.93 (1H, d, *J* 8.5, dichlorophenyl 6-H), 7.70 (1H, d, *J* 8.7, Ar 6-H), 7.54 (1H, d, *J* 2.1, dichlorophenyl 3-H), 7.36 (1H, dd, *J* 8.5 and 2.1, dichlorophenyl 5-H), 7.25 (1H, d, *J* 2.4, Ar 3-H), 7.09 (1H, dd, *J* 8.7 and 2.6, Ar 5-H), 7.01 (1H, s, oxazole 4-H), 3.46 (3H, s, NMe); δ_C (101 MHz; $CDCl_3$) 155.7 (oxazole C-2), 150.3 (Ar C-4), 149.5 (oxazole C-5), 137.1 (dichlorophenyl C), 136.1 (Ar C-6), 134.5 (q, $^2J_{CF}$ 32, Ar C-2), 133.1 (dichlorophenyl C), 131.5 (dichlorophenyl CH), 131.3 (dichlorophenyl CH), 127.7 (dichlorophenyl CH), 124.5 (dichlorophenyl C), 122.4 (q, $^1J_{CF}$ 274, CF_3), 118.9 (oxazole C-4), 117.1 (Ar C-5), 116.6 (*C*N), 112.5 (q, $^3J_{CF}$ 5, Ar C-3), 100.3 (q, $^3J_{CF}$ 2, Ar C-1), 39.6 (N*Me*); ν_{max}/cm^{-1} (film) 3090, 2925,

2225, 1597, 1561, 1505, 1447, 1356, 1272, 1176, 1132; *m/z* (ES) [MH$^+$] 412.4 (100%, MH$^+$); HRMS Found: 412.0225 ($C_{18}H_{11}{}^{35}Cl_2F_3N_3O$ requires *MH* 412.0225).

4-[(2-*tert*-Butyl-1,3-oxazol-5-yl)(methyl)amino]-2-(trifluoromethyl)benzonitrile 24

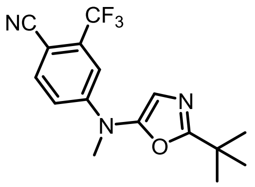

By general procedure E, using the diazo-substrate **3** and trimethylacetonitrile with $Rh_2(OAc)_4$ in CH_2Cl_2 followed by flash chromatography, eluting with 80:20 Hexane—EtOAc gave the desired oxazole (13.3 mg, 22%) as a colourless oil. R_f: 0.54 (80:20 Hexane—EtOAc); δ_H (400 MHz; $CDCl_3$) 7.66 (1H, d, *J* 8.7, Ar 6-H), 7.07 (1H, d, *J* 2.5, Ar 3-H), 6.92 (1H, dd, *J* 8.7 and 2.5, Ar 5-H), 6.73 (1H, s, oxazole 4-H), 3.37 (3H, s, NMe), 1.39 (9H, s, *t*-Bu); δ_C (101 MHz; $CDCl_3$) 168.9 (oxazole C-2), 150.2 (Ar C-4), 149.0 (oxazole C-5), 136.1 (Ar C-6), 134.3 (q, $^2J_{CF}$ 32, Ar C-2), 122.5 (q, $^1J_{CF}$ 274, *C*F$_3$), 118.3 (oxazole C-4), 116.4 (*C*N), 116.2 (Ar C-5), 111.8 (q, $^3J_{CF}$ 5, Ar C-3), 99.4 (q, $^3J_{CF}$ 2, Ar C-1), 39.7 (N*Me*), 34.3 (*t*-Bu *Cq*), 28.5 (*t*-Bu *Me$_3$*); ν_{max}/cm^{-1} (film) 2973, 2933, 2225, 1604, 1556, 1506, 1449, 1356, 1270, 1176, 1044; *m/z* (ES) [MH$^+$] 324.5 (100%, MH$^+$); HRMS Found: 324.1321 ($C_{16}H_{17}F_3N_3O$ requires *MH* 324.1318).

4-[(2-(4-Methoxyphenyl)-1,3-oxazol-5-yl)(methyl)amino]-2-(trifluoromethyl)benzonitrile 25

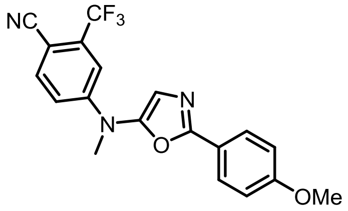

By general procedure E, using the diazo-substrate **3** and 4-methoxybenzonitrile with $Rh_2(OAc)_4$ in CH_2Cl_2 followed by flash chromatography, eluting with 80:20 Hexane—EtOAc gave the desired oxazole (12.4 mg, 20%) as a colourless oil. R_f: 0.35 (80:20 Hexane—EtOAc); δ_H (500 MHz; $CDCl_3$) 7.93 (2H, d, *J* 8.9, methoxyphenyl 2×2-H), 7.67 (1H, d, *J* 8.7, Ar 6-H), 7.19 (1H, d, *J* 2.4, Ar 3-H), 7.02 (1H, dd, *J* 8.7 and 2.4, Ar 5-H), 6.98 (2H, d, *J* 8.9, methoxyphenyl 2×3-H), 6.94 (1H, s, oxazole 4-H), 3.87 (3H, s, OMe), 3.46 (3H, s, NMe); δ_C (101 MHz; $CDCl_3$) 161.9 (oxazole C-2 or methoxyphenyl C-4), 159.5 (oxazole C-2 or methoxyphenyl C-4), 150.3 (Ar C-4), 148.9 (oxazole C-5), 136.1 (Ar C-6), 134.5 (q, $^2J_{CF}$ 32, Ar C-2), 128.0 (methoxyphenyl 2×C-2), 122.5 (q, $^1J_{CF}$ 274, *C*F$_3$), 120.2 (oxazole C-4), 119.9 (methoxyphenyl C-1), 116.6 (Ar C-5), 116.3 (*C*N), 114.5 (methoxyphenyl 2×C-3), 111.8 (q, $^3J_{CF}$ 5, Ar C-3), 99.7 (q, $^3J_{CF}$ 2, Ar C-1), 55.6 (O*Me*), 39.8 (N*Me*); ν_{max}/cm^{-1} (film) 2946, 2840, 2225, 1732, 1604, 1498, 1448, 1320, 1219, 1131, 1080; *m/z* (ES) [MH$^+$] 374.2 (100%, MH$^+$); HRMS Found: 374.1112 ($C_{19}H_{15}F_3N_3O_2$ requires *MH* 374.1111).

4-[(2-(4-Bromo-3-methylphenyl)-1,3-oxazol-5-yl)(methyl)amino]-2-(trifluoromethyl)benzonitrile 26

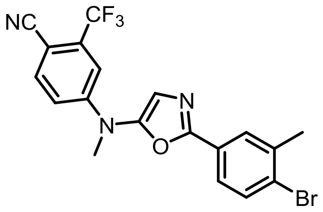

By general procedure E, using the diazo-substrate **3** and 4-bromo-3-methylbenzonitrile with $Rh_2(OAc)_4$ in CH_2Cl_2 followed by flash chromatography, eluting with 80:20 Hexane—EtOAc gave the desired oxazole (13.8 mg, 18%) as a colourless oil. R_f: 0.48 (80:20 Hexane—EtOAc); δ_H (400 MHz; $CDCl_3$) 7.86 (1H, d, *J* 1.5, bromomethylphenyl 2-H), 7.68 (1H, d, *J* 8.7, Ar 6-H), 7.67-7.61 (2H, m, bromomethylphenyl 5-H and bromomethylphenyl 6-H), 7.20 (1H, d, *J* 2.7, Ar 3-H), 7.04 (1H, dd, *J* 8.7 and 2.7, Ar 5-H), 6.96 (1H, s, oxazole 4-H), 3.44 (3H, s, NMe), 2.46 (3H, s, CMe); δ_C (101 MHz; $CDCl_3$) 158.5 (oxazole C-2), 149.9 (Ar C-4 or oxazole C-5), 149.6 (Ar C-4 or oxazole C-5), 139.0 (bromomethylphenyl C-3), 136.1 (Ar C-6), 134.5 (q, $^2J_{CF}$ 32, Ar C-2), 133.2 (bromomethylphenyl C-5 or bromomethylphenyl C-6), 128.3 (bromomethylphenyl C-2), 127.9 (bromomethylphenyl C-1 or bromomethylphenyl C-4), 126.2 (bromomethylphenyl C-1 or bromomethylphenyl C-4), 124.9 (bromomethylphenyl C-5 or bromomethylphenyl C-6), 122.5 (q, $^1J_{CF}$ 274, *C*F_3), 120.1 (oxazole C-4), 116.8 (Ar C-5), 116.2 (*C*N), 112.0 (q, $^3J_{CF}$ 5, Ar C-3), 100.0 (q, $^3J_{CF}$ 2, Ar C-1), 39.7 (N*Me*), 23.1 (C*Me*); ν_{max}/cm^{-1} (film) 2923, 2225, 1599, 1471, 1446, 1355, 1270, 1116, 1074; *m/z* (ES) [MH^+] 436.1 (100%, MH^+); HRMS Found: 436.0268 ($C_{19}H_{14}{}^{79}BrF_3N_3O$ requires *MH* 436.0266).

2-(Benzyloxy)-*N*-[4-cyano-3-(trifluoromethyl)phenyl]-*N*-methylacetamide 27

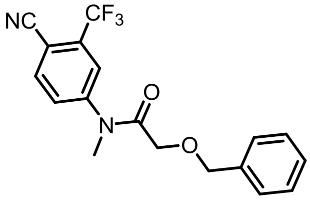

By general procedure E, using the diazo-substrate **3** and benzyl alcohol with $Rh_2(OAc)_4$ in CH_2Cl_2 followed by mass directed liquid chromatography purification, gave the desired ether (18.4 mg, 48%) as a colourless oil. δ_H (300 MHz; $CDCl_3$) 7.82 (1H, d, *J* 8.3, Ar 5-H), 7.68 (1H, d, *J* 2.1, Ar 2-H), 7.54 (1H, dd, *J* 8.3 and 2.1, Ar 6-H), 7.41 – 7.31 (3H, m, Ph), 7.22 – 7.16 (2H, m, Ph), 4.54 (2H, s, PhCH_2), 4.14 (2H, s, acetamide 2-H_2), 3.38 (3H, s, NMe); δ_C (75 MHz; $CDCl_3$) 168.9 (acetamide C-1), 147.2 (Ar C-1), 136.6 (Ph C-1), 135.7 (Ar C-5), 134.1 (q, $^2J_{CF}$ 35, Ar C-3), 129.2 (Ar C-6), 128.5 (Ph C-2 or C-3), 128.2 (Ph C-4), 128.0 (Ph C-2 or C-3), 124.2 (q, $^3J_{CF}$ 5, Ar C-2), 121.9 (q, $^1J_{CF}$ 275, *C*F_3), 114.9 (-*C*N), 108.0 (Ar C-4), 73.5 (acetamide C-2), 69.1 (O*C*H_2Ph), 37.1 (N*C*H_3); ν_{max}/cm^{-1} (film) 2861, 2231, 1675, 1327, 1313, 1129; *m/z* (ES) [MH^+] 371.3 (100%, MNa^+); HRMS Found: 371.0985 ($C_{18}H_{15}F_3N_2NaO_2$ requires *MNa* 371.0983).

N-[4-Cyano-3-(trifluoromethyl)phenyl]-N-methyl-2-(oxan-2-ylmethoxy)acetamide 28

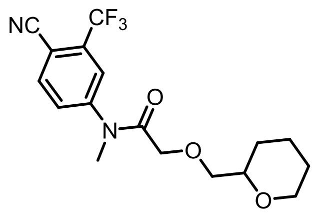

By general procedure E, using the diazo-substrate **3** and tetrahydropyran-2-methanol with $Rh_2(OAc)_4$ in CH_2Cl_2 followed by mass directed liquid chromatography purification, gave the desired ether (13.9 mg, 33%) as a colourless oil. δ_H (300 MHz; $CDCl_3$) 7.86 (1H, d, *J* 8.3, Ar 5-H), 7.72 (1H, d, *J* 2.0, Ar 2-H), 7.60 (1H, dd, *J* 8.3 and 2.0, Ar 6-H), 4.20 (1H, d, *J* 14.3, acetamide 2-Ha), 4.14 (1H, d, *J* 14.3, acetamide 2-Hb), 3.95 (1H, ddt, *J* 11.4, 3.4 and 1.7, pyran 2-H), 3.49 – 3.33 (4H, m, pyran 6-H_2 and methoxy CH_2), 3.37 (3H, s, NMe), 1.90 – 1.77 (1H, m, pyran 3-H_A), 1.76 – 1.60 (1H, m, pyran 3-H_B), 1.59 – 1.34 (3H, m, pyran 4-H_2 and 5-H_A), 1.33 – 1.10 (1H, m, pyran 5-H_B); δ_C (75 MHz; $CDCl_3$) 169.1 (acetamide C-1), 147.3 (Ar C-1), 135.8 (Ar C-5), 129.3 (Ar C-6), 124.3 (Ar C-2), 122.1 (q, $^1J_{CF}$ 269, CF_3), 114.9 (-*C*N), 107.9 (Ar C-4), 76.7 (pyran C-2), 75.1 (acetamide C-2), 70.6 (methoxyCH_2), 68.3 (pyran C-6), 37.1 (NCH_3), 27.8 (pyran C-3 or C-5), 25.8 (pyran C-3 or C-5), 22.9 (pyran C-4) *Ar C-3 not observed*; ν_{max}/cm^{-1} (film) 3084, 2851, 2231, 1666, 1128, 1088, 1049; *m/z* (ES) [MH^+] 357.4 (100%, MH^+); HRMS Found: 379.1257, ($C_{17}H_{19}F_3N_2NaO_3$ requires *MNa* 379.1245).

N-[4-Cyano-3-(trifluoromethyl)phenyl]-N-methyl-2-(oxan-4-ylmethoxy)acetamide 29

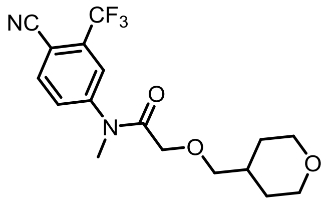

By general procedure E, using the diazo-substrate **3** and tetrahydropyran-4-methanol with $Rh_2(OAc)_4$ in CH_2Cl_2 followed by mass directed liquid chromatography purification, gave the desired ether (20.1 mg, 51%) as a colourless oil. δ_H (300 MHz; $CDCl_3$) 7.90 (1H, d, *J* 8.3, Ar 5-H), 7.75 (1H, d, *J* 2.0, Ar 2-H), 7.61 (1H, dd, *J* 8.3 and 2.0, Ar 6-H), 4.11 (2H, s, acetamide 2-H_2), 3.96 (2H, dd, *J* 10.5 and 4.5, pyran 2-Ha), 3.40 (3H, s, NMe), 3.35 (2H, dt, *J* 10.5 and 2.1, pyran 2-Hb), 3.30 (2H, d, *J* 6.5, OCH_2pyran) 1.91 – 1.70 (1H, m, pyran 4-H), 1.66 – 1.52 (2H, dd, *J* 13.4 and 2.1, pyran 3-Ha), 1.28 (2H, dtd, *J* 13.4, 12.1 and 4.5, pyran 3-Hb); δ_C (101 MHz; $CDCl_3$) 169.1 (acetamide C-1), 147.4 (Ar C-1), 135.9 (Ar C-5), 134.3 (q, $^2J_{CF}$ 33, Ar C-3), 129.7 (Ar C-6), 124.4 (Ar C-2), 122.0 (q, $^1J_{CF}$ 271, CF_3), 114.9 (-*C*N), 108.1 (Ar C-4), 77.4 (methoxyCH_2), 70.5 (acetamide C-2), 67.6 (pyran C-2), 37.2 (NCH_3), 35.5 (pyran C-4), 29.8 (pyran C-3); ν_{max}/cm^{-1} (film) 2916, 2846, 2231, 1673, 1608, 1130, 1088; *m/z* (ES) [MNa^+] 379.3 (100%, MNa^+); HRMS Found: 379.1254, ($C_{17}H_{19}F_3N_2NaO_3$ requires *MNa* 379.1245).

2-[(4-Chlorophenyl)methoxy]-*N*-[4-cyano-3-(trifluoromethyl)phenyl]-*N*-methylacetamide 30

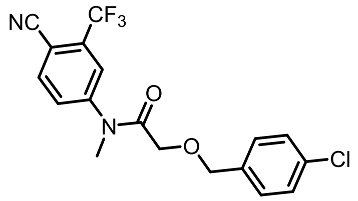

By general procedure E, using the diazo-substrate **3** and 4-chlorobenzyl alcohol with $Rh_2(OAc)_4$ in CH_2Cl_2 followed by mass directed liquid chromatography purification, gave the desired ether (10.4 mg, 20%) as a colourless oil. δ_H (300 MHz; $CDCl_3$) 7.84 (1H, d, *J* 8.3, Ar 5-H), 7.68 (1H, d, *J* 2.0, Ar 2-H), 7.55 (1H, dd, *J* 8.3 and 2.0, Ar 6-H), 7.31 (2H, d, *J* 8.5, chlorophenyl 3-H), 7.19 (2H, d, *J* 8.5, chlorophenyl 2-H), 4.50 (2H, s, OCH_2chlorophenyl), 4.11 (2H, s, acetamide 2-H_2), 3.37 (3H, s, NMe); δ_C (101 MHz; $CDCl_3$) 168.8 (acetamide C-1), 147.3 (Ar C-1), 135.9 (Ar C-5), 135.3 (C-1 chlorophenyl), 134.2 (Ar C-3), 134.1 (chlorophenyl C-4), 129.4 (Ar C-6 and C-2 chlorophenyl), 128.9 (C-3 chlorophenyl), 124.4 (Ar C-2), 114.9 (-*C*N), 106.3 (Ar C-4), 72.8 (acetamide C-2), 69.2 (methoxyCH_2), 37.2 (NCH_3), CF_3 *not observed*; ν_{max}/cm^{-1} (film) 3085, 3049, 2232, 1663, 1111; *m/z* (ES) [MNa^+] 405.4 (100%, MNa^+); HRMS Found: 405.0593, ($C_{18}H_{14}ClF_3N_2NaO_2$ requires *MNa* 405.0594).

***N*-[4-Cyano-3-(trifluoromethyl)phenyl]-2-(3,4-dihydro-2*H*-1-benzopyran-2-ylmethoxy)-*N*-methylacetamide 31**

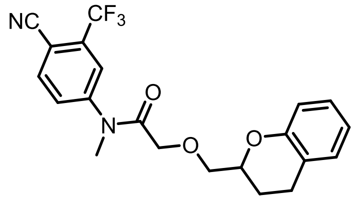

By general procedure E, using the diazo-substrate **3** and 3,4-dihydro-2*H*-chromen-2-yl-methanol with $Rh_2(OAc)_4$ in CH_2Cl_2 followed by mass directed liquid chromatography purification, gave the desired ether (17.6 mg, 40%) as a colourless oil. (300 MHz; $CDCl_3$) 7.84 (1H, d, *J* 8.3, Ar 5-H), 7.73 (1H, d, *J* 2.0, Ar 2-H), 7.62 (1H, dd, *J* 8.3 and 2.0, Ar 6-H), 7.09 (1H, td, *J* 7.4 and 1.8, benzopyran 6-H), 7.04 (1H, dt, *J* 8.1 and 1.2, benzopyran 7-H), 6.84 (1H, td, *J* 7.4 and 1.2, benzopyran 5-H), 6.74 (1H, dd, *J* 8.1 and 1.8, benzopyran 8-H), 4.27 (1H, d, *J* 14.2, acetamide 2-Ha), 4.25 (1H, d, *J* 14.2, acetamide 2-Hb), 4.20 – 4.10 (1H, m, benzopyran H-2), 3.71 (2H, ap d, *J* 4.8, methoxy CH_2), 3.40 (3H, s, NMe), 2.89 (1H, ddd, *J* 16.5, 10.9 and 5.9 benzopyran 4-H_A), 2.75 (1H, ddd, *J* 16.5, 5.7 and 3.4, benzopyran 4-H_B), 1.95 (1H, dddd, *J* 13.5, 5.9, 3.4 and 2.6, benzopyran 3-H_A), 1.76 (1H, dddd, *J* 13.5, 10.8, 9.8 and 5.7, benzopyran 3-H_B); δ_C (101 MHz; $CDCl_3$) 169.0 (acetamide C-1), 154.4 (benzopyran C-8a), 147.3 (Ar C-1), 135.9 (Ar C-5) 134.2 (q , $^2J_{CF}$, 32 Ar C-3) 129.7 (benzopyran), 129.5 (Ar C-6), 127.5 (benzopyran), 124.2 (q, $^3J_{CF}$ 5, Ar C-2), 121.9 (q, $^1J_{CF}$ 274, CF_3), 121.8 (benzopyran), 120.6 (benzopyran), 116.7 (benzopyran), 114.9 (-*C*N), 108.3 (Ar C-4), 75.0 (methoxy CH_2), 74.1 (acetamide C-2), 70.9 (benzopyran C-2), 37.2 (NCH_3), 24.4 (CH_2CH_2Ph), 24.1 (CH_2CH_2Ph); ν_{max}/cm^{-1} (film) 3079, 2929, 2231, 1677, 1178, 1131, 1002, 728; *m/z* (ES) [MH^+] 405.4 (100%, MH^+); HRMS Found: 405.1434, ($C_{21}H_{20}F_3N_2O_3$ requires *MH* 405.1426).

***N*-[4-Cyano-3-(trifluoromethyl)phenyl]-2-(cyclopropylmethoxy)-*N*-methylacetamide 32**

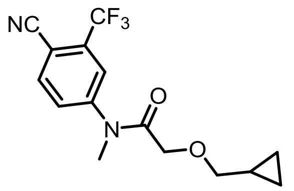

By general procedure E, using the diazo-substrate **3** and cyclopropanemethanol with $Rh_2(OAc)_4$ in CH_2Cl_2 followed by mass directed liquid chromatography purification, gave the desired ether (19.8 mg, 58%) as a colourless oil. δ_H (300 MHz; $CDCl_3$) 7.88 (1H, d, *J* 8.3, Ar 5-H), 7.74 (1H, d, *J* 2.1, Ar 2-H), 7.60 (1H, dd, *J* 8.3 and 2.1, Ar 6-H), 4.12 (2H, s, acetamide 2-H_2), 3.28 (3H, s, NMe), 3.28 (2H, d, *J* 7.0, methoxy CH_2), 1.07 – 0.79 (1H, m, cyclopropyl C*H*), 0.55 – 0.47 (2H, m, cyclopropyl CH_A), 0.19 – 0.11 (2H, m, cyclopropyl CH_B); δ_C (101 MHz; $CDCl_3$) 169.3 (acetamide C-1), 147.5 (Ar C-1), 135.9 (Ar C-5), 134.2 (q, $^2J_{CF}$ 33, Ar C-3), 129.3 (Ar C-6), 124.4 (q, $^3J_{CF}$ 5, Ar C-2), 122.0 (q, $^1J_{CF}$ 274, *C*F_3), 115.0 (-*C*N), 108.1 (Ar C-4), 76.4 (acetamide C-2), 69.8 (methoxy*C*H_2), 37.2 (N*C*H_3), 10.3 (cyclopropyl), 3.1 (cyclopropyl); ν_{max}/cm^{-1} (film) 3083, 3049, 2915, 2231, 1669, 1130, 1110; *m/z* (ES) [MNa^+] 335.4 (100%, MNa^+); HRMS Found: 335.0985, ($C_{15}H_{15}F_3N_2NaO_2$ requires *MNa* 335.0983).

***N*-[4-Cyano-3-(trifluoromethyl)phenyl]-*N*-methyl-2-(oxolan-2-ylmethoxy)acetamide 33**

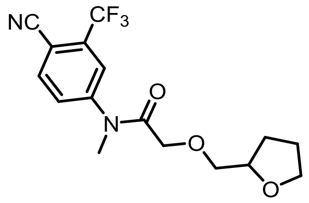

By general procedure E, using the diazo-substrate **3** and tetrahydrofurfuryl methanol with $Rh_2(OAc)_4$ in CH_2Cl_2 followed by mass directed liquid chromatography purification, gave the desired ether (19.8 mg, 53%) as a colourless oil. δ_H (500 MHz; $CDCl_3$) 7.87 (1H, d, *J* 8.3, Ar 5-H), 7.73 (1H, d, *J* 1.6, Ar 2-H), 7.61 (1H, dd, *J* 8.3 and 2, Ar 6-H), 4.21 (1H, d, *J* 14.1, acetamide 2-Ha), 4.16 (1H, d, *J* 14.1, acetamide 2-Hb), 4.01 – 3.89 (1H, m, tetrahydofuran 2-H), 3.81 (1H, dt, *J* 8.1 and 6.7, tetrahydrofuran 5-H_A), 3.73 (1H, dt, *J* 8.1 and 6.9, tetrahydrofuran 5-H_B), 3.54 (1H, dd, *J* 10.4 and 3.3, methoxy CH_A), 3.42 (1H, dd, *J* 10.4 and 6.7, methoxy CH_B), 3.38 (3H, s, NMe), 1.96 – 1.79 (3H, m, tetrahydrofuran 3-H_{AB} and 4-H_A), 1.58 – 1.47 (1H, m, tetrahydrofuran 4-H_B); δ_C (125 MHz; $CDCl_3$) 169.2 (acetamide C-1), 147.4 (Ar C-1), 135.9 (Ar C-5), 134.1 (Ar C-3), 129.4 (Ar C-6), 124.4 (Ar C-2), 121.8 (q, $^1J_{CF}$ 271, *C*F_3), 115.1 (-*C*N), 108.1 (Ar C-4), 77.9 (methoxy*C*H_2), 74.2 (tetrahydrofuran C-2), 70.6 (acetamide C-2), 68.5 (tetrahydrofuran C-5), 37.2 (N*C*H_3), 27.9 (tetrahydrofuran C-3), 25.7 (tetrahydrofuran C-4); ν_{max}/cm^{-1} (film) 3080, 3047, 2980, 2916, 2873, 2229, 1672, 1120; *m/z* (ES) [MNa^+] 365.2 (100%, MNa^+); HRMS Found: 343.1281, ($C_{16}H_{18}F_3N_2O_3$ requires *MH* 343.1270).

N-[4-Cyano-3-(trifluoromethyl)phenyl]-N-methyl-2-(4,4,4-trifluorobutoxy)acetamide 34

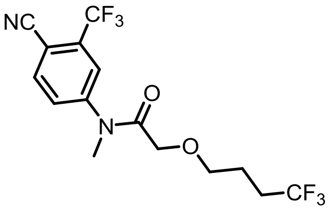

By general procedure E, using the diazo-substrate **3** and 4,4,4-trifluoro-1-butanol with $Rh_2(OAc)_4$ in CH_2Cl_2 followed by mass directed liquid chromatography purification, gave the desired ether (5.9 mg, 15%) as a colourless oil. δ_H (300 MHz; $CDCl_3$) 7.89 (1H, d, J 8.3, Ar 5-H), 7.72 (1H, dd, J 2.1, Ar 2-H), 7.59 (1H, dd, J 8.3 and 2.1, Ar 6-H), 4.09 (2H, s, acetamide CH_2), 3.52 (2H, t, J 6.1, butoxy CH_2), 3.38 (3H, s, NMe), 2.26 – 2.04 (2H, m, butyl 2-H_2), 1.86 – 1.72 (2H, m, butyl 3-H_2); δ_C (101 MHz; $CDCl_3$) 168.7 (acetamide C-1), 147.1 (Ar C-1), 135.9 (Ar C-5), 134.4 (q, $^2J_{CF}$ 33, Ar C-3), 129.2 (Ar C-6), 127.1 (q, $^1J_{CF}$ 276, butyl CF$_3$), 124.3 (Ar C-2), 121.8 (q, $^1J_{CF}$ 271, CF$_3$), 114.7 (-CN), 69.9 (butyl C-1 and (C(O)CH$_2$O)), 37.0 (NCH$_3$), 30.5 (q, $^2J_{CF}$ 29.2, butyl C-3), 22.3 (butyl C-2), *Ar C-4 not observed*; ν_{max}/cm^{-1} (film) 3056, 2875, 2232, 1675, 1264, 1139, 732; *m/z* (ES) [MNa$^+$] 391.2 (100%, MNa$^+$); HRMS Found: 391.0856, ($C_{15}H_{14}F_6N_2NaO_2$ requires *MNa* 391.0857),

1-*tert*-Butyl 2-methyl (2*S*,4*R*)-4-({[4-cyano-3-(trifluoromethyl)phenyl](methyl)carbamoyl}-methoxy)pyrrolidine-1,2-dicarboxylate 35

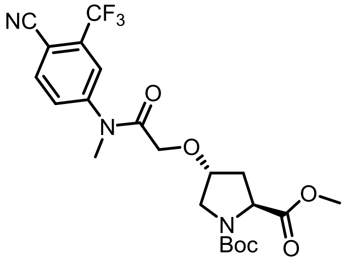

By general procedure E, using the diazo-substrate **3** and *N*-Boc-*trans*-4-hydroxy-L-proline methyl ester with $Rh_2(OAc)_4$ in CH_2Cl_2 followed by mass directed liquid chromatography purification, gave the desired ether (22.1 mg, 41%) as a colourless oil. δ_H (300 MHz; $CDCl_3$) 7.89 (1H, d, J 8.2, Ar 5-H), 7.71 (1H, d, J 2.0, Ar 2-H), 7.59 (1H, dd, J 8.2 and 2.0, Ar 6-H), 4.32 – 4.23 (1H, m, pyrrolidine 2-H), 4.19 – 3.97 (3H, m, C(O)CH_2O and pyrrolidine 4-H), 3.72 (3H, s, OMe), 3.60 (1H, d, J 11.7, pyrrolidine 5-H_A), 3.53 (1H, dd, J 11.7 and 4.1, pyrrolidine 5-H_B), 3.37 (3H, s, NMe), 2.39 – 2.18 (1H, m, pyrrolidine 3-H_A), 2.09 – 1.99 (1H, m, pyrrolidine 3-H_B), *1.45 (s, minor C(O)O^{t}Bu rotamer)*, 1.41 (9H, s, C(O)OC(CH_3)$_3$; δ_C (101 MHz; $CDCl_3$) *minor rotamer denoted in italics where possible* 173.5 (C(O)OMe)), *173.2* (C(O)OMe)), 168.6 (acetamide C-1), *154.3* (C(O)O^{t}Bu), 153.8 (C(O)O^{t}Bu), 147.2 (Ar C-1), 136.1 (Ar C-5), 134.4 (q, $^2J_{CF}$ 35, Ar C-3), 129.5 (Ar C-6), 124.5 (Ar C-2), 121.9 (q, $^1J_{CF}$ 270, CF$_3$), 114.9 (-CN), 108.4 (Ar C-4), 80.6 (OC(CH$_3$)$_3$), *78.5* (C(O)CH$_2$O), 77.8 (C(O)CH$_2$O), *68.4*, 68.1, 57.9, *57.5*, *52.4*, 52.2, *51.7*, 51.0, 37.2 (NCH$_3$), 36.7, *35.3*, *28.5*, 28.4; ν_{max}/cm^{-1} (film) 2978, 2232, 1745, 1685, 1397, 1130; *m/z* (ES) [MH$^+$] 386.2 (100%, M-(C(O)O^{t}Bu)H$^+$, 65%, MH$^+$); HRMS Found: 486.1856, ($C_{22}H_{27}F_3N_3O_6$ requires *MH* 486.1852).

N-[4-Cyano-3-(trifluoromethyl)phenyl]-2-(cyclohexyloxy)-N-methylacetamide 36

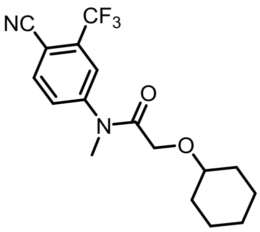

By general procedure E, using the diazo-substrate **3** and cyclohexanol with $Rh_2(OAc)_4$ in CH_2Cl_2 followed by mass directed liquid chromatography purification, gave the desired ether (11.6 mg, 31%). δ_H (300 MHz; $CDCl_3$) 7.88 (1H, d, *J* 8.3, Ar 5-H), 7.75 (1H, d, *J* 1.8, Ar 2-H), 7.62 (1H, dd, *J* 8.3 and 1.9, Ar 6-H), 4.12, (2H, s, C(O)CH_2O), 3.41(3H, s, NMe), 3.24 (1H, br s, OC*H* cyclohexyl), 1.78 (2H, br s, cyclohexyl), 1.66 (2H, br s, cyclohexyl), 1.51 (1H, br s, cyclohexyl), 1.32 – 1.10 (5H, m, cyclohexyl); δ_C (75 MHz; $CDCl_3$) 169.6 (acetamide C-1), 147.7 (Ar C-1), 135.7 (Ar C-5), 134.0 (q, $^2J_{CF}$ 32, Ar C-3), 129.2 (Ar C-6), 124.4 (q, $^3J_{CF}$ 5, Ar C-2), 121.9 (q, $^1J_{CF}$ 271, CF_3), 114.9 (-*C*N), 108.0 (Ar C-4), 78.6 (cyclohexyl C-1), 67.6 (acetamide C-2), 37.2 (NCH_3), 31.7 (cyclohexyl), 25.5 (cyclohexyl), 23.8 (cyclohexyl); ν_{max}/cm^{-1} (film) 2939, 2857, 2231, 1672, 1136, 1113, 1033; *m/z* (ES) [MH^+] 703.3 (100%, $[M]_2Na^+$); HRMS Found: 363.1298, ($C_{17}H_{19}F_3N_2NaO_2$ requires *MNa* 363.1296).

N-[4-Cyano-3-(trifluoromethyl)phenyl]-2-(2-methoxy-2-phenylethoxy)-N-methylacetamide 37

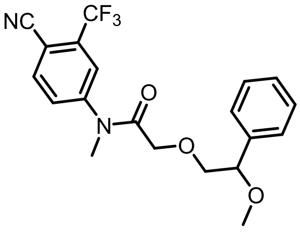

By general procedure E, using the diazo-substrate **3** and 2-methoxy-2-phenylethanol with $Rh_2(OAc)_4$ in CH_2Cl_2 followed by mass directed liquid chromatography purification, gave the desired ether (8.1 mg, 19%). δ_H (300 MHz; $CDCl_3$) 7.86 (1H, d, *J* 8.3, Ar 5-H), 7.72 (1H, d, *J* 2.0, Ar 2-H), 7.57 (1H, dd, *J* 8.3 and 2.0, Ar 6-H), 7.41 – 7.26 (5H, m, Ph), 4.35 (1H, dd, *J* 6.5 and 4.8, PhC*H*OMe), 4.24 (1H, d, *J* 14, acetamide 2-Ha), 4.14 (1H, d, *J* 14, acetamide 2-Hb), 3.66 – 3.58 (2H m, methoxy CH_{AB}), 3.36 (3H, s, OMe), 3.26 (3H, s, NMe); δ_C (75 MHz; $CDCl_3$) 169.1 (acetamide C-1), 147.3 (Ar C-1), 138.5 (C-1 phenyl), 135.9 (Ar C-5), 129.4 (Ar C-6), 128.7 (phenyl), 128.4 (C-4 phenyl), 127.1 (phenyl), 124.4 (Ar C-2), 114.9 (-*C*N), 107.9 (Ar C-4), 83.2 (MeO*C*HPh), 76.0 (methoxyCH_2), 70.8 (acetamide C-2), 56.9 (OCH_3), 37.2 (NCH_3), *Ar C-3 and CF_3 not observed*; ν_{max}/cm^{-1} (film) 3064, 2930, 2826, 2231, 1675, 1609, 1130; *m/z* (ES) [MNa^+] 415.3 (100%, MNa^+); HRMS Found: 393.1433, ($C_{20}H_{20}F_3N_2O_3$ requires *MH* 393.1426).

3.2 Chiral HPLC analysis for 3,4-Dihydro-*2H*-pyran-2-ylmethyl acetate, 19

Performed on an AS-H column by DAICEL, using anisocratic elution using 1% IPA in Hexane, 0.5 mL/min, for 60 min, detected at 210 nm. The *S*- enantiomer was found to have a retention time of 25.7 min, the *R*- enantiomer was found to have a retention time of 23.4 min.

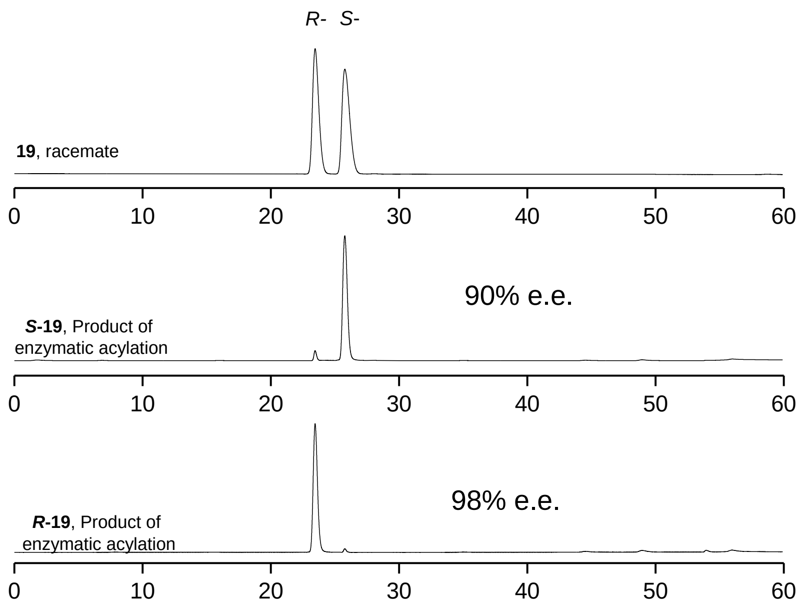

3.3 Chiral HPLC analysis for *N*-[4-cyano-3-(trifluoromethyl)phenyl]-(3,4-dihydro-2*H*-pyran-2-ylmethoxy)-*N*-methylacetamide, 13

Performed on an AD-H column by DAICEL, using an isocratic elution using 5 % IPA in Hexane, 1.0 mL/min, for 60 min, detected at 254 nm. The *S*- enantiomer was found to have a retention time of 29.4 min, the *R*- enantiomer was found to have a retention time of 33.2 min.

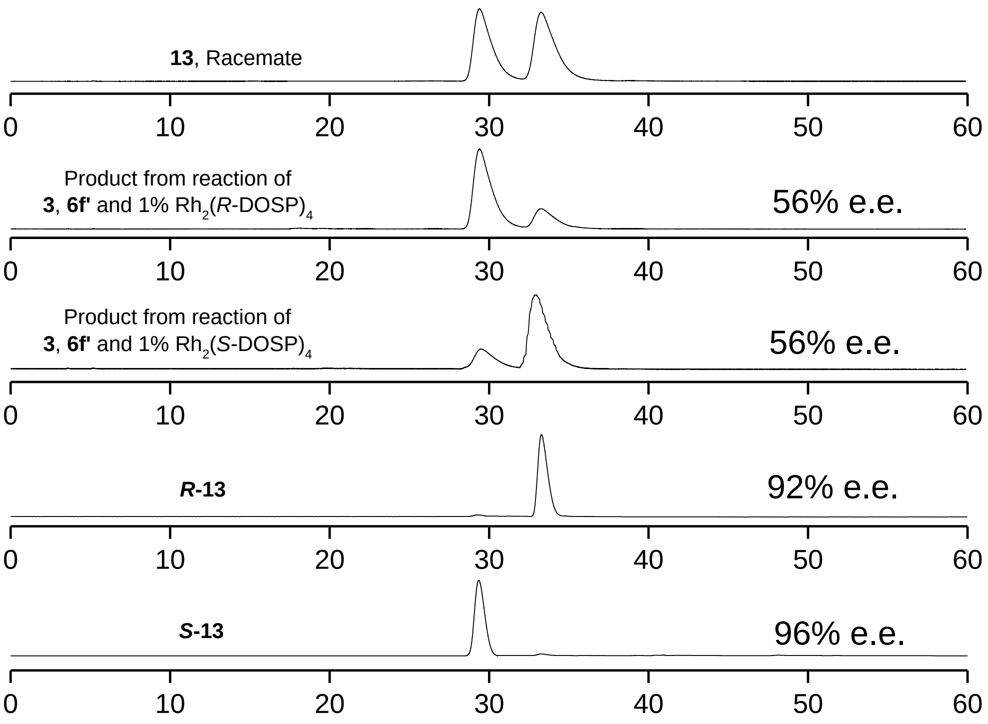

4 Extended Table 1: Reaction conditions and reaction outcomes for the selected scaled-up reactions

Round[a]	Reaction conditions	Product (Yield)[b]	EC_{50}[c]
1	**3**, **6f** (10 eq.), 1 mol% Rh_2(*S*-DOSP)$_4$, CH_2Cl_2	**7** (80%)	8.8 ± 0.7 μM
		**17** (11%)	>100 μM
1	**3**, **6a** (10 eq.), 1 mol% Rh_2(*S*-DOSP)$_4$, CH_2Cl_2	**8** (71%)	7.3 ± 0.2 μM
		**17** (12%)	See above
		**18** (12%)	>500 μM
2	**3**, **6o** (10 eq.), 1 mol% Rh_2(*S*-DOSP)$_4$, CH_2Cl_2	**10** (82%)	4.7 ± 0.1 μM
		**17** (13%)	see above
2	**3**, **6m** (10 eq.), 1 mol% Rh_2(*S*-DOSP)$_4$, CH_2Cl_2	**11** (76%)	4.9 ± 0.1 μM
		**17** (11%)	see above
		**18** (8%)	see above
2	**3**, **6r** (10 eq.), 1 mol% Rh_2(esp)$_2$, CH_2Cl_2	**12** (73%)	3.8 ± 0.2 μM
		**17** (14%)	see above
		**18** (13%)	see above
2	**5**, **6f** (10 eq.), 1 mol% Rh_2(OAc)$_4$, CH_2Cl_2	**20** (54%)	12.6 ± 0.9 μM
		**9** (19%)	790 ± 60 nM
2	**5**, **6n** (10 eq.), 1 mol% Rh_2(OAc)$_4$, CH_2Cl_2	**5** (67%)	>500 μM
		**9** (18%)	790 ± 60 nM
3	**3**, **6f’** (10 eq.), 1 mol% Rh_2(*R*-DOSP)$_4$, CH_2Cl_2	**13** (73%; 56% ee)	1.1 ± 0.1 μM
		**17** (10%)	see above
		**18** (12%)	see above
3	**5, 6e’**(10 eq.), 1 mol%Rh_2(OAc)$_4$, CH_2Cl_2	**14** (75%)	730 ± 30 nM
		**17** (11%)	see above
		**18** (8%)	see above

[a] Round of activity-directed synthesis (see text). [b] Yield of purified product. [c] Dose-dependent activity of the purified product.

5 Limited SAR study

5.1 Extended Table 2: Structure Activity Data for oxazole and ether series identified by ADS

Entry	Compound	Structure	AlogP[a]	MW	EC_{50} (μM)[b]
1	**21**	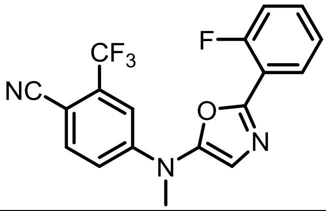	4.56	361.3	92 ± 5
2	**22**	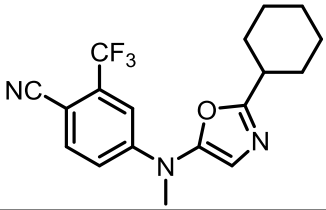	4.81	349.4	> 150
3	**23**	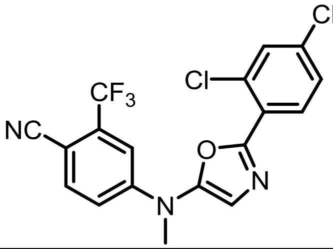	5.68	412.2	10.4 ± 0.6
4	**24**	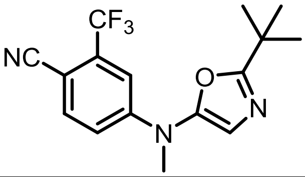	4.24	323.3	> 200
5	**25**	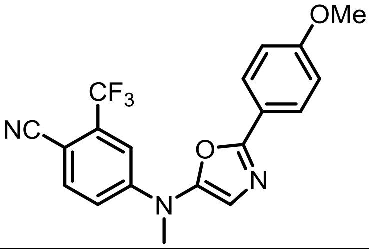	4.34	373.3	1.3 ± 0.1
6	**26**	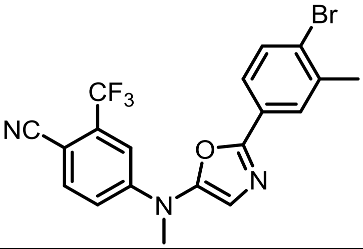	5.58	436.2	49 ± 5
7	**27**	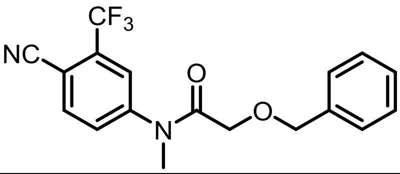	3.30	348.3	> 200
8	**28**	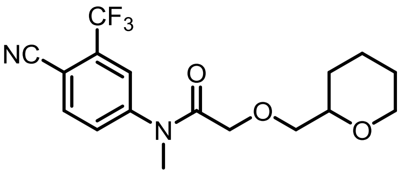	2.53	356.3	1.8 ± 0.1

9	**29**	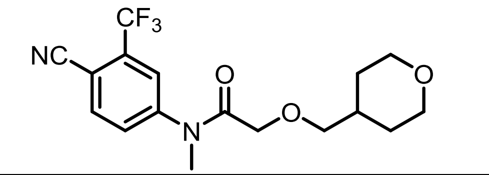	2.13	356.3	> 150
10	**30**	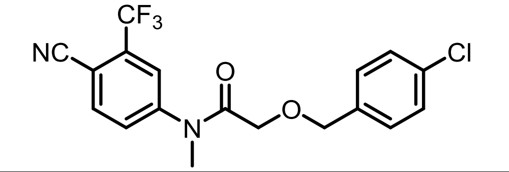	3.96	382.8	> 200
11	**31**	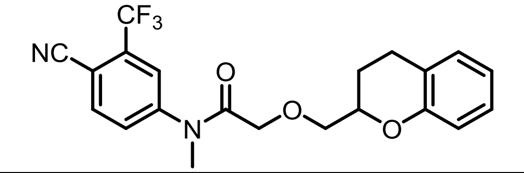	3.91	404.4	34 ± 1
12	**32**	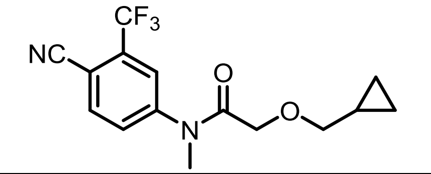	2.53	312.3	> 100
13	**33**	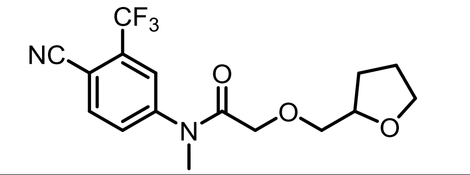	2.07	342.3	3.2 ± 0.2
14	**34**	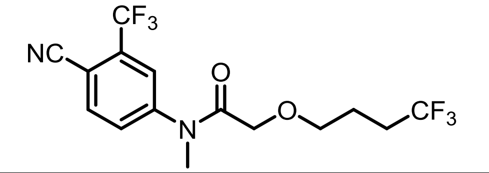	3.30	368.3	> 200
15	**35**	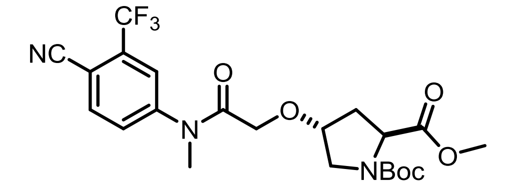	2.43	485.5	> 200
16	**36**	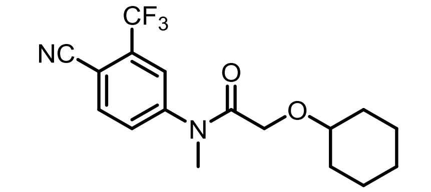	3.57	340.3	> 200
17	**37**	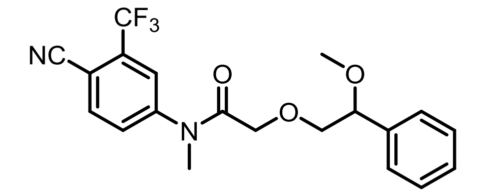	3.20	392.3	61 ± 4

[a] Atom based logP prediction calculated in Pipeline Pilot. [b] Dose-dependent activity of the purified product.

5.2 Activity and molecular properties of the analogues prepared.

The activity of compounds is denoted by the size of the circles (amides, black; oxazoles, red).

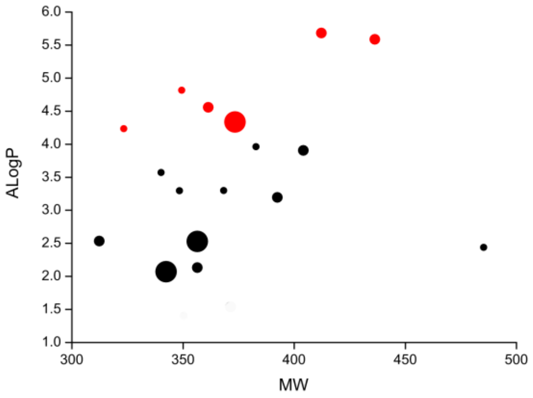

6 Dose-response curves for individual molecules:

EC_{50} values were obtained by performing logistic fits to the appropriate dose-response curves using the OriginPro v.8.6 software, utilising the linear regression equation:

$$y = A_1 + \frac{A_2 - A_1}{1 + 10^{\log(x_0 - x) \times p}}$$

The values of the parameters after fitting are shown in the inset boxes on each graph.

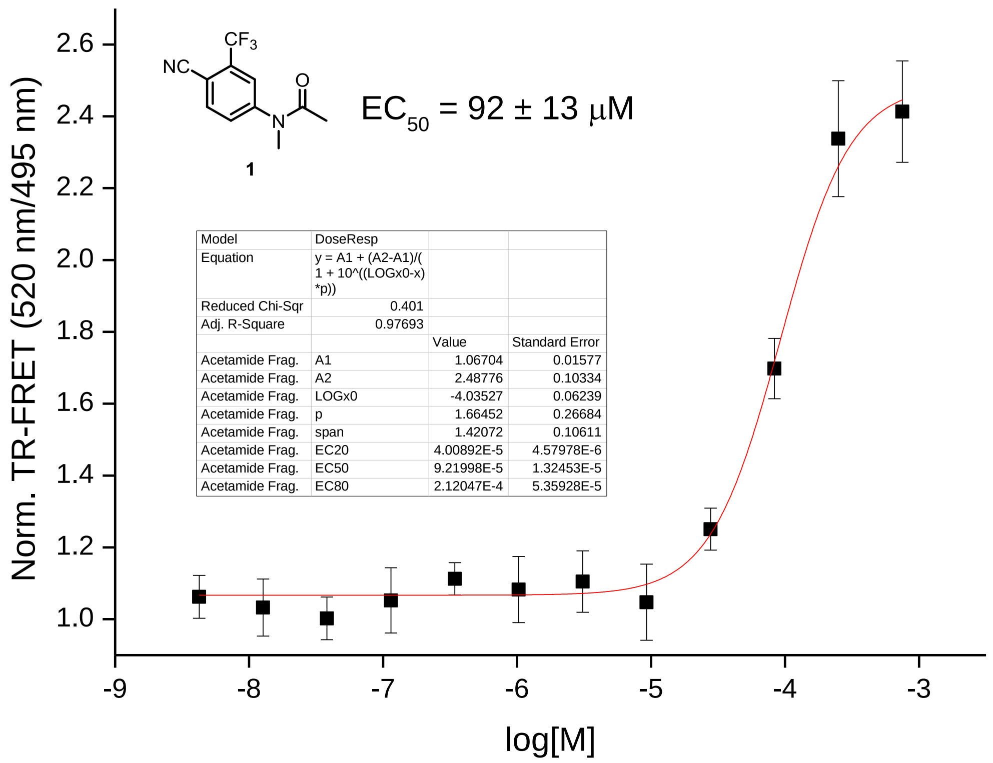

Model	DoseResp		
Equation	y = A1 + (A2-A1)/(1 + 10^((LOGx0-x) *p))		
Reduced Chi-Sqr		0.401	
Adj. R-Square		0.97693	
		Value	Standard Error
Acetamide Frag.	A1	1.06704	0.01577
Acetamide Frag.	A2	2.48776	0.10334
Acetamide Frag.	LOGx0	-4.03527	0.06239
Acetamide Frag.	p	1.66452	0.26684
Acetamide Frag.	span	1.42072	0.10611
Acetamide Frag.	EC20	4.00892E-5	4.57978E-6
Acetamide Frag.	EC50	9.21998E-5	1.32453E-5
Acetamide Frag.	EC80	2.12047E-4	5.35928E-5

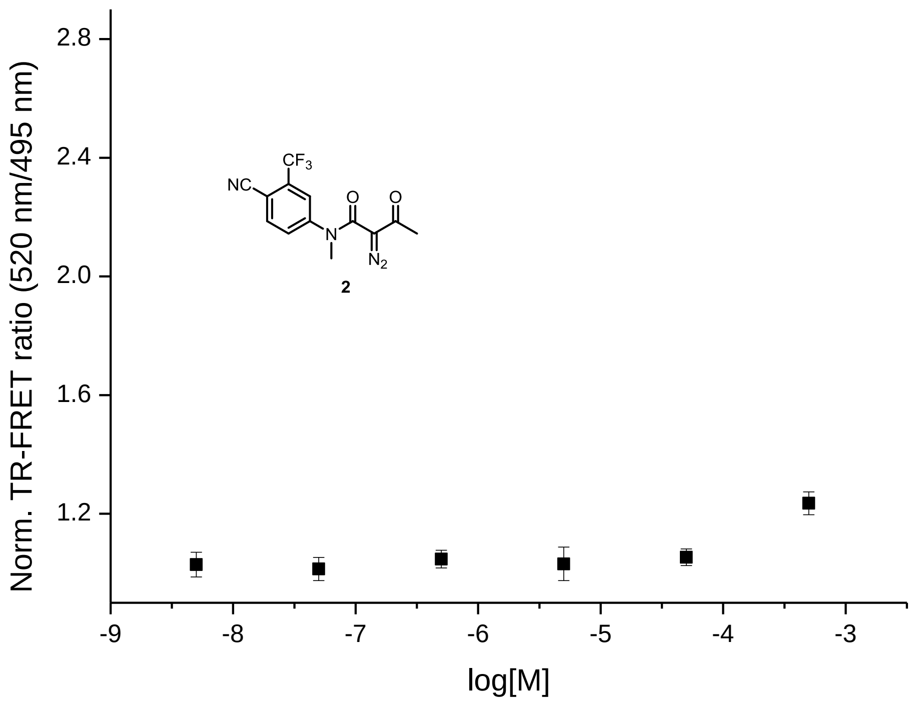

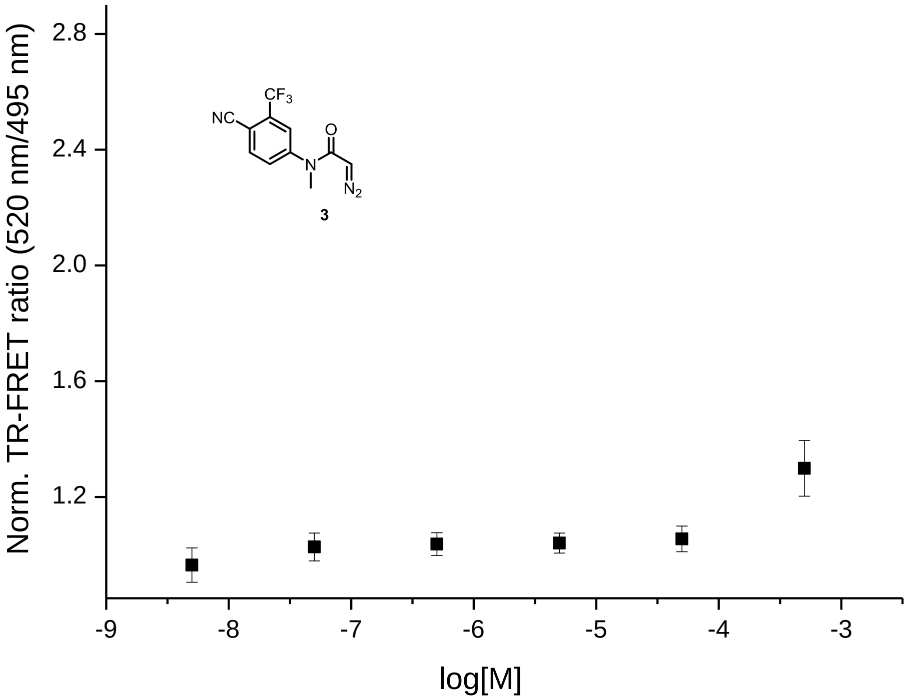

Norm. TR-FRET ratio (520 nm/495 nm)
2.8
2.4
2.0
1.6
1.2
-9
-8
-7
-6
-5
-4
-3
log[M]
NC
CF3
O
N
N2
3

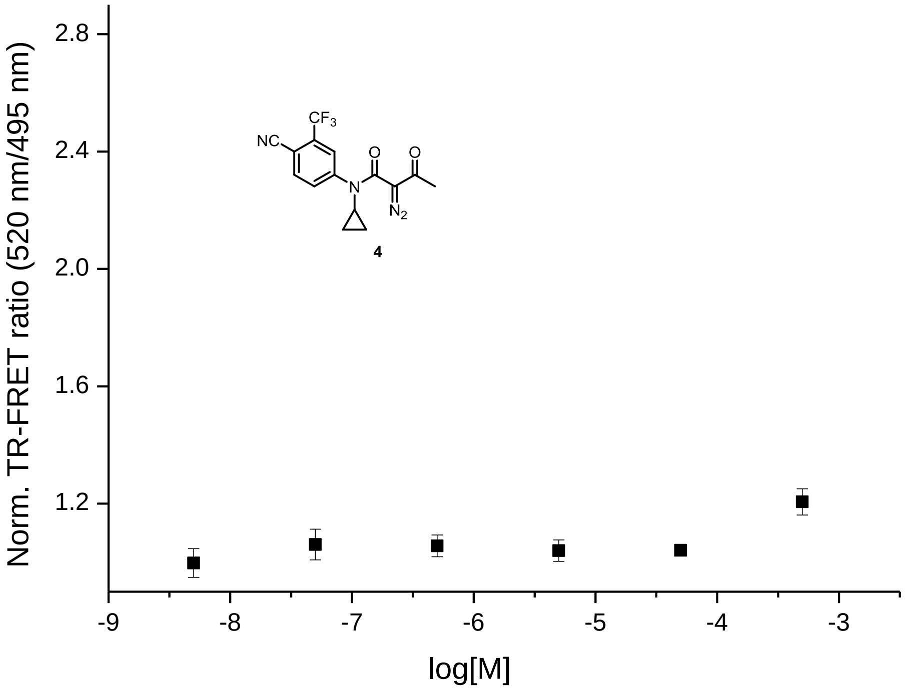

Norm. TR-FRET ratio (520 nm/495 nm)
2.8
2.4
2.0
1.6
1.2
-9
-8
-7
-6
-5
-4
-3
log[M]
NC
CF3
O
O
N
N2
4

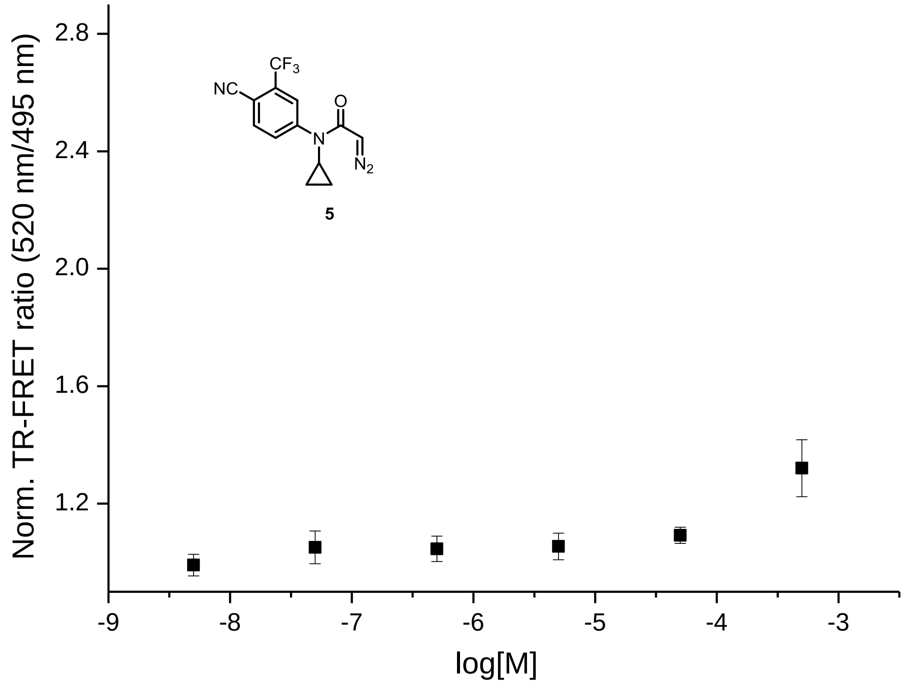
Norm. TR-FRET ratio (520 nm/495 nm)
log[M]
NC
CF3
O
N
N2
5

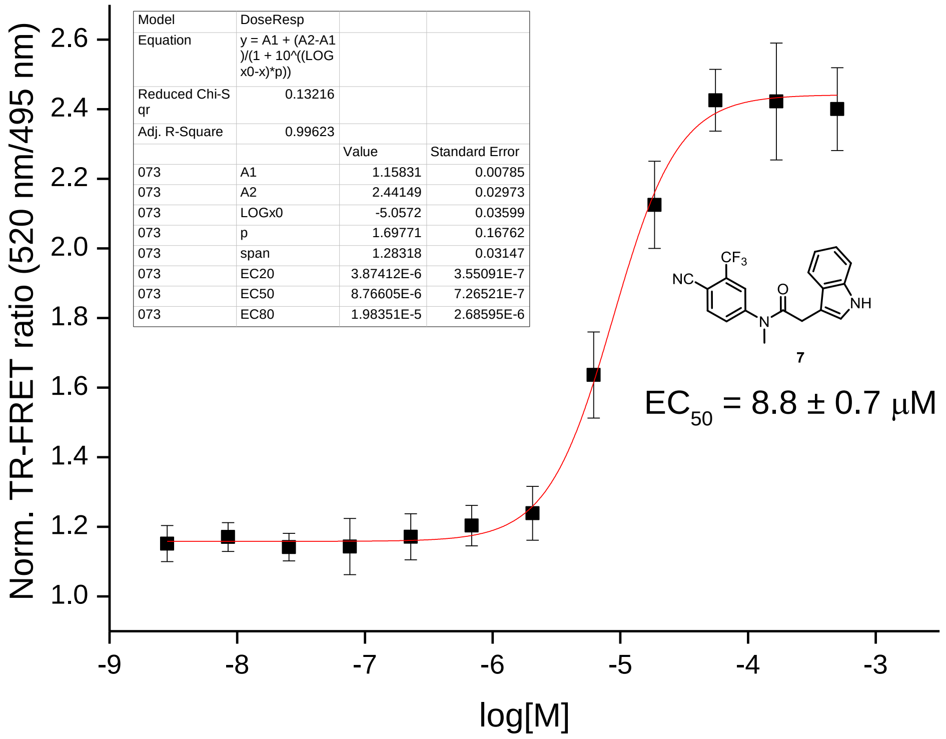
Model DoseResp
Equation y = A1 + (A2-A1)/(1 + 10^((LOGx0-x)*p))
Reduced Chi-Sqr 0.13216
Adj. R-Square 0.99623
Value Standard Error
073 A1 1.15831 0.00785
073 A2 2.44149 0.02973
073 LOGx0 -5.0572 0.03599
073 p 1.69771 0.16762
073 span 1.28318 0.03147
073 EC20 3.87412E-6 3.55091E-7
073 EC50 8.76605E-6 7.26521E-7
073 EC80 1.98351E-5 2.68595E-6
NC
CF3
O
N
NH
7
EC50 = 8.8 ± 0.7 μM
Norm. TR-FRET ratio (520 nm/495 nm)
log[M]

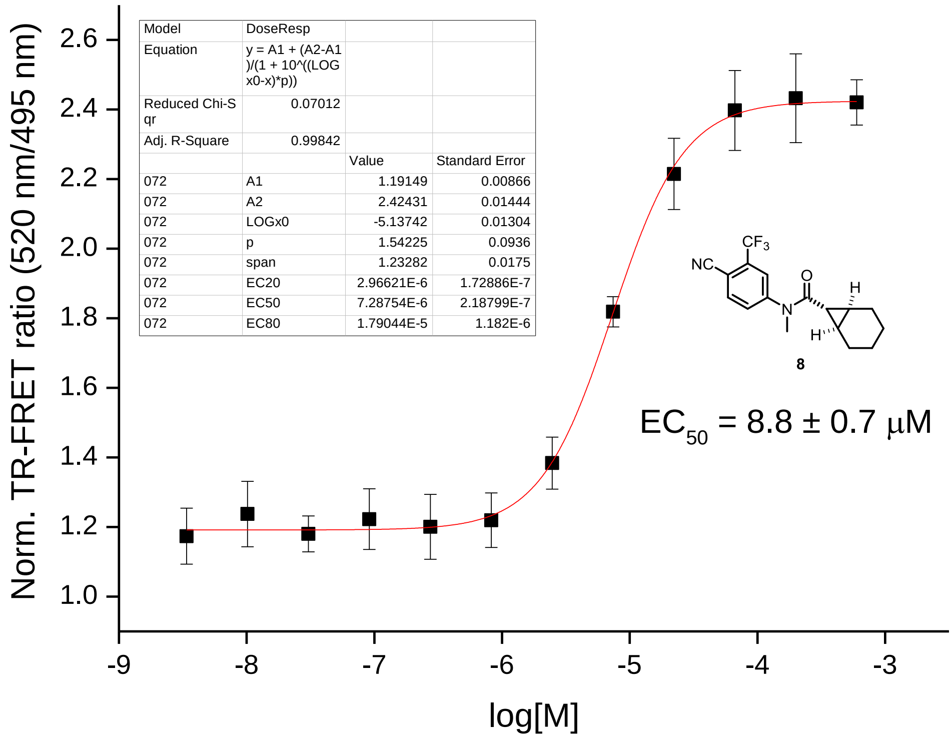
Model | DoseResp
Equation | y = A1 + (A2-A1)/(1 + 10^((LOGx0-x)*p))
Reduced Chi-Sqr | 0.07012
Adj. R-Square | 0.99842
Value | Standard Error
072 | A1 | 1.19149 | 0.00866
072 | A2 | 2.42431 | 0.01444
072 | LOGx0 | -5.13742 | 0.01304
072 | p | 1.54225 | 0.0936
072 | span | 1.23282 | 0.0175
072 | EC20 | 2.96621E-6 | 1.72886E-7
072 | EC50 | 7.28754E-6 | 2.18799E-7
072 | EC80 | 1.79044E-5 | 1.182E-6
CF3
NC
O
N
H
H
8
EC50 = 8.8 ± 0.7 μM
Norm. TR-FRET ratio (520 nm/495 nm)
2.6
2.4
2.2
2.0
1.8
1.6
1.4
1.2
1.0
-9
-8
-7
-6
-5
-4
-3
log[M]

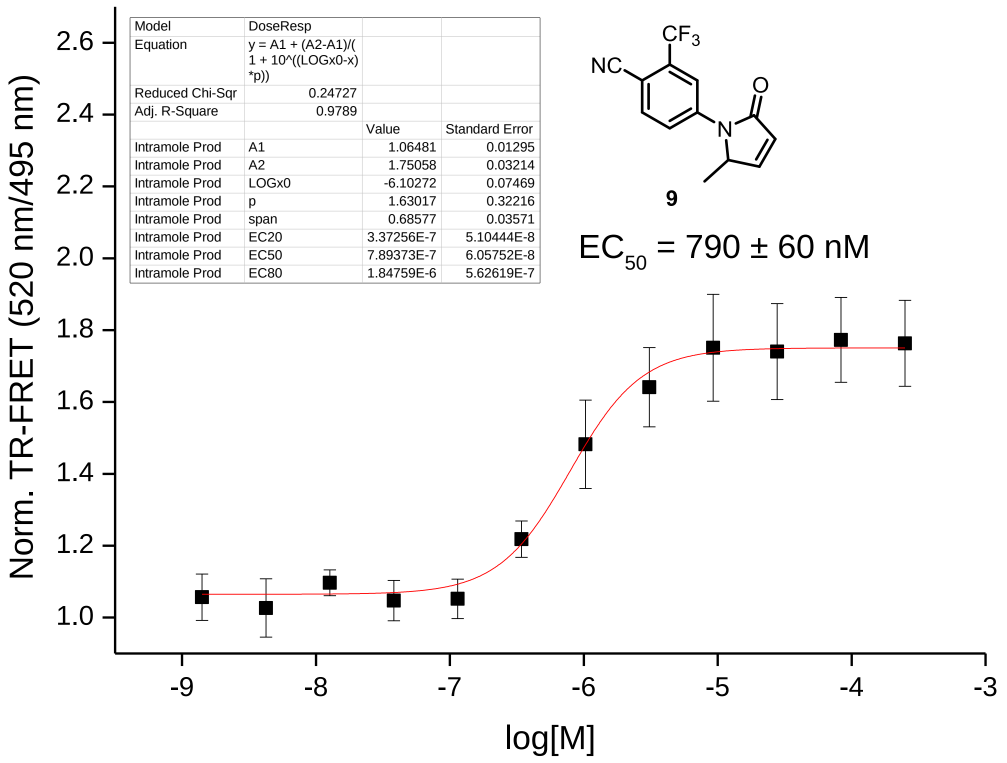
Model | DoseResp
Equation | y = A1 + (A2-A1)/(1 + 10^((LOGx0-x)*p))
Reduced Chi-Sqr | 0.24727
Adj. R-Square | 0.9789
Value | Standard Error
Intramole Prod | A1 | 1.06481 | 0.01295
Intramole Prod | A2 | 1.75058 | 0.03214
Intramole Prod | LOGx0 | -6.10272 | 0.07469
Intramole Prod | p | 1.63017 | 0.32216
Intramole Prod | span | 0.68577 | 0.03571
Intramole Prod | EC20 | 3.37256E-7 | 5.10444E-8
Intramole Prod | EC50 | 7.89373E-7 | 6.05752E-8
Intramole Prod | EC80 | 1.84759E-6 | 5.62619E-7
CF3
NC
O
N
9
EC50 = 790 ± 60 nM
Norm. TR-FRET (520 nm/495 nm)
2.6
2.4
2.2
2.0
1.8
1.6
1.4
1.2
1.0
-9
-8
-7
-6
-5
-4
-3
log[M]

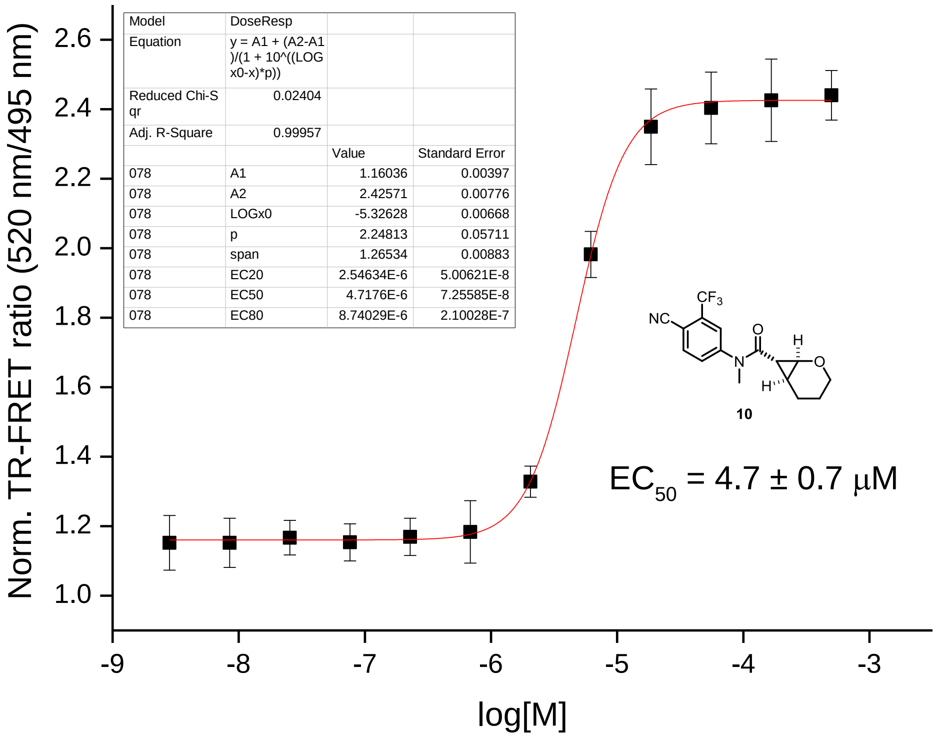
Model DoseResp
Equation y = A1 + (A2-A1)/(1 + 10^((LOGx0-x)*p))
Reduced Chi-Sqr 0.02404
Adj. R-Square 0.99957
Value Standard Error
078 A1 1.16036 0.00397
078 A2 2.42571 0.00776
078 LOGx0 -5.32628 0.00668
078 p 2.24813 0.05711
078 span 1.26534 0.00883
078 EC20 2.54634E-6 5.00621E-8
078 EC50 4.7176E-6 7.25585E-8
078 EC80 8.74029E-6 2.10028E-7
NC
CF3
O
N
H
O
H
10
EC50 = 4.7 ± 0.7 μM
Norm. TR-FRET ratio (520 nm/495 nm)
2.6
2.4
2.2
2.0
1.8
1.6
1.4
1.2
1.0
-9
-8
-7
-6
-5
-4
-3
log[M]

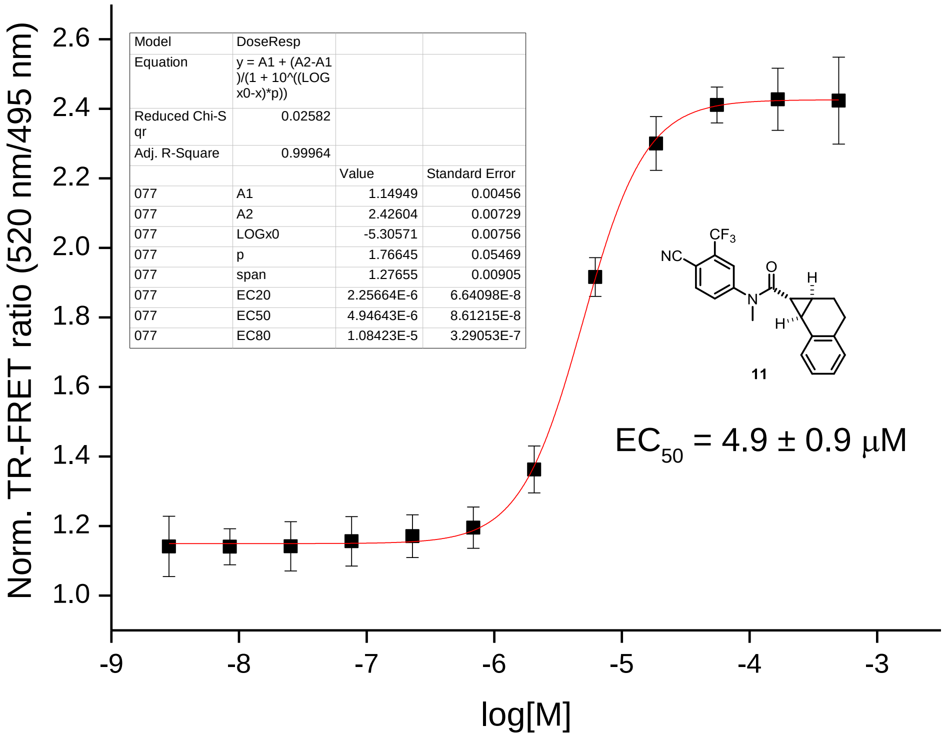
Model DoseResp
Equation y = A1 + (A2-A1)/(1 + 10^((LOGx0-x)*p))
Reduced Chi-Sqr 0.02582
Adj. R-Square 0.99964
Value Standard Error
077 A1 1.14949 0.00456
077 A2 2.42604 0.00729
077 LOGx0 -5.30571 0.00756
077 p 1.76645 0.05469
077 span 1.27655 0.00905
077 EC20 2.25664E-6 6.64098E-8
077 EC50 4.94643E-6 8.61215E-8
077 EC80 1.08423E-5 3.29053E-7
NC
CF3
O
N
H
H
11
EC50 = 4.9 ± 0.9 μM
Norm. TR-FRET ratio (520 nm/495 nm)
2.6
2.4
2.2
2.0
1.8
1.6
1.4
1.2
1.0
-9
-8
-7
-6
-5
-4
-3
log[M]

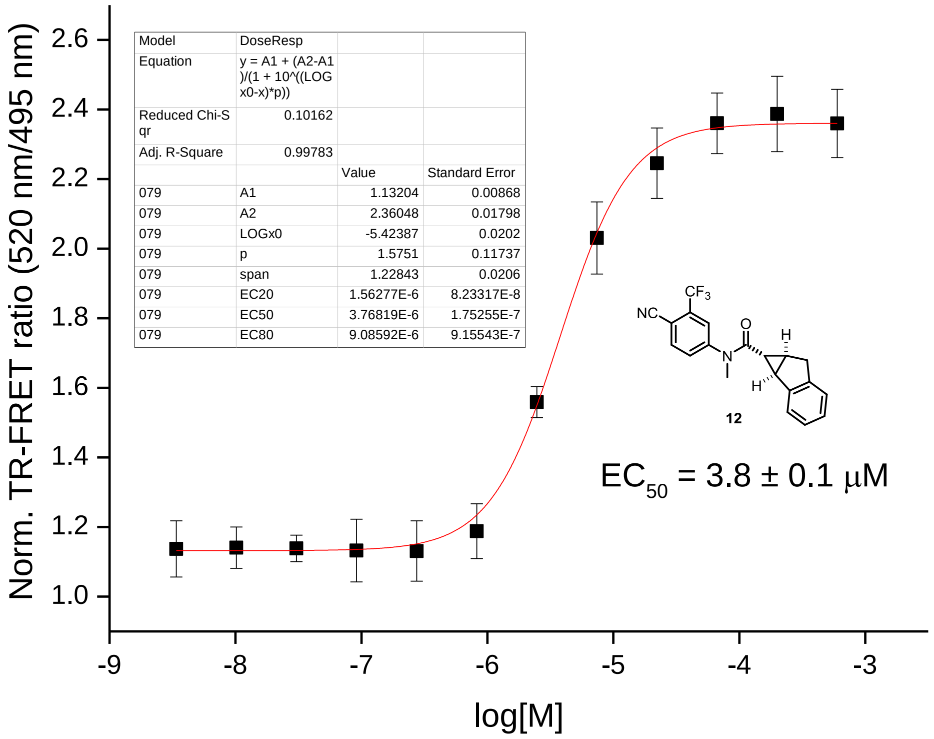

Model DoseResp
Equation y = A1 + (A2-A1)/(1 + 10^((LOGx0-x)*p))
Reduced Chi-Sqr 0.10162
Adj. R-Square 0.99783
Value Standard Error
079 A1 1.13204 0.00868
079 A2 2.36048 0.01798
079 LOGx0 -5.42387 0.0202
079 p 1.5751 0.11737
079 span 1.22843 0.0206
079 EC20 1.56277E-6 8.23317E-8
079 EC50 3.76819E-6 1.75255E-7
079 EC80 9.08592E-6 9.15543E-7
NC
CF3
O
N
H
H
12
EC50 = 3.8 ± 0.1 μM
Norm. TR-FRET ratio (520 nm/495 nm)
log[M]

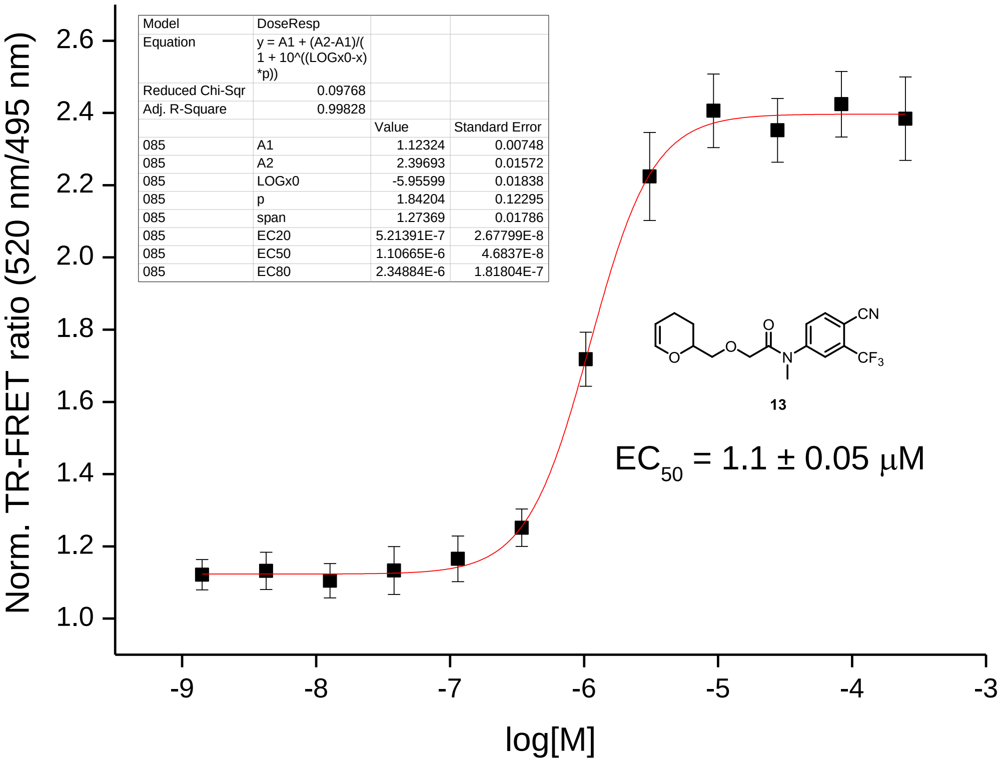

Model DoseResp
Equation y = A1 + (A2-A1)/(1 + 10^((LOGx0-x)*p))
Reduced Chi-Sqr 0.09768
Adj. R-Square 0.99828
Value Standard Error
085 A1 1.12324 0.00748
085 A2 2.39693 0.01572
085 LOGx0 -5.95599 0.01838
085 p 1.84204 0.12295
085 span 1.27369 0.01786
085 EC20 5.21391E-7 2.67799E-8
085 EC50 1.10665E-6 4.6837E-8
085 EC80 2.34884E-6 1.81804E-7
O
O
O
N
CN
CF3
13
EC50 = 1.1 ± 0.05 μM
Norm. TR-FRET ratio (520 nm/495 nm)
log[M]

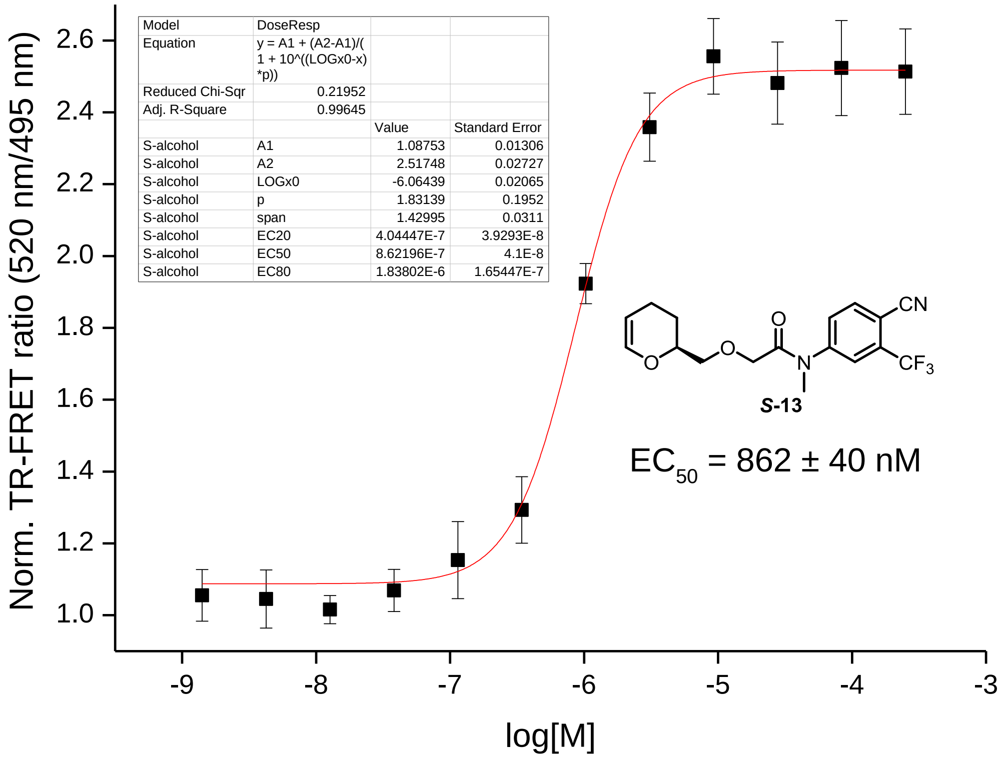

Model DoseResp
Equation y = A1 + (A2-A1)/(1 + 10^((LOGx0-x) *p))
Reduced Chi-Sqr 0.21952
Adj. R-Square 0.99645
Value Standard Error
S-alcohol A1 1.08753 0.01306
S-alcohol A2 2.51748 0.02727
S-alcohol LOGx0 -6.06439 0.02065
S-alcohol p 1.83139 0.1952
S-alcohol span 1.42995 0.0311
S-alcohol EC20 4.04447E-7 3.9293E-8
S-alcohol EC50 8.62196E-7 4.1E-8
S-alcohol EC80 1.83802E-6 1.65447E-7
CN
O
O
N
CF3
S-13
EC50 = 862 ± 40 nM
Norm. TR-FRET ratio (520 nm/495 nm)
log[M]

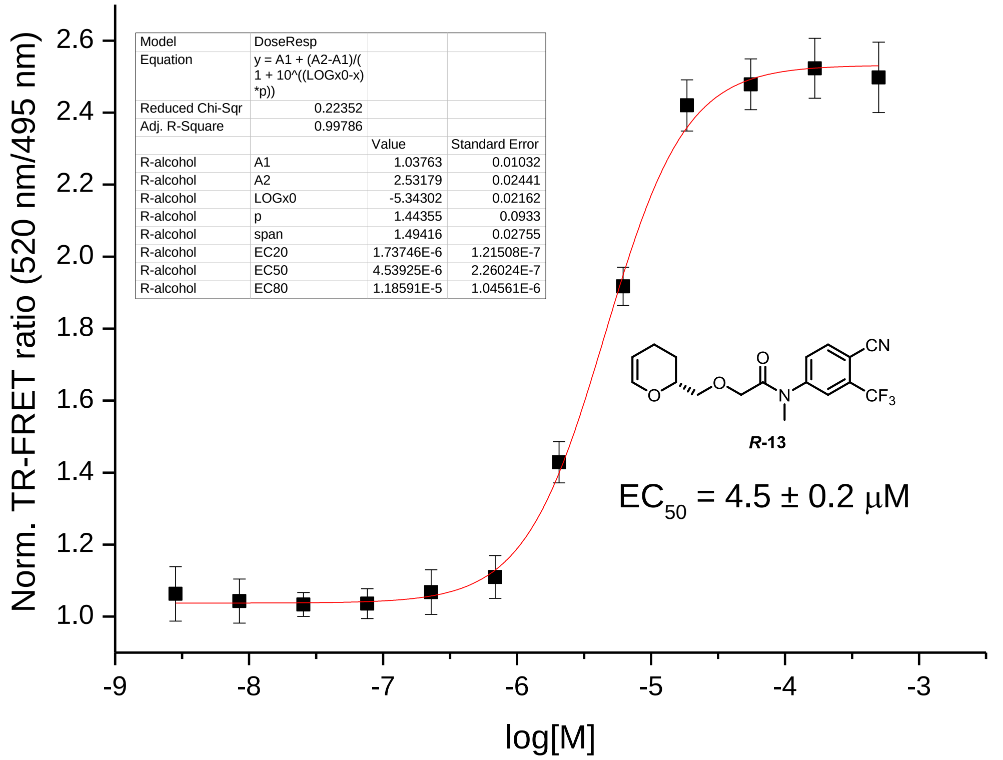

Model DoseResp
Equation y = A1 + (A2-A1)/(1 + 10^((LOGx0-x) *p))
Reduced Chi-Sqr 0.22352
Adj. R-Square 0.99786
Value Standard Error
R-alcohol A1 1.03763 0.01032
R-alcohol A2 2.53179 0.02441
R-alcohol LOGx0 -5.34302 0.02162
R-alcohol p 1.44355 0.0933
R-alcohol span 1.49416 0.02755
R-alcohol EC20 1.73746E-6 1.21508E-7
R-alcohol EC50 4.53925E-6 2.26024E-7
R-alcohol EC80 1.18591E-5 1.04561E-6
CN
O
O
N
CF3
R-13
EC50 = 4.5 ± 0.2 μM
Norm. TR-FRET ratio (520 nm/495 nm)
log[M]

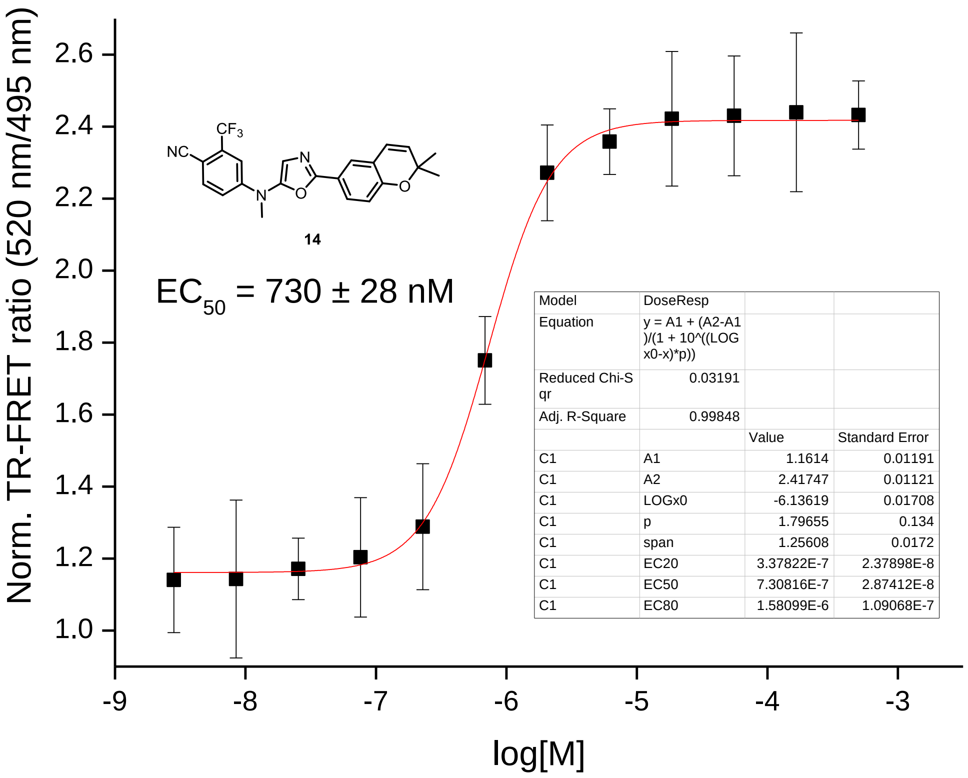

Model	DoseResp		
Equation	y = A1 + (A2-A1)/(1 + 10^((LOGx0-x)*p))		
Reduced Chi-Sqr	0.03191		
Adj. R-Square	0.99848		
		Value	Standard Error
C1	A1	1.1614	0.01191
C1	A2	2.41747	0.01121
C1	LOGx0	-6.13619	0.01708
C1	p	1.79655	0.134
C1	span	1.25608	0.0172
C1	EC20	3.37822E-7	2.37898E-8
C1	EC50	7.30816E-7	2.87412E-8
C1	EC80	1.58099E-6	1.09068E-7

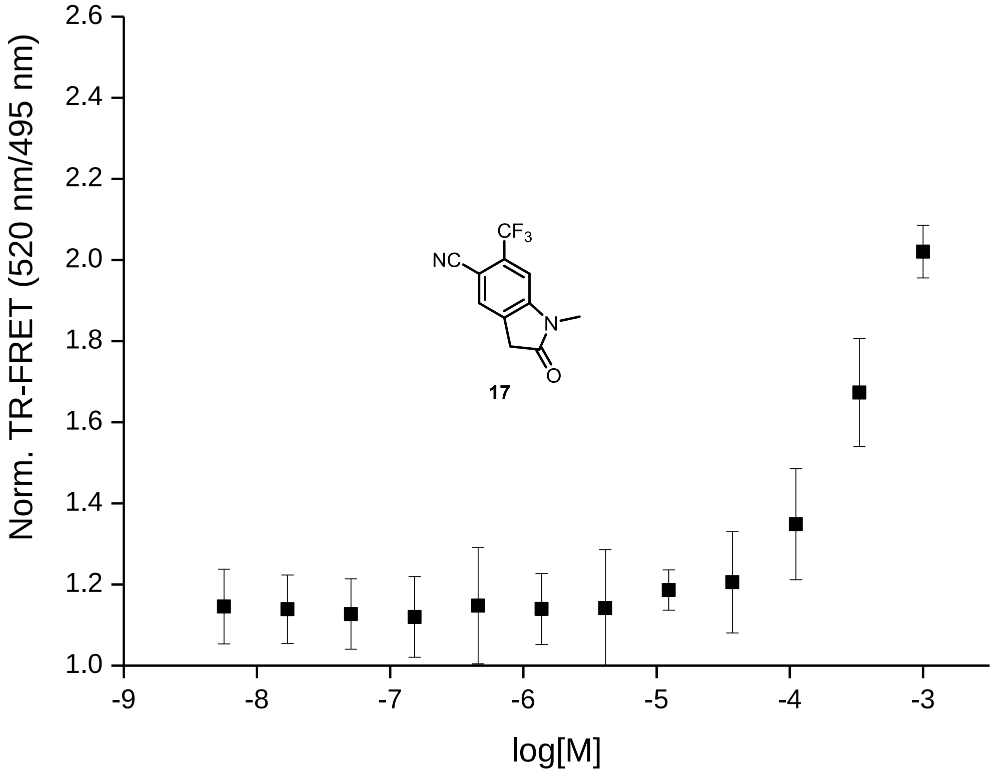

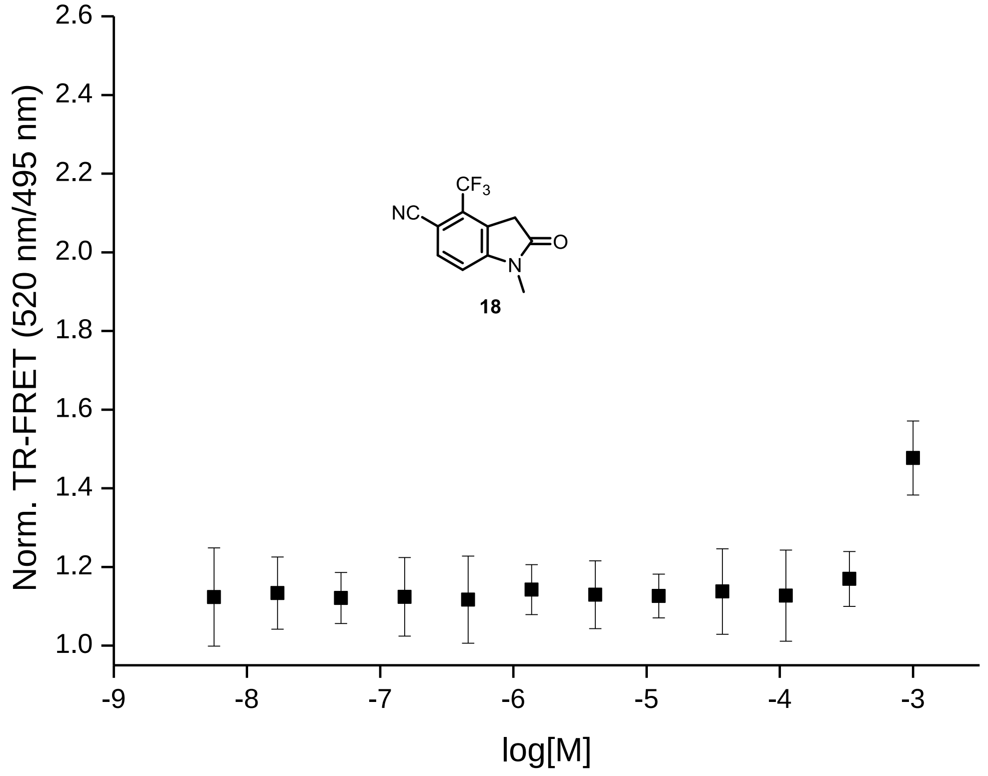

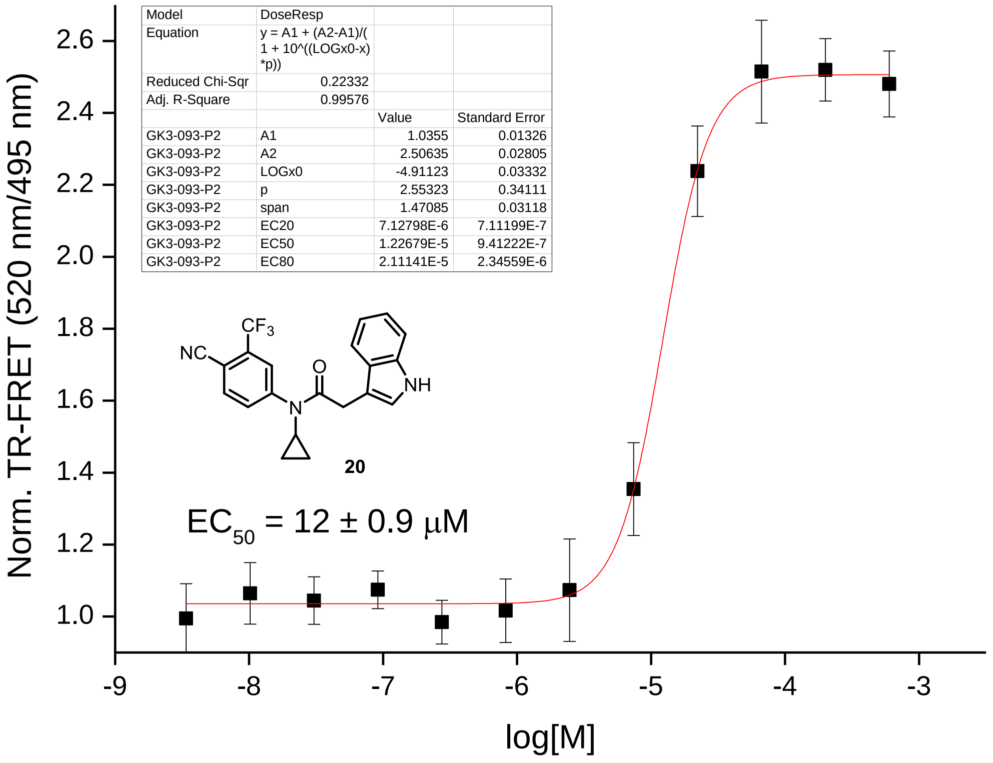

Model	DoseResp		
Equation	y = A1 + (A2-A1)/(1 + 10^((LOGx0-x) *p))		
Reduced Chi-Sqr	0.22332		
Adj. R-Square	0.99576		
		Value	Standard Error
GK3-093-P2	A1	1.0355	0.01326
GK3-093-P2	A2	2.50635	0.02805
GK3-093-P2	LOGx0	-4.91123	0.03332
GK3-093-P2	p	2.55323	0.34111
GK3-093-P2	span	1.47085	0.03118
GK3-093-P2	EC20	7.12798E-6	7.11199E-7
GK3-093-P2	EC50	1.22679E-5	9.41222E-7
GK3-093-P2	EC80	2.11141E-5	2.34559E-6

Oxazole **21**

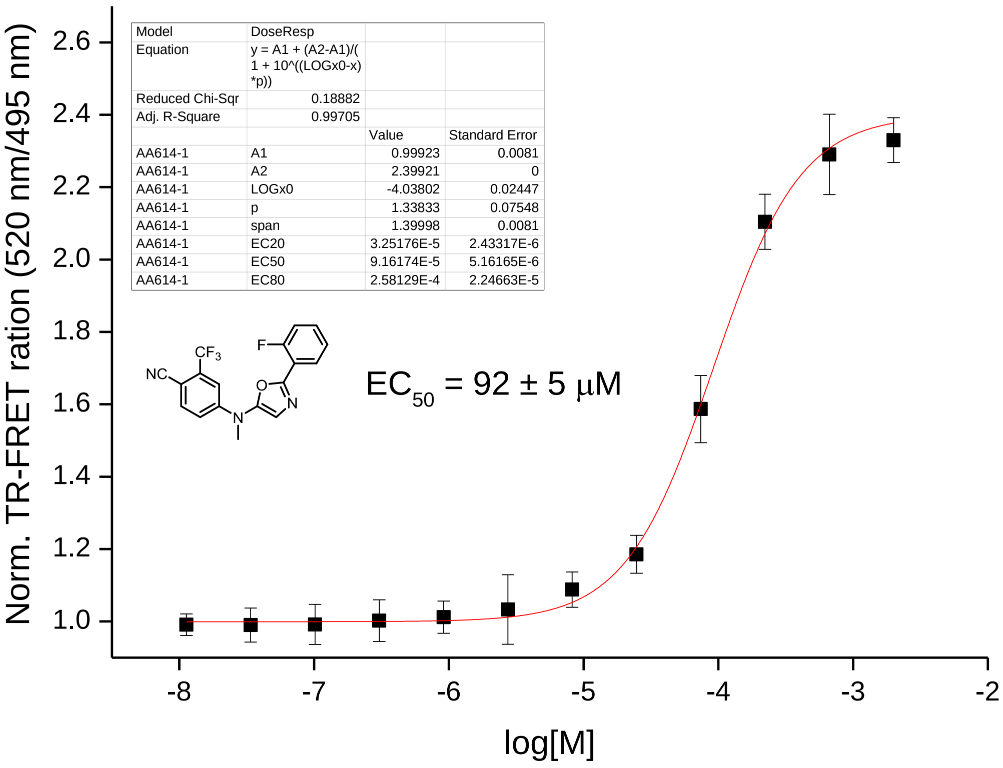

Oxazole **22**

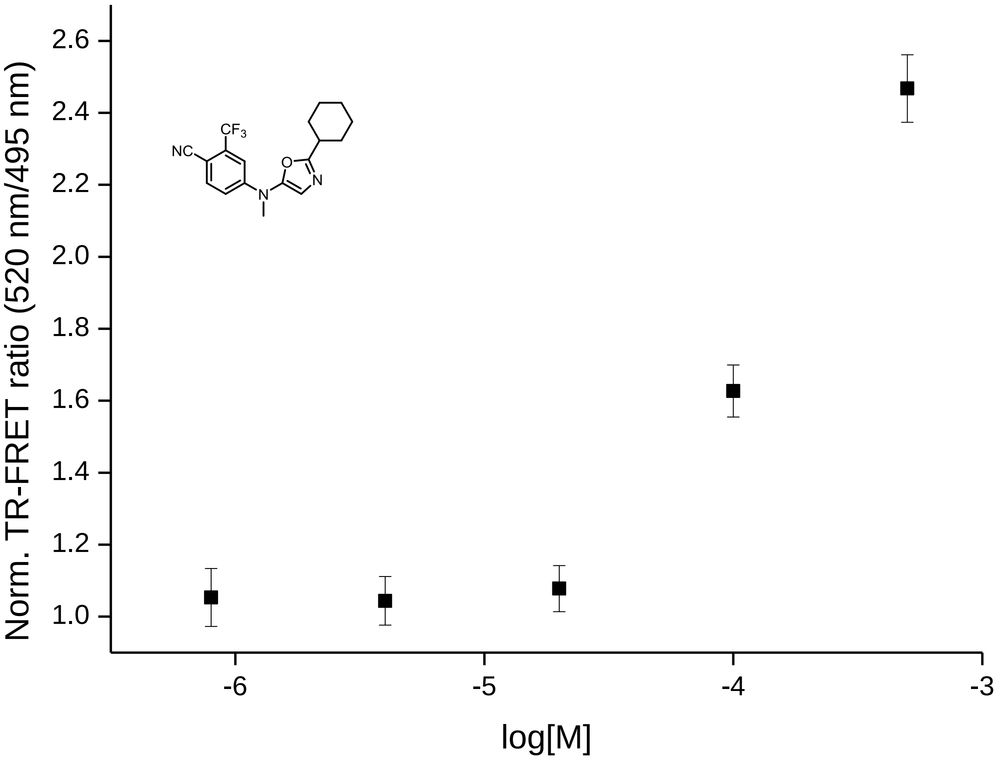

Oxazole **23**

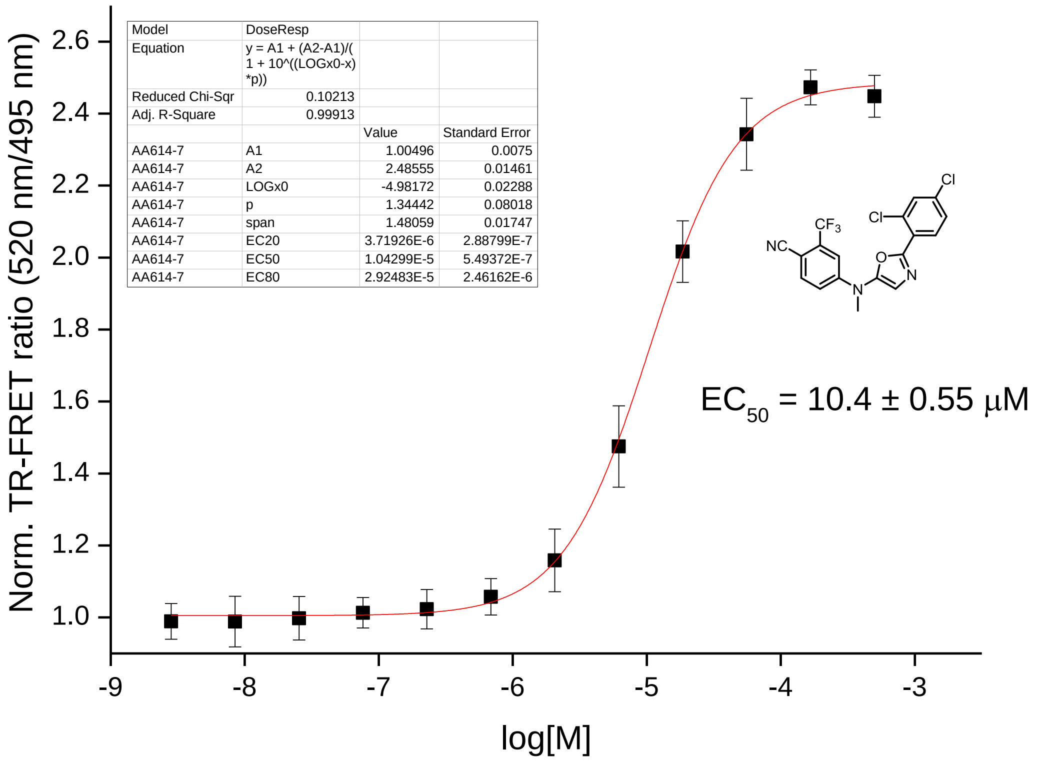

Model	DoseResp		
Equation	y = A1 + (A2-A1)/(1 + 10^((LOGx0-x) *p))		
Reduced Chi-Sqr		0.10213	
Adj. R-Square		0.99913	
		Value	Standard Error
AA614-7	A1	1.00496	0.0075
AA614-7	A2	2.48555	0.01461
AA614-7	LOGx0	-4.98172	0.02288
AA614-7	p	1.34442	0.08018
AA614-7	span	1.48059	0.01747
AA614-7	EC20	3.71926E-6	2.88799E-7
AA614-7	EC50	1.04299E-5	5.49372E-7
AA614-7	EC80	2.92483E-5	2.46162E-6

Oxazole **24**

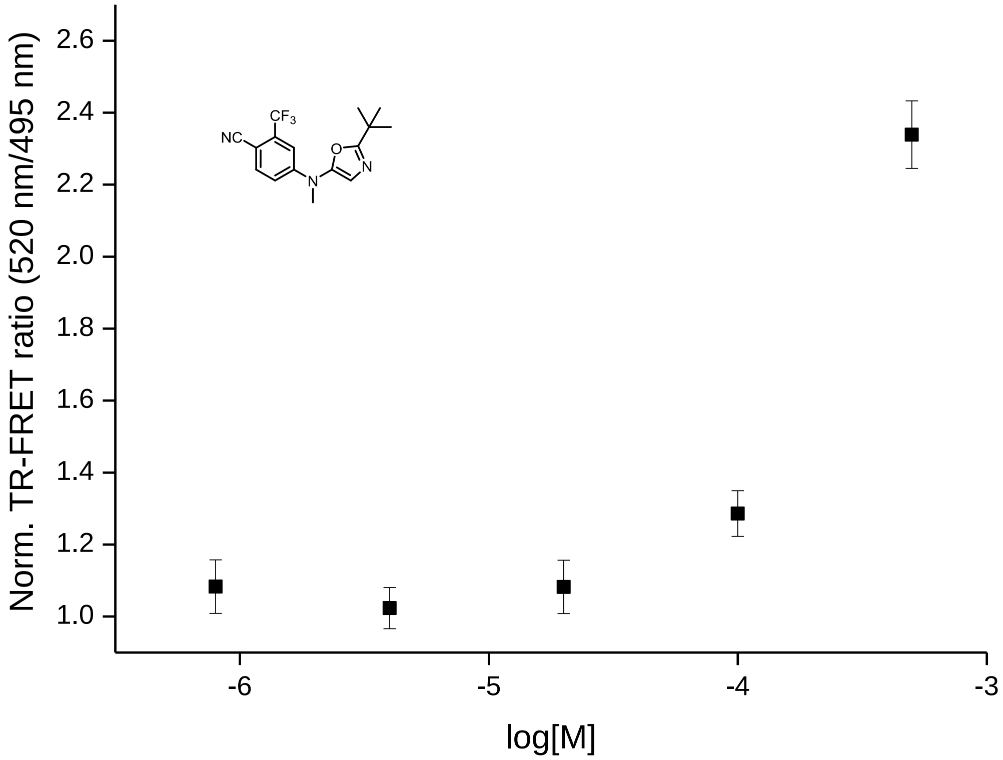

Oxazole **25**

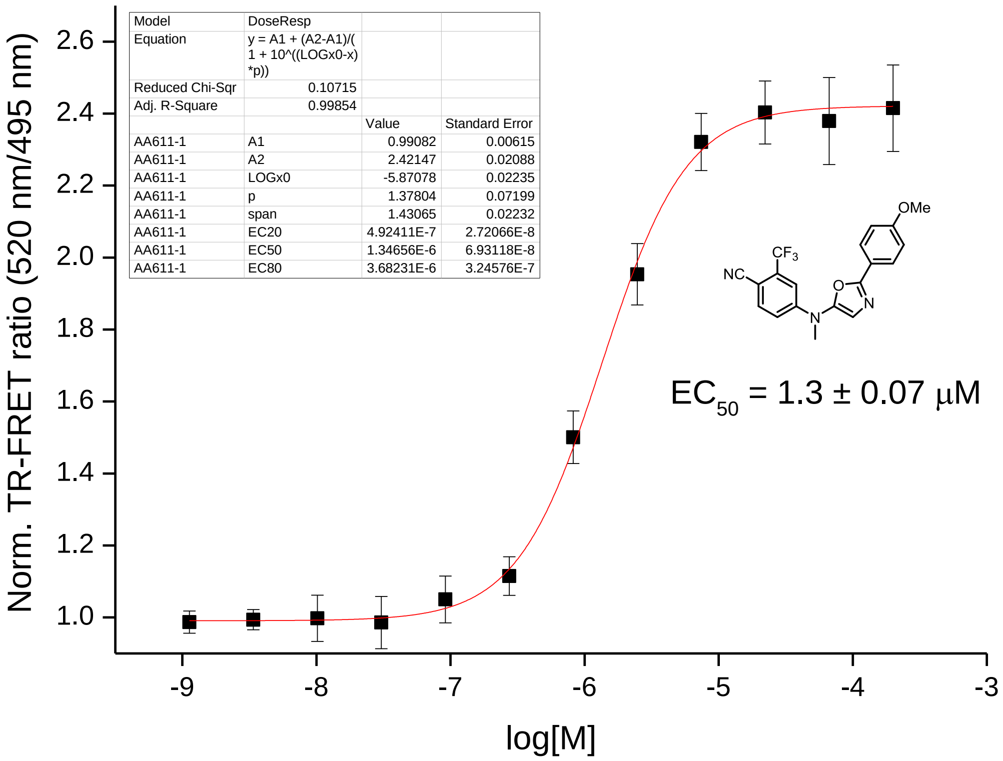

Model	DoseResp		
Equation	y = A1 + (A2-A1)/(1 + 10^((LOGx0-x) *p))		
Reduced Chi-Sqr	0.10715		
Adj. R-Square	0.99854		
		Value	Standard Error
AA611-1	A1	0.99082	0.00615
AA611-1	A2	2.42147	0.02088
AA611-1	LOGx0	-5.87078	0.02235
AA611-1	p	1.37804	0.07199
AA611-1	span	1.43065	0.02232
AA611-1	EC20	4.92411E-7	2.72066E-8
AA611-1	EC50	1.34656E-6	6.93118E-8
AA611-1	EC80	3.68231E-6	3.24576E-7

Oxazole **26**

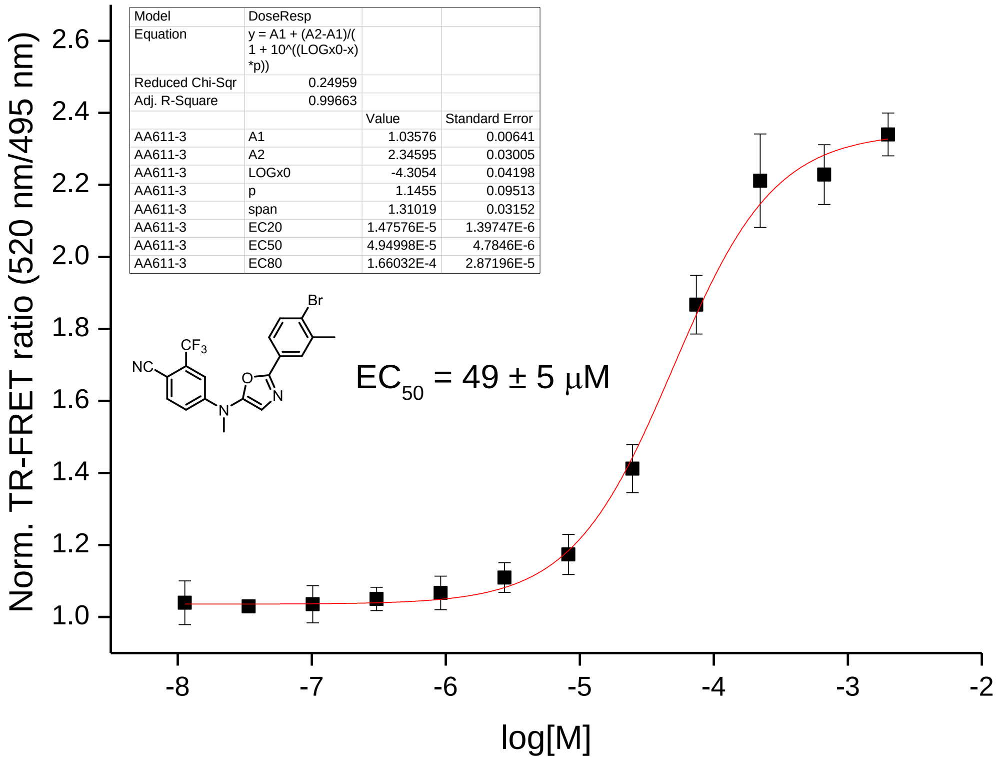

Model	DoseResp		
Equation	y = A1 + (A2-A1)/(1 + 10^((LOGx0-x) *p))		
Reduced Chi-Sqr	0.24959		
Adj. R-Square	0.99663		
		Value	Standard Error
AA611-3	A1	1.03576	0.00641
AA611-3	A2	2.34595	0.03005
AA611-3	LOGx0	-4.3054	0.04198
AA611-3	p	1.1455	0.09513
AA611-3	span	1.31019	0.03152
AA611-3	EC20	1.47576E-5	1.39747E-6
AA611-3	EC50	4.94998E-5	4.7846E-6
AA611-3	EC80	1.66032E-4	2.87196E-5

Ether **27**

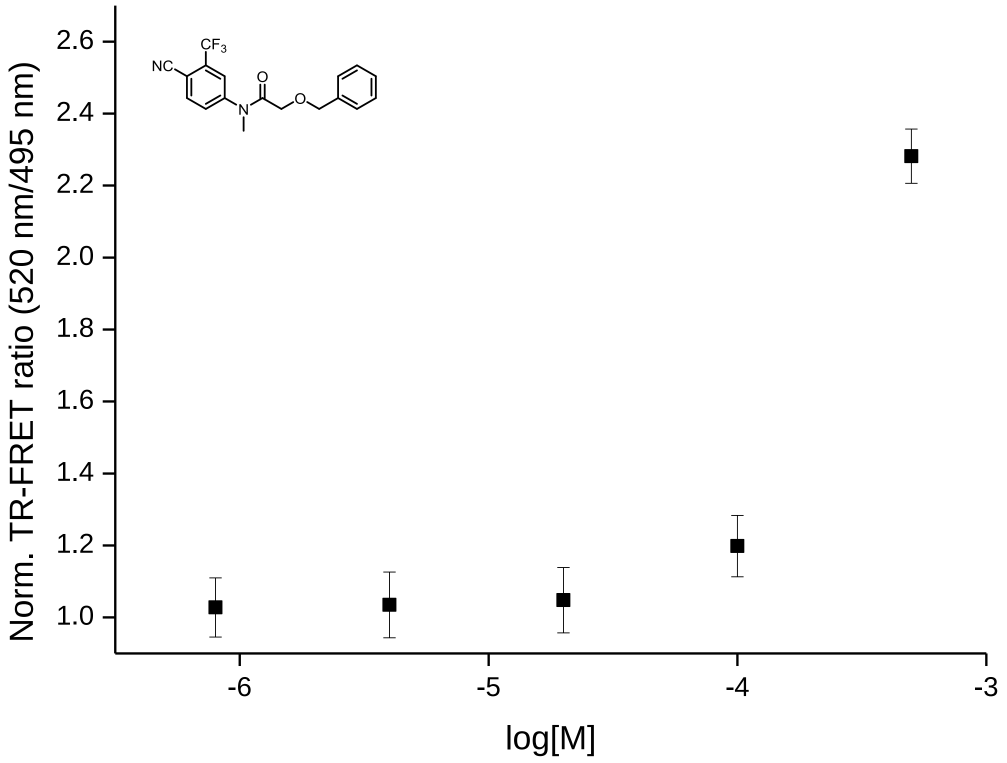

Ether **28**

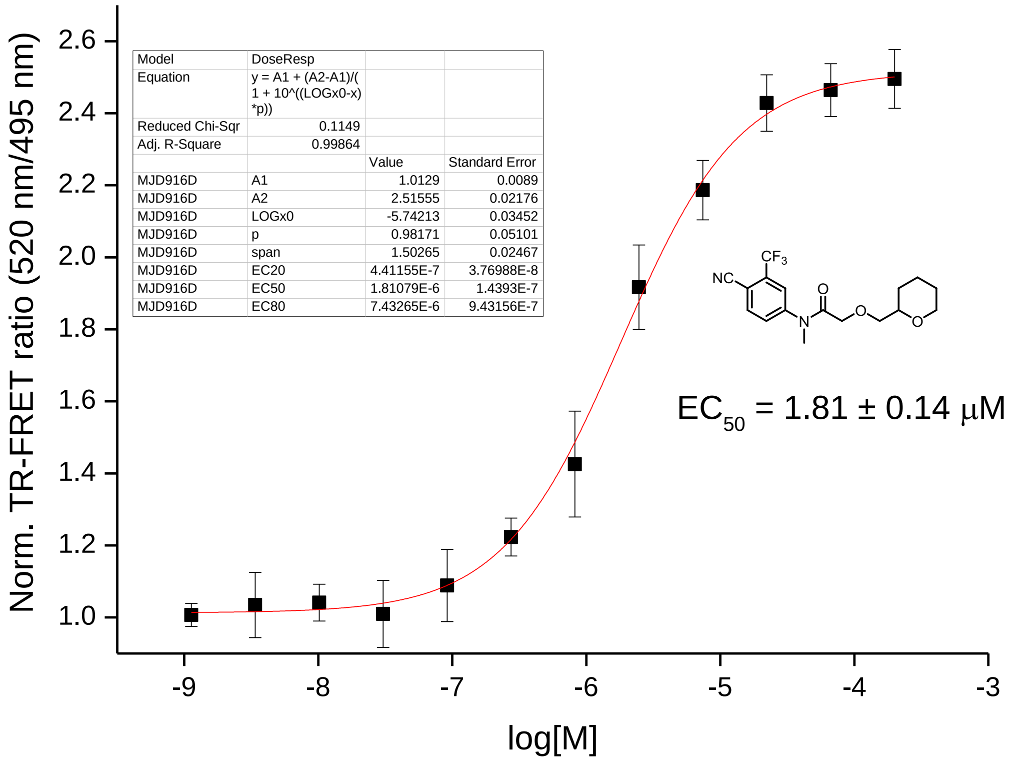

Ether **29**

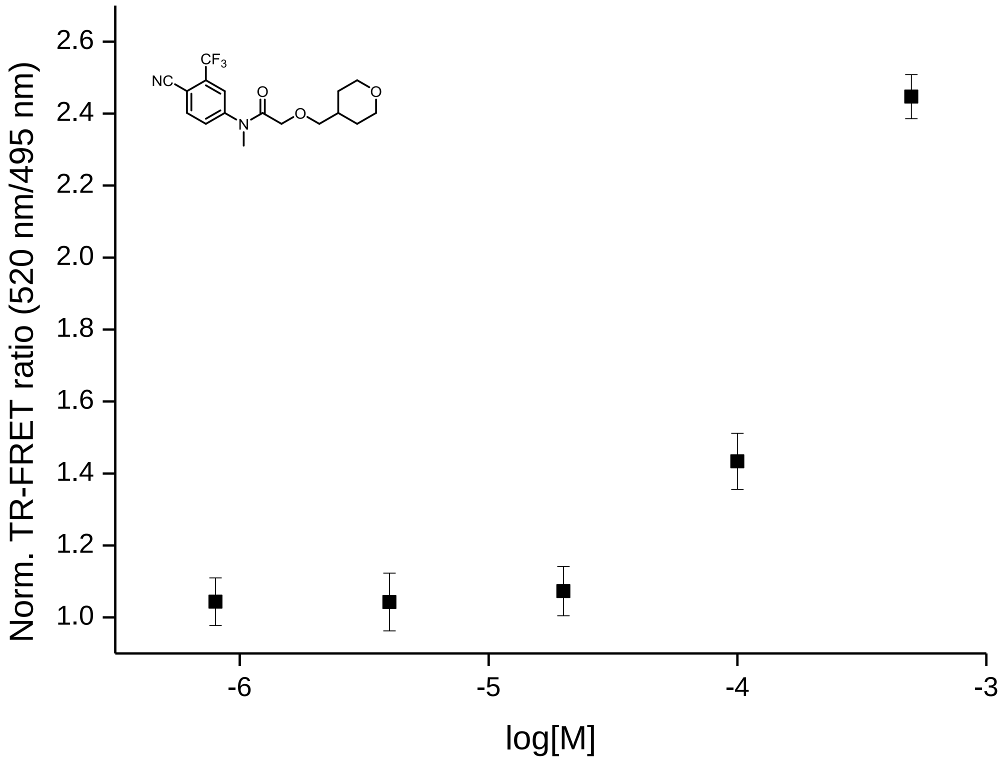

Ether **30**

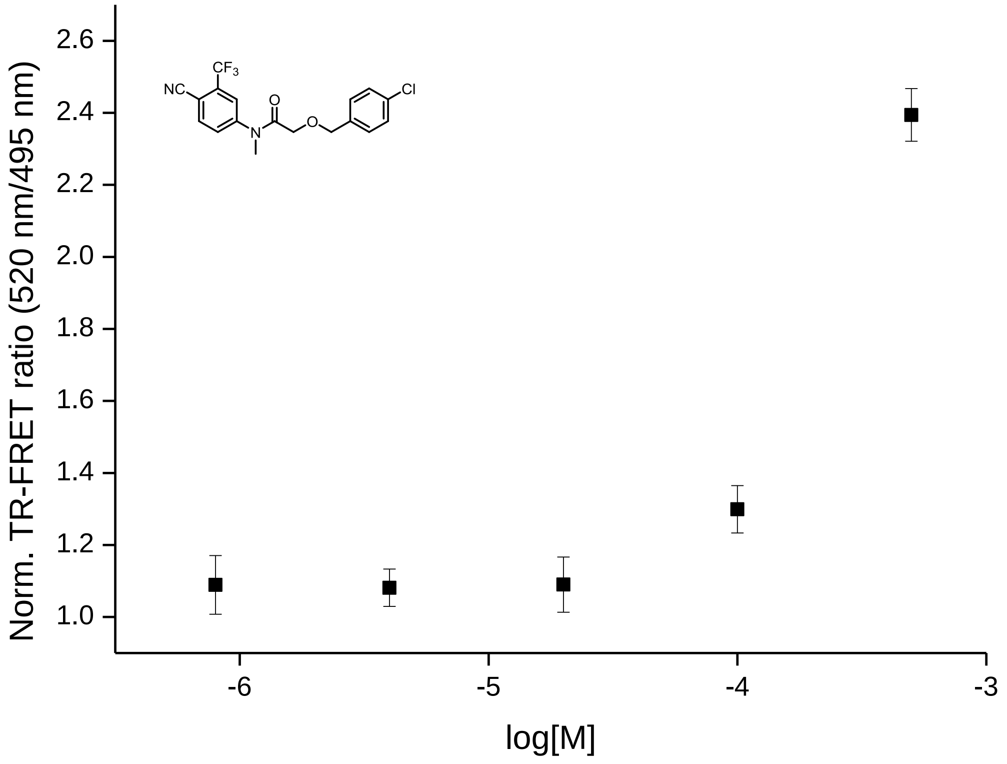

Ether **31**

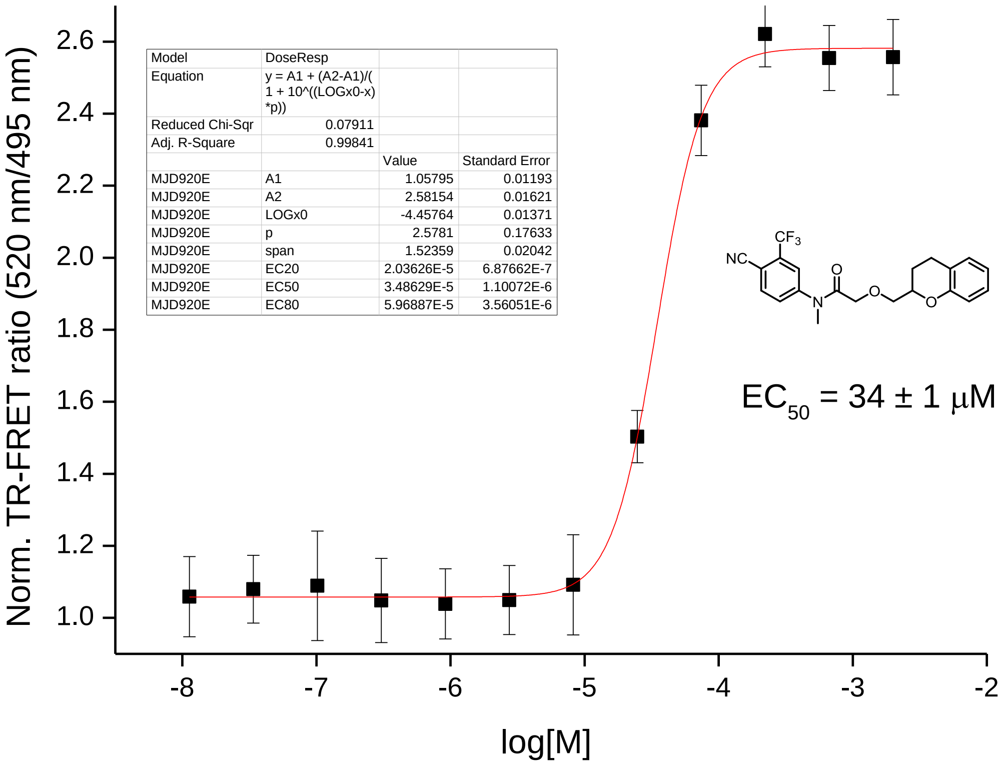

Ether **32**

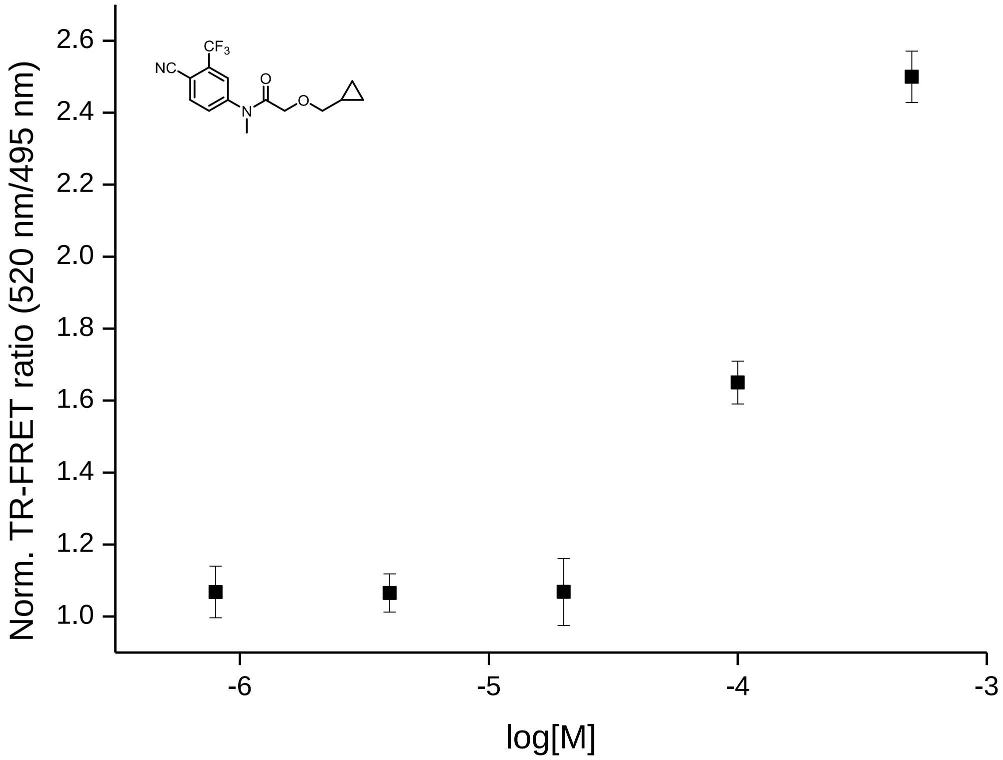

Ether **33**

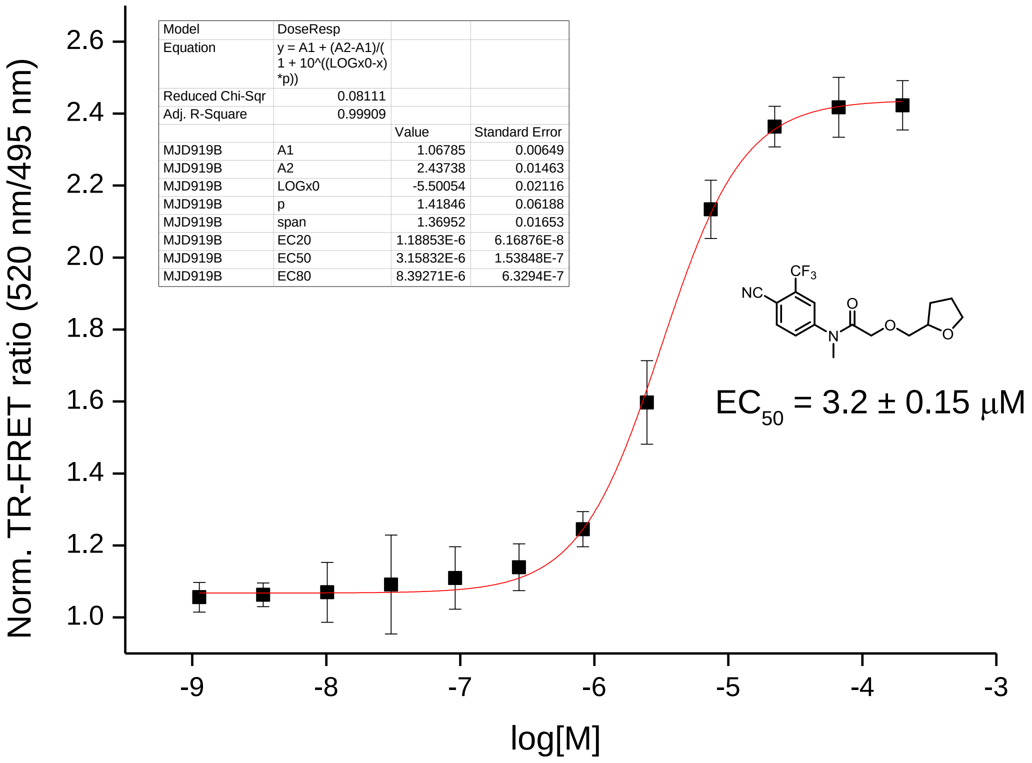

Ether **34**

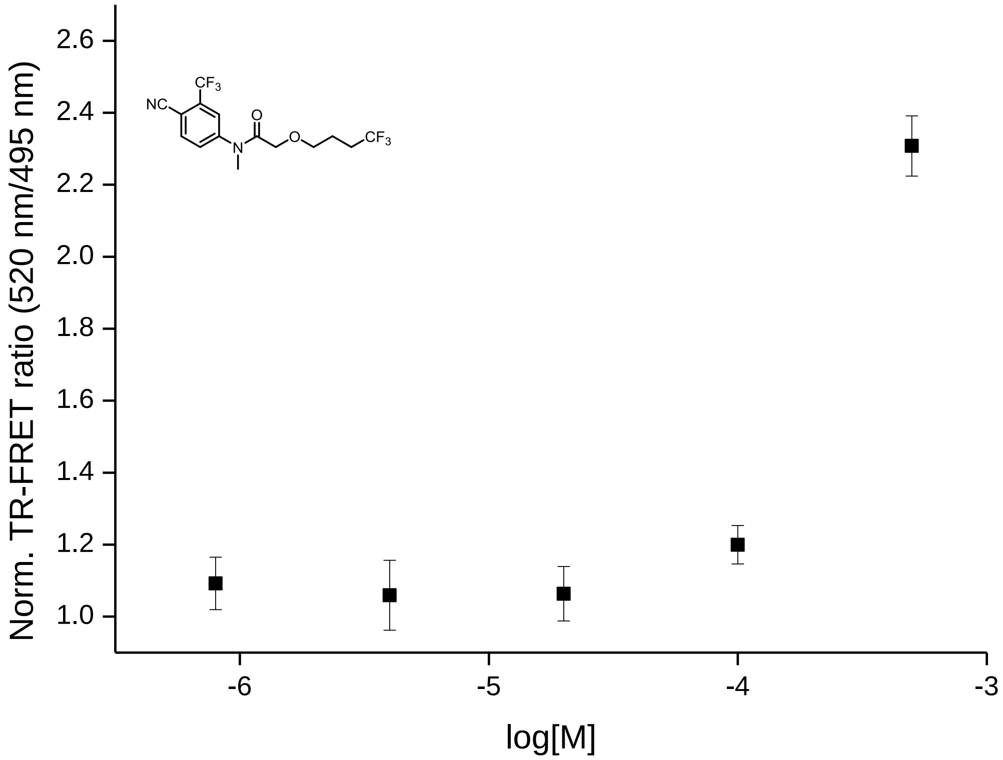

Ether **35**

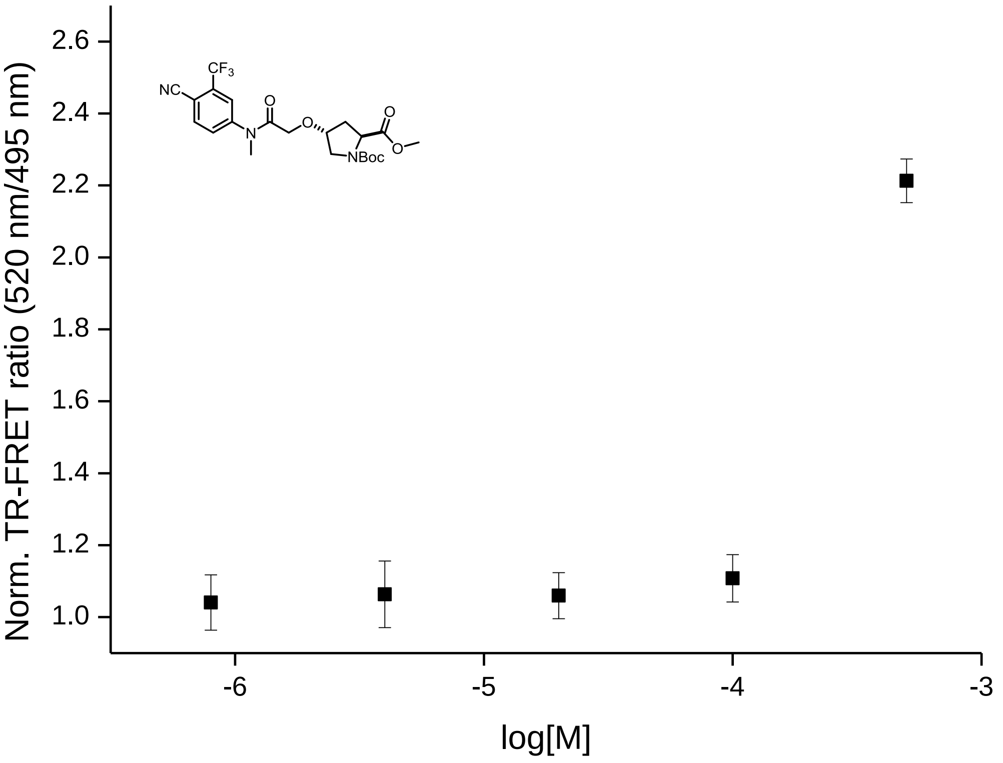

Ether **36**

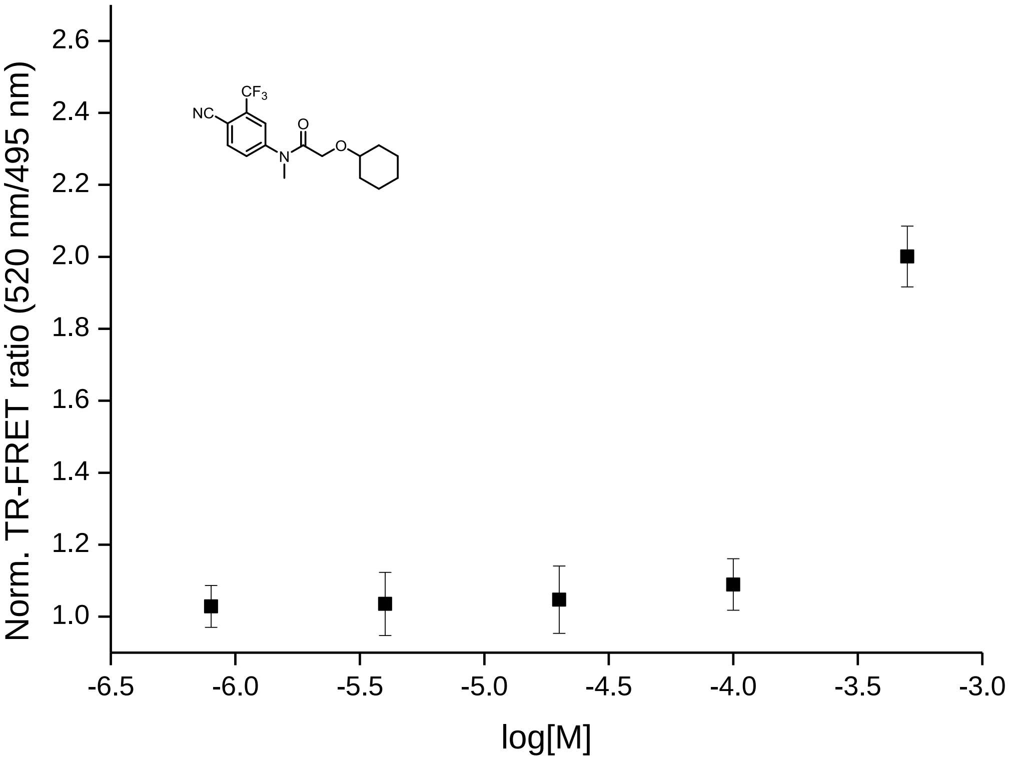

Ether **37**

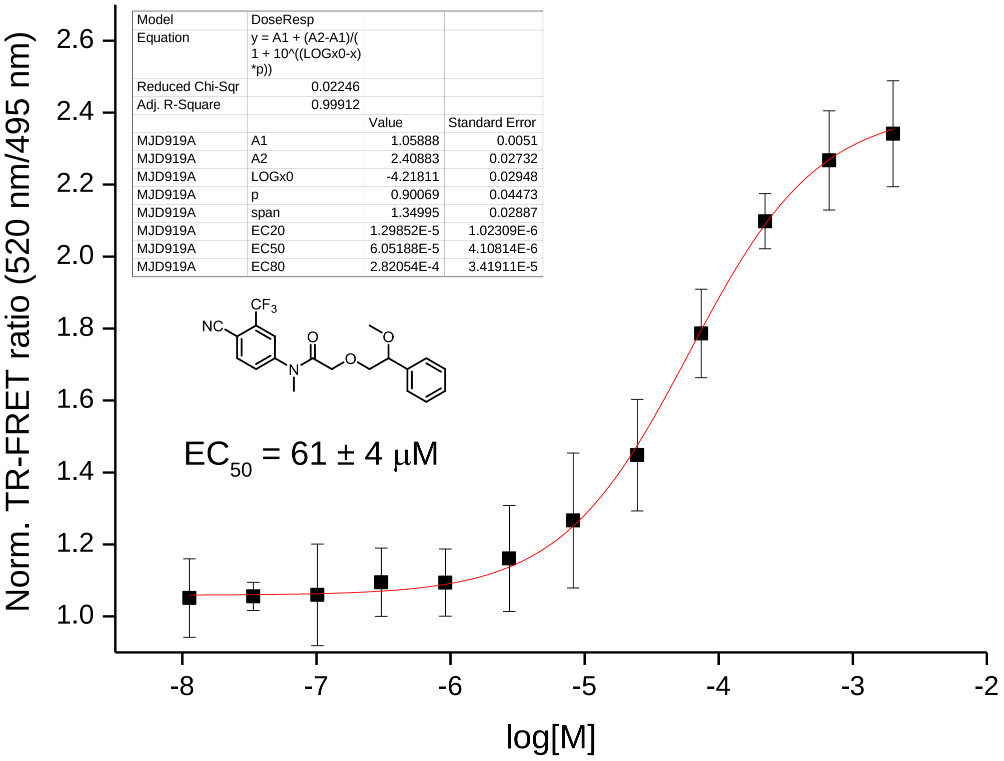
Model DoseResp
Equation y = A1 + (A2-A1)/(1 + 10^((LOGx0-x) *p))
Reduced Chi-Sqr 0.02246
Adj. R-Square 0.99912
Value Standard Error
MJD919A A1 1.05888 0.0051
MJD919A A2 2.40883 0.02732
MJD919A LOGx0 -4.21811 0.02948
MJD919A p 0.90069 0.04473
MJD919A span 1.34995 0.02887
MJD919A EC20 1.29852E-5 1.02309E-6
MJD919A EC50 6.05188E-5 4.10814E-6
MJD919A EC80 2.82054E-4 3.41911E-5
NC
CF3
O
N
O
O
EC50 = 61 ± 4 µM
Norm. TR-FRET ratio (520 nm/495 nm)
2.6
2.4
2.2
2.0
1.8
1.6
1.4
1.2
1.0
-8
-7
-6
-5
-4
-3
-2
log[M]

7 Reaction array Data:

Reaction mixtures from round 1 assayed at a total product concentration Σ[Pn] = 10 μM. Reaction mixtures from round 2 assayed at total product concentrations Σ[Pn] = 10 μM and Σ[Pn] = 5μM. Reaction mixtures from round 3 assayed at total product concentrations Σ[Pn] = 5μM and Σ[Pn] = 1μM. Biological activity is expressed relative to 5 μM testosterone. See Reaction Array Tablesfor the catalysts (represented by different colours) and solvents (represented by different shapes) used. Experiments were performed in duplicate (typical error 1-7%).

Reaction array 1:

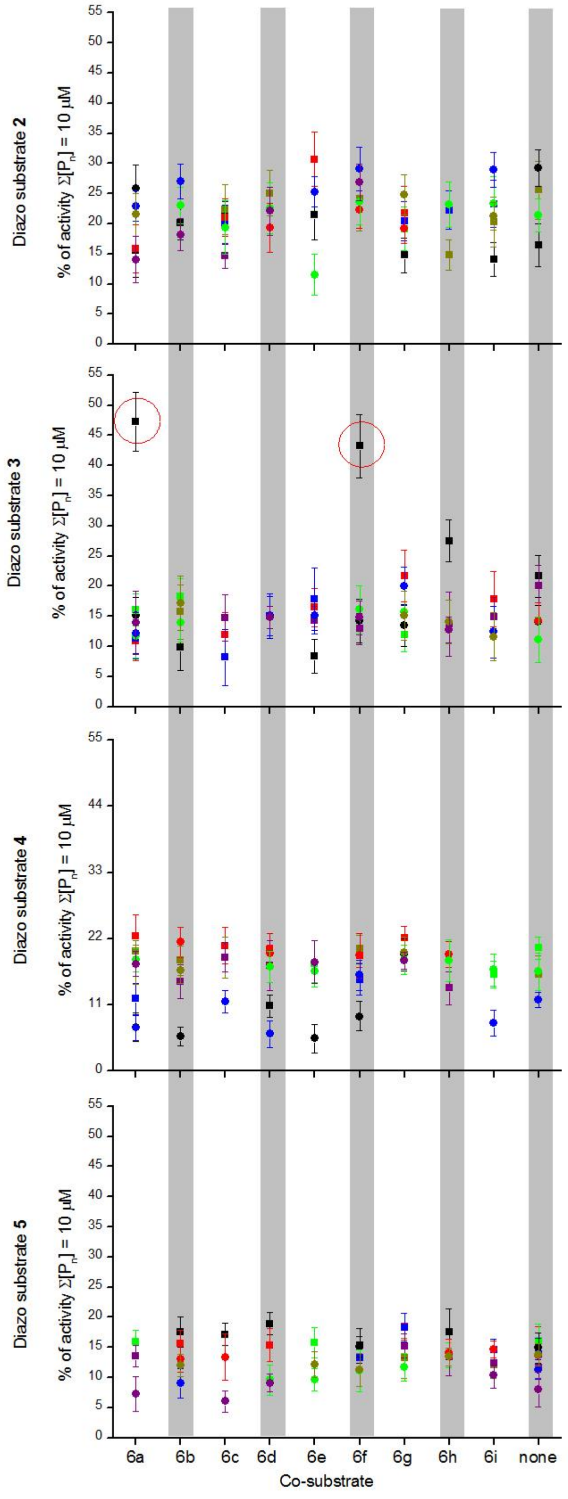

Reaction array 2:

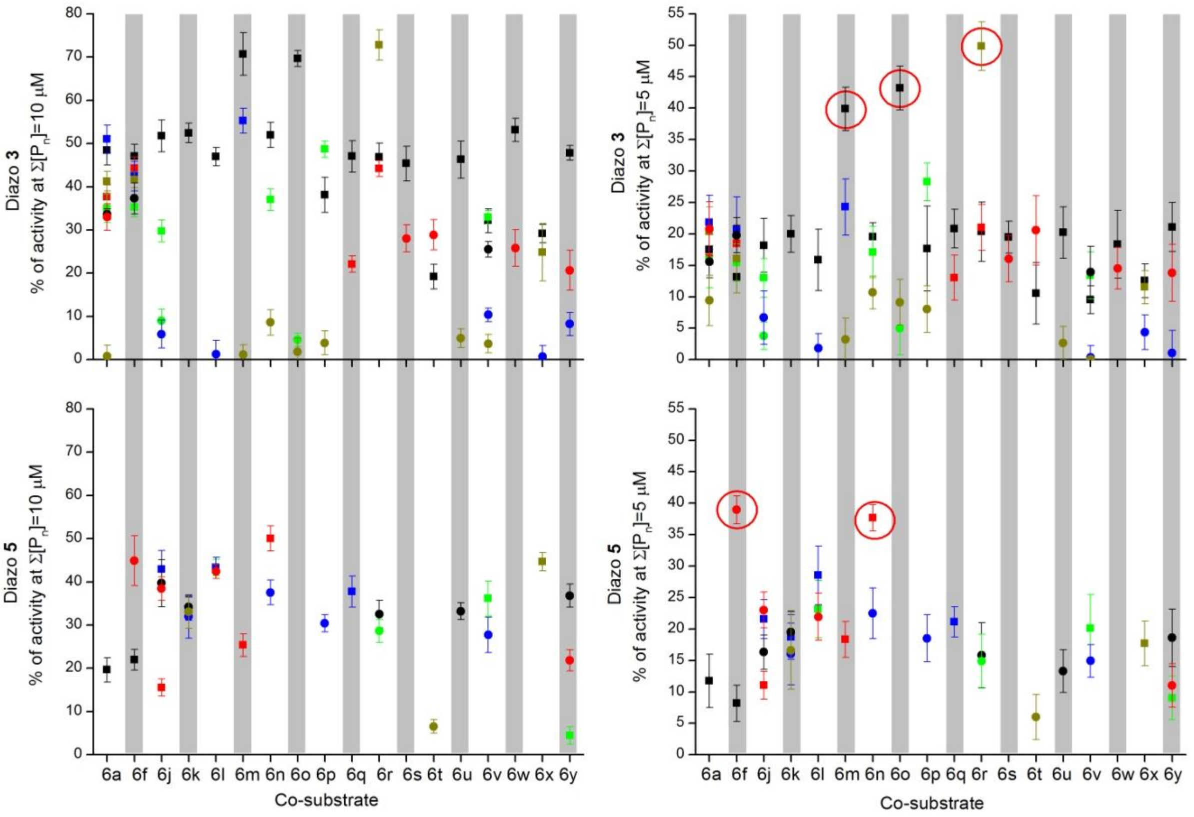

Reaction Array 3:

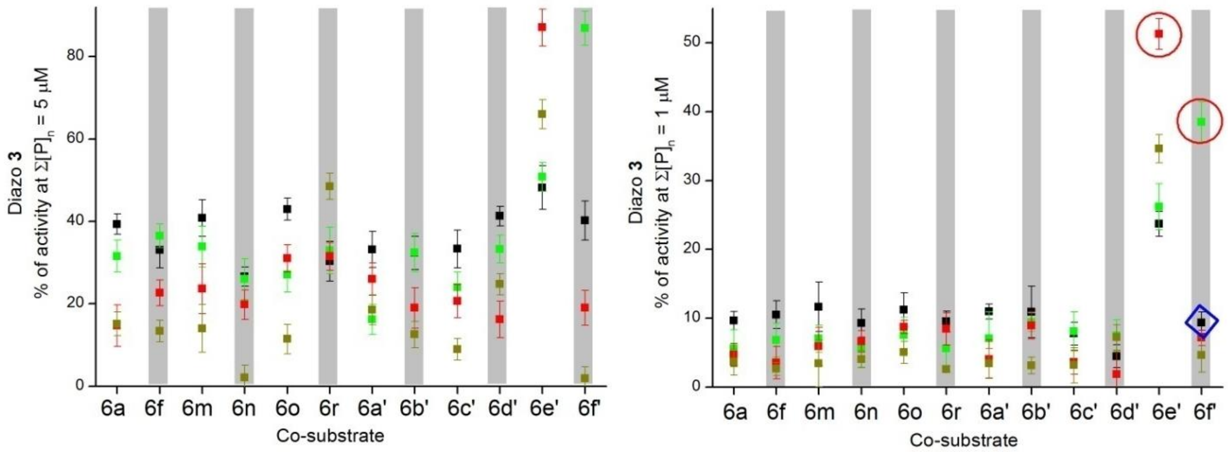

8 Activity of individual co-substrates:

To demonstrate that any unreacted co-substrate would not interfere with the assay, mock microreactions with potential co-substrates for each Activity-Directed Synthesis round were performed. Stock solutions of the co-substrates (8 µL of 12.5 M solution in CH_2Cl_2) were added to the wells of the custom made 96-well PTFE plates with the appropriate solvent (92 µL, either CH_2Cl_2 or toluene) to give a final concentration of 1.0 M of each co-substrate. The microreactions were mixed by pipetting sealed and left for 48 h. After scavenging for 24 h, the solutions were left to evaporate and the crude dissolved in dimethyl sulfoxide to give a final solution of 1.0 M of co-substrate. These stock solutions were subsequently further diluted in DMSO and buffer and screened at a concentration of 1 mM (1% DMSO in buffer) following General Procedure G.

Co-substrates for reaction array 1:

Entry	Co-substrate	Structure	POA at 1 mM[a]
1	6a	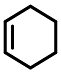	3 ± 0.5 %
2	6b	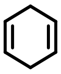	5 ± 1 %
3	6c	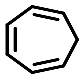	6 ± 1 %
4	6d	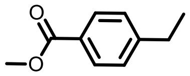	2 ± 0.5 %
5	6e	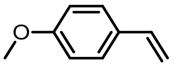	8 ± 2 %
6	6f	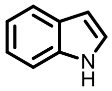	1 ± 1 %
7	6g	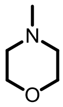	8 ± 1 %
8	6h	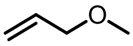	9 ± 2 %
9	6i	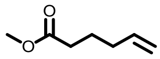	1 ± 0.5 %
10	-	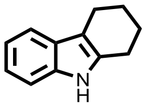	57 ± 5%
11	-	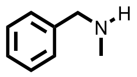	22 ± 3 %
12	-	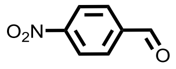	34 ± 5 %

a: POA = Percentage of Activity relative to 5 µM Testosterone solution.

Co-substrates for reaction array 2:

Entry	Co-substrate	Structure	POAat1 mM[a]	Entry	Co-substrate	Structure	POA at 1 mM[a]
**1**	**6j**	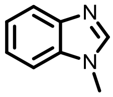	2 ± 1 %	**9**	**6r**	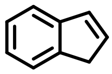	1 ± 1 %
**2**	**6k**	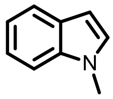	1 ± 1 %	**10**	**6s**	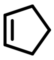	0 ± 1 %
**3**	**6l**	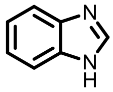	1 ± 1 %	**11**	**6t**	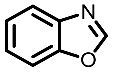	0 ± 1 %
**4**	**6m**	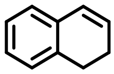	0 ± 1 %	**12**	**6u**	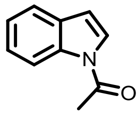	4 ± 2%
**5**	**6n**	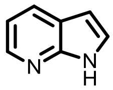	1 ± 1 %	**13**	**6v**	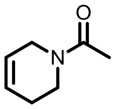	1 ± 1 %
**6**	**6o**	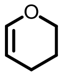	0 ± 1 %	**14**	**6w**	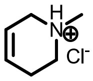	0 ± 1 %
**7**	**6p**	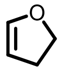	1 ± 1 %	**15**	**6x**	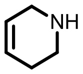	6 ± 2%
**8**	**6q**	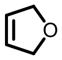	0 ± 1 %	**16**	**6y**	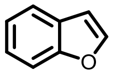	2 ± 1%

a: POA = Percentage of Activity relative to 5 μM Testosterone solution.

Co-substrates for reaction array 3:

Entry	Co-substrate	Structure	POA at 1 mM[a]
**1**	**6a'**	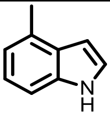	9 ± 1 %
**2**	**6b'**	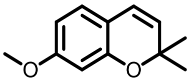	5 ± 1 %
**3**	**6c'**	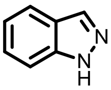	5 ± 1 %
**4**	**6d'**	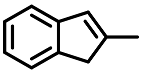	7 ± 1 %
**5**	**6e'**	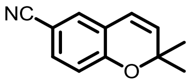	12 ± 2 %
**6**	**6f'**	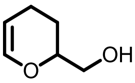	3 ± 2 %

a: POA = Percentage of Activity relative to 5 μM Testosterone solution.

9 Reaction array tables

Reaction array 1 data: Activities of product mixtures prepared in round one. Product mixtures were assayed at a total product concentration of 10 μMfollowing General Procedure G.

Diazo-substrate	Co substrate	Catalyst	Solvent	POA at [10 μM][a]
**2**	**6a**	Rh_2(S-DOSP)$_4$	CH_2Cl_2	15.8 ± 4
**2**	**6b**	Rh_2(S-DOSP)$_4$	CH_2Cl_2	20.1 ± 3
**2**	**6c**	Rh_2(S-DOSP)$_4$	CH_2Cl_2	21.5 ± 2
**2**	**6e**	Rh_2(S-DOSP)$_4$	CH_2Cl_2	21.4 ± 4
**2**	**6g**	Rh_2(S-DOSP)$_4$	CH_2Cl_2	14.8 ± 3
**2**	**6i**	Rh_2(S-DOSP)$_4$	CH_2Cl_2	14 ± 3
**2**	None	Rh_2(S-DOSP)$_4$	CH_2Cl_2	16.5 ± 4
**2**	**6a**	Rh_2(TFA)$_4$	CH_2Cl_2	15.9 ± 4
**2**	**6c**	Rh_2(TFA)$_4$	CH_2Cl_2	21.1 ± 3
**2**	**6e**	Rh_2(TFA)$_4$	CH_2Cl_2	30.7 ± 5
**2**	**6g**	Rh_2(TFA)$_4$	CH_2Cl_2	21.7 ± 4
**2**	**6g**	Rh_2(5R-MEPY)$_4$	CH_2Cl_2	19.2 ± 4
**2**	**6c**	Rh_2(Oct)$_4$	CH_2Cl_2	19.9 ± 3
**2**	**6g**	Rh_2(Oct)$_4$	CH_2Cl_2	20.4 ± 3
**2**	**6h**	Rh_2(Oct)$_4$	CH_2Cl_2	22.3 ± 3
**2**	**6i**	Rh_2(Oct)$_4$	CH_2Cl_2	23.3 ± 4
**2**	**6c**	Rh_2(4S-MEOX)$_4$	CH_2Cl_2	22.4 ± 4
**2**	**6d**	Rh_2(4S-MEOX)$_4$	CH_2Cl_2	25 ± 4
**2**	**6f**	Rh_2(4S-MEOX)$_4$	CH_2Cl_2	24.2 ± 5
**2**	**6h**	Rh_2(4S-MEOX)$_4$	CH_2Cl_2	14.9 ± 3
**2**	**6i**	Rh_2(4S-MEOX)$_4$	CH_2Cl_2	20.3 ± 4
**2**	None	Rh_2(4S-MEOX)$_4$	CH_2Cl_2	25.6 ± 5
**2**	**6c**	Rh_2(cap)$_4$	CH_2Cl_2	14.7 ± 2
**2**	**6a**	Rh_2(S-DOSP)$_4$	PhMe	25.8 ± 4
**2**	None	Rh_2(S-DOSP)$_4$	PhMe	29.3 ± 3
**2**	**6d**	Rh_2(TFA)$_4$	PhMe	19.3 ± 4
**2**	**6f**	Rh_2(TFA)$_4$	PhMe	22.3 ± 3
**2**	**6g**	Rh_2(TFA)$_4$	PhMe	19.2 ± 3
**2**	**6b**	Rh_2(5R-MEPY)$_4$	PhMe	23 ± 3
**2**	**6c**	Rh_2(5R-MEPY)$_4$	PhMe	19.4 ± 4
**2**	**6d**	Rh_2(5R-MEPY)$_4$	PhMe	22.6 ± 4
**2**	**6e**	Rh_2(5R-MEPY)$_4$	PhMe	11.6 ± 3
**2**	**6f**	Rh_2(5R-MEPY)$_4$	PhMe	23.6 ± 4
**2**	**6h**	Rh_2(5R-MEPY)$_4$	PhMe	23.2 ± 4
**2**	**6i**	Rh_2(5R-MEPY)$_4$	PhMe	23.4 ± 4
**2**	None	Rh_2(5R-MEPY)$_4$	PhMe	21.4 ± 3
**2**	**6a**	Rh_2(Oct)$_4$	PhMe	22.9 ± 2
**2**	**6b**	Rh_2(Oct)$_4$	PhMe	27 ± 3
**2**	**6e**	Rh_2(Oct)$_4$	PhMe	25.3 ± 3
**2**	**6f**	Rh_2(Oct)$_4$	PhMe	29.1 ± 4
**2**	**6i**	Rh_2(Oct)$_4$	PhMe	29 ± 3
**2**	**6a**	Rh_2(4S-MEOX)$_4$	PhMe	21.5 ± 4

**2**	**6g**	Rh_2(4S-MEOX)$_4$	PhMe	24.8 ± 3
**2**	**6i**	Rh_2(4S-MEOX)$_4$	PhMe	21.2 ± 2
**2**	**6a**	Rh_2(cap)$_4$	PhMe	14 ± 4
**2**	**6b**	Rh_2(cap)$_4$	PhMe	18.2 ± 3
**2**	**6d**	Rh_2(cap)$_4$	PhMe	22.1 ± 4
**2**	**6f**	Rh_2(cap)$_4$	PhMe	26.8 ± 3
**3**	**6a**	Rh_2(S-DOSP)$_4$	CH_2Cl_2	47.3 ± 5
**3**	**6b**	Rh_2(S-DOSP)$_4$	CH_2Cl_2	9.9 ± 4
**3**	**6e**	Rh_2(S-DOSP)$_4$	CH_2Cl_2	8.4 ± 3
**3**	**6f**	Rh_2(S-DOSP)$_4$	CH_2Cl_2	43.2 ± 5
**3**	**6h**	Rh_2(S-DOSP)$_4$	CH_2Cl_2	27.5 ± 3
**3**	None	Rh_2(S-DOSP)$_4$	CH_2Cl_2	21.7 ± 3
**3**	**6a**	Rh_2(TFA)$_4$	CH_2Cl_2	10.9 ± 3
**3**	**6c**	Rh_2(TFA)$_4$	CH_2Cl_2	12 ± 4
**3**	**6e**	Rh_2(TFA)$_4$	CH_2Cl_2	16.5 ± 3
**3**	**6g**	Rh_2(TFA)$_4$	CH_2Cl_2	21.7 ± 4
**3**	**6i**	Rh_2(TFA)$_4$	CH_2Cl_2	17.9 ± 5
**3**	**6a**	Rh_2(5R-MEPY)$_4$	CH_2Cl_2	16 ± 3
**3**	**6b**	Rh_2(5R-MEPY)$_4$	CH_2Cl_2	18.4 ± 3
**3**	**6g**	Rh_2(5R-MEPY)$_4$	CH_2Cl_2	12 ± 3
**3**	**6a**	Rh_2(Oct)$_4$	CH_2Cl_2	11.3 ± 3
**3**	**6c**	Rh_2(Oct)$_4$	CH_2Cl_2	8.2 ± 5
**3**	**6d**	Rh_2(Oct)$_4$	CH_2Cl_2	15 ± 3
**3**	**6e**	Rh_2(Oct)$_4$	CH_2Cl_2	17.8 ± 5
**3**	**6b**	Rh_2(4S-MEOX)$_4$	CH_2Cl_2	15.7 ± 5
**3**	**6c**	Rh_2(cap)$_4$	CH_2Cl_2	14.8 ± 4
**3**	**6e**	Rh_2(cap)$_4$	CH_2Cl_2	14.4 ± 2
**3**	**6f**	Rh_2(cap)$_4$	CH_2Cl_2	12.9 ± 3
**3**	**6h**	Rh_2(cap)$_4$	CH_2Cl_2	13.7 ± 5
**3**	**6i**	Rh_2(cap)$_4$	CH_2Cl_2	15 ± 3
**3**	None	Rh_2(cap)$_4$	CH_2Cl_2	20.1 ± 3
**3**	**6a**	Rh_2(S-DOSP)$_4$	PhMe	15.1 ± 3
**3**	**6f**	Rh_2(S-DOSP)$_4$	PhMe	14.3 ± 4
**3**	**6g**	Rh_2(S-DOSP)$_4$	PhMe	13.5 ± 4
**3**	None	Rh_2(S-DOSP)$_4$	PhMe	14.1 ± 3
**3**	None	Rh_2(TFA)$_4$	PhMe	14.2 ± 3
**3**	**6a**	Rh_2(5R-MEPY)$_4$	PhMe	11.8 ± 4
**3**	**6b**	Rh_2(5R-MEPY)$_4$	PhMe	13.9 ± 3
**3**	**6f**	Rh_2(5R-MEPY)$_4$	PhMe	16.2 ± 4
**3**	**6g**	Rh_2(5R-MEPY)$_4$	PhMe	15.6 ± 4
**3**	None	Rh_2(5R-MEPY)$_4$	PhMe	11.1 ± 4
**3**	**6a**	Rh_2(Oct)$_4$	PhMe	12.2 ± 3
**3**	**6d**	Rh_2(Oct)$_4$	PhMe	15.1 ± 4
**3**	**6e**	Rh_2(Oct)$_4$	PhMe	15.1 ± 3
**3**	**6g**	Rh_2(Oct)$_4$	PhMe	20 ± 3
**3**	**6i**	Rh_2(Oct)$_4$	PhMe	12.4 ± 4
**3**	**6b**	Rh_2(4S-MEOX)$_4$	PhMe	17.2 ± 4
**3**	**6g**	Rh_2(4S-MEOX)$_4$	PhMe	15.1 ± 4
**3**	**6h**	Rh_2(4S-MEOX)$_4$	PhMe	14.1 ± 4
**3**	**6i**	Rh_2(4S-MEOX)$_4$	PhMe	11.6 ± 4

**3**	**6a**	$Rh_2(cap)_4$	PhMe	13.9 ± 5
**3**	**6d**	$Rh_2(cap)_4$	PhMe	14.8 ± 2
**3**	**6f**	$Rh_2(cap)_4$	PhMe	14.8 ± 3
**3**	**6h**	$Rh_2(cap)_4$	PhMe	12.7 ± 2
**4**	**6d**	Rh_2(S-DOSP)$_4$	CH_2Cl_2	10.8 ± 2
**4**	**6a**	$Rh_2(TFA)_4$	CH_2Cl_2	22.4 ± 4
**4**	**6c**	$Rh_2(TFA)_4$	CH_2Cl_2	20.8 ± 3
**4**	**6d**	$Rh_2(TFA)_4$	CH_2Cl_2	20.2 ± 3
**4**	**6f**	$Rh_2(TFA)_4$	CH_2Cl_2	19.1 ± 2
**4**	**6g**	$Rh_2(TFA)_4$	CH_2Cl_2	22.1 ± 2
**4**	**6e**	Rh_2(5R-MEPY)$_4$	CH_2Cl_2	17.9 ± 4
**4**	**6i**	Rh_2(5R-MEPY)$_4$	CH_2Cl_2	16 ± 2
**4**	None	Rh_2(5R-MEPY)$_4$	CH_2Cl_2	20.4 ± 2
**4**	**6a**	$Rh_2(Oct)_4$	CH_2Cl_2	12 ± 3
**4**	**6f**	$Rh_2(Oct)_4$	CH_2Cl_2	15.2 ± 3
**4**	**6a**	Rh_2(4S-MEOX)$_4$	CH_2Cl_2	19.9 ± 2
**4**	**6b**	Rh_2(4S-MEOX)$_4$	CH_2Cl_2	18.4 ± 2
**4**	**6c**	Rh_2(4S-MEOX)$_4$	CH_2Cl_2	18.9 ± 3
**4**	**6f**	Rh_2(4S-MEOX)$_4$	CH_2Cl_2	20.3 ± 2
**4**	None	Rh_2(4S-MEOX)$_4$	CH_2Cl_2	16.1 ± 3
**4**	**6b**	$Rh_2(cap)_4$	CH_2Cl_2	14.9 ± 3
**4**	**6c**	$Rh_2(cap)_4$	CH_2Cl_2	18.9 ± 2
**4**	**6d**	$Rh_2(cap)_4$	CH_2Cl_2	17.6 ± 4
**4**	**6h**	$Rh_2(cap)_4$	CH_2Cl_2	13.8 ± 3
**4**	**6a**	Rh_2(S-DOSP)$_4$	PhMe	7.3 ± 2
**4**	**6b**	Rh_2(S-DOSP)$_4$	PhMe	5.7 ± 2
**4**	**6e**	Rh_2(S-DOSP)$_4$	PhMe	5.4 ± 2
**4**	**6f**	Rh_2(S-DOSP)$_4$	PhMe	9.1 ± 2
**4**	**6g**	Rh_2(S-DOSP)$_4$	PhMe	19.3 ± 3
**4**	**6h**	$Rh_2(TFA)_4$	PhMe	19.4 ± 2
**4**	**6b**	$Rh_2(TFA)_4$	PhMe	21.4 ± 3
**4**	**6f**	$Rh_2(TFA)_4$	PhMe	19.2 ± 4
**4**	**6d**	$Rh_2(TFA)_4$	PhMe	19.5 ± 2
**4**	**6a**	Rh_2(5R-MEPY)$_4$	PhMe	18.5 ± 2
**4**	**6d**	Rh_2(5R-MEPY)$_4$	PhMe	17.4 ± 3
**4**	**6e**	Rh_2(5R-MEPY)$_4$	PhMe	16.6 ± 2
**4**	**6g**	Rh_2(5R-MEPY)$_4$	PhMe	18.5 ± 2
**4**	**6h**	Rh_2(5R-MEPY)$_4$	PhMe	18.4 ± 3
**4**	**6i**	Rh_2(5R-MEPY)$_4$	PhMe	16.9 ± 3
**4**	None	Rh_2(5R-MEPY)$_4$	PhMe	16.5 ± 3
**4**	**6c**	$Rh_2(Oct)_4$	PhMe	7.2 ± 2
**4**	**6a**	$Rh_2(Oct)_4$	PhMe	11.5 ± 2
**4**	**6d**	$Rh_2(Oct)_4$	PhMe	6.1 ± 2
**4**	**6f**	$Rh_2(Oct)_4$	PhMe	15.9 ± 3
**4**	**6i**	$Rh_2(Oct)_4$	PhMe	8 ± 2
**4**	None	$Rh_2(Oct)_4$	PhMe	11.9 ± 1
**4**	**6a**	Rh_2(4S-MEOX)$_4$	PhMe	17.7 ± 3
**4**	**6b**	Rh_2(4S-MEOX)$_4$	PhMe	16.8 ± 2
**4**	**6g**	Rh_2(4S-MEOX)$_4$	PhMe	19.7 ± 3
**4**	**6a**	$Rh_2(cap)_4$	PhMe	17.8 ± 2

**4**	**6e**	$Rh_2(cap)_4$	PhMe	18.1 ± 4
**4**	**6g**	$Rh_2(cap)_4$	PhMe	18.4 ± 1
**5**	**6b**	$Rh_2(S\text{-}DOSP)_4$	CH_2Cl_2	17.5 ± 2
**5**	**6c**	$Rh_2(S\text{-}DOSP)_4$	CH_2Cl_2	17.2 ± 2
**5**	**6d**	$Rh_2(S\text{-}DOSP)_4$	CH_2Cl_2	18.9 ± 2
**5**	**6f**	$Rh_2(S\text{-}DOSP)_4$	CH_2Cl_2	15.3 ± 3
**5**	**6h**	$Rh_2(S\text{-}DOSP)_4$	CH_2Cl_2	17.6 ± 4
**5**	**6b**	$Rh_2(TFA)_4$	CH_2Cl_2	15.7 ± 2
**5**	**6d**	$Rh_2(TFA)_4$	CH_2Cl_2	15.4 ± 3
**5**	**6f**	$Rh_2(TFA)_4$	CH_2Cl_2	13.2 ± 2
**5**	**6g**	$Rh_2(TFA)_4$	CH_2Cl_2	18.5 ± 2
**5**	None	$Rh_2(TFA)_4$	CH_2Cl_2	16 ± 2
**5**	**6a**	$Rh_2(5R\text{-}MEPY)_4$	CH_2Cl_2	15.9 ± 2
**5**	**6e**	$Rh_2(5R\text{-}MEPY)_4$	CH_2Cl_2	15.7 ± 3
**5**	**6h**	$Rh_2(5R\text{-}MEPY)_4$	CH_2Cl_2	13.7 ± 2
**5**	None	$Rh_2(5R\text{-}MEPY)_4$	CH_2Cl_2	16.1 ± 3
**5**	**6f**	$Rh_2(Oct)_4$	CH_2Cl_2	13.2 ± 2
**5**	**6g**	$Rh_2(Oct)_4$	CH_2Cl_2	18.3 ± 2
**5**	**6i**	$Rh_2(Oct)_4$	CH_2Cl_2	14.7 ± 2
**5**	None	$Rh_2(Oct)_4$	CH_2Cl_2	13.9 ± 3
**5**	**6g**	$Rh_2(4S\text{-}MEOX)_4$	CH_2Cl_2	13.2 ± 3
**5**	**6i**	$Rh_2(4S\text{-}MEOX)_4$	CH_2Cl_2	12.1 ± 2
**5**	**6a**	$Rh_2(cap)_4$	CH_2Cl_2	13.5 ± 2
**5**	**6g**	$Rh_2(cap)_4$	CH_2Cl_2	15.2 ± 2
**5**	**6h**	$Rh_2(cap)_4$	CH_2Cl_2	13.3 ± 3
**5**	**6i**	$Rh_2(cap)_4$	CH_2Cl_2	12.4 ± 2
**5**	None	$Rh_2(cap)_4$	CH_2Cl_2	11.7 ± 2
**5**	**6f**	$Rh_2(S\text{-}DOSP)_4$	PhMe	15.1 ± 2
**5**	None	$Rh_2(S\text{-}DOSP)_4$	PhMe	14.9 ± 2
**5**	**6b**	$Rh_2(TFA)_4$	PhMe	13.1 ± 2
**5**	**6c**	$Rh_2(TFA)_4$	PhMe	13.4 ± 4
**5**	**6h**	$Rh_2(TFA)_4$	PhMe	14.1 ± 2
**5**	**6i**	$Rh_2(TFA)_4$	PhMe	14.7 ± 1
**5**	None	$Rh_2(TFA)_4$	PhMe	13.7 ± 2
**5**	**6d**	$Rh_2(5R\text{-}MEPY)_4$	PhMe	9.6 ± 3
**5**	**6e**	$Rh_2(5R\text{-}MEPY)_4$	PhMe	9.7 ± 2
**5**	**6f**	$Rh_2(5R\text{-}MEPY)_4$	PhMe	11.1 ± 3
**5**	**6g**	$Rh_2(5R\text{-}MEPY)_4$	PhMe	11.7 ± 2
**5**	None	$Rh_2(Oct)_4$	PhMe	11.3 ± 2
**5**	**6b**	$Rh_2(Oct)_4$	PhMe	9 ± 2
**5**	**6b**	$Rh_2(4S\text{-}MEOX)_4$	PhMe	12 ± 2
**5**	**6e**	$Rh_2(4S\text{-}MEOX)_4$	PhMe	12.2 ± 2
**5**	**6f**	$Rh_2(4S\text{-}MEOX)_4$	PhMe	11.3 ± 3
**5**	**6h**	$Rh_2(4S\text{-}MEOX)_4$	PhMe	13.4 ± 2
**5**	None	$Rh_2(4S\text{-}MEOX)_4$	PhMe	13.8 ± 2
**5**	**6a**	$Rh_2(cap)_4$	PhMe	7.3 ± 3
**5**	**6c**	$Rh_2(cap)_4$	PhMe	6 ± 2
**5**	**6d**	$Rh_2(cap)_4$	PhMe	9.1 ± 1
**5**	**6i**	$Rh_2(cap)_4$	PhMe	10.5 ± 2
**5**	None	$Rh_2(cap)_4$	PhMe	8 ± 3

a: POA = Percentage of Activity relative to 5 μM Testosterone solution.

Reaction array 2 data: Activities of product mixtures prepared in round two. Product mixtures were assayed at a total product concentration of 10 mM and 5 μM following General Procedure G.

Diazo-substrate	Co substrate	Catalyst	Solvent	POA at [10 μM][a]
**3**	**6a**	Rh_2(S-DOSP)$_4$	CH_2Cl_2	48.4 ± 3
**3**	**6f**	Rh_2(S-DOSP)$_4$	CH_2Cl_2	47.1 ± 3
**3**	**6j**	Rh_2(S-DOSP)$_4$	CH_2Cl_2	51.8 ± 4
**3**	**6k**	Rh_2(S-DOSP)$_4$	CH_2Cl_2	52.4 ± 2
**3**	**6l**	Rh_2(S-DOSP)$_4$	CH_2Cl_2	47 ± 2
**3**	**6m**	Rh_2(S-DOSP)$_4$	CH_2Cl_2	70.7 ± 5
**3**	**6n**	Rh_2(S-DOSP)$_4$	CH_2Cl_2	52 ± 3
**3**	**6o**	Rh_2(S-DOSP)$_4$	CH_2Cl_2	69.6 ± 2
**3**	**6p**	Rh_2(S-DOSP)$_4$	CH_2Cl_2	38.1 ± 4
**3**	**6q**	Rh_2(S-DOSP)$_4$	CH_2Cl_2	47 ± 4
**3**	**6r**	Rh_2(S-DOSP)$_4$	CH_2Cl_2	46.9 ± 3
**3**	**6s**	Rh_2(S-DOSP)$_4$	CH_2Cl_2	45.4 ± 4
**3**	**6t**	Rh_2(S-DOSP)$_4$	CH_2Cl_2	19.2 ± 3
**3**	**6u**	Rh_2(S-DOSP)$_4$	CH_2Cl_2	46.3 ± 4
**3**	**6v**	Rh_2(S-DOSP)$_4$	CH_2Cl_2	32.1 ± 3
**3**	**6w**	Rh_2(S-DOSP)$_4$	CH_2Cl_2	53.1 ± 3
**3**	**6x**	Rh_2(S-DOSP)$_4$	CH_2Cl_2	29.1 ± 2
**3**	**6y**	Rh_2(S-DOSP)$_4$	CH_2Cl_2	47.8 ± 2
**3**	**6a**	$Rh_2(OAc)_4$	CH_2Cl_2	37.6 ± 3
**3**	**6f**	$Rh_2(OAc)_4$	CH_2Cl_2	44.3 ± 3
**3**	**6q**	$Rh_2(OAc)_4$	CH_2Cl_2	22.1 ± 2
**3**	**6r**	$Rh_2(OAc)_4$	CH_2Cl_2	44.2 ± 2
**3**	**6a**	$Rh_2(pfb)_4$	CH_2Cl_2	35 ± 3
**3**	**6f**	$Rh_2(pfb)_4$	CH_2Cl_2	35.3 ± 2
**3**	**6j**	$Rh_2(pfb)_4$	CH_2Cl_2	29.8 ± 3
**3**	**6n**	$Rh_2(pfb)_4$	CH_2Cl_2	37 ± 2
**3**	**6p**	$Rh_2(pfb)_4$	CH_2Cl_2	48.7 ± 2
**3**	**6v**	$Rh_2(pfb)_4$	CH_2Cl_2	32.9 ± 2
**3**	**6a**	$Rh_2(tpa)_4$	CH_2Cl_2	51 ± 3
**3**	**6f**	$Rh_2(tpa)_4$	CH_2Cl_2	42.5 ± 3
**3**	**6m**	$Rh_2(tpa)_4$	CH_2Cl_2	55.3 ± 3
**3**	**6a**	$Rh_2(esp)_2$	CH_2Cl_2	41.2 ± 2
**3**	**6f**	$Rh_2(esp)_2$	CH_2Cl_2	41.6 ± 2
**3**	**6r**	$Rh_2(esp)_2$	CH_2Cl_2	72.8 ± 3
**3**	**6x**	$Rh_2(esp)_2$	CH_2Cl_2	24.8 ± 7
**3**	**6a**	Rh_2(S-DOSP)$_4$	PhMe	33.7 ± 1
**3**	**6f**	Rh_2(S-DOSP)$_4$	PhMe	37.3 ± 4
**3**	**6v**	Rh_2(S-DOSP)$_4$	PhMe	25.5 ± 2
**3**	**6a**	$Rh_2(OAc)_4$	PhMe	33 ± 3
**3**	**6s**	$Rh_2(OAc)_4$	PhMe	28 ± 3
**3**	**6t**	$Rh_2(OAc)_4$	PhMe	28.9 ± 4
**3**	**6w**	$Rh_2(OAc)_4$	PhMe	25.8 ± 4
**3**	**6y**	$Rh_2(OAc)_4$	PhMe	20.6 ± 5

**3**	**6j**	$Rh_2(pfb)_4$	PhMe	9 ± 3
**3**	**6o**	$Rh_2(pfb)_4$	PhMe	4.6 ± 2
**3**	**6j**	$Rh_2(tpa)_4$	PhMe	5.9 ± 3
**3**	**6l**	$Rh_2(tpa)_4$	PhMe	1.2 ± 3
**3**	**6v**	$Rh_2(tpa)_4$	PhMe	10.4 ± 2
**3**	**6x**	$Rh_2(tpa)_4$	PhMe	0.7 ± 3
**3**	**6y**	$Rh_2(tpa)_4$	PhMe	8.2 ± 3
**3**	**6a**	$Rh_2(esp)_2$	PhMe	0.8 ± 2
**3**	**6m**	$Rh_2(esp)_2$	PhMe	1.2 ± 2
**3**	**6n**	$Rh_2(esp)_2$	PhMe	8.6 ± 3
**3**	**6o**	$Rh_2(esp)_2$	PhMe	1.8 ± 3
**3**	**6p**	$Rh_2(esp)_2$	PhMe	3.9 ± 3
**3**	**6u**	$Rh_2(esp)_2$	PhMe	4.9 ± 2
**3**	**6v**	$Rh_2(esp)_2$	PhMe	3.7 ± 2
**5**	**6a**	$Rh_2(S\text{-}DOSP)_4$	CH_2Cl_2	19.6 ± 3
**5**	**6f**	$Rh_2(S\text{-}DOSP)_4$	CH_2Cl_2	22 ± 2
**5**	**6j**	$Rh_2(OAc)_4$	CH_2Cl_2	15.6 ± 2
**5**	**6m**	$Rh_2(OAc)_4$	CH_2Cl_2	25.3 ± 3
**5**	**6n**	$Rh_2(OAc)_4$	CH_2Cl_2	50 ± 3
**5**	**6l**	$Rh_2(pfb)_4$	CH_2Cl_2	43.3 ± 2
**5**	**6v**	$Rh_2(pfb)_4$	CH_2Cl_2	36.1 ± 4
**5**	**6y**	$Rh_2(pfb)_4$	CH_2Cl_2	4.4 ± 2
**5**	**6j**	$Rh_2(tpa)_4$	CH_2Cl_2	42.9 ± 4
**5**	**6k**	$Rh_2(tpa)_4$	CH_2Cl_2	33.8 ± 3
**5**	**6l**	$Rh_2(tpa)_4$	CH_2Cl_2	43.2 ± 2
**5**	**6q**	$Rh_2(tpa)_4$	CH_2Cl_2	37.7 ± 4
**5**	**6x**	$Rh_2(esp)_2$	CH_2Cl_2	44.7 ± 2
**5**	**6j**	$Rh_2(S\text{-}DOSP)_4$	PhMe	39.7 ± 5
**5**	**6k**	$Rh_2(S\text{-}DOSP)_4$	PhMe	34.2 ± 3
**5**	**6r**	$Rh_2(S\text{-}DOSP)_4$	PhMe	32.6 ± 3
**5**	**6u**	$Rh_2(S\text{-}DOSP)_4$	PhMe	33.2 ± 2
**5**	**6y**	$Rh_2(S\text{-}DOSP)_4$	PhMe	36.8 ± 3
**5**	**6f**	$Rh_2(OAc)_4$	PhMe	44.9 ± 6
**5**	**6j**	$Rh_2(OAc)_4$	PhMe	38.4 ± 3
**5**	**6l**	$Rh_2(OAc)_4$	PhMe	42.4 ± 2
**5**	**6y**	$Rh_2(OAc)_4$	PhMe	21.8 ± 2
**5**	**6r**	$Rh_2(pfb)_4$	PhMe	28.6 ± 3
**5**	**6k**	$Rh_2(tpa)_4$	PhMe	32 ± 5
**5**	**6n**	$Rh_2(tpa)_4$	PhMe	37.5 ± 3
**5**	**6p**	$Rh_2(tpa)_4$	PhMe	30.4 ± 2
**5**	**6v**	$Rh_2(tpa)_4$	PhMe	27.7 ± 4
**5**	**6k**	$Rh_2(esp)_2$	PhMe	33.1 ± 4
**5**	**6t**	$Rh_2(esp)_2$	PhMe	6.6 ± 2

a: POA = Percentage of Activity relative to 5 μM Testosterone solution.

**Diazo-substrate**	**Co substrate**	**Catalyst**	**Solvent**	**POA at [5 μM][a]**
**3**	**6a**	Rh_2(S-DOSP)$_4$	CH_2Cl_2	17.5 ± 2
**3**	**6f**	Rh_2(S-DOSP)$_4$	CH_2Cl_2	13.1 ± 3
**3**	**6j**	Rh_2(S-DOSP)$_4$	CH_2Cl_2	18.2 ± 4
**3**	**6k**	Rh_2(S-DOSP)$_4$	CH_2Cl_2	20 ± 3
**3**	**6l**	Rh_2(S-DOSP)$_4$	CH_2Cl_2	15.9 ± 5
**3**	**6m**	Rh_2(S-DOSP)$_4$	CH_2Cl_2	39.9 ± 3
**3**	**6n**	Rh_2(S-DOSP)$_4$	CH_2Cl_2	19.6 ± 2
**3**	**6o**	Rh_2(S-DOSP)$_4$	CH_2Cl_2	43.2 ± 4
**3**	**6p**	Rh_2(S-DOSP)$_4$	CH_2Cl_2	17.7 ± 7
**3**	**6q**	Rh_2(S-DOSP)$_4$	CH_2Cl_2	20.8 ± 3
**3**	**6r**	Rh_2(S-DOSP)$_4$	CH_2Cl_2	20.4 ± 5
**3**	**6s**	Rh_2(S-DOSP)$_4$	CH_2Cl_2	19.5 ± 3
**3**	**6t**	Rh_2(S-DOSP)$_4$	CH_2Cl_2	10.5 ± 5
**3**	**6u**	Rh_2(S-DOSP)$_4$	CH_2Cl_2	20.2 ± 4
**3**	**6v**	Rh_2(S-DOSP)$_4$	CH_2Cl_2	9.5 ± 2
**3**	**6w**	Rh_2(S-DOSP)$_4$	CH_2Cl_2	18.3 ± 5
**3**	**6x**	Rh_2(S-DOSP)$_4$	CH_2Cl_2	12.5 ± 3
**3**	**6y**	Rh_2(S-DOSP)$_4$	CH_2Cl_2	21.1 ± 4
**3**	**6a**	$Rh_2(OAc)_4$	CH_2Cl_2	16.3 ± 5
**3**	**6f**	$Rh_2(OAc)_4$	CH_2Cl_2	18.4 ± 3
**3**	**6q**	$Rh_2(OAc)_4$	CH_2Cl_2	13 ± 4
**3**	**6r**	$Rh_2(OAc)_4$	CH_2Cl_2	21 ± 4
**3**	**6a**	$Rh_2(pfb)_4$	CH_2Cl_2	16 ± 5
**3**	**6f**	$Rh_2(pfb)_4$	CH_2Cl_2	15.4 ± 3
**3**	**6j**	$Rh_2(pfb)_4$	CH_2Cl_2	13 ± 3
**3**	**6n**	$Rh_2(pfb)_4$	CH_2Cl_2	17.1 ± 4
**3**	**6p**	$Rh_2(pfb)_4$	CH_2Cl_2	28.3 ± 3
**3**	**6v**	$Rh_2(pfb)_4$	CH_2Cl_2	13.4 ± 4
**3**	**6a**	$Rh_2(tpa)_4$	CH_2Cl_2	21.8 ± 4
**3**	**6f**	$Rh_2(tpa)_4$	CH_2Cl_2	20.8 ± 5
**3**	**6m**	$Rh_2(tpa)_4$	CH_2Cl_2	24.3 ± 4
**3**	**6a**	$Rh_2(esp)_2$	CH_2Cl_2	20.4 ± 4
**3**	**6f**	$Rh_2(esp)_2$	CH_2Cl_2	16 ± 5
**3**	**6r**	$Rh_2(esp)_2$	CH_2Cl_2	49.9 ± 4
**3**	**6x**	$Rh_2(esp)_2$	CH_2Cl_2	11.5 ± 3
**3**	**6a**	Rh_2(S-DOSP)$_4$	PhMe	15.6 ± 3
**3**	**6f**	Rh_2(S-DOSP)$_4$	PhMe	19.8 ± 3
**3**	**6v**	Rh_2(S-DOSP)$_4$	PhMe	13.9 ± 4
**3**	**6a**	$Rh_2(OAc)_4$	PhMe	20.8 ± 4
**3**	**6s**	$Rh_2(OAc)_4$	PhMe	16.1 ± 4
**3**	**6t**	$Rh_2(OAc)_4$	PhMe	20.6 ± 6
**3**	**6w**	$Rh_2(OAc)_4$	PhMe	14.5 ± 3
**3**	**6y**	$Rh_2(OAc)_4$	PhMe	13.8 ± 5
**3**	**6j**	$Rh_2(pfb)_4$	PhMe	3.8 ± 2
**3**	**6o**	$Rh_2(pfb)_4$	PhMe	5 ± 4
**3**	**6j**	$Rh_2(tpa)_4$	PhMe	6.7 ± 4
**3**	**6l**	$Rh_2(tpa)_4$	PhMe	1.8 ± 2
**3**	**6v**	$Rh_2(tpa)_4$	PhMe	0.4 ± 2

**3**	**6x**	$Rh_2(tpa)_4$	PhMe	4.3 ± 3
**3**	**6y**	$Rh_2(tpa)_4$	PhMe	1.1 ± 4
**3**	**6a**	$Rh_2(esp)_2$	PhMe	9.4 ± 4
**3**	**6m**	$Rh_2(esp)_2$	PhMe	3.2 ± 3
**3**	**6n**	$Rh_2(esp)_2$	PhMe	10.7 ± 3
**3**	**6o**	$Rh_2(esp)_2$	PhMe	9.1 ± 4
**3**	**6p**	$Rh_2(esp)_2$	PhMe	8 ± 4
**3**	**6u**	$Rh_2(esp)_2$	PhMe	2.6 ± 3
**3**	**6v**	$Rh_2(esp)_2$	PhMe	0.1 ± 0
**5**	**6a**	$Rh_2(S\text{-}DOSP)_4$	CH_2Cl_2	11.8 ± 4
**5**	**6f**	$Rh_2(S\text{-}DOSP)_4$	CH_2Cl_2	8.2 ± 3
**5**	**6j**	$Rh_2(OAc)_4$	CH_2Cl_2	11.1 ± 2
**5**	**6m**	$Rh_2(OAc)_4$	CH_2Cl_2	18.4 ± 3
**5**	**6n**	$Rh_2(OAc)_4$	CH_2Cl_2	37.7 ± 2
**5**	**6l**	$Rh_2(pfb)_4$	CH_2Cl_2	23.2 ± 5
**5**	**6v**	$Rh_2(pfb)_4$	CH_2Cl_2	20.1 ± 5
**5**	**6y**	$Rh_2(pfb)_4$	CH_2Cl_2	9 ± 3
**5**	**6j**	$Rh_2(tpa)_4$	CH_2Cl_2	21.6 ± 3
**5**	**6k**	$Rh_2(tpa)_4$	CH_2Cl_2	18.7 ± 4
**5**	**6l**	$Rh_2(tpa)_4$	CH_2Cl_2	28.5 ± 5
**5**	**6q**	$Rh_2(tpa)_4$	CH_2Cl_2	21.1 ± 2
**5**	**6x**	$Rh_2(esp)_2$	CH_2Cl_2	17.7 ± 4
**5**	**6j**	$Rh_2(S\text{-}DOSP)_4$	PhMe	16.3 ± 3
**5**	**6k**	$Rh_2(S\text{-}DOSP)_4$	PhMe	19.5 ± 3
**5**	**6r**	$Rh_2(S\text{-}DOSP)_4$	PhMe	15.8 ± 5
**5**	**6u**	$Rh_2(S\text{-}DOSP)_4$	PhMe	13.3 ± 3
**5**	**6y**	$Rh_2(S\text{-}DOSP)_4$	PhMe	18.6 ± 5
**5**	**6f**	$Rh_2(OAc)_4$	PhMe	39 ± 2
**5**	**6j**	$Rh_2(OAc)_4$	PhMe	23 ± 3
**5**	**6l**	$Rh_2(OAc)_4$	PhMe	21.9 ± 4
**5**	**6y**	$Rh_2(OAc)_4$	PhMe	11 ± 3
**5**	**6r**	$Rh_2(pfb)_4$	PhMe	14.9 ± 4
**5**	**6k**	$Rh_2(tpa)_4$	PhMe	16 ± 5
**5**	**6n**	$Rh_2(tpa)_4$	PhMe	22.5 ± 4
**5**	**6p**	$Rh_2(tpa)_4$	PhMe	18.5 ± 4
**5**	**6v**	$Rh_2(tpa)_4$	PhMe	14.9 ± 3
**5**	**6k**	$Rh_2(esp)_2$	PhMe	16.6 ± 6
**5**	**6t**	$Rh_2(esp)_2$	PhMe	6 ± 4

a: POA = Percentage of Activity relative to 5 μM Testosterone solution.

Reaction array 3 data: Activities of product mixtures prepared in round three. Product mixtures were assayed at a total product concentration of 5 μM and 1 μM following General Procedure G.

Diazo-substrate	Co substrate	Catalyst	Solvent	POA at [5 μM][a]
**3**	**6a**	Rh_2(S-DOSP)$_4$	CH_2Cl_2	39.3 ± 2
**3**	**6f**	Rh_2(S-DOSP)$_4$	CH_2Cl_2	33 ± 4
**3**	**6m**	Rh_2(S-DOSP)$_4$	CH_2Cl_2	40.8 ± 4
**3**	**6n**	Rh_2(S-DOSP)$_4$	CH_2Cl_2	26.6 ± 2
**3**	**6o**	Rh_2(S-DOSP)$_4$	CH_2Cl_2	43 ± 3
**3**	**6r**	Rh_2(S-DOSP)$_4$	CH_2Cl_2	30.3 ± 5
**3**	**6a'**	Rh_2(S-DOSP)$_4$	CH_2Cl_2	33.2 ± 4
**3**	**6b'**	Rh_2(S-DOSP)$_4$	CH_2Cl_2	32.3 ± 4
**3**	**6c'**	Rh_2(S-DOSP)$_4$	CH_2Cl_2	33.3 ± 5
**3**	**6d'**	Rh_2(S-DOSP)$_4$	CH_2Cl_2	41.3 ± 2
**3**	**6e'**	Rh_2(S-DOSP)$_4$	CH_2Cl_2	48.2 ± 5
**3**	**6f'**	Rh_2(S-DOSP)$_4$	CH_2Cl_2	40.2 ± 5
**3**	**6a**	Rh_2(R-DOSP)$_4$	CH_2Cl_2	31.6 ± 4
**3**	**6f**	Rh_2(R-DOSP)$_4$	CH_2Cl_2	36.5 ± 3
**3**	**6m**	Rh_2(R-DOSP)$_4$	CH_2Cl_2	33.9 ± 5
**3**	**6n**	Rh_2(R-DOSP)$_4$	CH_2Cl_2	26 ± 5
**3**	**6o**	Rh_2(R-DOSP)$_4$	CH_2Cl_2	27 ± 4
**3**	**6r**	Rh_2(R-DOSP)$_4$	CH_2Cl_2	32.9 ± 6
**3**	**6a'**	Rh_2(R-DOSP)$_4$	CH_2Cl_2	16.2 ± 4
**3**	**6b'**	Rh_2(R-DOSP)$_4$	CH_2Cl_2	32.4 ± 5
**3**	**6c'**	Rh_2(R-DOSP)$_4$	CH_2Cl_2	23.9 ± 4
**3**	**6d'**	Rh_2(R-DOSP)$_4$	CH_2Cl_2	33.3 ± 3
**3**	**6e'**	Rh_2(R-DOSP)$_4$	CH_2Cl_2	50.8 ± 4
**3**	**6f'**	Rh_2(R-DOSP)$_4$	CH_2Cl_2	86.9 ± 4
**3**	**6a**	$Rh_2(OAc)_4$	CH_2Cl_2	14.7 ± 5
**3**	**6f**	$Rh_2(OAc)_4$	CH_2Cl_2	22.7 ± 3
**3**	**6m**	$Rh_2(OAc)_4$	CH_2Cl_2	23.7 ± 6
**3**	**6n**	$Rh_2(OAc)_4$	CH_2Cl_2	19.8 ± 4
**3**	**6o**	$Rh_2(OAc)_4$	CH_2Cl_2	31 ± 3
**3**	**6r**	$Rh_2(OAc)_4$	CH_2Cl_2	31.5 ± 3
**3**	**6a'**	$Rh_2(OAc)_4$	CH_2Cl_2	25.9 ± 4
**3**	**6b'**	$Rh_2(OAc)_4$	CH_2Cl_2	19 ± 5
**3**	**6c'**	$Rh_2(OAc)_4$	CH_2Cl_2	20.7 ± 4
**3**	**6d'**	$Rh_2(OAc)_4$	CH_2Cl_2	16.2 ± 4
**3**	**6e'**	$Rh_2(OAc)_4$	CH_2Cl_2	87.1 ± 5
**3**	**6f'**	$Rh_2(OAc)_4$	CH_2Cl_2	19 ± 4
**3**	**6a**	$Rh_2(esp)_2$	CH_2Cl_2	15.1 ± 3
**3**	**6f**	$Rh_2(esp)_2$	CH_2Cl_2	13.4 ± 3
**3**	**6m**	$Rh_2(esp)_2$	CH_2Cl_2	14 ± 6
**3**	**6n**	$Rh_2(esp)_2$	CH_2Cl_2	2.1 ± 3
**3**	**6o**	$Rh_2(esp)_2$	CH_2Cl_2	11.4 ± 4
**3**	**6r**	$Rh_2(esp)_2$	CH_2Cl_2	48.5 ± 3
**3**	**6a'**	$Rh_2(esp)_2$	CH_2Cl_2	18.5 ± 4

**3**	**6b'**	$Rh_2(esp)_2$	CH_2Cl_2	12.6 ± 3
**3**	**6c'**	$Rh_2(esp)_2$	CH_2Cl_2	9 ± 3
**3**	**6d'**	$Rh_2(esp)_2$	CH_2Cl_2	24.7 ± 3
**3**	**6e'**	$Rh_2(esp)_2$	CH_2Cl_2	66 ± 4
**3**	**6f'**	$Rh_2(esp)_2$	CH_2Cl_2	1.9 ± 3

a: POA = Percentage of Activity relative to 5 μM Testosterone solution.

**Diazo-substrate**	**Co substrate**	**Catalyst**	**Solvent**	**POA at [1 μM][a]**
**3**	**6a**	$Rh_2(S\text{-}DOSP)_4$	CH_2Cl_2	9.7 ± 1
**3**	**6f**	$Rh_2(S\text{-}DOSP)_4$	CH_2Cl_2	10.5 ± 2
**3**	**6m**	$Rh_2(S\text{-}DOSP)_4$	CH_2Cl_2	11.7 ± 4
**3**	**6n**	$Rh_2(S\text{-}DOSP)_4$	CH_2Cl_2	9.3 ± 2
**3**	**6o**	$Rh_2(S\text{-}DOSP)_4$	CH_2Cl_2	11.2 ± 2
**3**	**6r**	$Rh_2(S\text{-}DOSP)_4$	CH_2Cl_2	9.6 ± 2
**3**	**6a'**	$Rh_2(S\text{-}DOSP)_4$	CH_2Cl_2	11 ± 1
**3**	**6b'**	$Rh_2(S\text{-}DOSP)_4$	CH_2Cl_2	10.9 ± 4
**3**	**6c'**	$Rh_2(S\text{-}DOSP)_4$	CH_2Cl_2	7.8 ± 2
**3**	**6d'**	$Rh_2(S\text{-}DOSP)_4$	CH_2Cl_2	4.5 ± 2
**3**	**6e'**	$Rh_2(S\text{-}DOSP)_4$	CH_2Cl_2	23.7 ± 2
**3**	**6f'**	$Rh_2(S\text{-}DOSP)_4$	CH_2Cl_2	9.3 ± 2
**3**	**6a**	$Rh_2(R\text{-}DOSP)_4$	CH_2Cl_2	5.6 ± 3
**3**	**6f**	$Rh_2(R\text{-}DOSP)_4$	CH_2Cl_2	6.8 ± 2
**3**	**6m**	$Rh_2(R\text{-}DOSP)_4$	CH_2Cl_2	7 ± 2
**3**	**6n**	$Rh_2(R\text{-}DOSP)_4$	CH_2Cl_2	5.6 ± 3
**3**	**6o**	$Rh_2(R\text{-}DOSP)_4$	CH_2Cl_2	7.5 ± 3
**3**	**6r**	$Rh_2(R\text{-}DOSP)_4$	CH_2Cl_2	5.6 ± 3
**3**	**6a'**	$Rh_2(R\text{-}DOSP)_4$	CH_2Cl_2	7.1 ± 4
**3**	**6b'**	$Rh_2(R\text{-}DOSP)_4$	CH_2Cl_2	9.4 ± 1
**3**	**6c'**	$Rh_2(R\text{-}DOSP)_4$	CH_2Cl_2	8.1 ± 3
**3**	**6d'**	$Rh_2(R\text{-}DOSP)_4$	CH_2Cl_2	7.5 ± 2
**3**	**6e'**	$Rh_2(R\text{-}DOSP)_4$	CH_2Cl_2	26.2 ± 3
**3**	**6f'**	$Rh_2(R\text{-}DOSP)_4$	CH_2Cl_2	38.5 ± 3
**3**	**6a**	$Rh_2(OAc)_4$	CH_2Cl_2	4.7 ± 2
**3**	**6f**	$Rh_2(OAc)_4$	CH_2Cl_2	3.6 ± 2
**3**	**6m**	$Rh_2(OAc)_4$	CH_2Cl_2	5.9 ± 3
**3**	**6n**	$Rh_2(OAc)_4$	CH_2Cl_2	6.6 ± 2
**3**	**6o**	$Rh_2(OAc)_4$	CH_2Cl_2	8.7 ± 1
**3**	**6r**	$Rh_2(OAc)_4$	CH_2Cl_2	8.5 ± 2
**3**	**6a'**	$Rh_2(OAc)_4$	CH_2Cl_2	4.1 ± 3
**3**	**6b'**	$Rh_2(OAc)_4$	CH_2Cl_2	9 ± 2
**3**	**6c'**	$Rh_2(OAc)_4$	CH_2Cl_2	3.6 ± 2
**3**	**6d'**	$Rh_2(OAc)_4$	CH_2Cl_2	1.9 ± 3
**3**	**6e'**	$Rh_2(OAc)_4$	CH_2Cl_2	51.2 ± 2
**3**	**6f'**	$Rh_2(OAc)_4$	CH_2Cl_2	7.2 ± 1
**3**	**6a**	$Rh_2(esp)_2$	CH_2Cl_2	3.7 ± 2
**3**	**6f**	$Rh_2(esp)_2$	CH_2Cl_2	2.6 ± 1
**3**	**6m**	$Rh_2(esp)_2$	CH_2Cl_2	3.5 ± 3
**3**	**6n**	$Rh_2(esp)_2$	CH_2Cl_2	4.1 ± 1
**3**	**6o**	$Rh_2(esp)_2$	CH_2Cl_2	5 ± 2

**3**	**6r**	$Rh_2(esp)_2$	CH_2Cl_2	2.6 ± 0
**3**	**6a'**	$Rh_2(esp)_2$	CH_2Cl_2	3.5 ± 2
**3**	**6b'**	$Rh_2(esp)_2$	CH_2Cl_2	3.2 ± 1
**3**	**6c'**	$Rh_2(esp)_2$	CH_2Cl_2	3.2 ± 3
**3**	**6d'**	$Rh_2(esp)_2$	CH_2Cl_2	7.2 ± 2
**3**	**6e'**	$Rh_2(esp)_2$	CH_2Cl_2	34.6 ± 2
**3**	**6f'**	$Rh_2(esp)_2$	CH_2Cl_2	4.7 ± 2

a: POA = Percentage of Activity relative to 5 μM Testosterone solution.

10 LCMS analysis of product mixtures from round 1

LCMS analysis was using a Phenomenex Luna C18(2), 50x2mm, 5μ particle size column using a gradient elution of 95:5 water-MeCN (0.1% Formic Acid) mix for 1.8 min, 90:0 water-MeCN (0.1% Formic Acid) mix for 0.7 min and 95:5 water-MeCN (0.1% Formic Acid) mix for 0.5 min. Uv detection was at 260 nm. In addition to the base peak chromatogram extracted ion chromatograms were generated for masses corresponding to the products of intra and intermolecular reactions, dimerization and hydrolysis. A total of twenty product mixtures form round 1 (*ca*. 10%) were randomly selected for analysis.

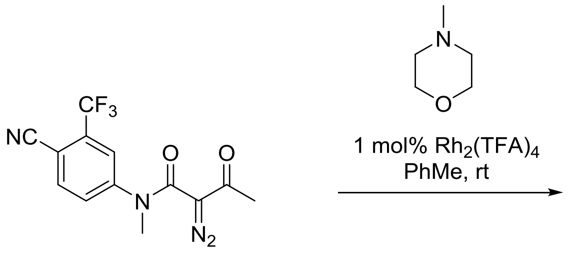

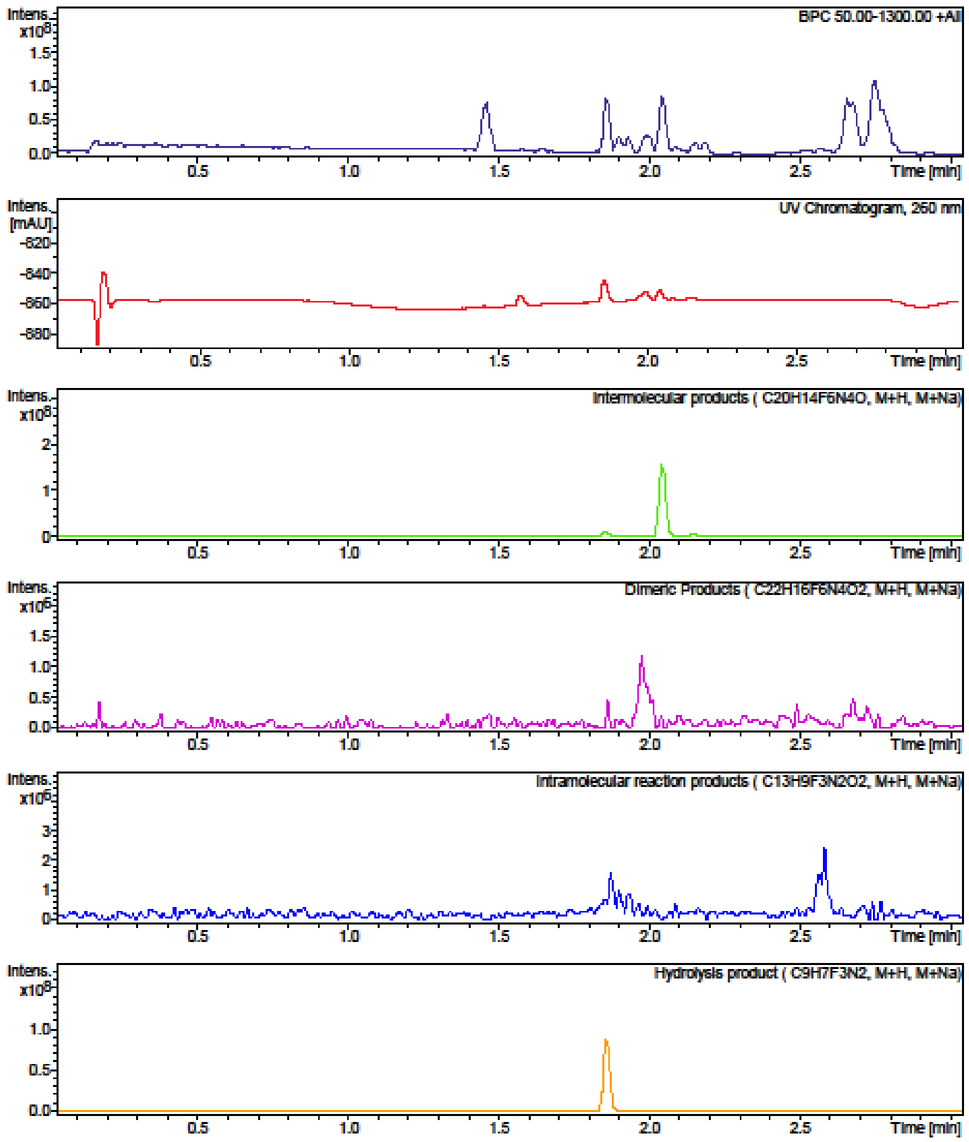

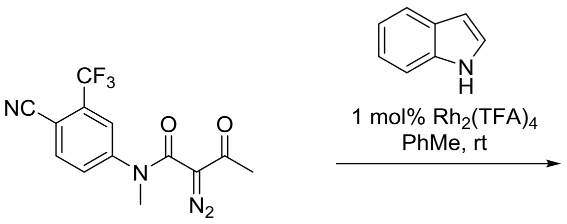
CF3
NC
O
O
N
N2
N
H
1 mol% Rh2(TFA)4
PhMe, rt

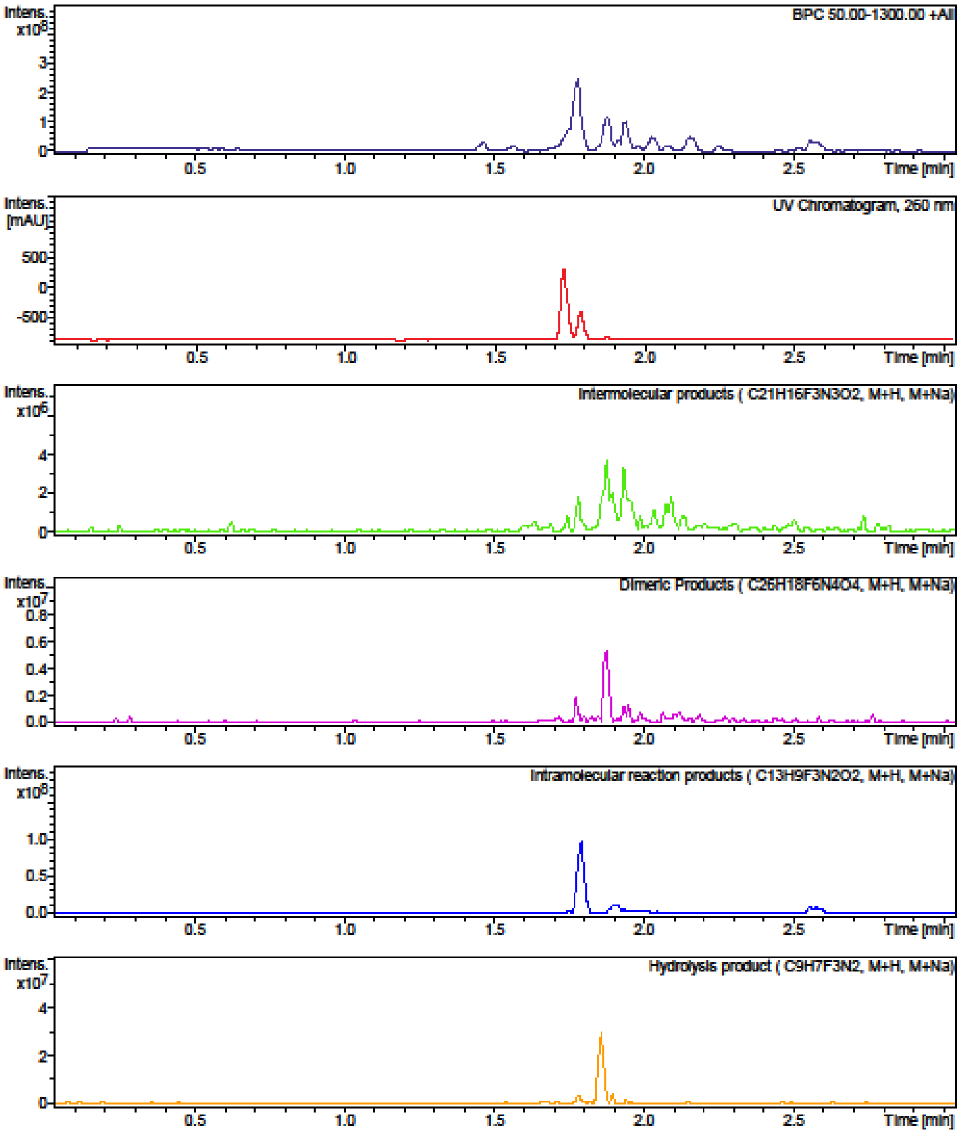
BPC 50.00-1300.00 +All
UV Chromatogram, 260 nm
Intermolecular products (C21H16F3N3O2, M+H, M+Na)
Dimeric Products (C26H18F6N4O4, M+H, M+Na)
Intramolecular reaction products (C13H9F3N2O2, M+H, M+Na)
Hydrolysis product (C9H7F3N2, M+H, M+Na)
Intens.
Time [min]

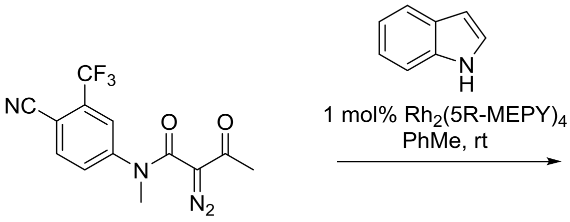
CF3
NC
O
O
N
N2
N
H
1 mol% Rh2(5R-MEPY)4
PhMe, rt

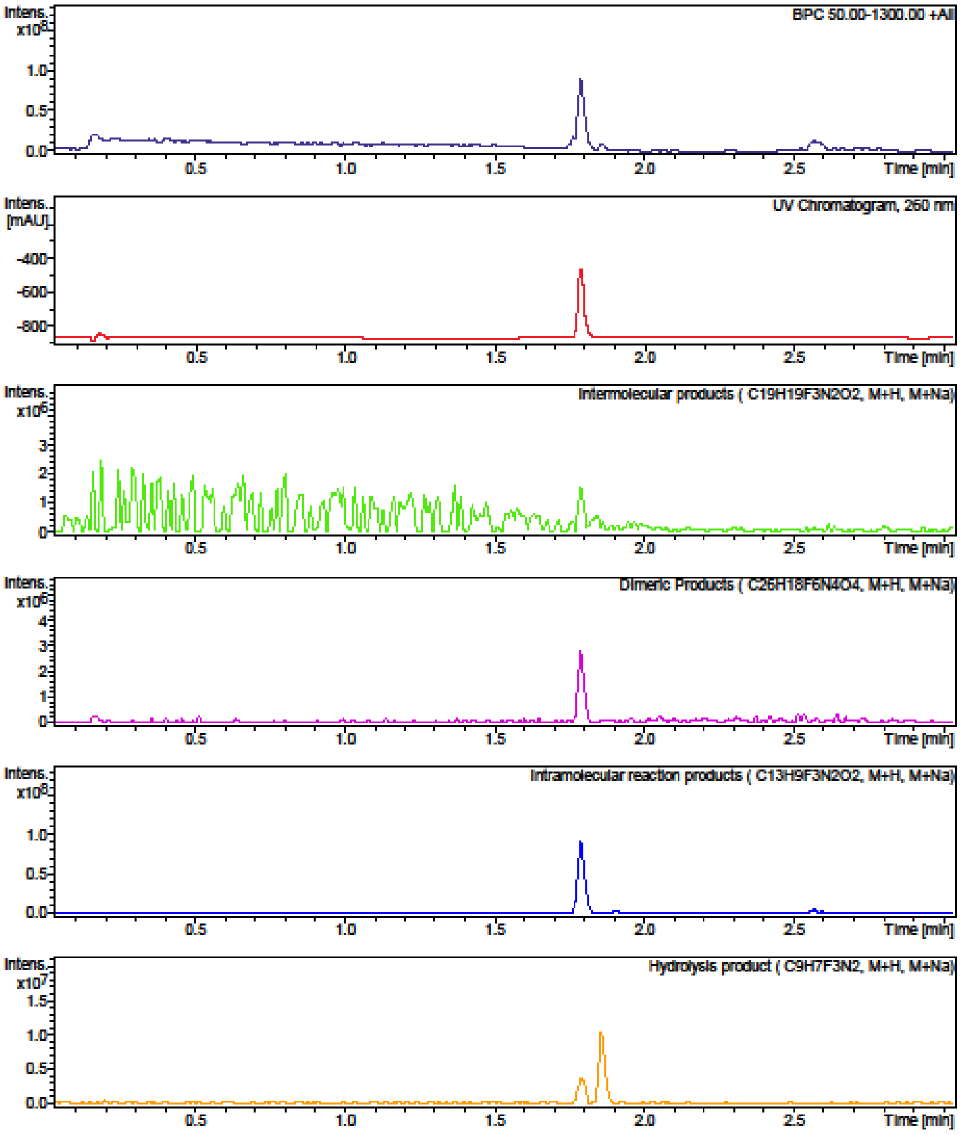
BPC 50.00-1300.00 +All
UV Chromatogram, 260 nm
Intermolecular products (C19H19F3N2O2, M+H, M+Na)
Dimeric Products (C26H18F6N4O4, M+H, M+Na)
Intramolecular reaction products (C13H9F3N2O2, M+H, M+Na)
Hydrolysis product (C9H7F3N2, M+H, M+Na)
Intens.
Time [min]

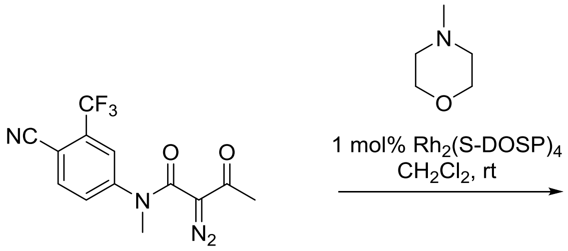
NC
CF3
O
O
N
N2
N
O
1 mol% Rh2(S-DOSP)4
CH2Cl2, rt

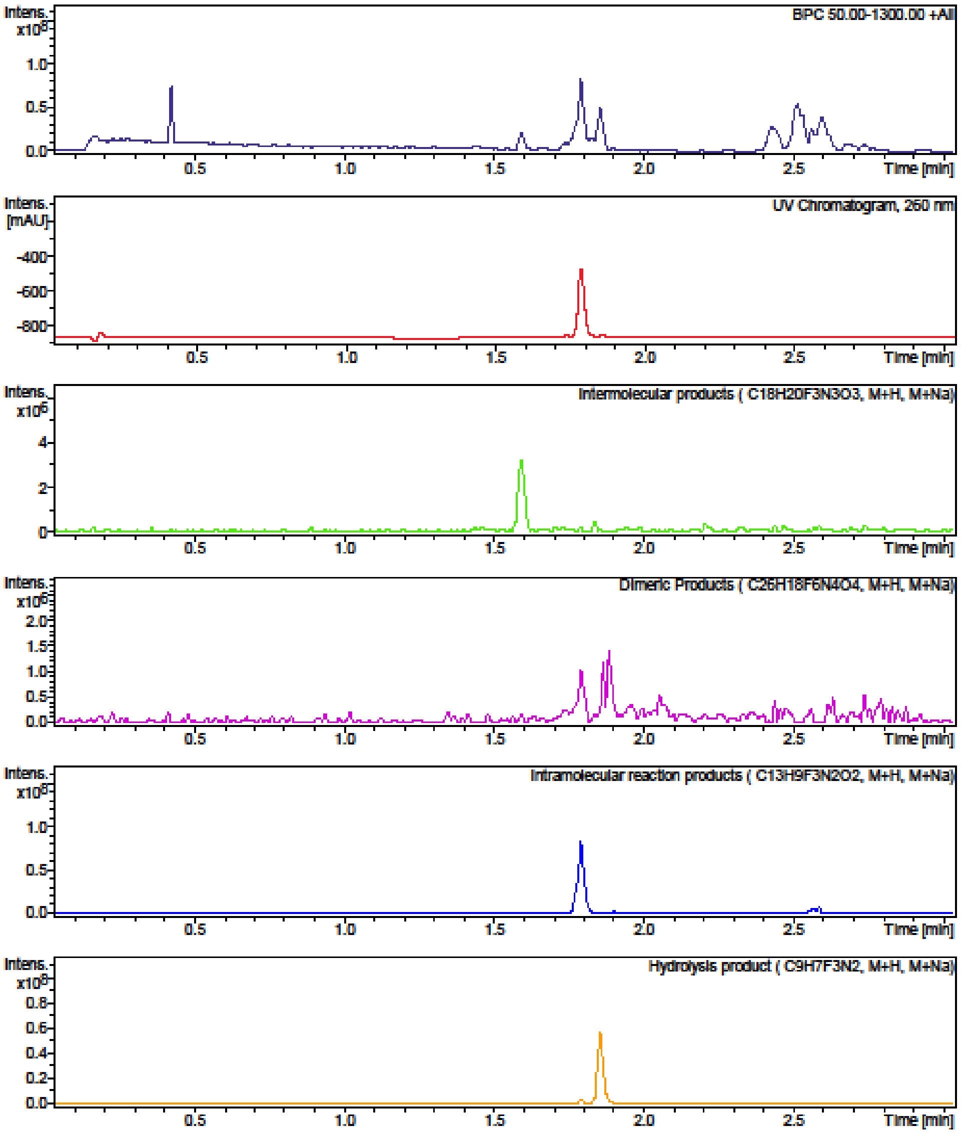
BPC 50.00-1300.00 +All
UV Chromatogram, 260 nm
Intermolecular products (C18H20F3N3O3, M+H, M+Na)
Dimeric Products (C26H18F6N4O4, M+H, M+Na)
Intramolecular reaction products (C13H9F3N2O2, M+H, M+Na)
Hydrolysis product (C9H7F3N2, M+H, M+Na)
Intens.
x10^8
x10^6
[mAU]
Time [min]

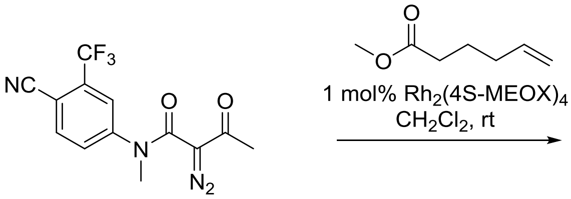
CF3
NC
O
O
N
N2
O
O
1 mol% Rh2(4S-MEOX)4
CH2Cl2, rt

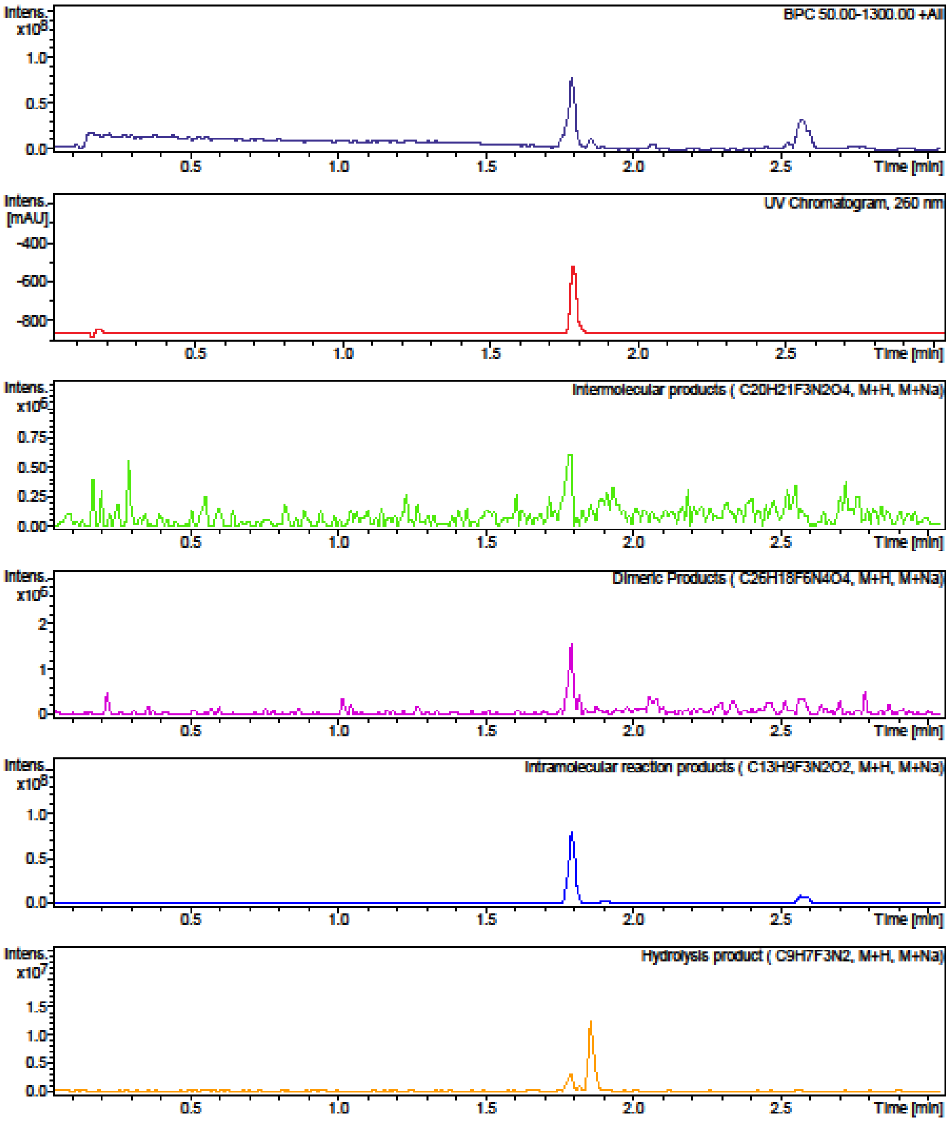
BPC 50.00-1300.00 +All
UV Chromatogram, 260 nm
Intermolecular products (C20H21F3N2O4, M+H, M+Na)
Dimeric Products (C26H18F6N4O4, M+H, M+Na)
Intramolecular reaction products (C13H9F3N2O2, M+H, M+Na)
Hydrolysis product (C9H7F3N2, M+H, M+Na)
Intens.
Time [min]

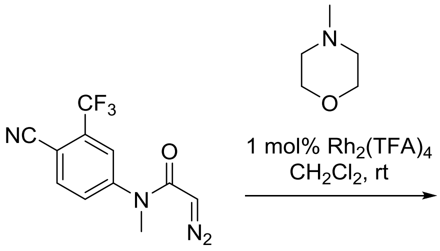
NC
CF3
O
N
N2
N
O
1 mol% Rh2(TFA)4
CH2Cl2, rt

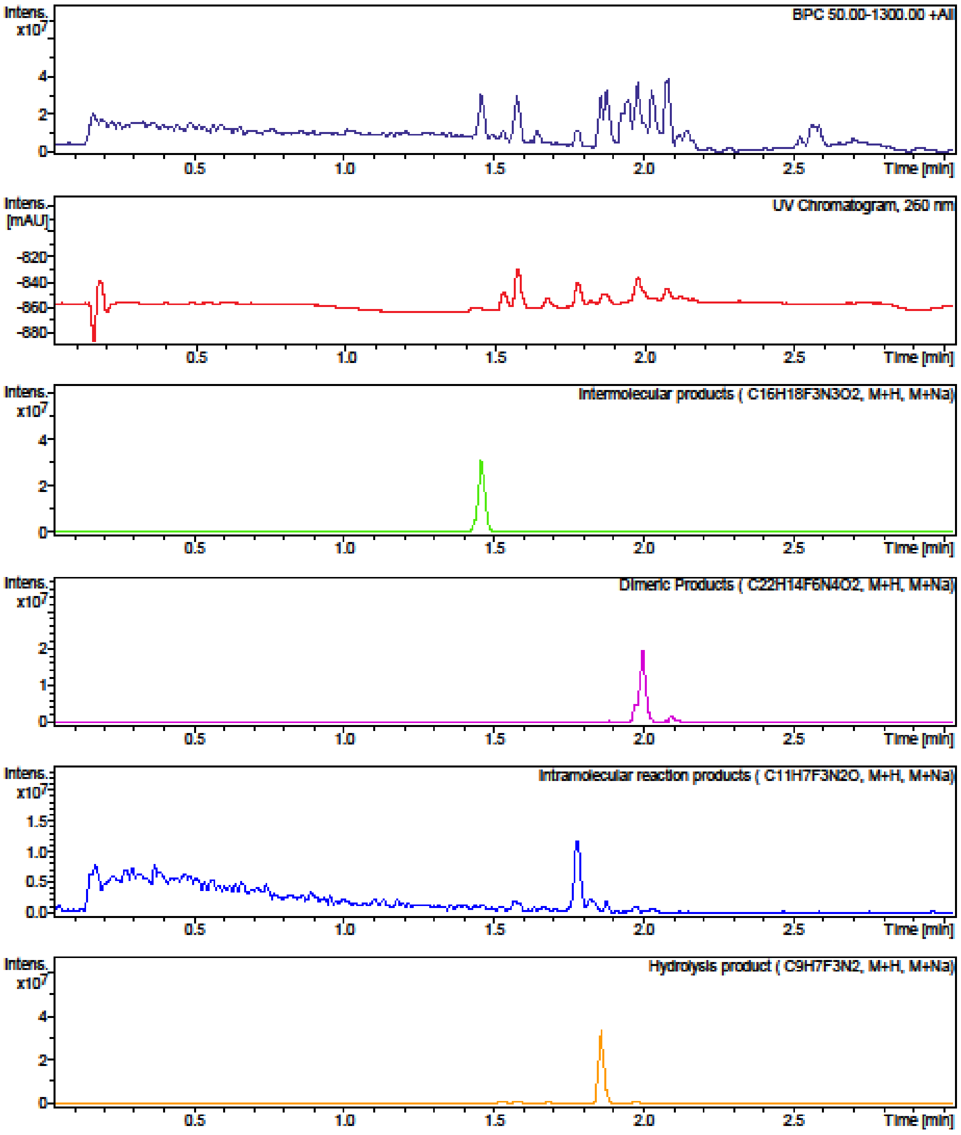
BPC 50.00-1300.00 +All
UV Chromatogram, 260 nm
Intermolecular products (C16H18F3N3O2, M+H, M+Na)
Dimeric Products (C22H14F6N4O2, M+H, M+Na)
Intramolecular reaction products (C11H7F3N2O, M+H, M+Na)
Hydrolysis product (C9H7F3N2, M+H, M+Na)
Intens. x10^7
Intens. [mAU]
Time [min]

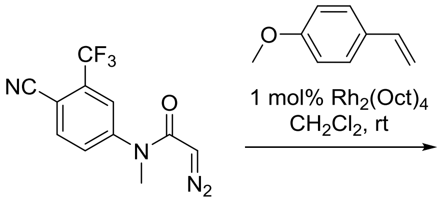
NC
CF3
O
N
N2
O
1 mol% Rh2(Oct)4
CH2Cl2, rt

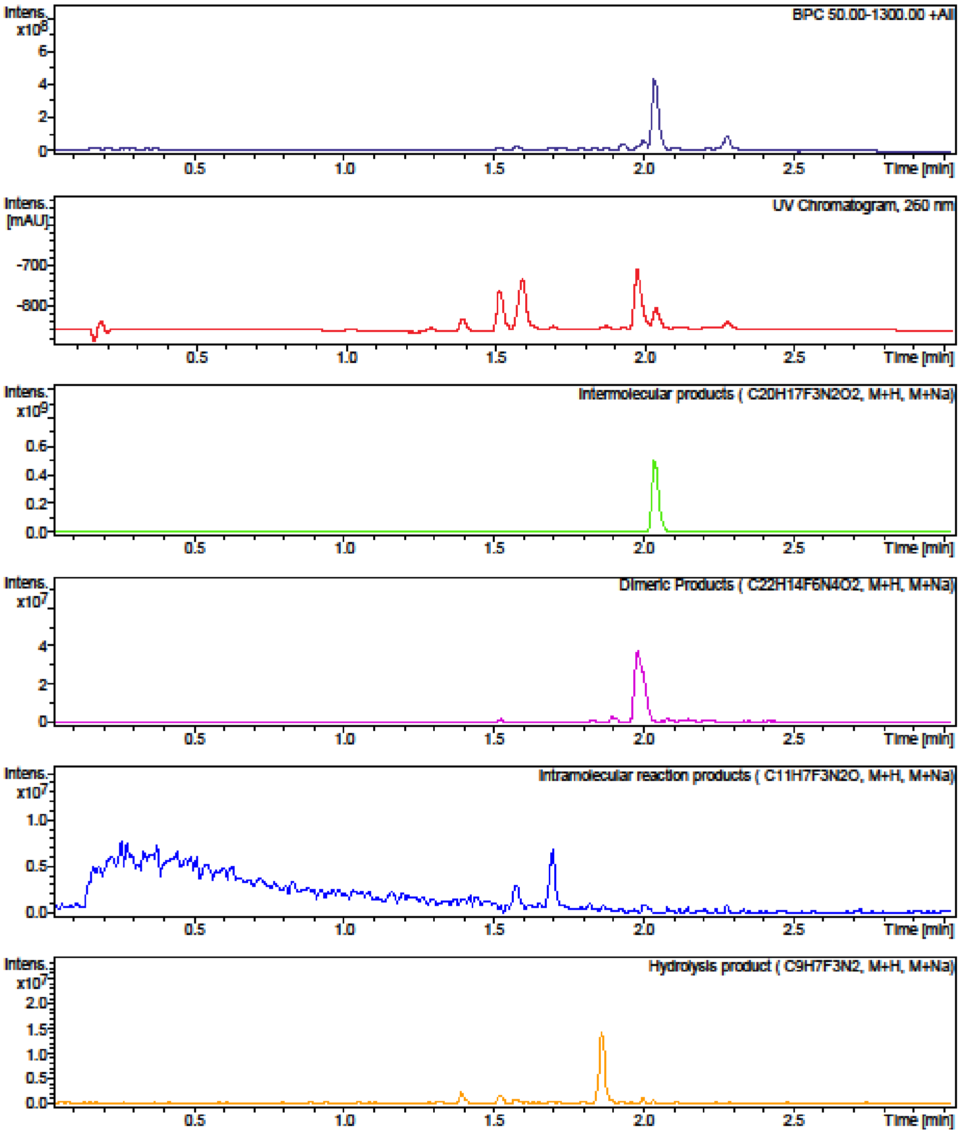
BPC 50.00-1300.00 +All
UV Chromatogram, 260 nm
Intermolecular products (C20H17F3N2O2, M+H, M+Na)
Dimeric Products (C22H14F6N4O2, M+H, M+Na)
Intramolecular reaction products (C11H7F3N2O, M+H, M+Na)
Hydrolysis product (C9H7F3N2, M+H, M+Na)
Intens.
Time [min]

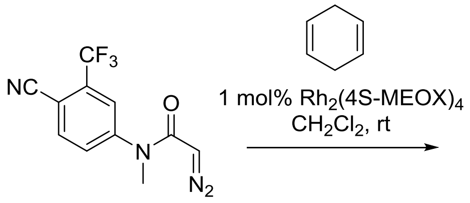
CF3
NC
O
N
N2
1 mol% Rh2(4S-MEOX)4
CH2Cl2, rt

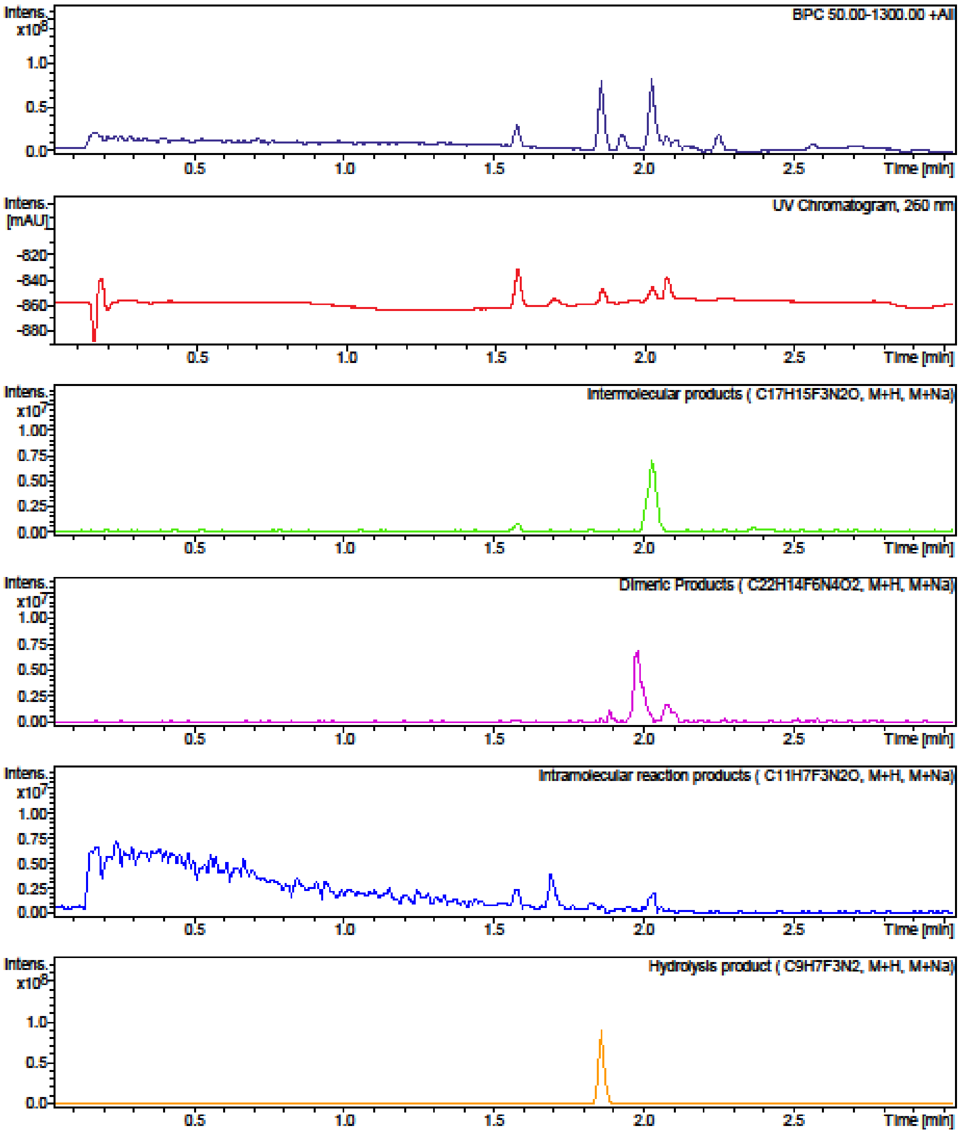
BPC 50.00-1300.00 +All
UV Chromatogram, 260 nm
Intermolecular products (C17H15F3N2O, M+H, M+Na)
Dimeric Products (C22H14F6N4O2, M+H, M+Na)
Intramolecular reaction products (C11H7F3N2O, M+H, M+Na)
Hydrolysis product (C9H7F3N2, M+H, M+Na)
Intens.
x10^8
[mAU]
x10^7
Time [min]

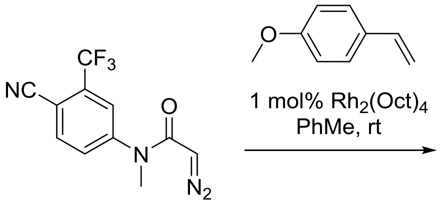

CF3
NC
O
N
N2
O
1 mol% Rh2(Oct)4
PhMe, rt

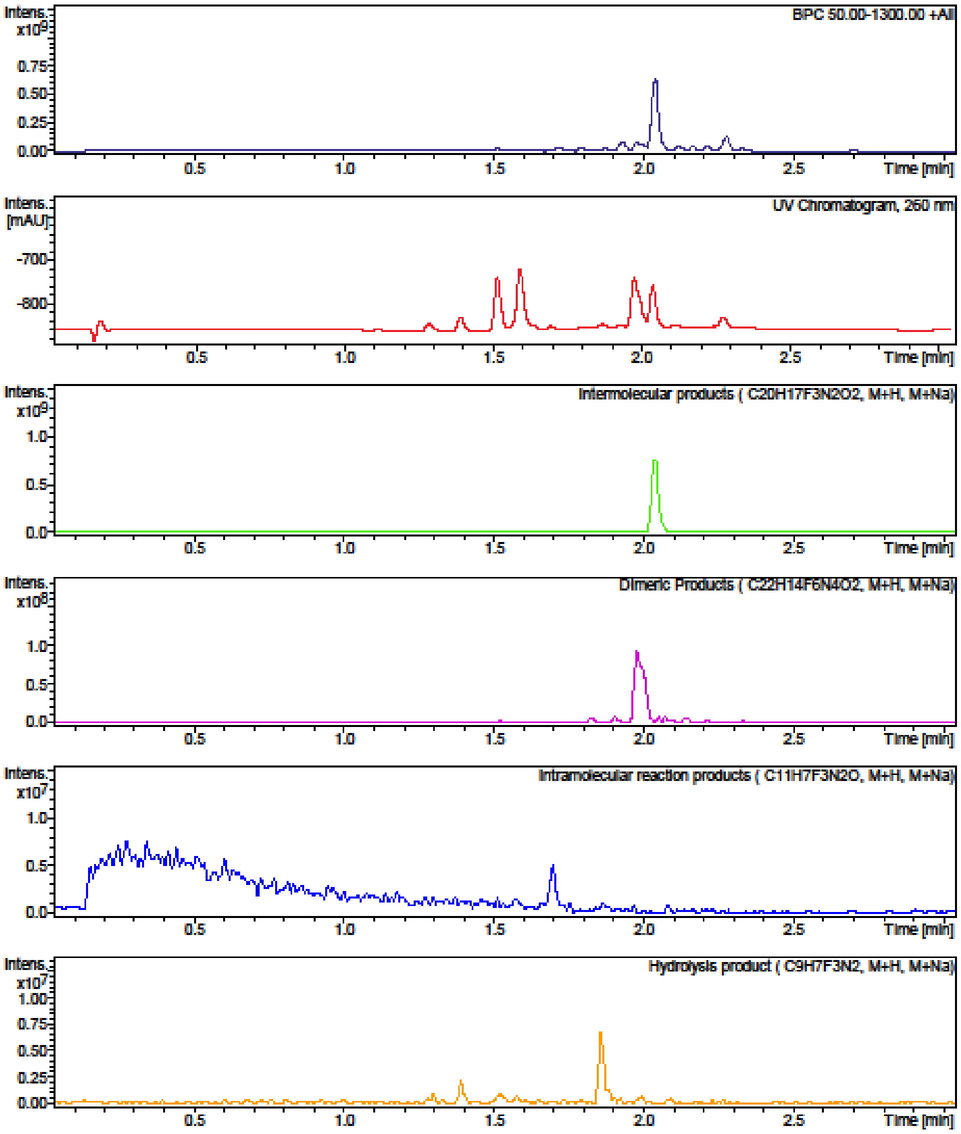

BPC 50.00-1300.00 +All
UV Chromatogram, 260 nm
Intermolecular products (C20H17F3N2O2, M+H, M+Na)
Dimeric Products (C22H14F6N4O2, M+H, M+Na)
Intramolecular reaction products (C11H7F3N2O, M+H, M+Na)
Hydrolysis product (C9H7F3N2, M+H, M+Na)
Intens.
Time [min]

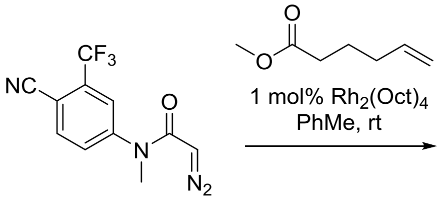
CF3
NC
O
N
N2
O
O
1 mol% Rh2(Oct)4
PhMe, rt

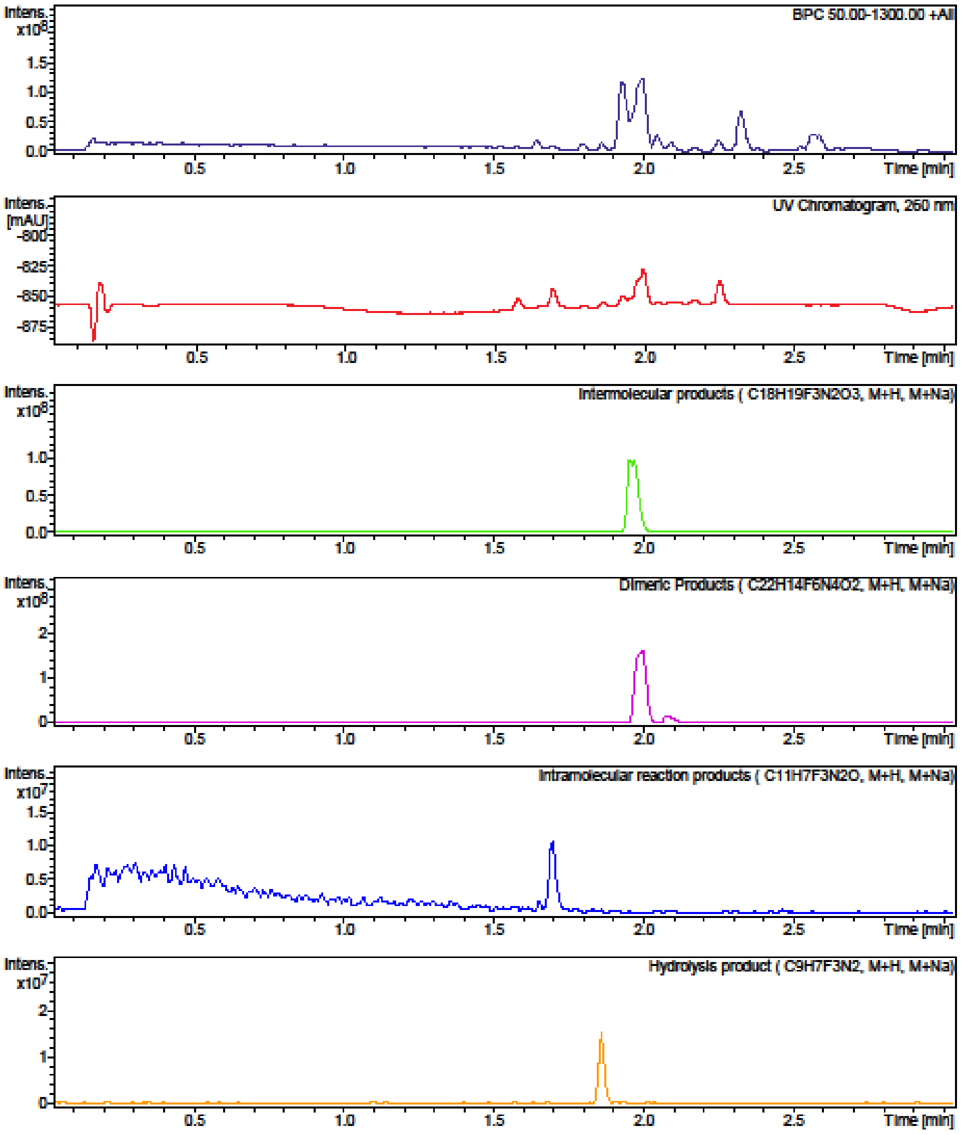
BPC 50.00-1300.00 +All
UV Chromatogram, 260 nm
Intermolecular products (C18H19F3N2O3, M+H, M+Na)
Dimeric Products (C22H14F6N4O2, M+H, M+Na)
Intramolecular reaction products (C11H7F3N2O, M+H, M+Na)
Hydrolysis product (C9H7F3N2, M+H, M+Na)
Intens.
x10^8
[mAU]
x10^7
Time [min]
0.5
1.0
1.5
2.0
2.5

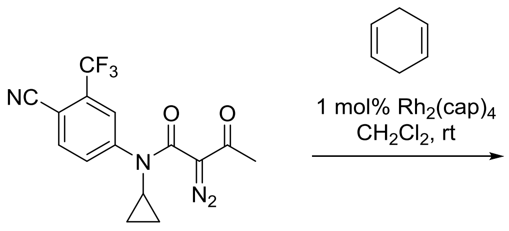
CF3
NC
O
O
N
N2
1 mol% Rh2(cap)4
CH2Cl2, rt

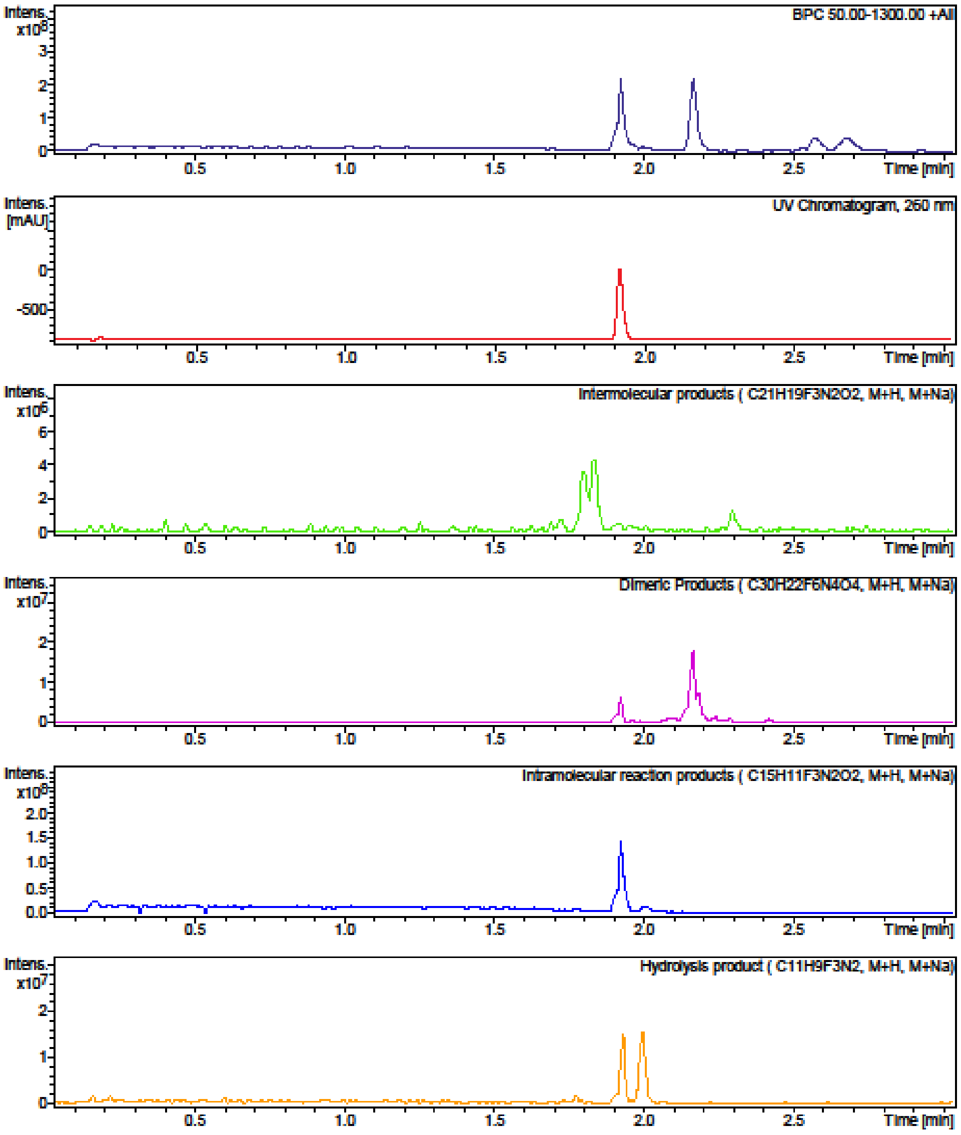
Intens. x10^8
BPC 50.00-1300.00 +All
Time [min]
Intens. [mAU]
UV Chromatogram, 260 nm
Intens. x10^6
Intermolecular products (C21H19F3N2O2, M+H, M+Na)
Intens. x10^7
Dimeric Products (C30H22F6N4O4, M+H, M+Na)
Intens. x10^6
Intramolecular reaction products (C15H11F3N2O2, M+H, M+Na)
Intens. x10^7
Hydrolysis product (C11H9F3N2, M+H, M+Na)

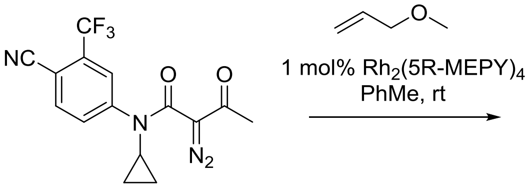
CF3
NC
O
O
N
N2
1 mol% Rh2(5R-MEPY)4
PhMe, rt

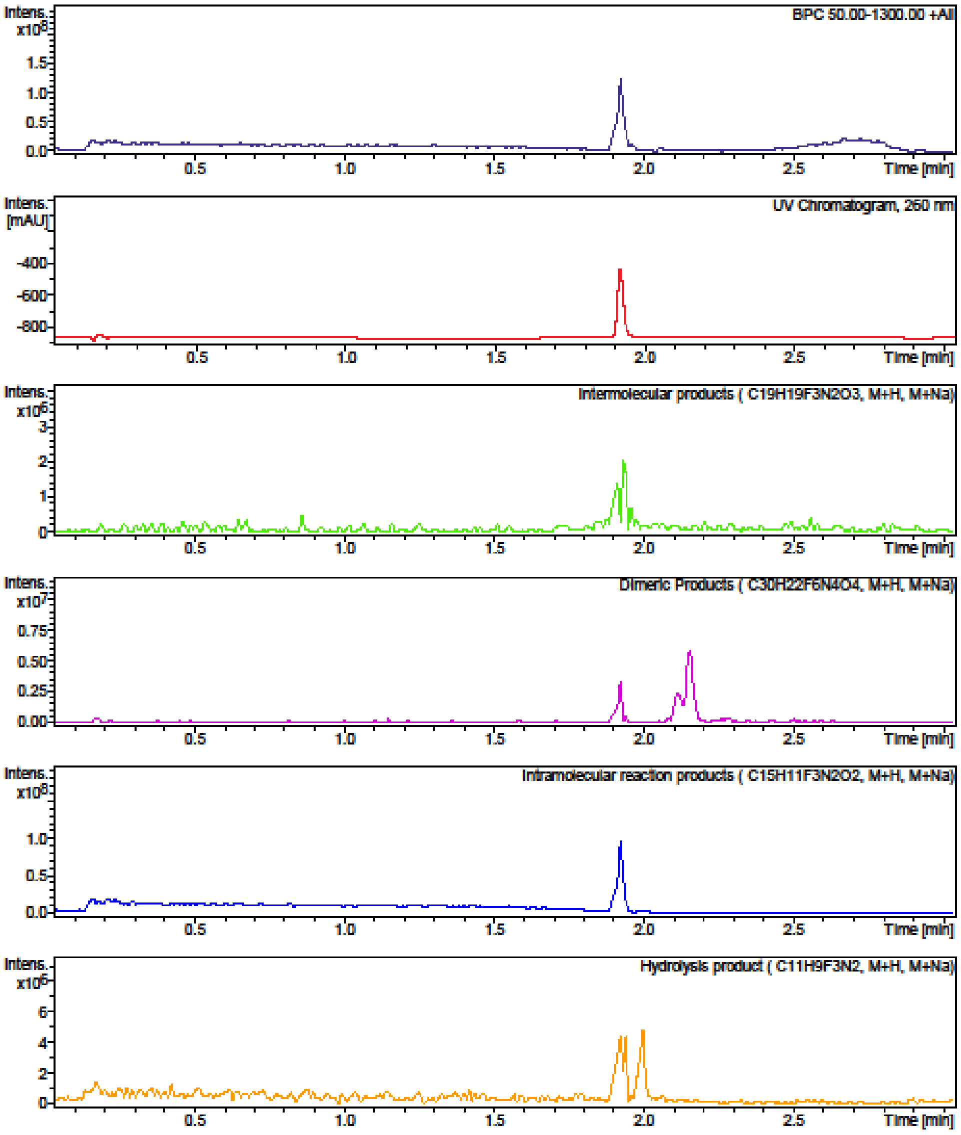
BPC 50.00-1300.00 +All
UV Chromatogram, 260 nm
Intermolecular products (C19H19F3N2O3, M+H, M+Na)
Dimeric Products (C30H22F6N4O4, M+H, M+Na)
Intramolecular reaction products (C15H11F3N2O2, M+H, M+Na)
Hydrolysis product (C11H9F3N2, M+H, M+Na)
Intens.
x10^8
[mAU]
x10^6
x10^7
Time [min]

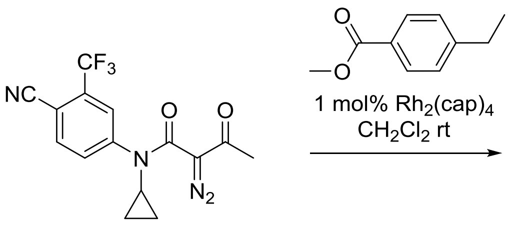
CF3
NC
O
O
N
N2
O
O
1 mol% Rh2(cap)4
CH2Cl2 rt

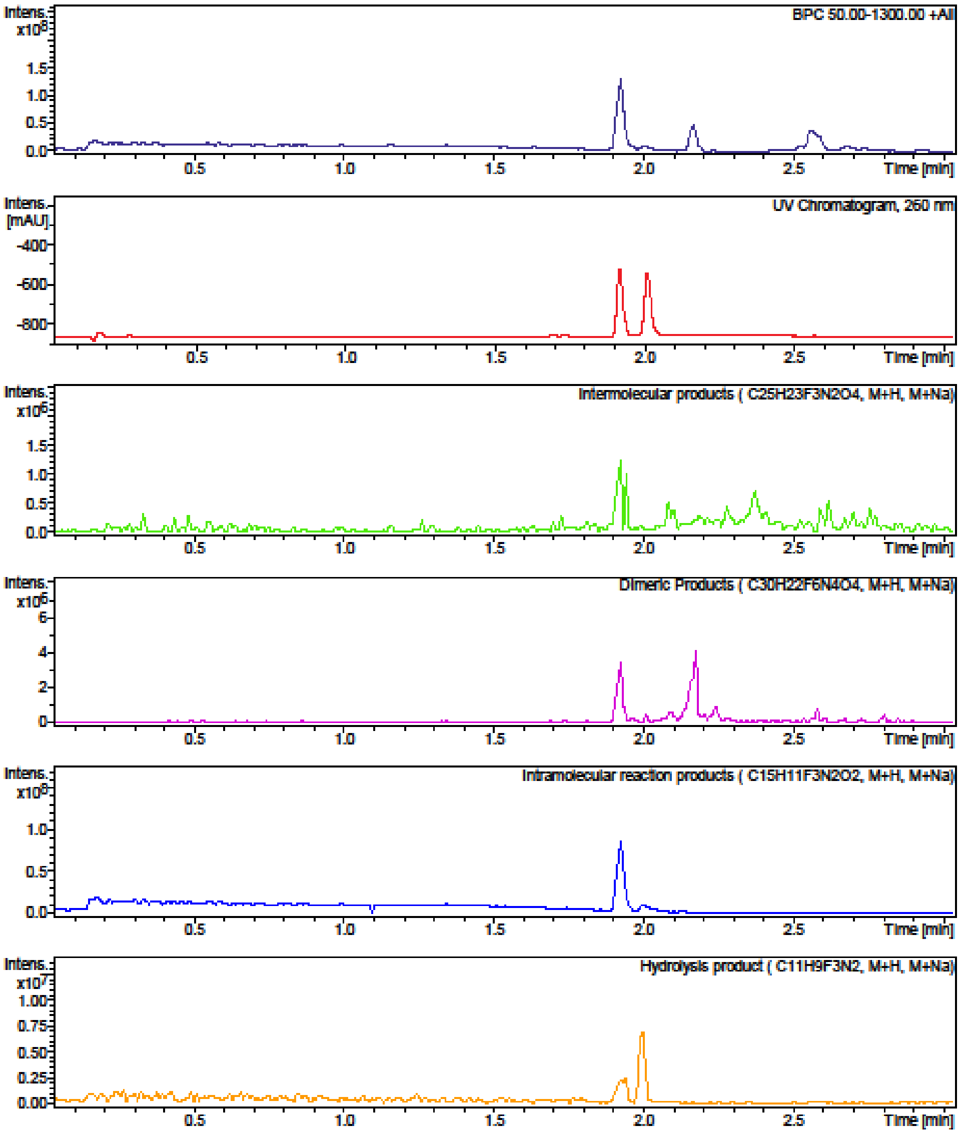
BPC 50.00-1300.00 +All
UV Chromatogram, 260 nm
Intermolecular products (C25H23F3N2O4, M+H, M+Na)
Dimeric Products (C30H22F6N4O4, M+H, M+Na)
Intramolecular reaction products (C15H11F3N2O2, M+H, M+Na)
Hydrolysis product (C11H9F3N2, M+H, M+Na)
Intens.
x10^8
Intens.
[mAU]
-400
-600
-800
Intens.
x10^6
Intens.
x10^6
Intens.
x10^8
Intens.
x10^7
Time [min]

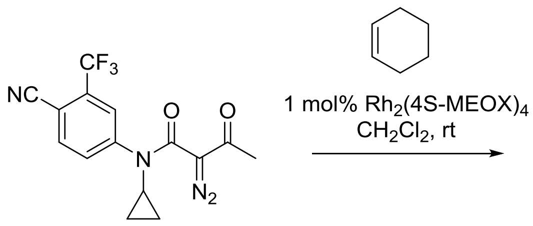
CF3
NC
O
O
N
N2
1 mol% Rh2(4S-MEOX)4
CH2Cl2, rt

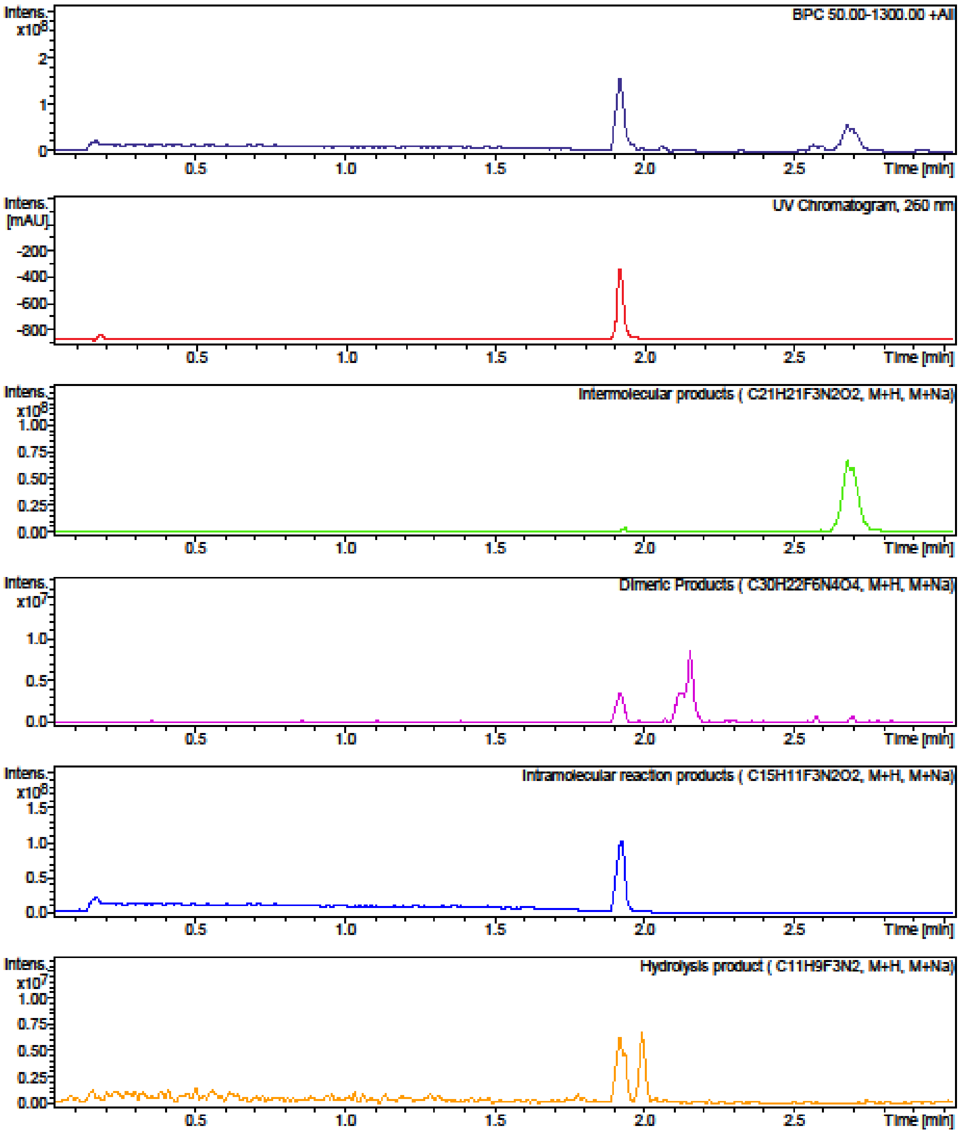
BPC 50.00-1300.00 +All
UV Chromatogram, 260 nm
Intermolecular products (C21H21F3N2O2, M+H, M+Na)
Dimeric Products (C30H22F6N4O4, M+H, M+Na)
Intramolecular reaction products (C15H11F3N2O2, M+H, M+Na)
Hydrolysis product (C11H9F3N2, M+H, M+Na)
Intens.
Time [min]

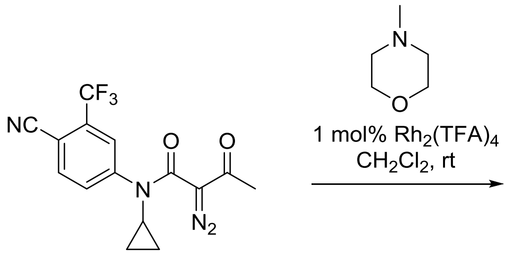
NC
CF3
O
O
N
N2
N
O
1 mol% Rh2(TFA)4
CH2Cl2, rt

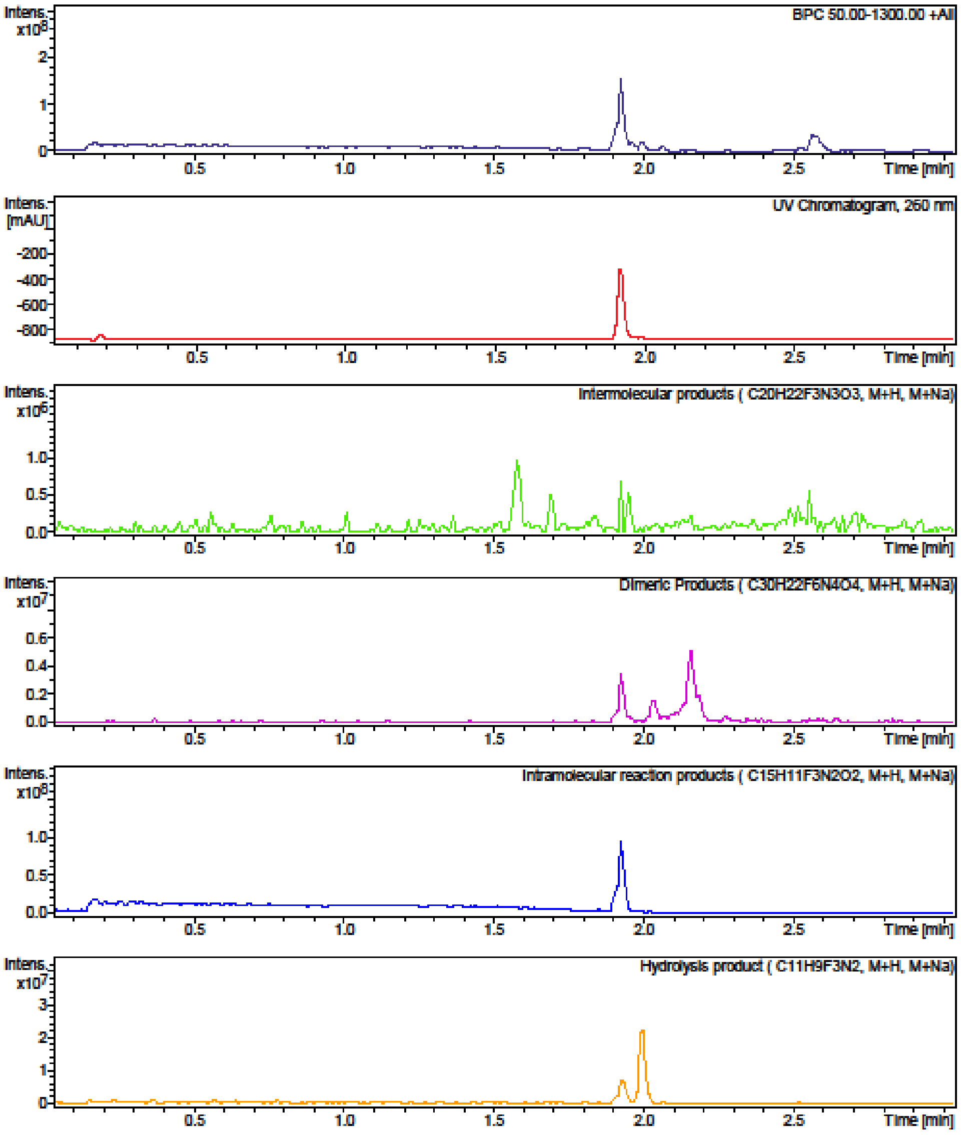
BPC 50.00-1300.00 +All
UV Chromatogram, 260 nm
Intermolecular products (C20H22F3N3O3, M+H, M+Na)
Dimeric Products (C30H22F6N4O4, M+H, M+Na)
Intramolecular reaction products (C15H11F3N2O2, M+H, M+Na)
Hydrolysis product (C11H9F3N2, M+H, M+Na)
Intens.
Time [min]

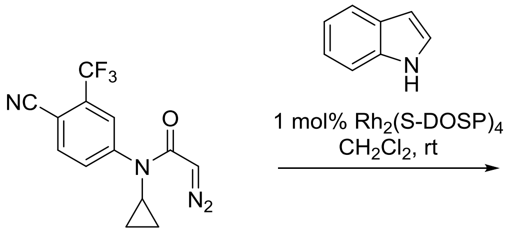
CF3
NC
O
N
N2
N
H
1 mol% Rh2(S-DOSP)4
CH2Cl2, rt

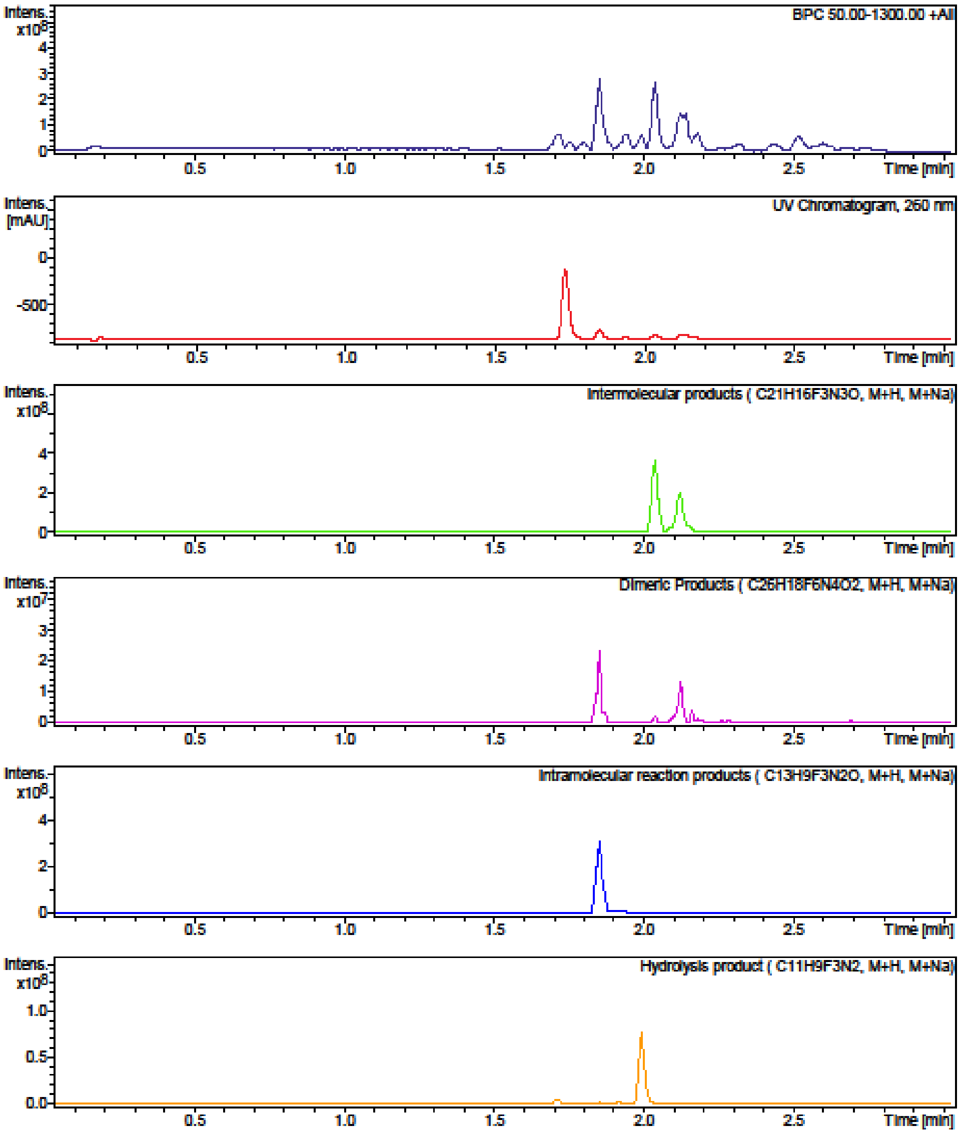
BPC 50.00-1300.00 +All
UV Chromatogram, 260 nm
Intermolecular products (C21H16F3N3O, M+H, M+Na)
Dimeric Products (C26H18F6N4O2, M+H, M+Na)
Intramolecular reaction products (C13H9F3N2O, M+H, M+Na)
Hydrolysis product (C11H9F3N2, M+H, M+Na)
Intens.
Time [min]

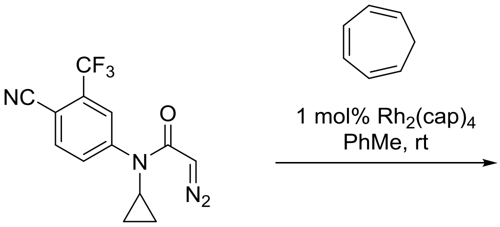
CF3
NC
O
N
N2
1 mol% Rh2(cap)4
PhMe, rt

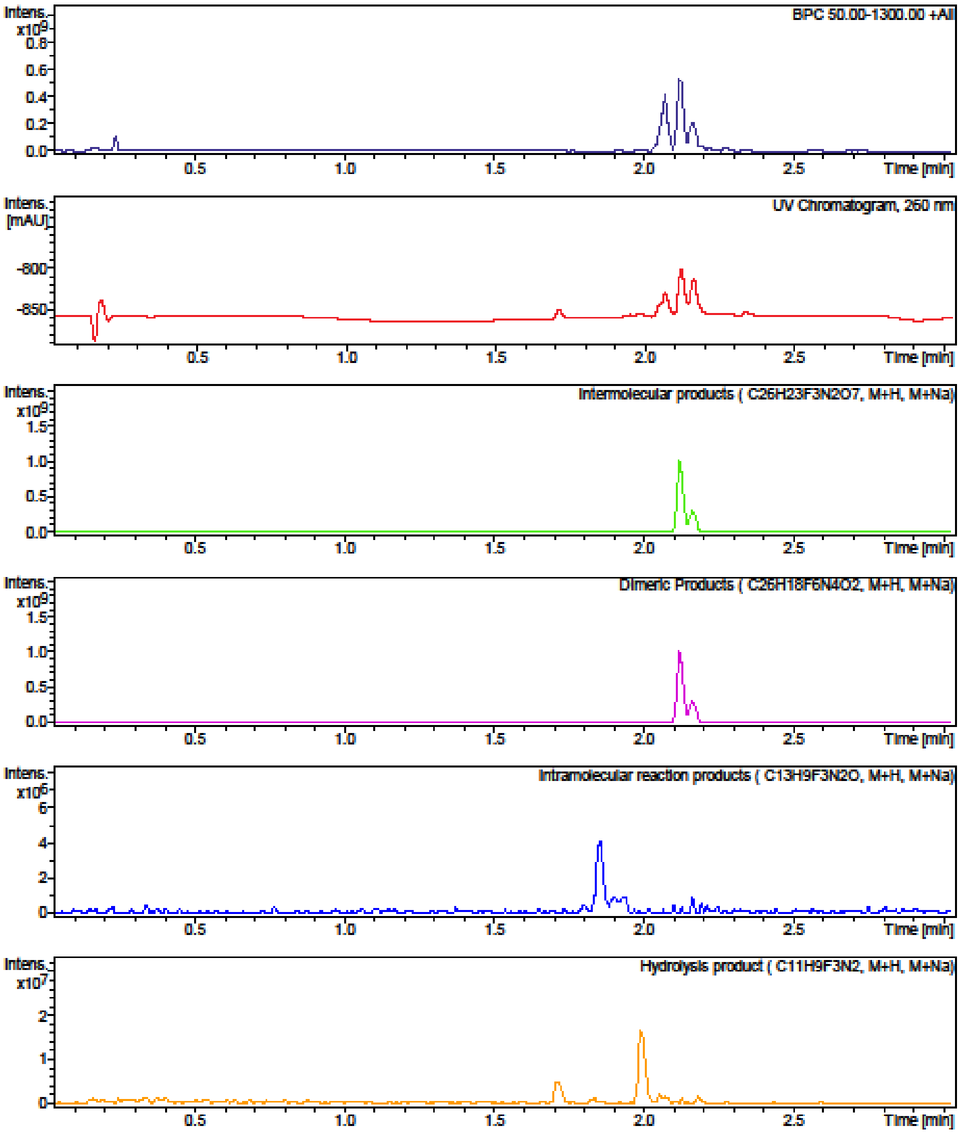
Intens.
x10^9
BPC 50.00-1300.00 +All
Time [min]
Intens.
[mAU]
UV Chromatogram, 260 nm
Time [min]
Intens.
x10^9
Intermolecular products (C26H23F3N2O7, M+H, M+Na)
Time [min]
Intens.
x10^9
Dimeric Products (C26H18F6N4O2, M+H, M+Na)
Time [min]
Intens.
x10^6
Intramolecular reaction products (C13H9F3N2O, M+H, M+Na)
Time [min]
Intens.
x10^7
Hydrolysis product (C11H9F3N2, M+H, M+Na)
Time [min]

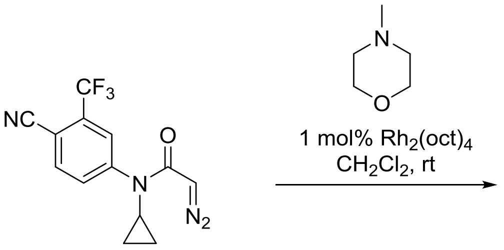
NC
CF3
O
N
N2
N
O
1 mol% Rh2(oct)4
CH2Cl2, rt

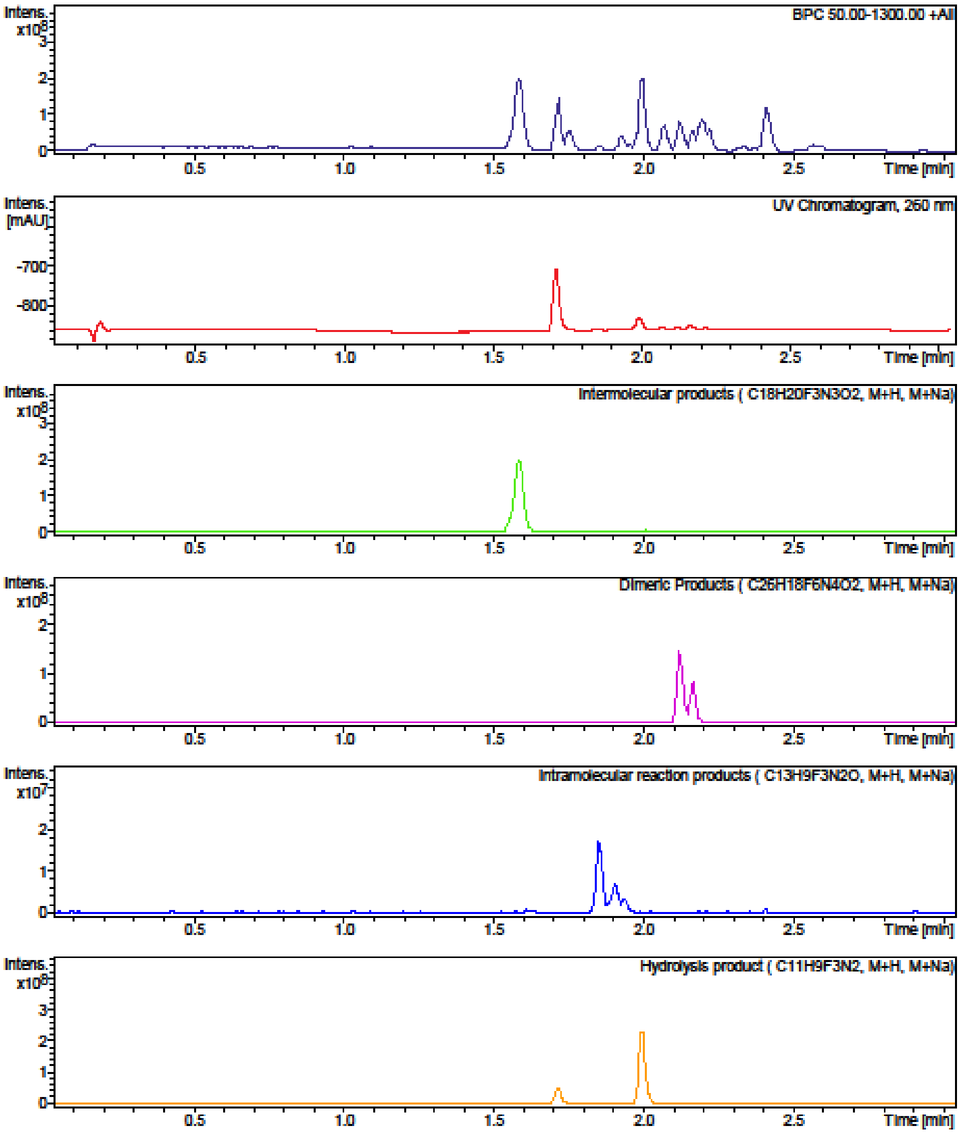
Intens. x10^8
BPC 50.00-1300.00 +All
Time [min]
Intens. [mAU]
UV Chromatogram, 260 nm
Intermolecular products (C18H20F3N3O2, M+H, M+Na)
Dimeric Products (C26H18F6N4O2, M+H, M+Na)
Intens. x10^7
Intramolecular reaction products (C13H9F3N2O, M+H, M+Na)
Hydrolysis product (C11H9F3N2, M+H, M+Na)

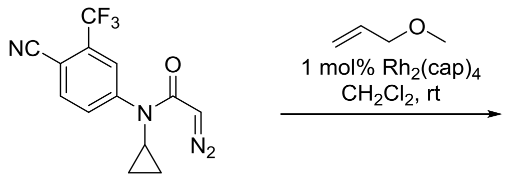
CF3
NC
O
N
N2
O
1 mol% Rh2(cap)4
CH2Cl2, rt

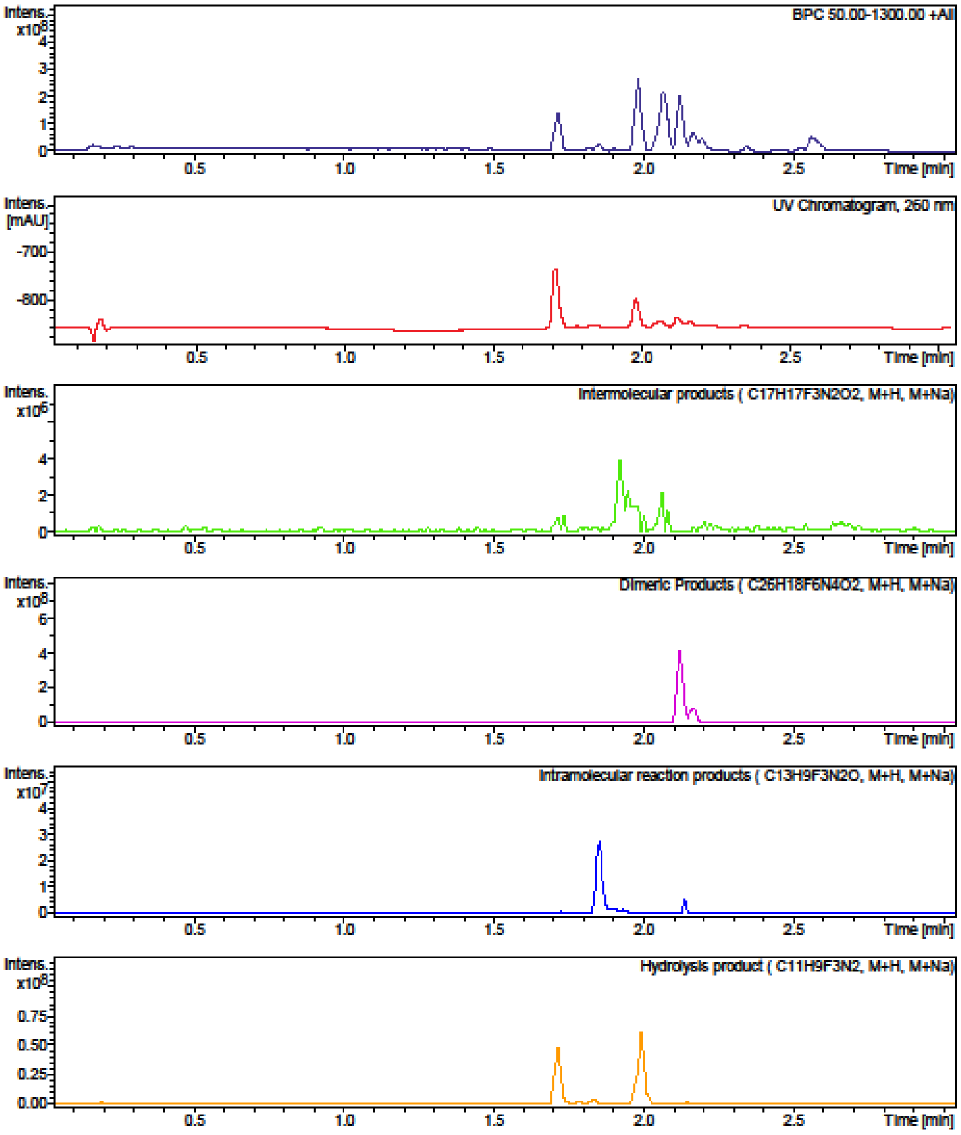
BPC 50.00-1300.00 +All
UV Chromatogram, 260 nm
Intermolecular products (C17H17F3N2O2, M+H, M+Na)
Dimeric Products (C26H18F6N4O2, M+H, M+Na)
Intramolecular reaction products (C13H9F3N2O, M+H, M+Na)
Hydrolysis product (C11H9F3N2, M+H, M+Na)
Intens.
Time [min]

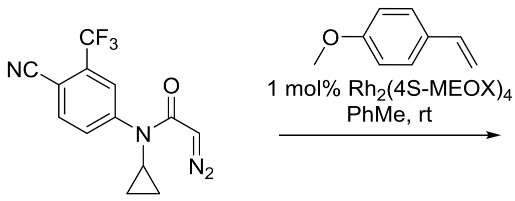
CF3
NC
O
N
N2
O
1 mol% Rh2(4S-MEOX)4
PhMe, rt

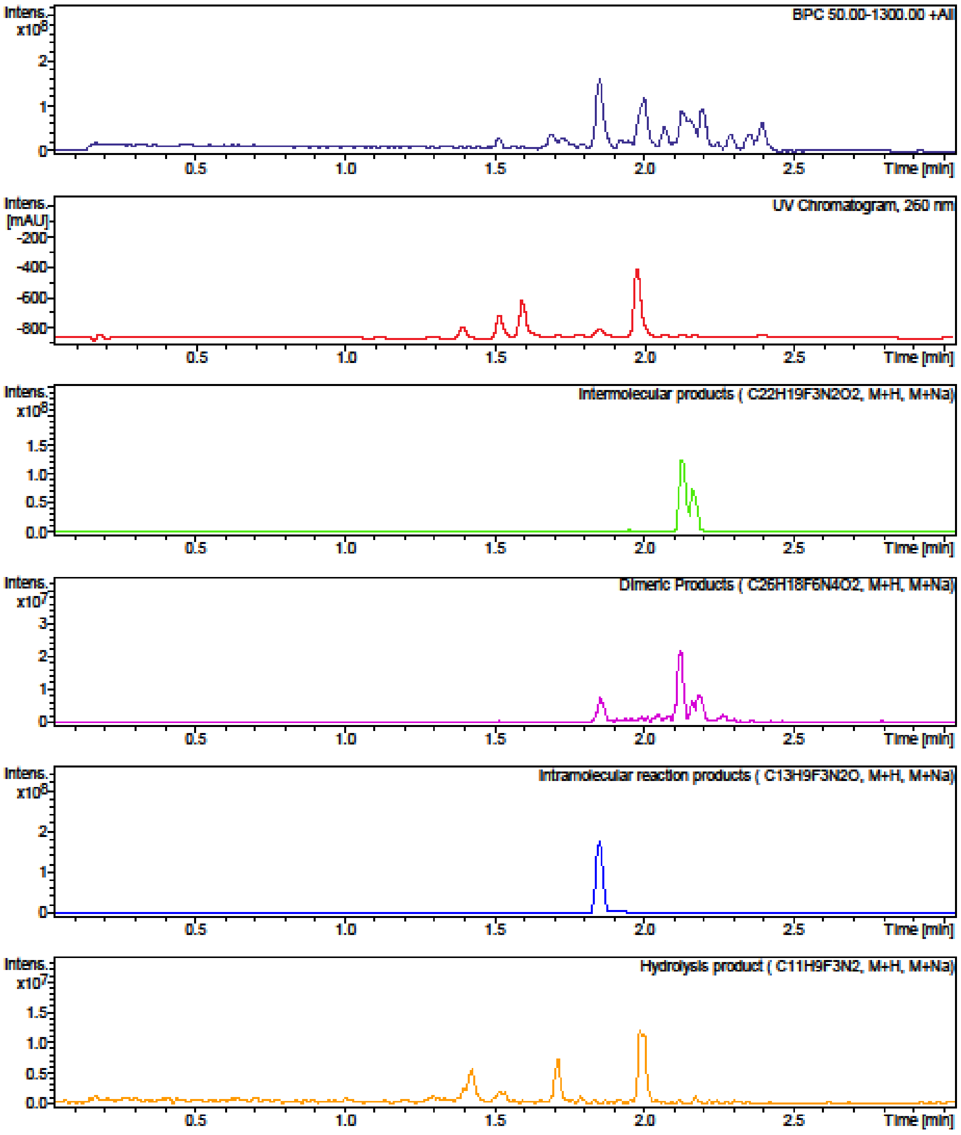
BPC 50.00-1300.00 +All
UV Chromatogram, 260 nm
Intermolecular products (C22H19F3N2O2, M+H, M+Na)
Dimeric Products (C26H18F6N4O2, M+H, M+Na)
Intramolecular reaction products (C13H9F3N2O, M+H, M+Na)
Hydrolysis product (C11H9F3N2, M+H, M+Na)
Intens.
x10^8
[mAU]
x10^7
Time [min]

11 NMR Spectra

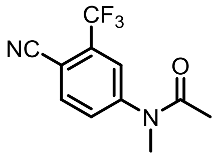

1

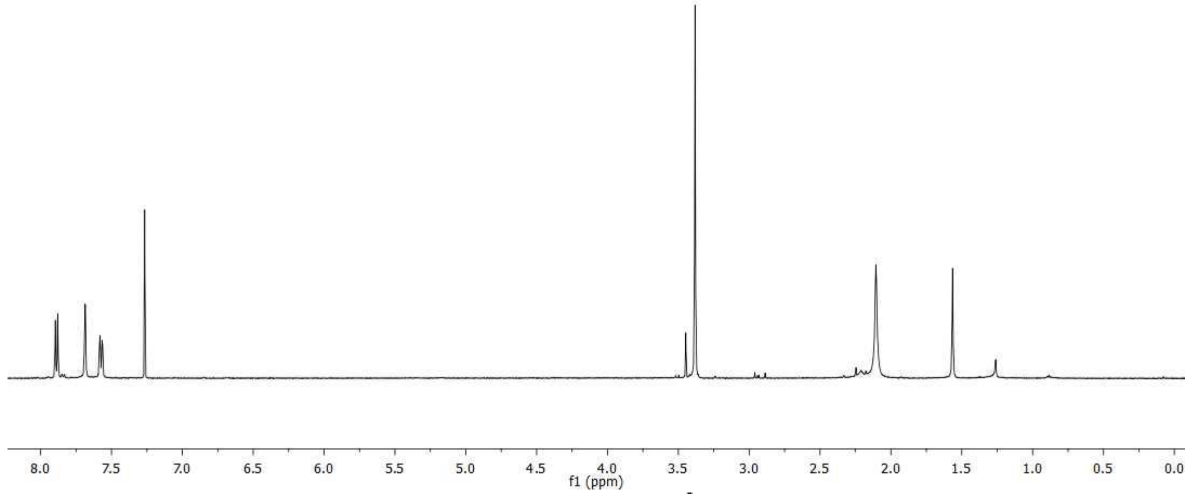

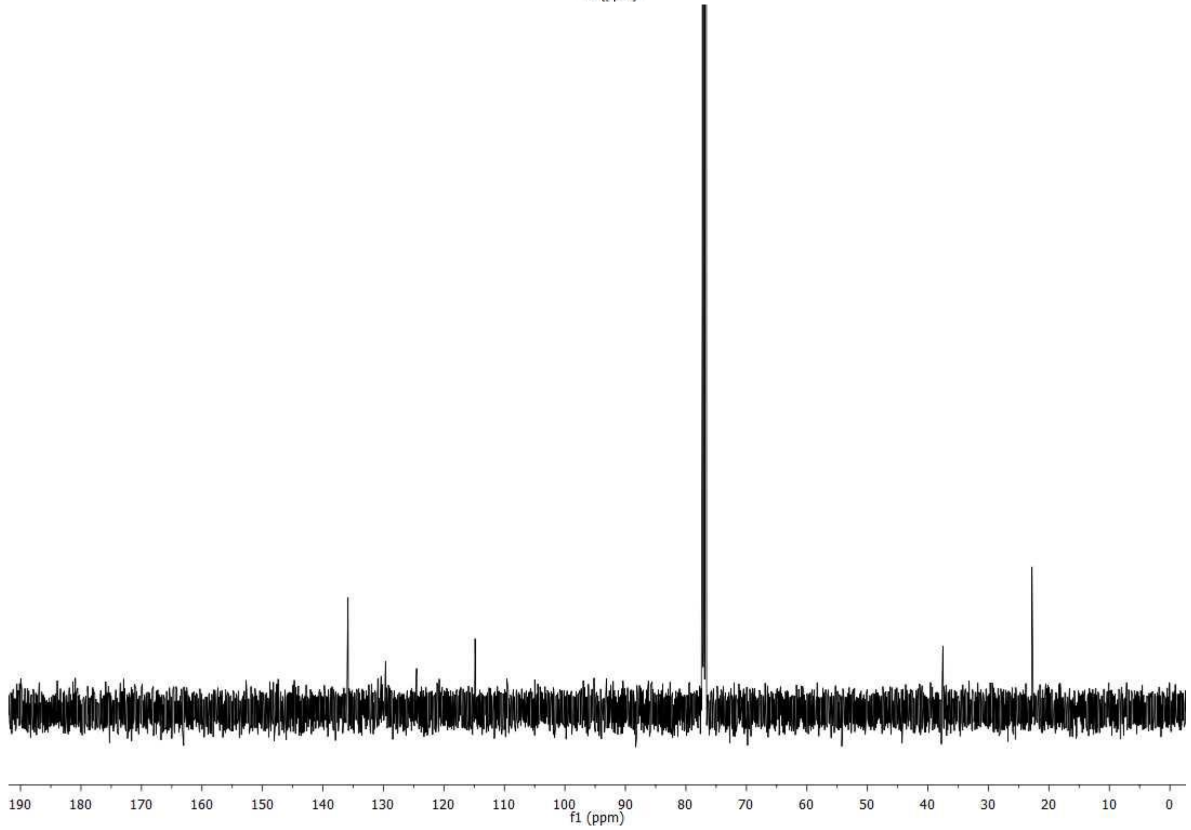

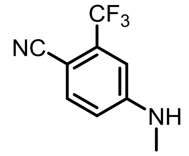
CF$_3$
NC
NH

15

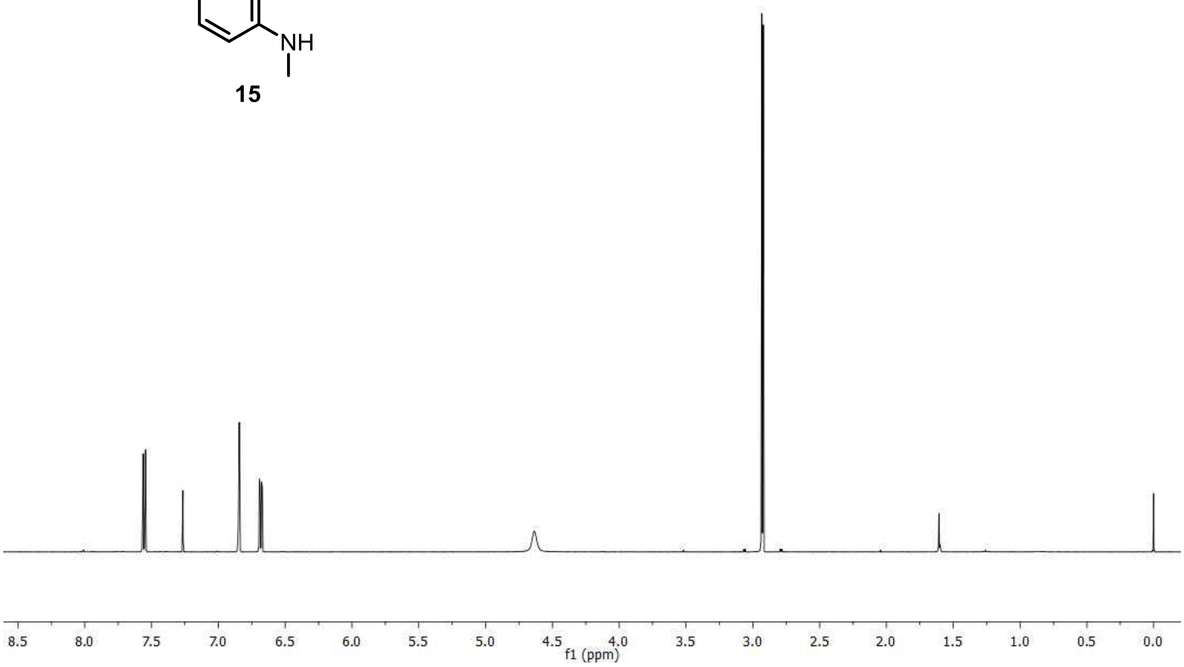
8.5 8.0 7.5 7.0 6.5 6.0 5.5 5.0 4.5 4.0 3.5 3.0 2.5 2.0 1.5 1.0 0.5 0.0
f1 (ppm)

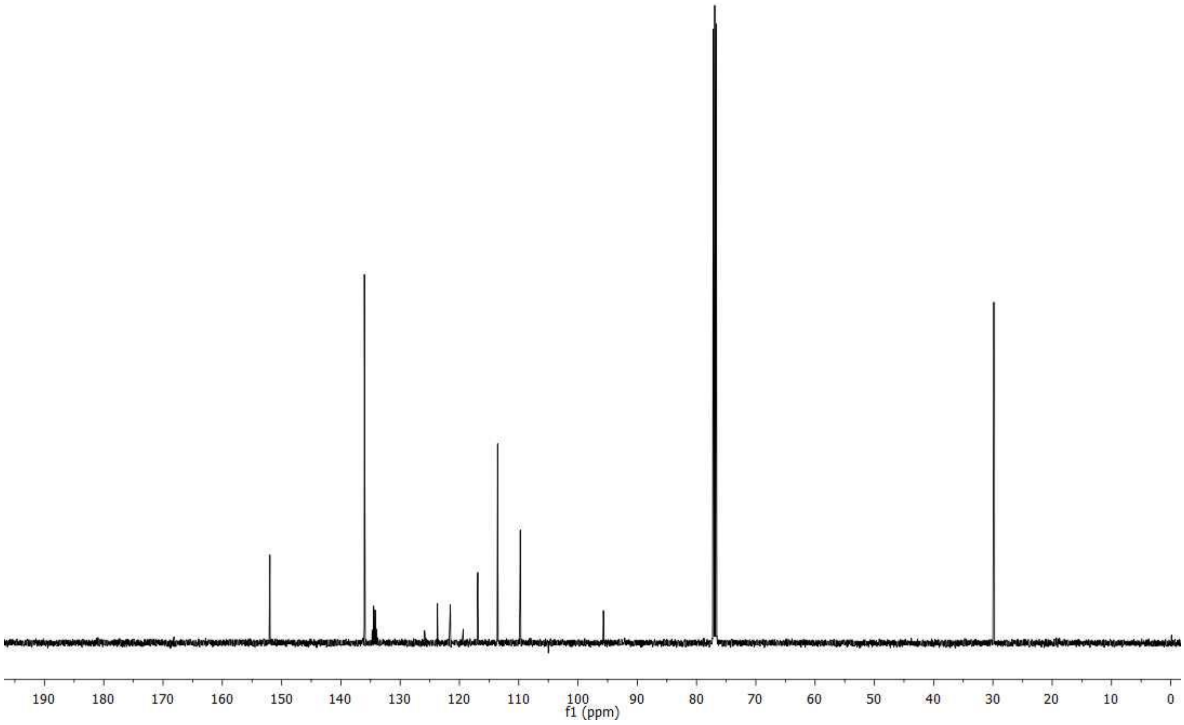
190 180 170 160 150 140 130 120 110 100 90 80 70 60 50 40 30 20 10 0
f1 (ppm)

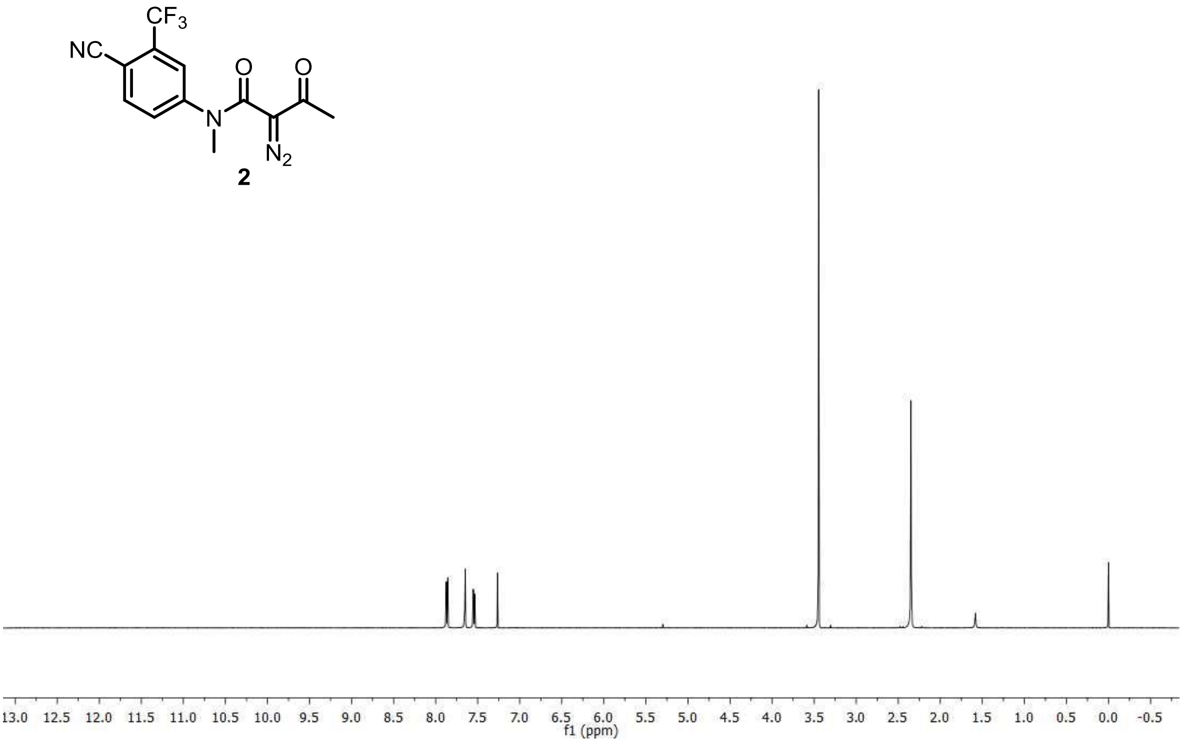
CF3
NC
O
O
N
N2

2

13.0 12.5 12.0 11.5 11.0 10.5 10.0 9.5 9.0 8.5 8.0 7.5 7.0 6.5 6.0 5.5 5.0 4.5 4.0 3.5 3.0 2.5 2.0 1.5 1.0 0.5 0.0 -0.5
f1 (ppm)

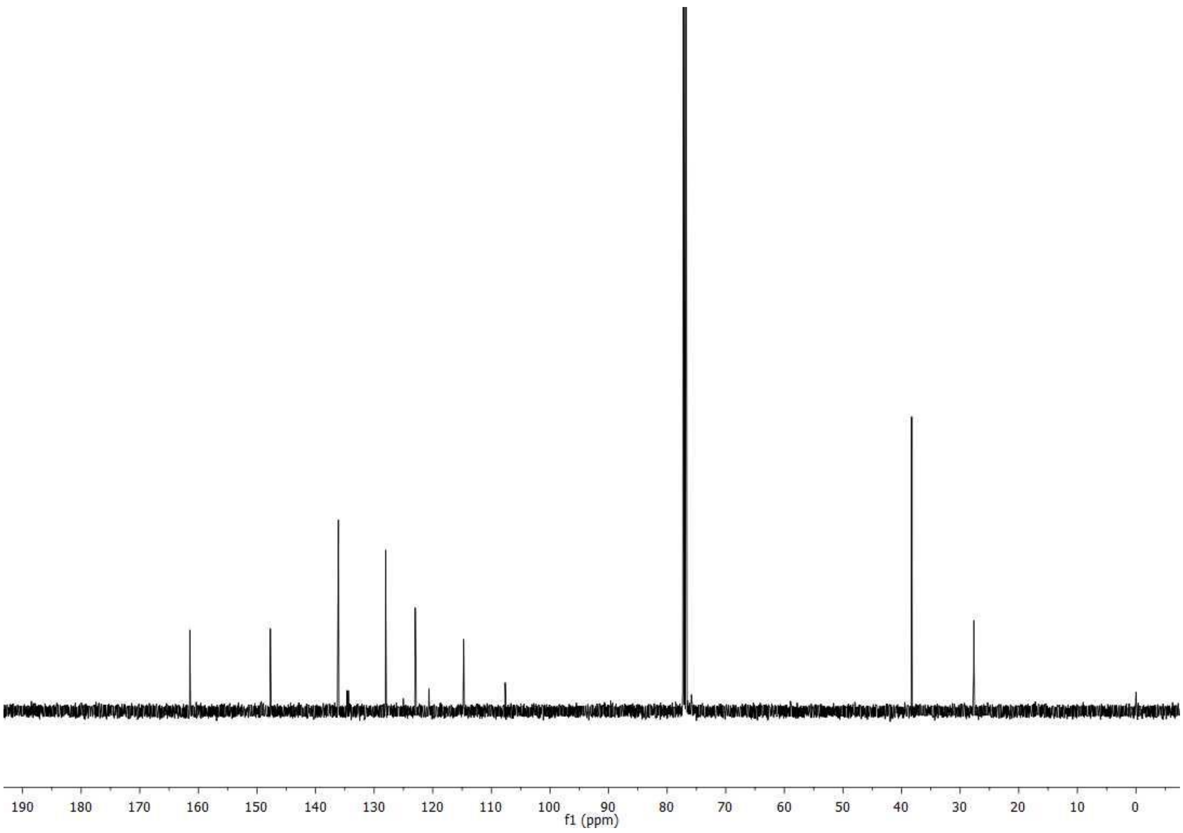
190 180 170 160 150 140 130 120 110 100 90 80 70 60 50 40 30 20 10 0
f1 (ppm)

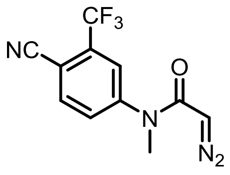
CF3
NC
O
N
N2

3

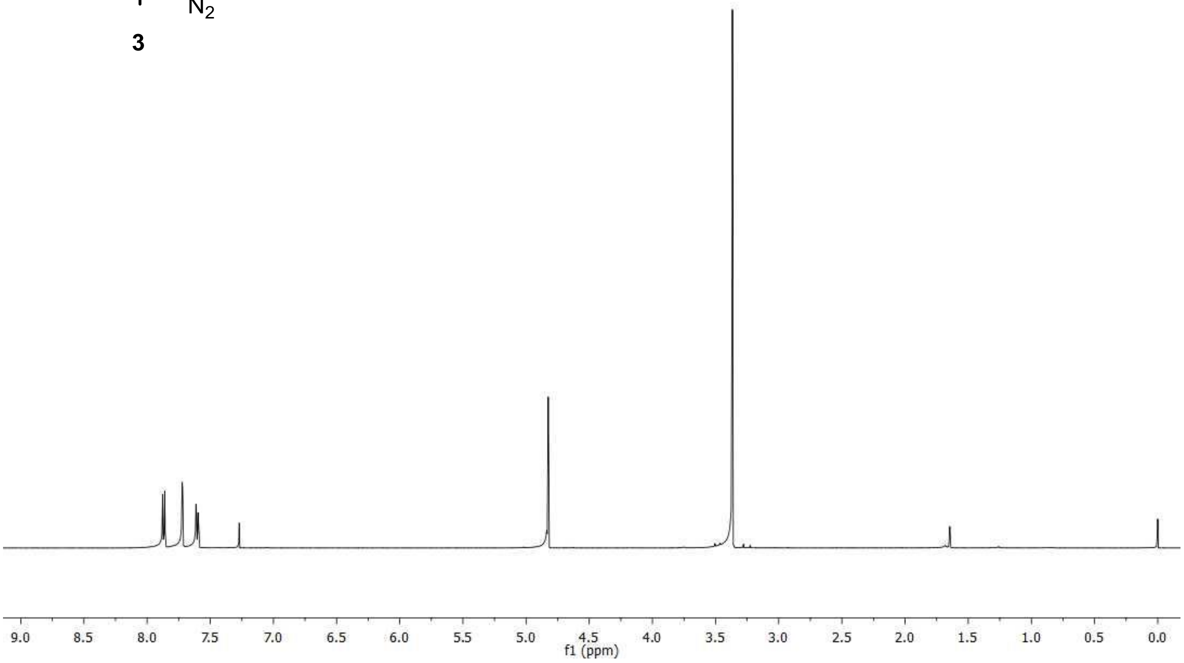
9.0 8.5 8.0 7.5 7.0 6.5 6.0 5.5 5.0 4.5 4.0 3.5 3.0 2.5 2.0 1.5 1.0 0.5 0.0
f1 (ppm)

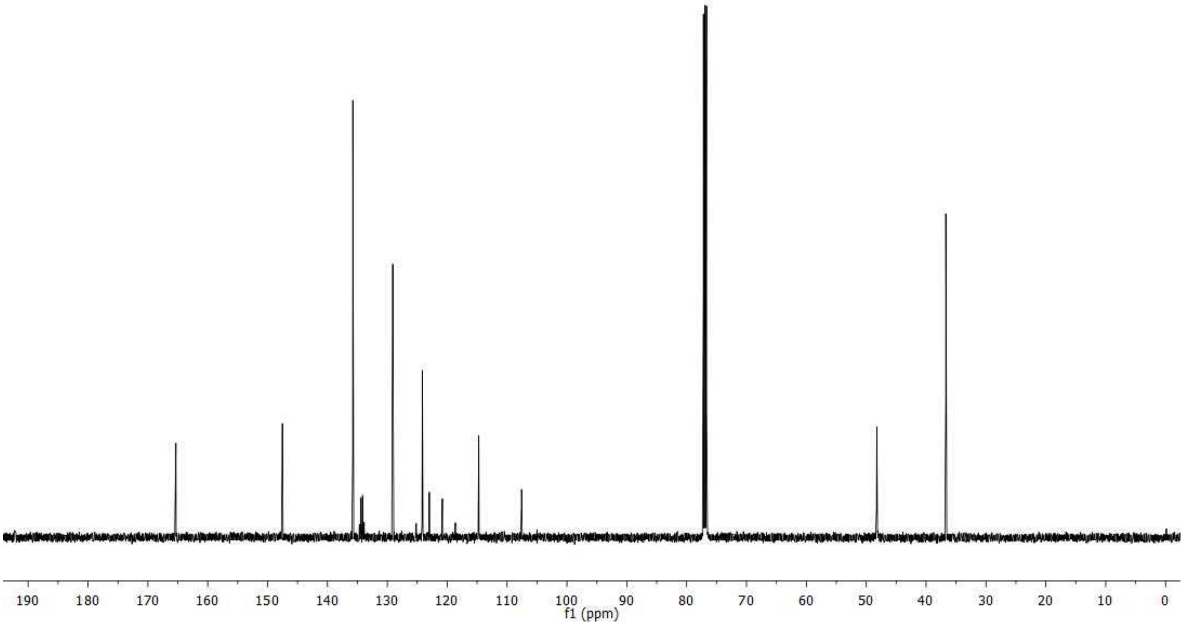
190 180 170 160 150 140 130 120 110 100 90 80 70 60 50 40 30 20 10 0
f1 (ppm)

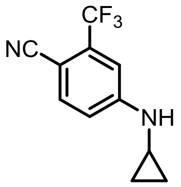
CF3
NC
NH

16

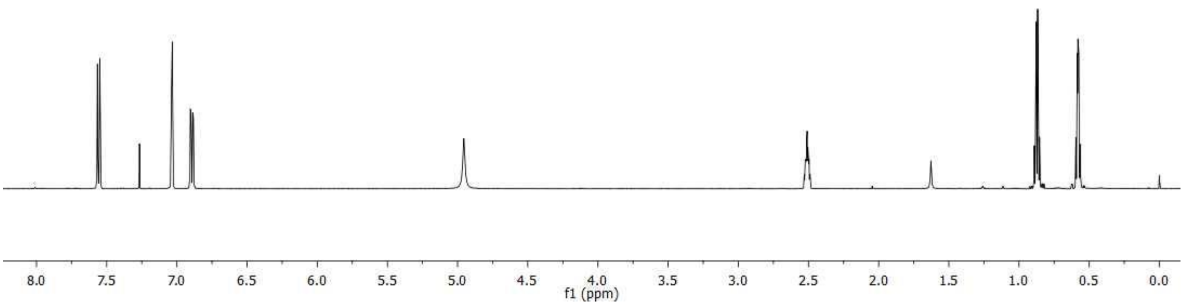
8.0 7.5 7.0 6.5 6.0 5.5 5.0 4.5 4.0 3.5 3.0 2.5 2.0 1.5 1.0 0.5 0.0
f1 (ppm)

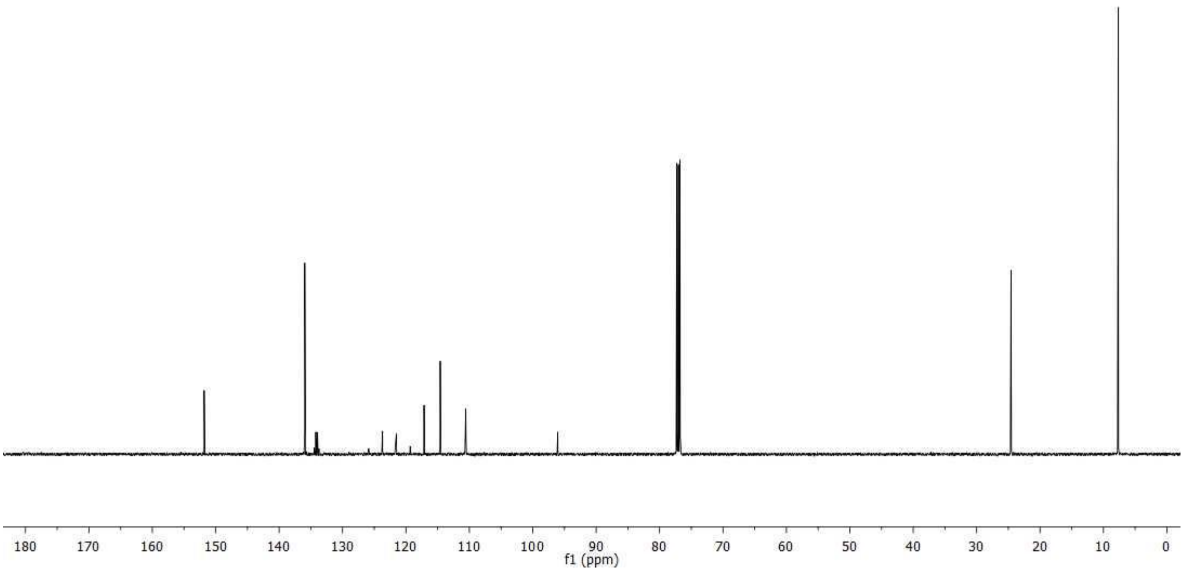
180 170 160 150 140 130 120 110 100 90 80 70 60 50 40 30 20 10 0
f1 (ppm)

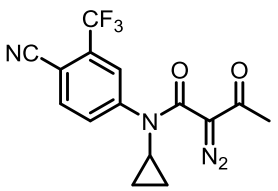
CF3
NC
O
O
N
N2

4

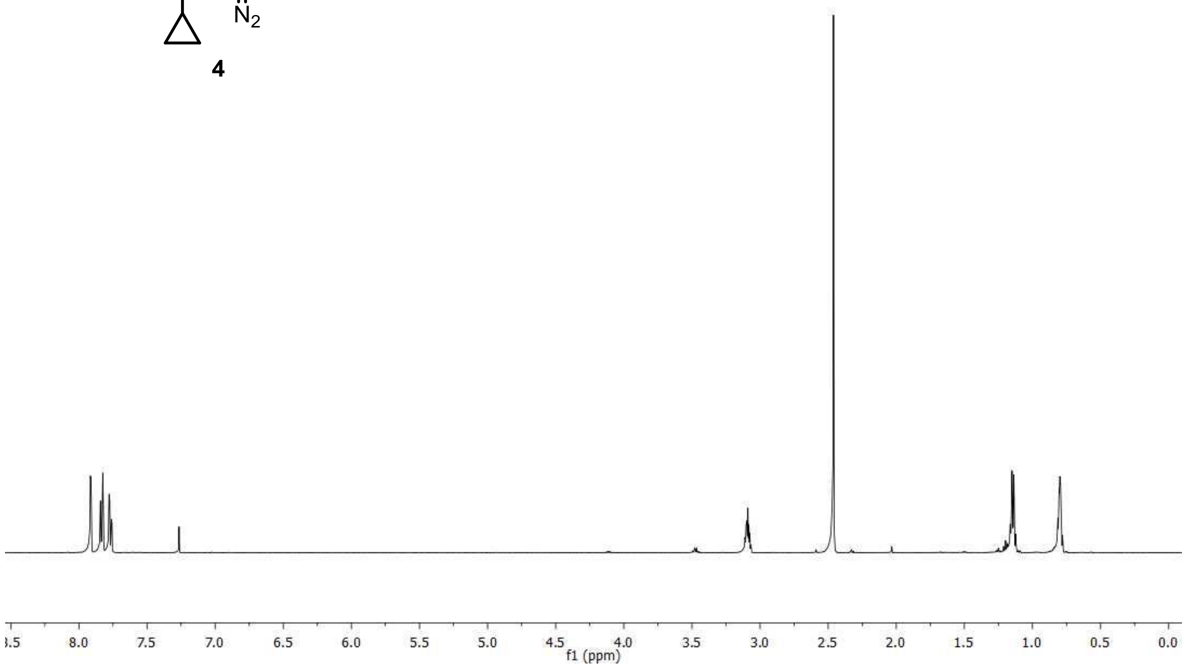
8.0 7.5 7.0 6.5 6.0 5.5 5.0 4.5 4.0 3.5 3.0 2.5 2.0 1.5 1.0 0.5 0.0
f1 (ppm)

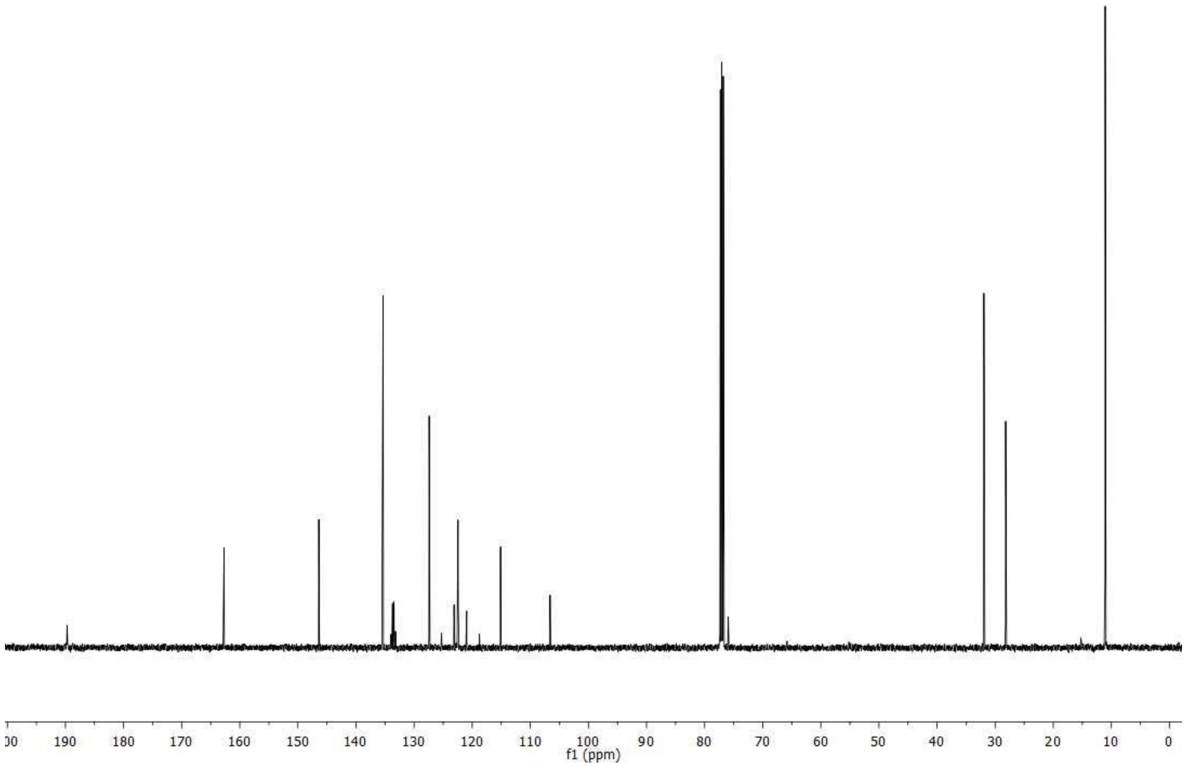
190 180 170 160 150 140 130 120 110 100 90 80 70 60 50 40 30 20 10 0
f1 (ppm)

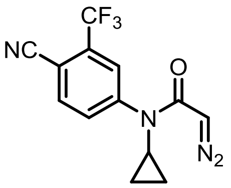
CF3
NC
O
N
N2

5

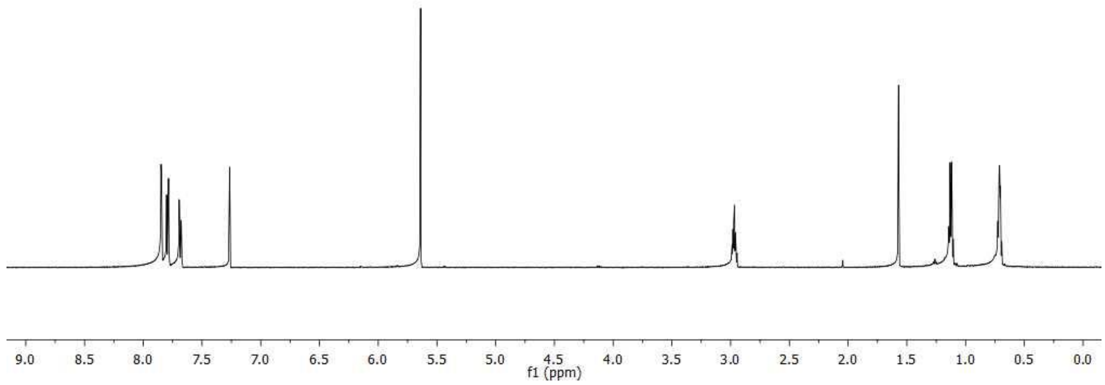
9.0 8.5 8.0 7.5 7.0 6.5 6.0 5.5 5.0 4.5 4.0 3.5 3.0 2.5 2.0 1.5 1.0 0.5 0.0
f1 (ppm)

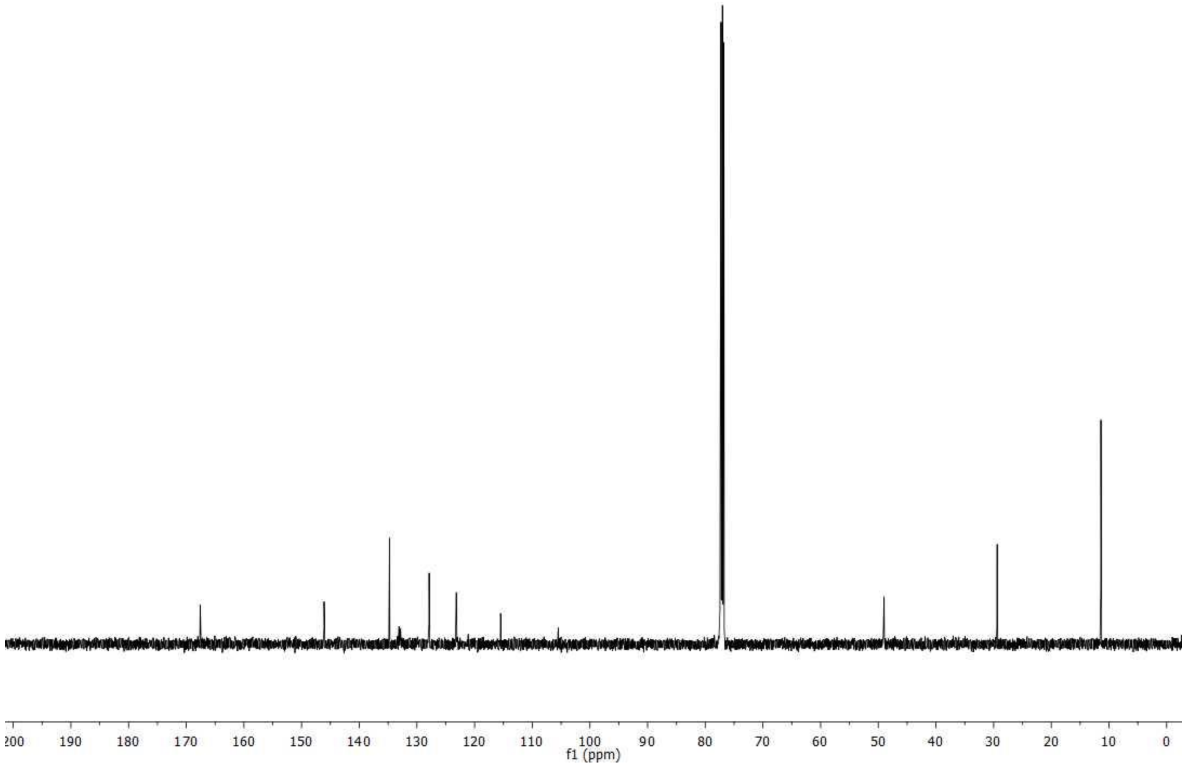
200 190 180 170 160 150 140 130 120 110 100 90 80 70 60 50 40 30 20 10 0
f1 (ppm)

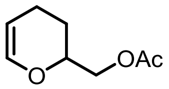
OAc
O

19

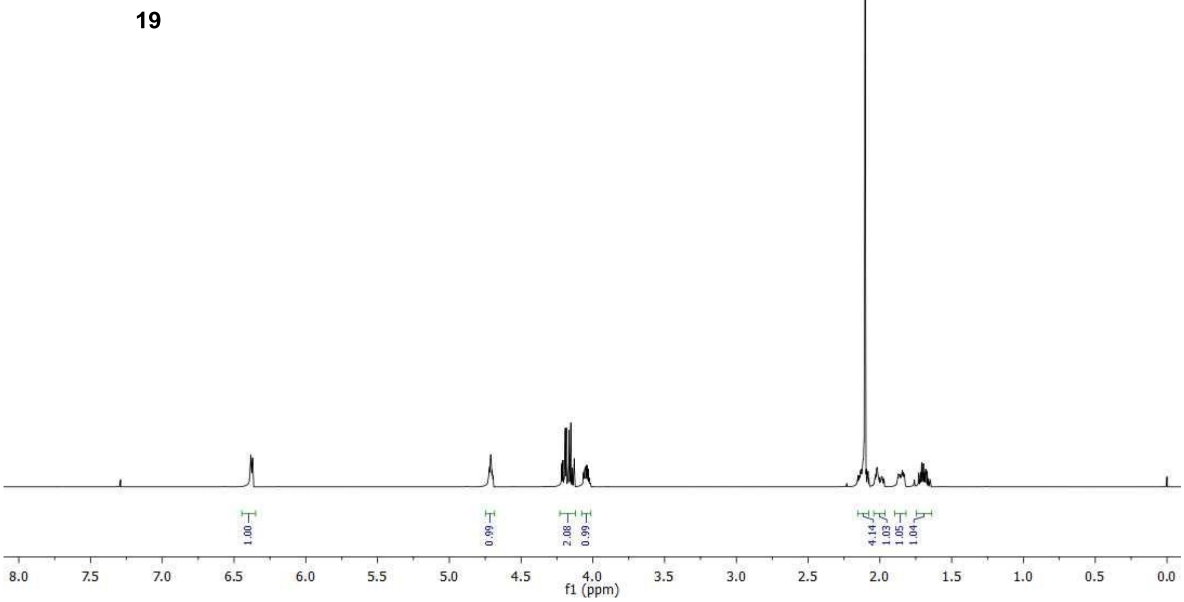
1.00
0.99
2.08
0.99
4.14
1.03
1.05
1.04
8.0 7.5 7.0 6.5 6.0 5.5 5.0 4.5 4.0 3.5 3.0 2.5 2.0 1.5 1.0 0.5 0.0
f1 (ppm)

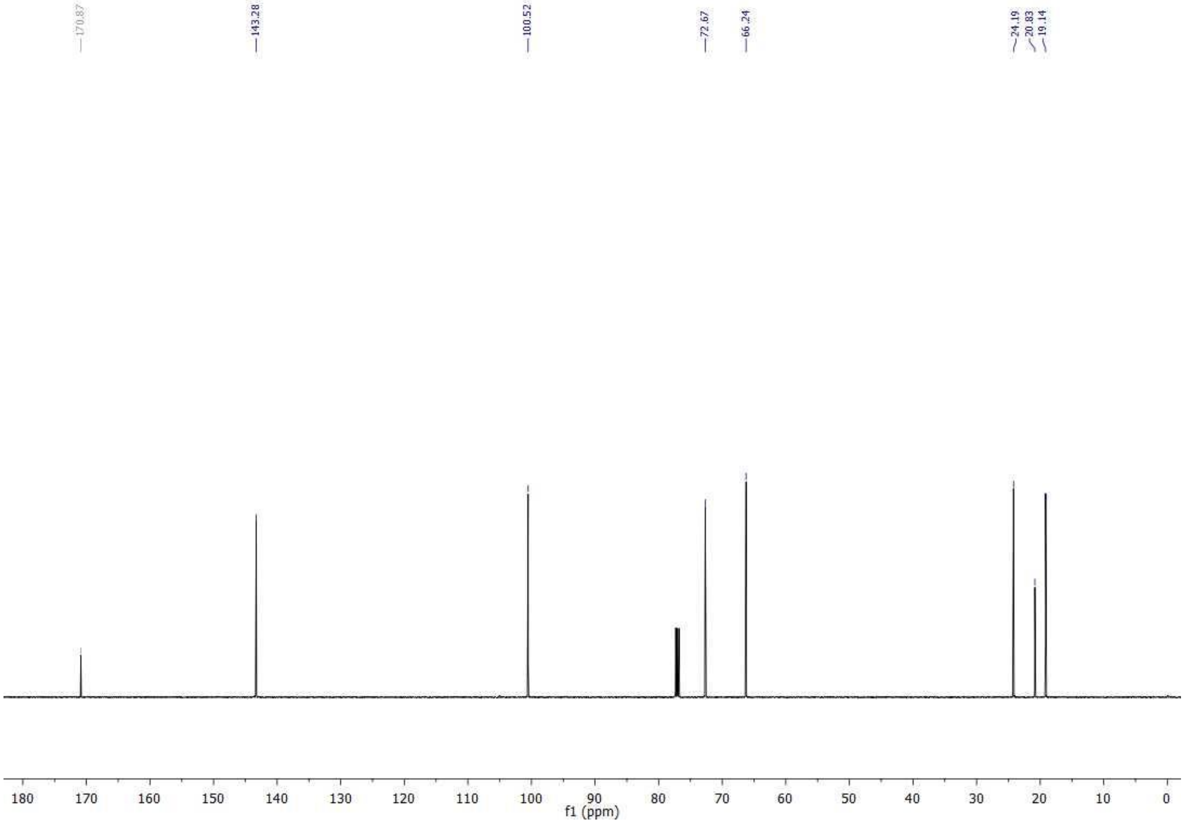
170.87
143.28
100.52
72.67
66.24
24.19
20.83
19.14
180 170 160 150 140 130 120 110 100 90 80 70 60 50 40 30 20 10 0
f1 (ppm)

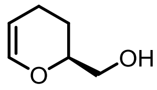
OH
O

***S*-6f'**

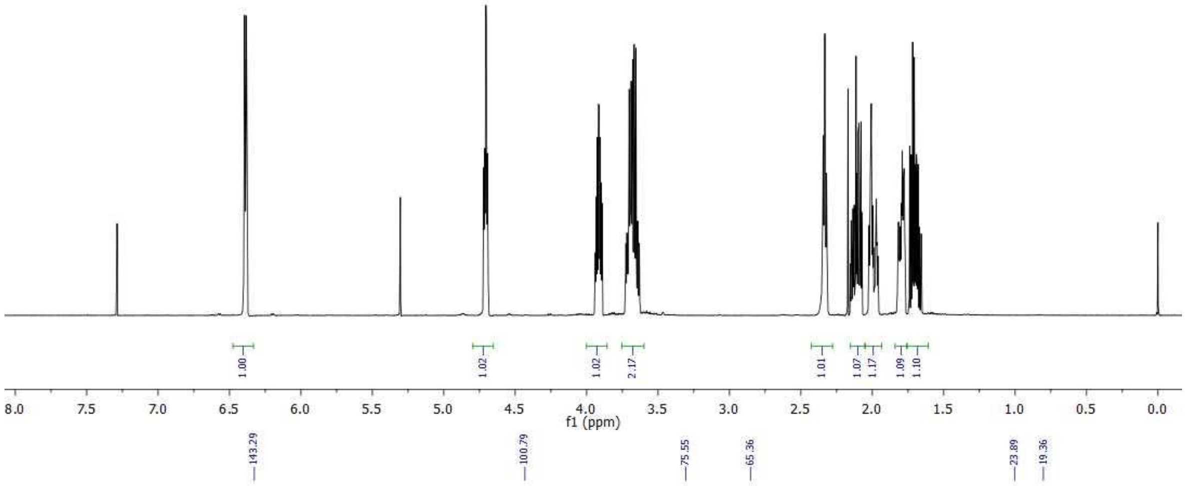
1.00
1.02
1.02
2.17
1.01
1.07
1.17
1.09
1.10
8.0 7.5 7.0 6.5 6.0 5.5 5.0 4.5 4.0 3.5 3.0 2.5 2.0 1.5 1.0 0.5 0.0
f1 (ppm)
143.29
100.79
75.55
65.36
23.89
19.36

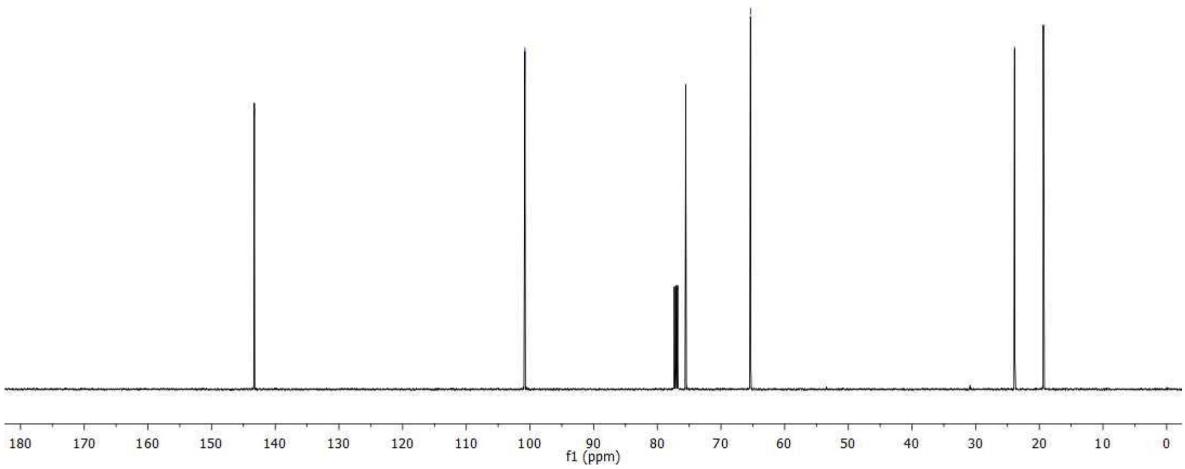
180 170 160 150 140 130 120 110 100 90 80 70 60 50 40 30 20 10 0
f1 (ppm)

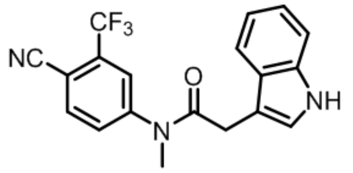
CF3
NC
O
NH
N

7

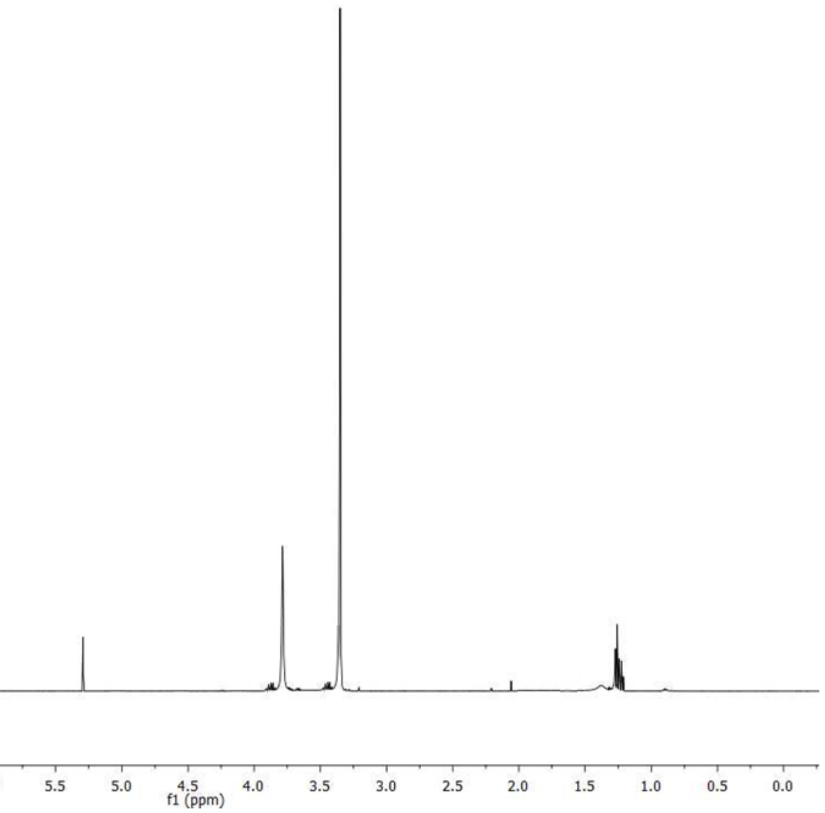
9.0 8.5 8.0 7.5 7.0 6.5 6.0 5.5 5.0 4.5 4.0 3.5 3.0 2.5 2.0 1.5 1.0 0.5 0.0
f1 (ppm)

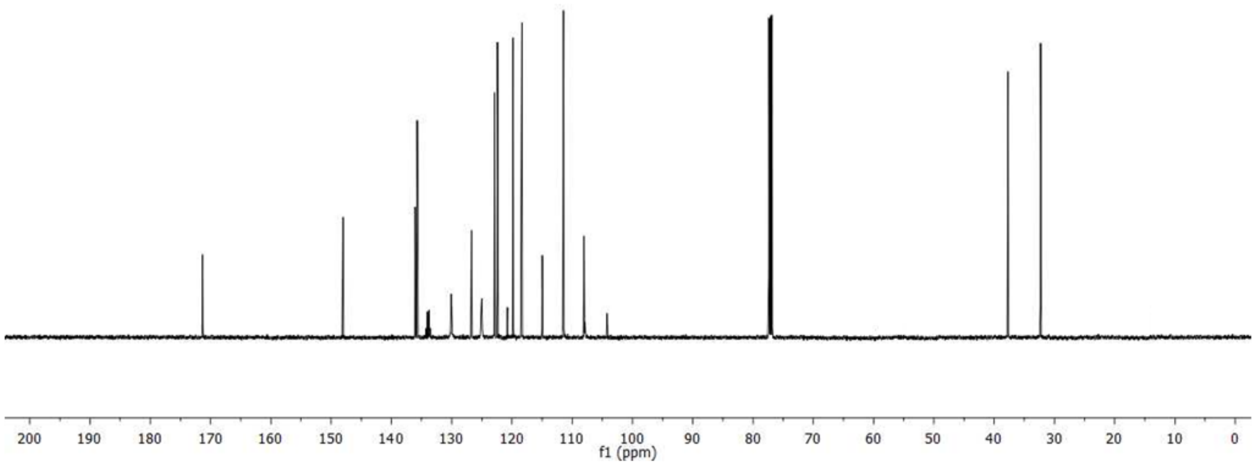
200 190 180 170 160 150 140 130 120 110 100 90 80 70 60 50 40 30 20 10 0
f1 (ppm)

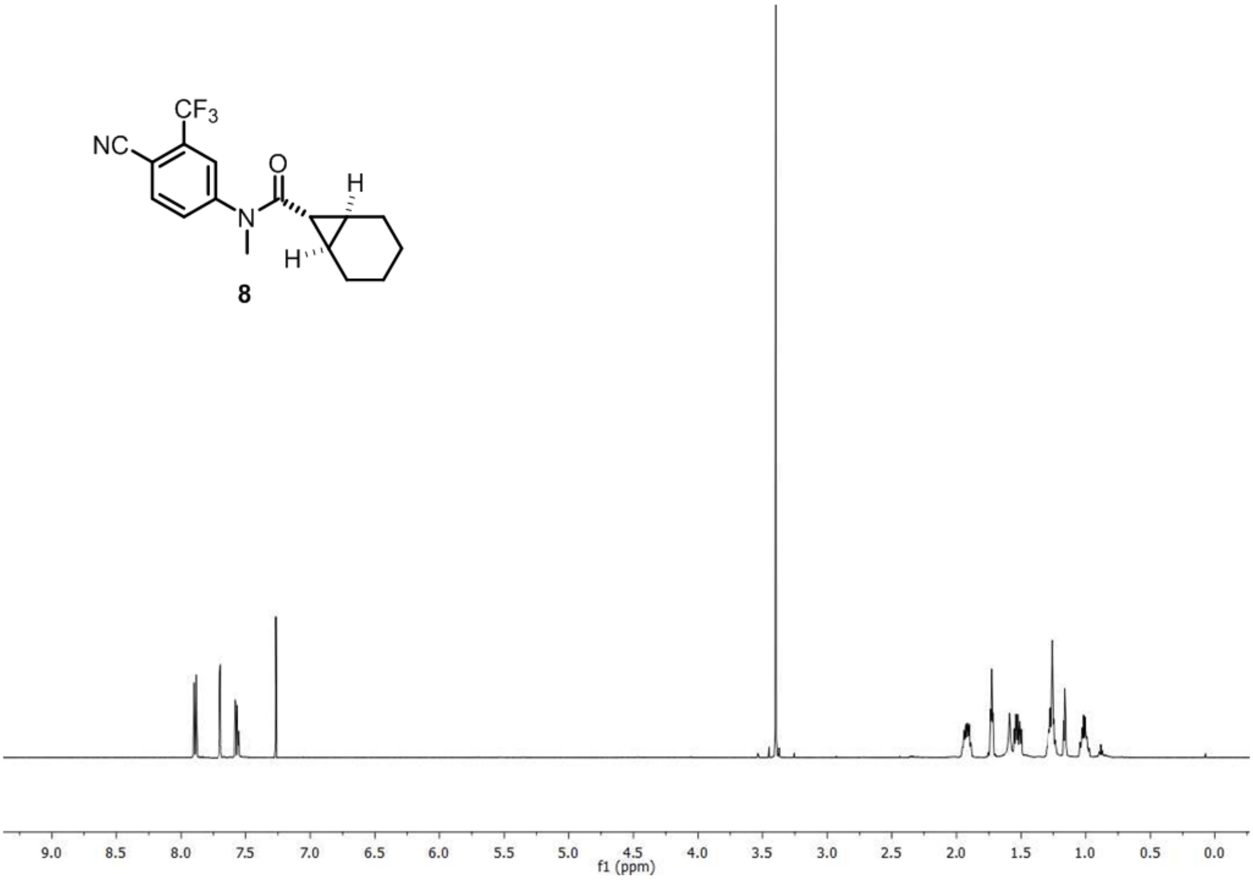
CF$_3$
NC
O
N
H
H
8
9.0 8.5 8.0 7.5 7.0 6.5 6.0 5.5 5.0 4.5 4.0 3.5 3.0 2.5 2.0 1.5 1.0 0.5 0.0
f1 (ppm)

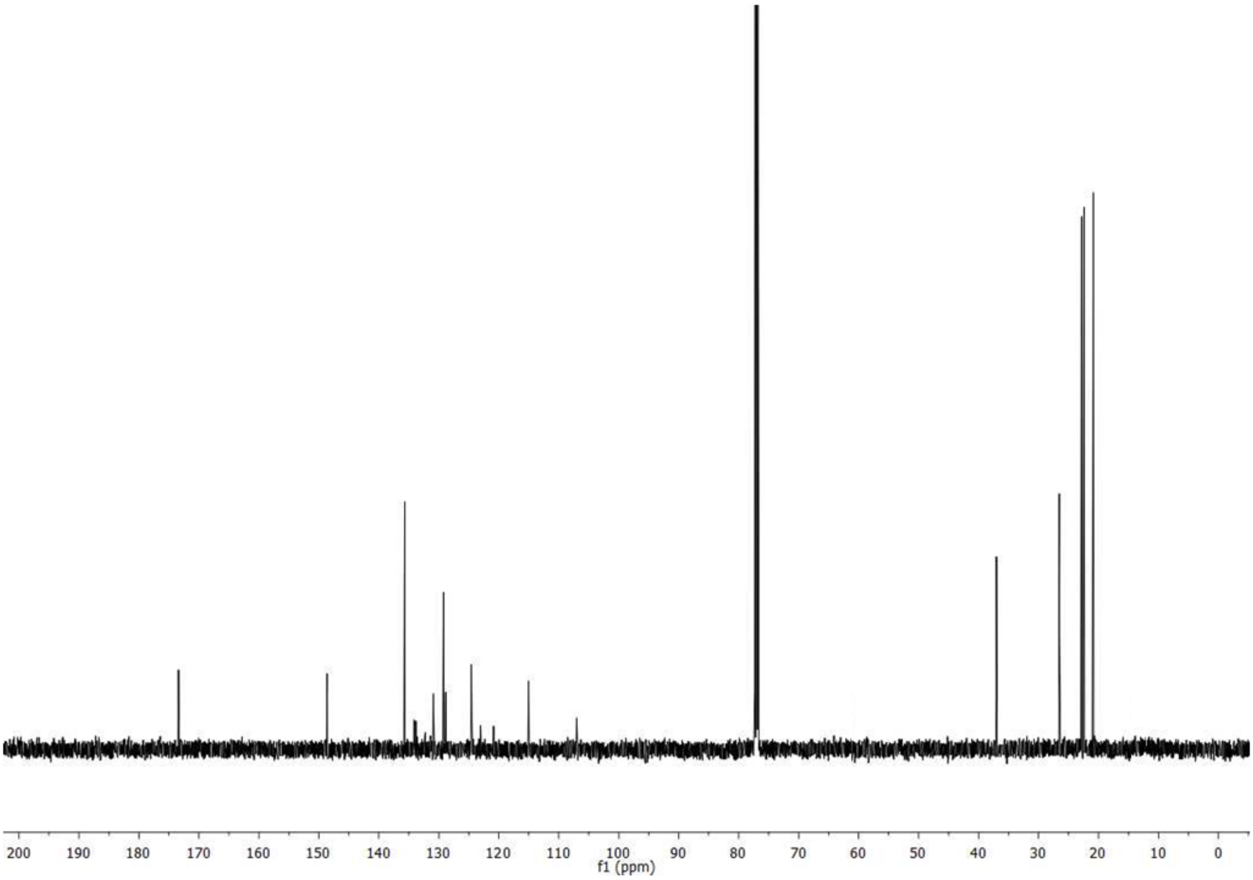
200 190 180 170 160 150 140 130 120 110 100 90 80 70 60 50 40 30 20 10 0
f1 (ppm)

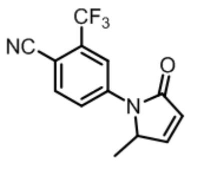

9

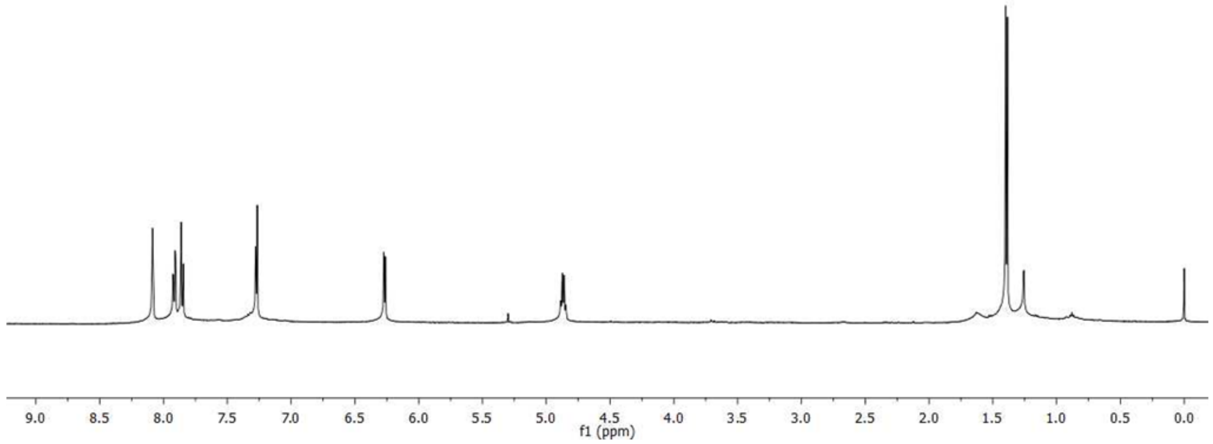
9.0
8.5
8.0
7.5
7.0
6.5
6.0
5.5
5.0
4.5
4.0
3.5
3.0
2.5
2.0
1.5
1.0
0.5
0.0
f1 (ppm)

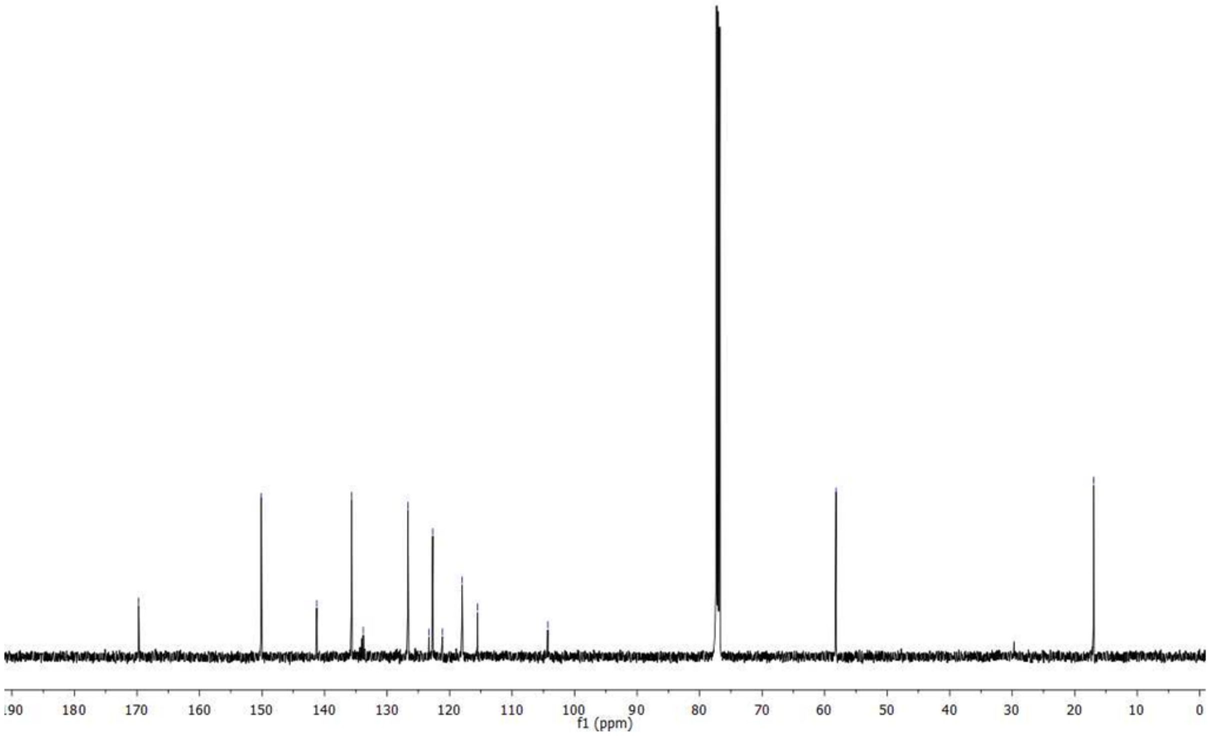
180
170
160
150
140
130
120
110
100
90
80
70
60
50
40
30
20
10
0
f1 (ppm)

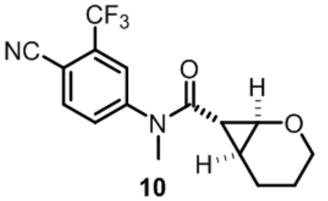
CF3
NC
O
H
O
N
H

10

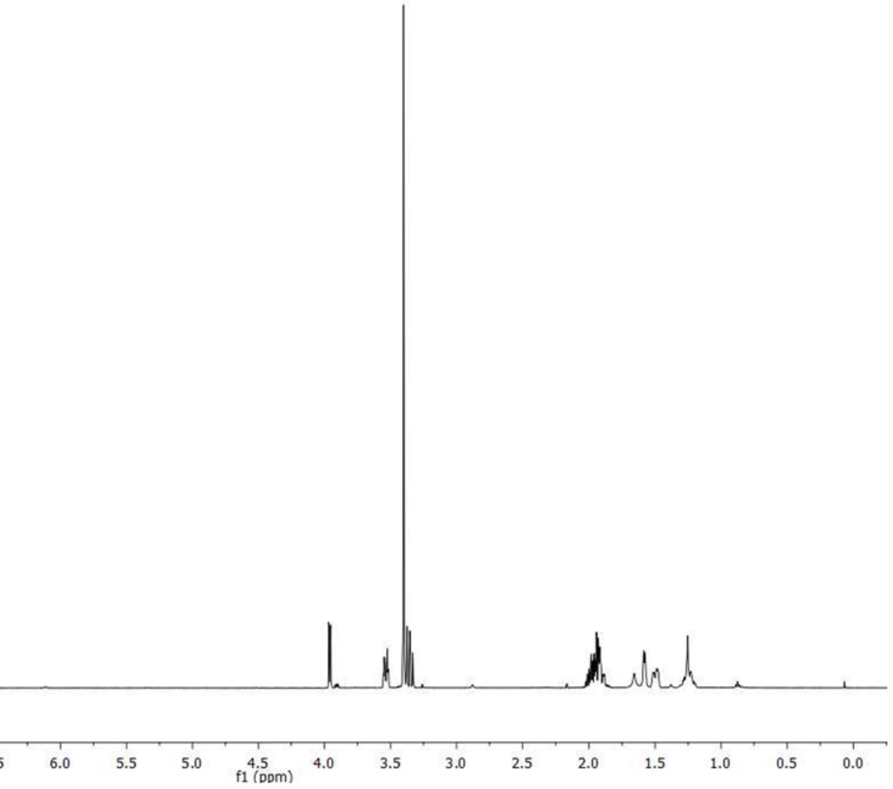
9.0 8.5 8.0 7.5 7.0 6.5 6.0 5.5 5.0 4.5 4.0 3.5 3.0 2.5 2.0 1.5 1.0 0.5 0.0
f1 (ppm)

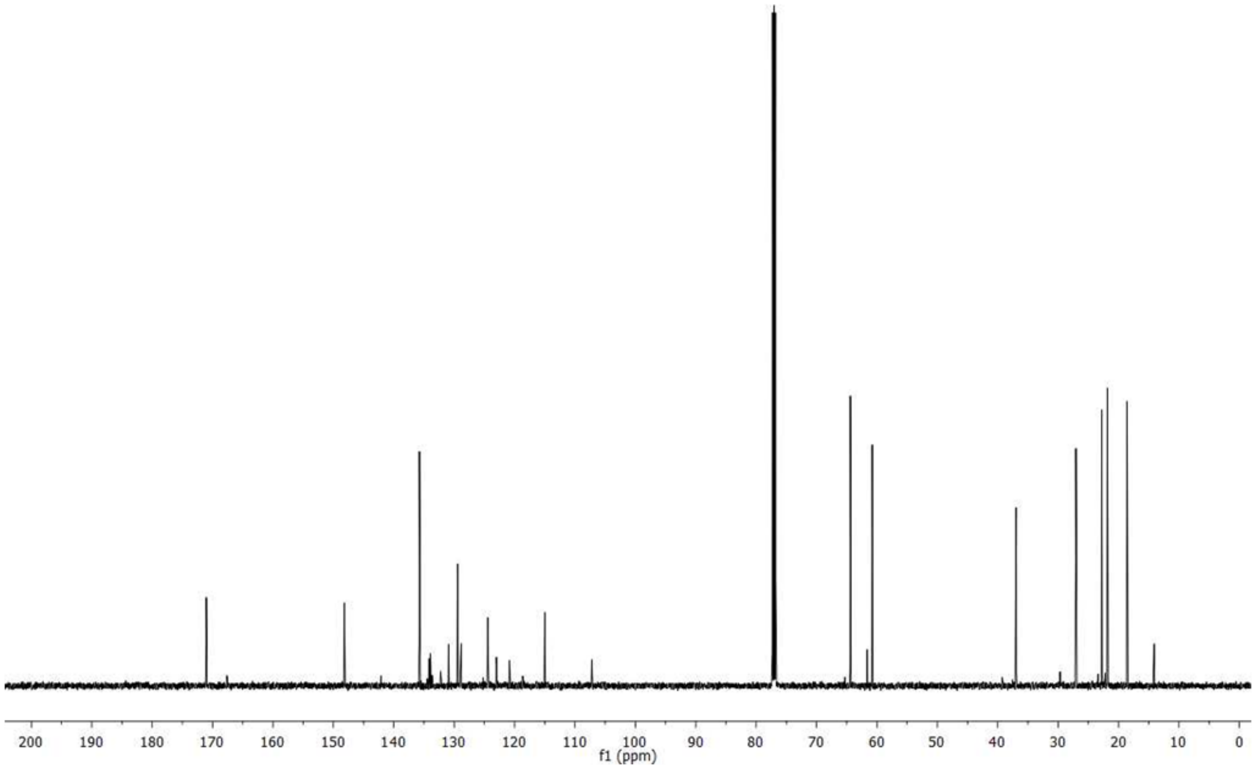
200 190 180 170 160 150 140 130 120 110 100 90 80 70 60 50 40 30 20 10 0
f1 (ppm)

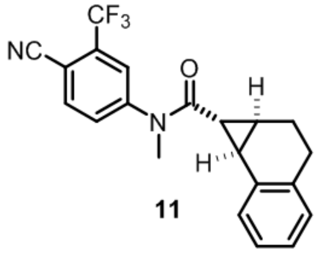

11

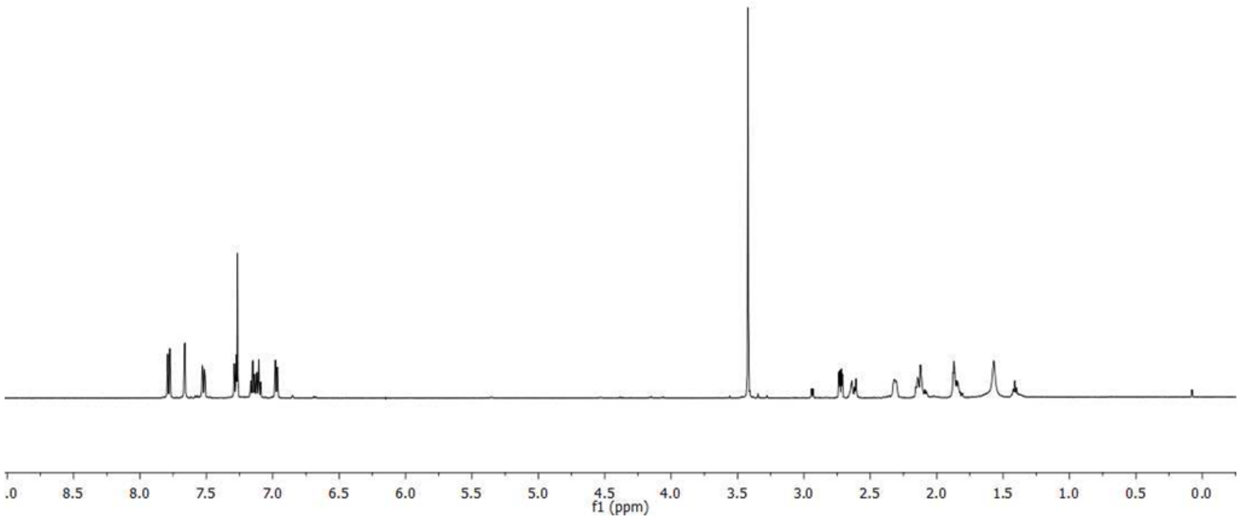

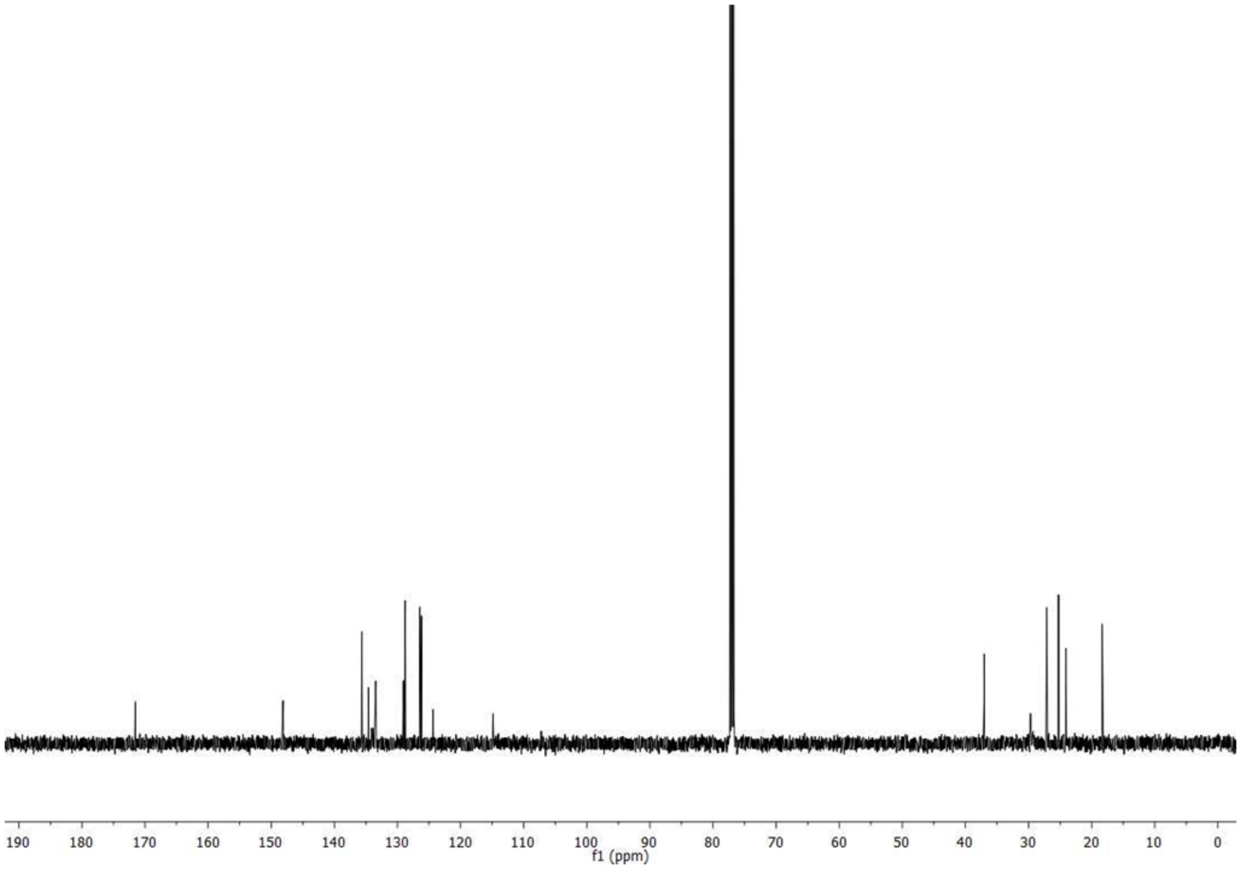

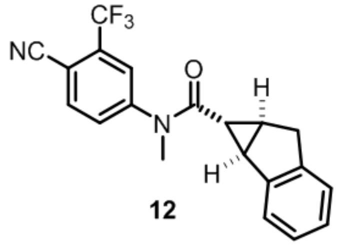
CF3
NC
O
N
H
H

12

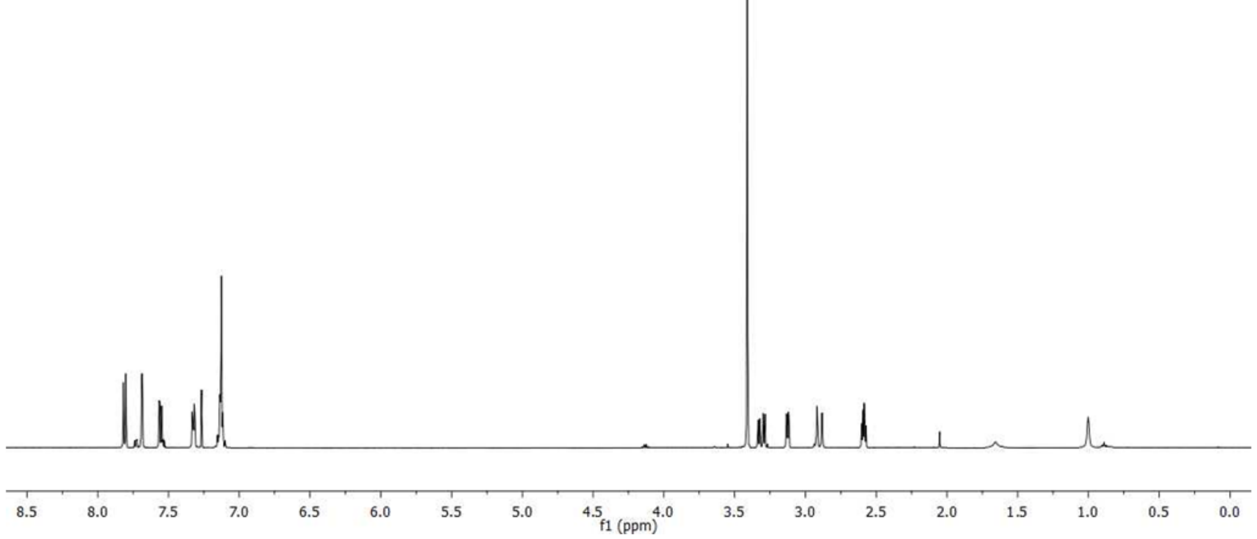
8.5
8.0
7.5
7.0
6.5
6.0
5.5
5.0
4.5
4.0
3.5
3.0
2.5
2.0
1.5
1.0
0.5
0.0
f1 (ppm)

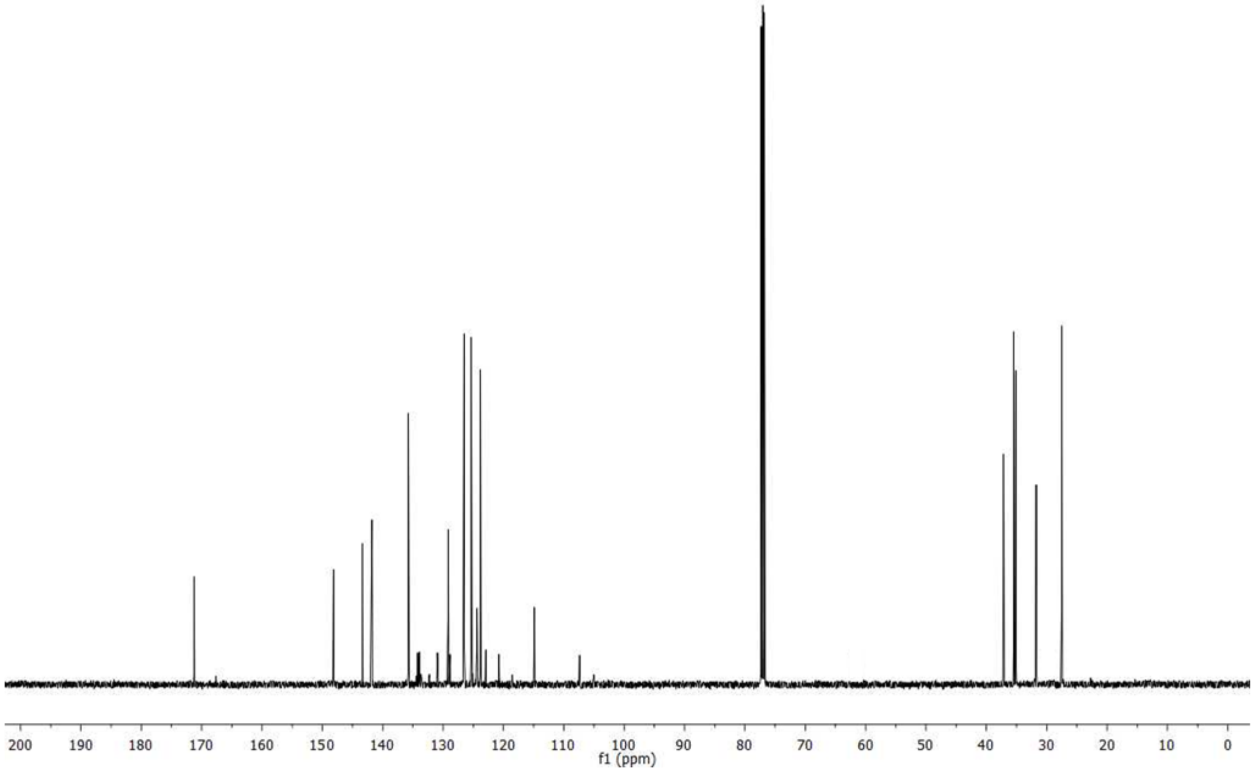
200
190
180
170
160
150
140
130
120
110
100
90
80
70
60
50
40
30
20
10
0
f1 (ppm)

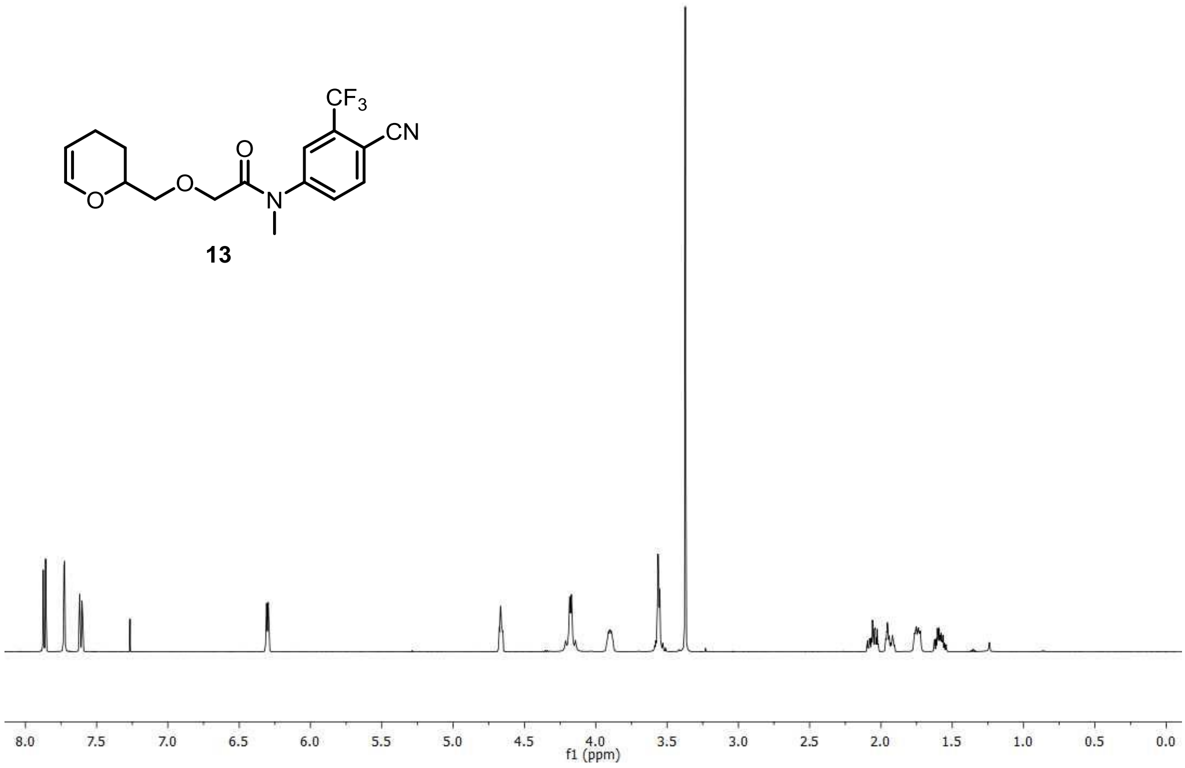
CF3
CN
O
N
O
O
13
8.0 7.5 7.0 6.5 6.0 5.5 5.0 4.5 4.0 3.5 3.0 2.5 2.0 1.5 1.0 0.5 0.0
f1 (ppm)

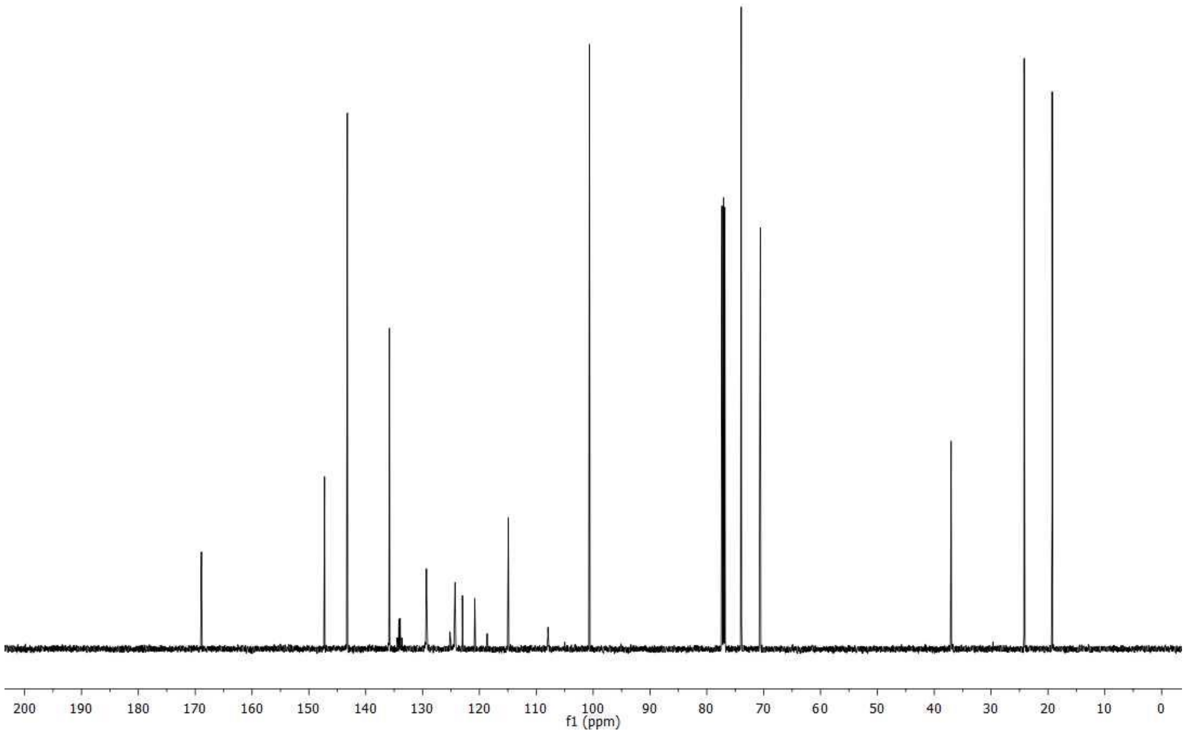
200 190 180 170 160 150 140 130 120 110 100 90 80 70 60 50 40 30 20 10 0
f1 (ppm)

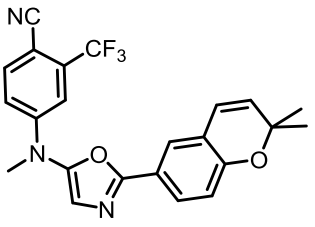
NC
CF3
N
O
N
O

14

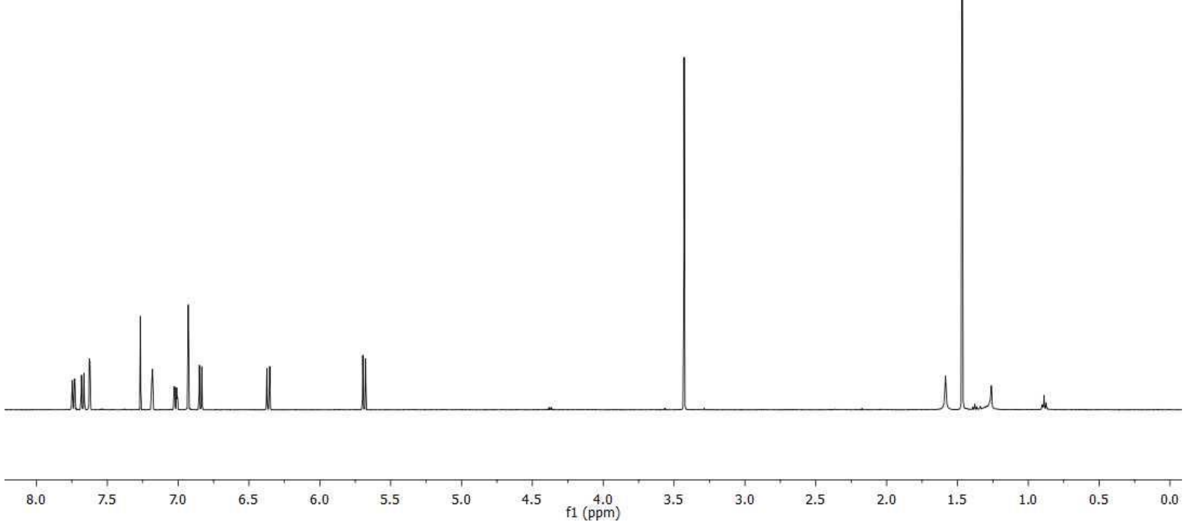
8.0
7.5
7.0
6.5
6.0
5.5
5.0
4.5
4.0
3.5
3.0
2.5
2.0
1.5
1.0
0.5
0.0
f1 (ppm)

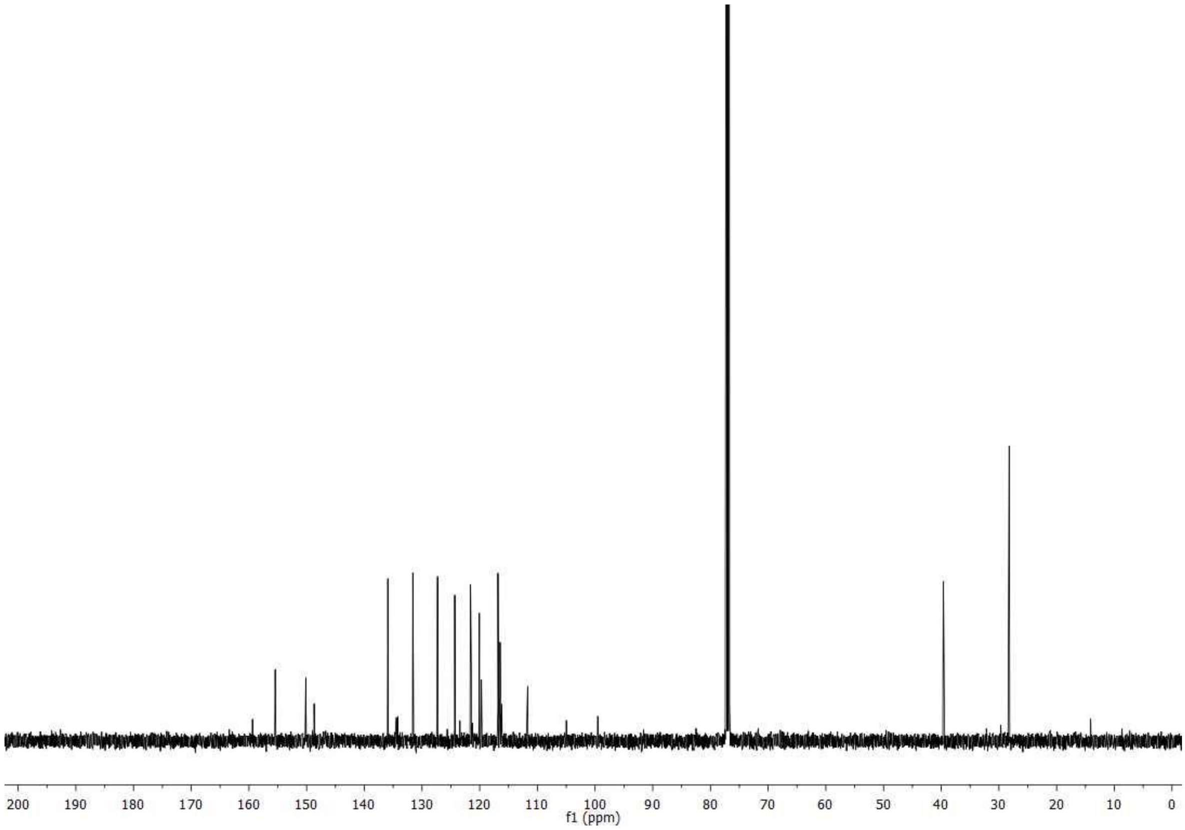
200
190
180
170
160
150
140
130
120
110
100
90
80
70
60
50
40
30
20
10
0
f1 (ppm)

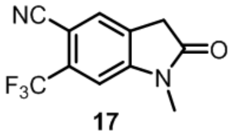
NC
O
F3C
N

17

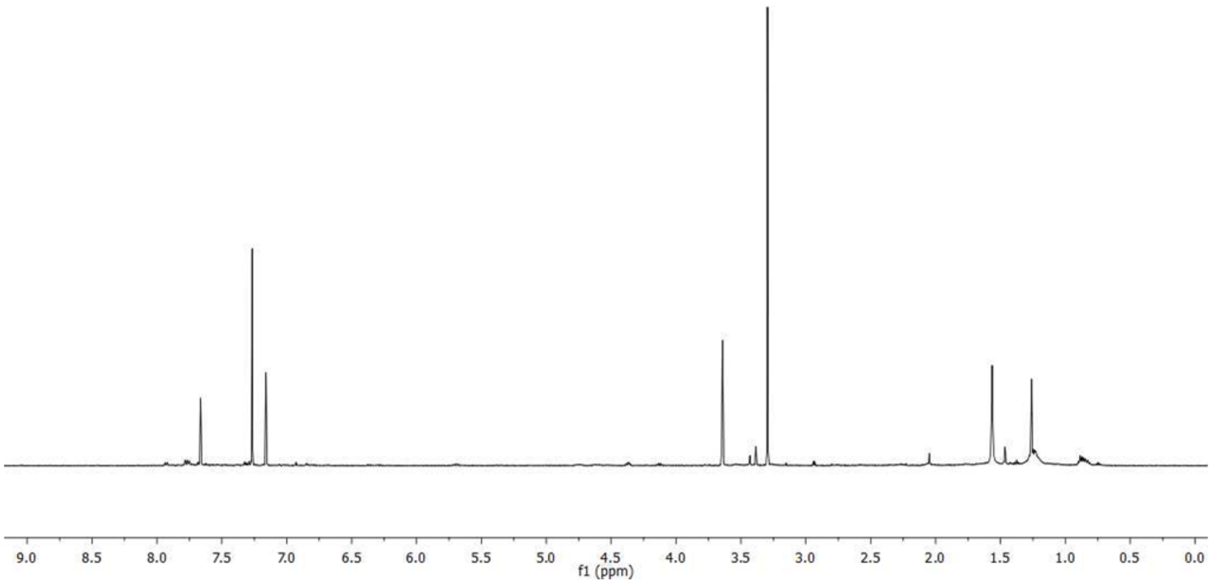
9.0 8.5 8.0 7.5 7.0 6.5 6.0 5.5 5.0 4.5 4.0 3.5 3.0 2.5 2.0 1.5 1.0 0.5 0.0
f1 (ppm)

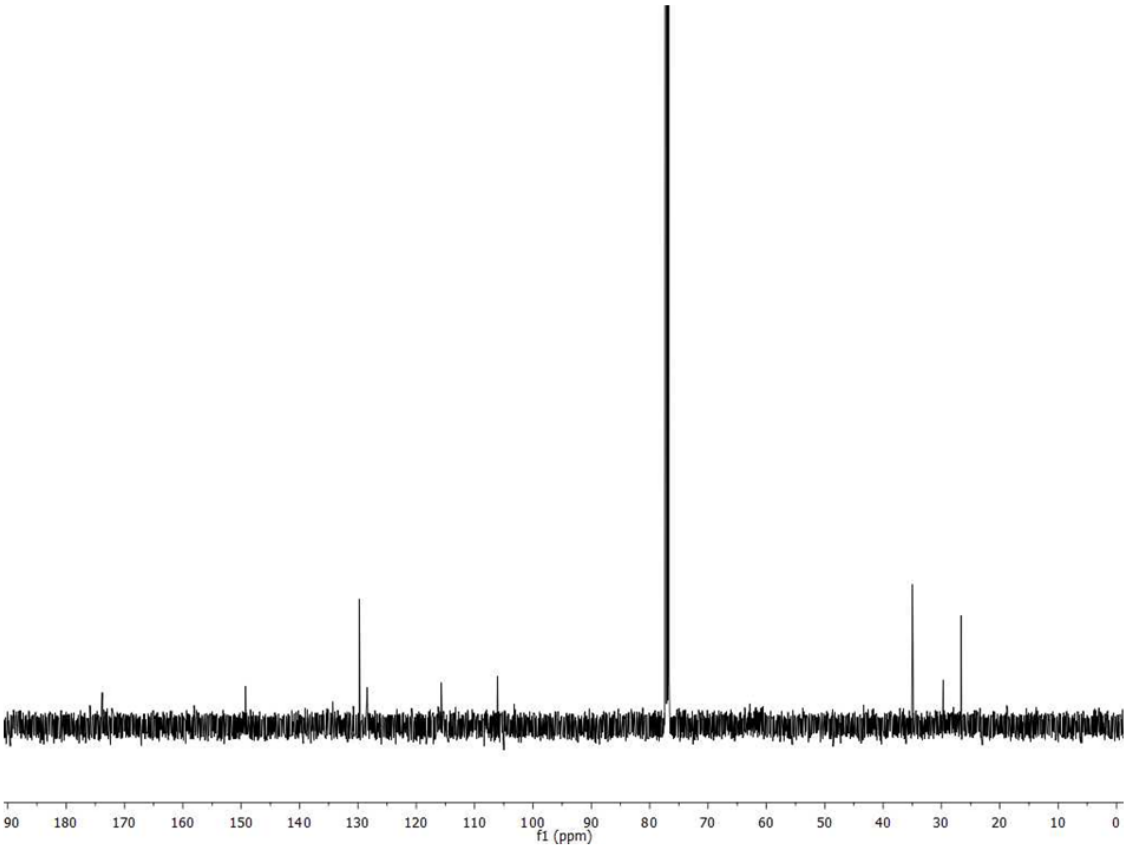
90 180 170 160 150 140 130 120 110 100 90 80 70 60 50 40 30 20 10 0
f1 (ppm)

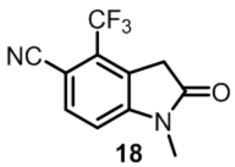
CF3
NC
O
N

18

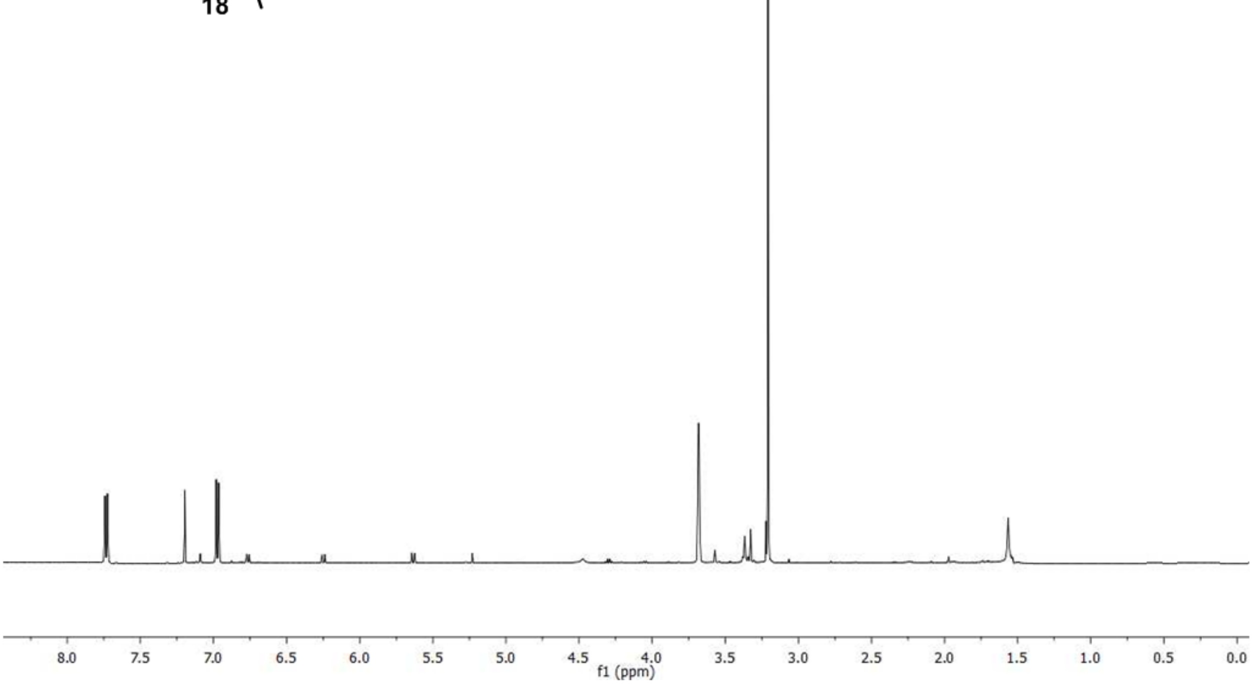
8.0 7.5 7.0 6.5 6.0 5.5 5.0 4.5 4.0 3.5 3.0 2.5 2.0 1.5 1.0 0.5 0.0
f1 (ppm)

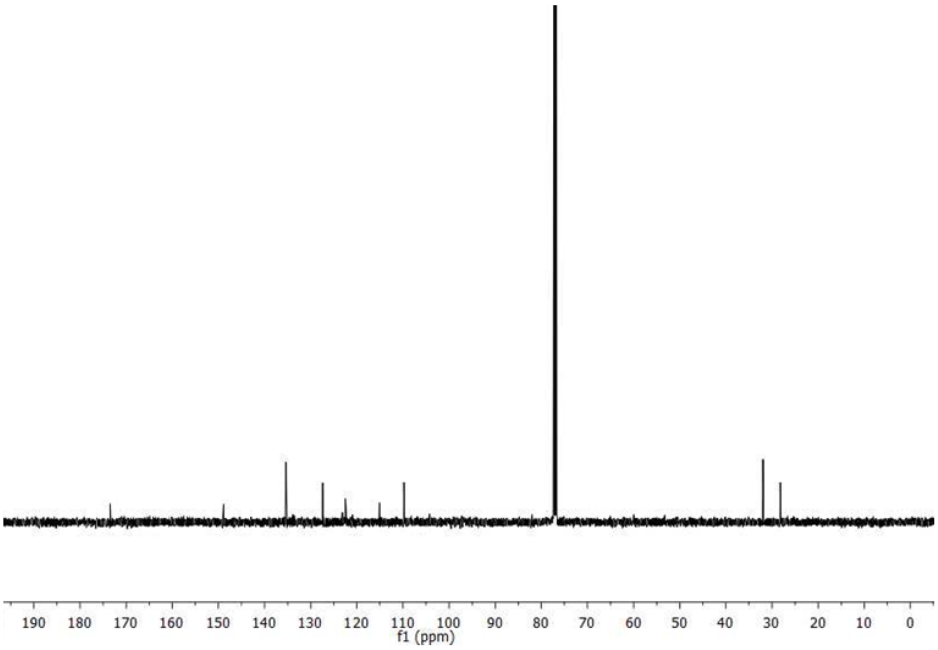
190 180 170 160 150 140 130 120 110 100 90 80 70 60 50 40 30 20 10 0
f1 (ppm)

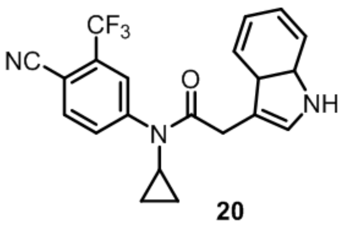

20

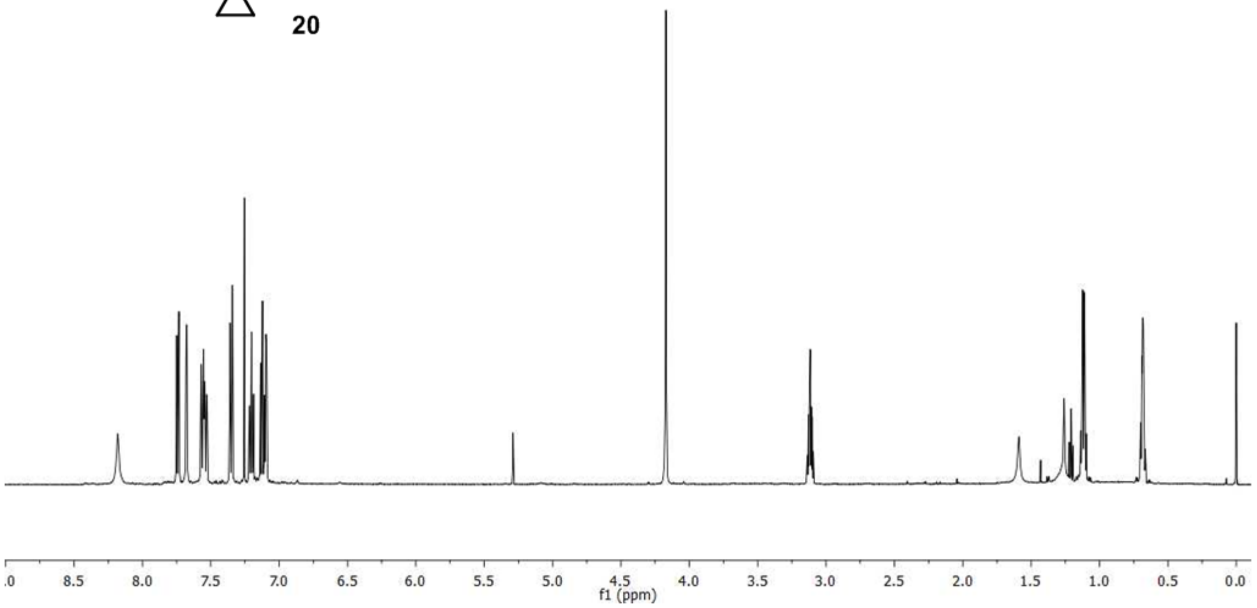

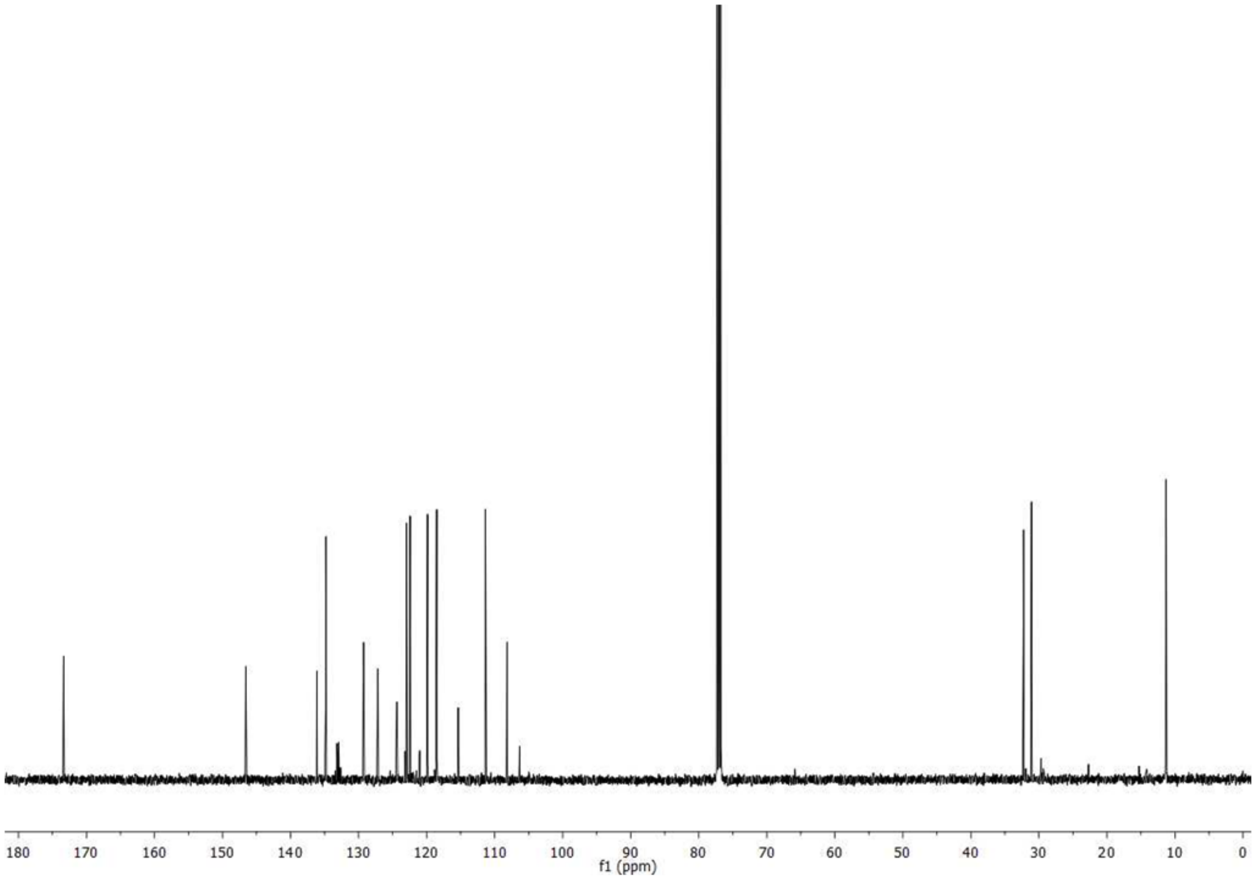

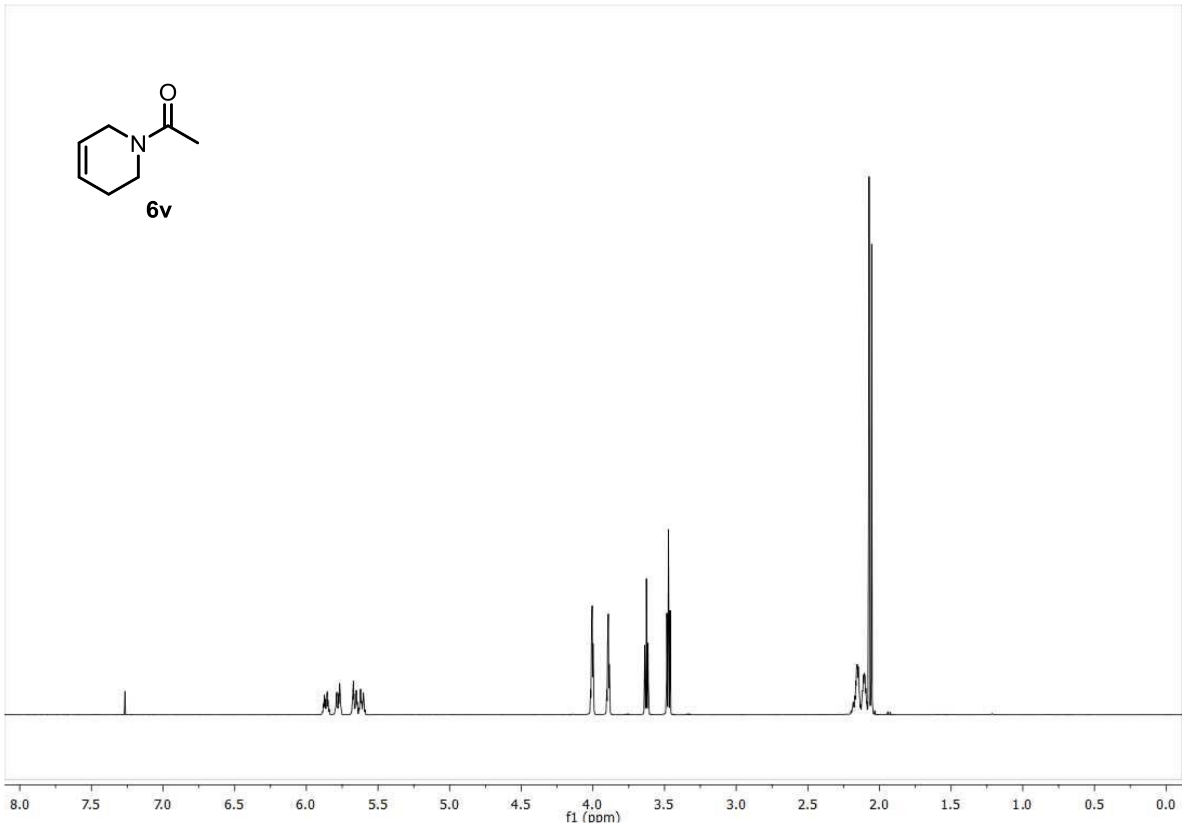
6v
8.0 7.5 7.0 6.5 6.0 5.5 5.0 4.5 4.0 3.5 3.0 2.5 2.0 1.5 1.0 0.5 0.0
f1 (ppm)

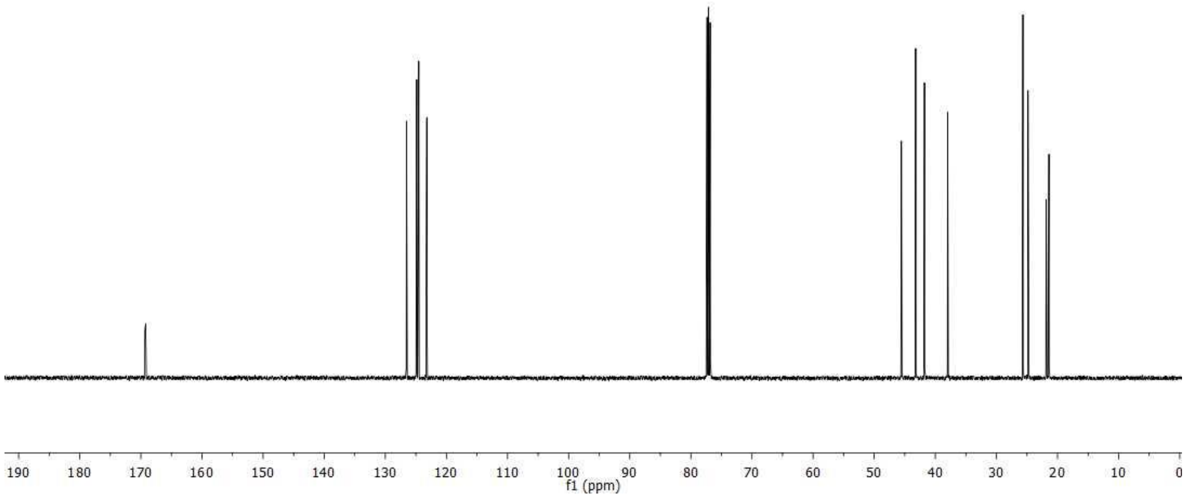
190 180 170 160 150 140 130 120 110 100 90 80 70 60 50 40 30 20 10 0
f1 (ppm)

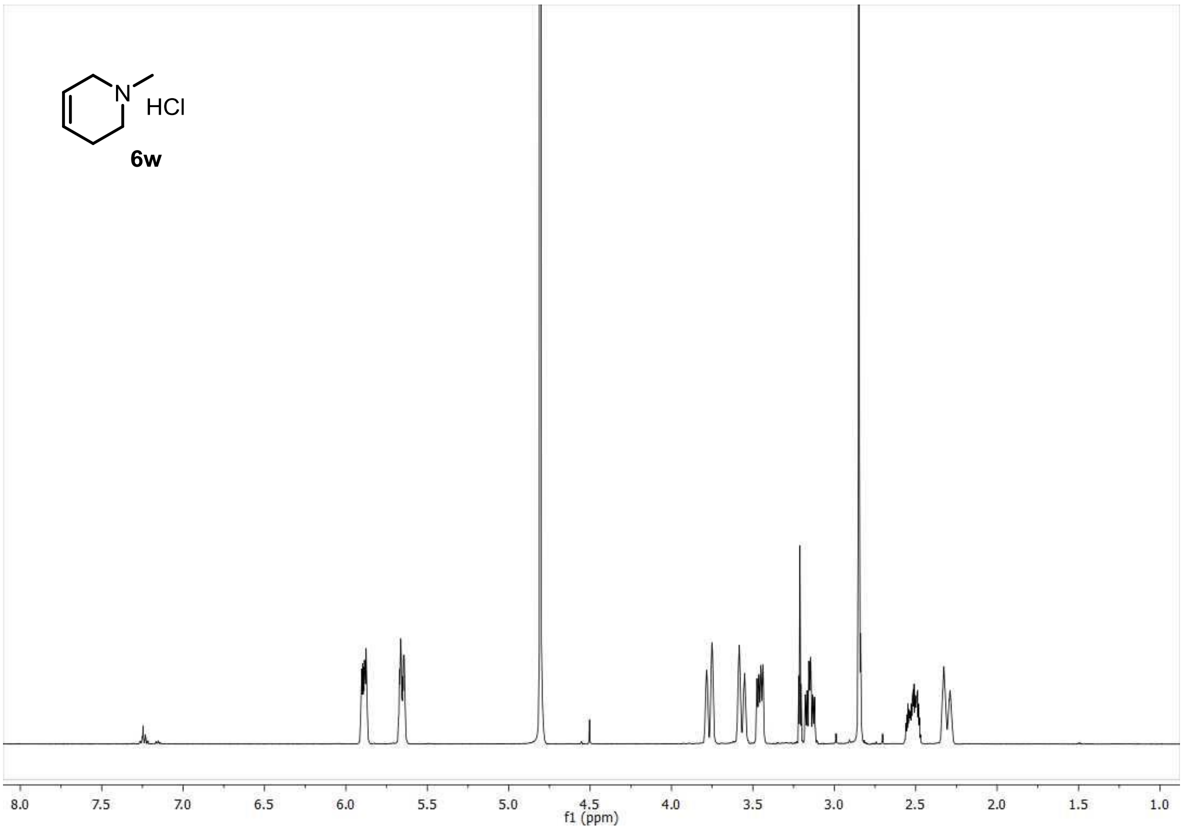
N
HCl
6w
8.0 7.5 7.0 6.5 6.0 5.5 5.0 4.5 4.0 3.5 3.0 2.5 2.0 1.5 1.0
f1 (ppm)

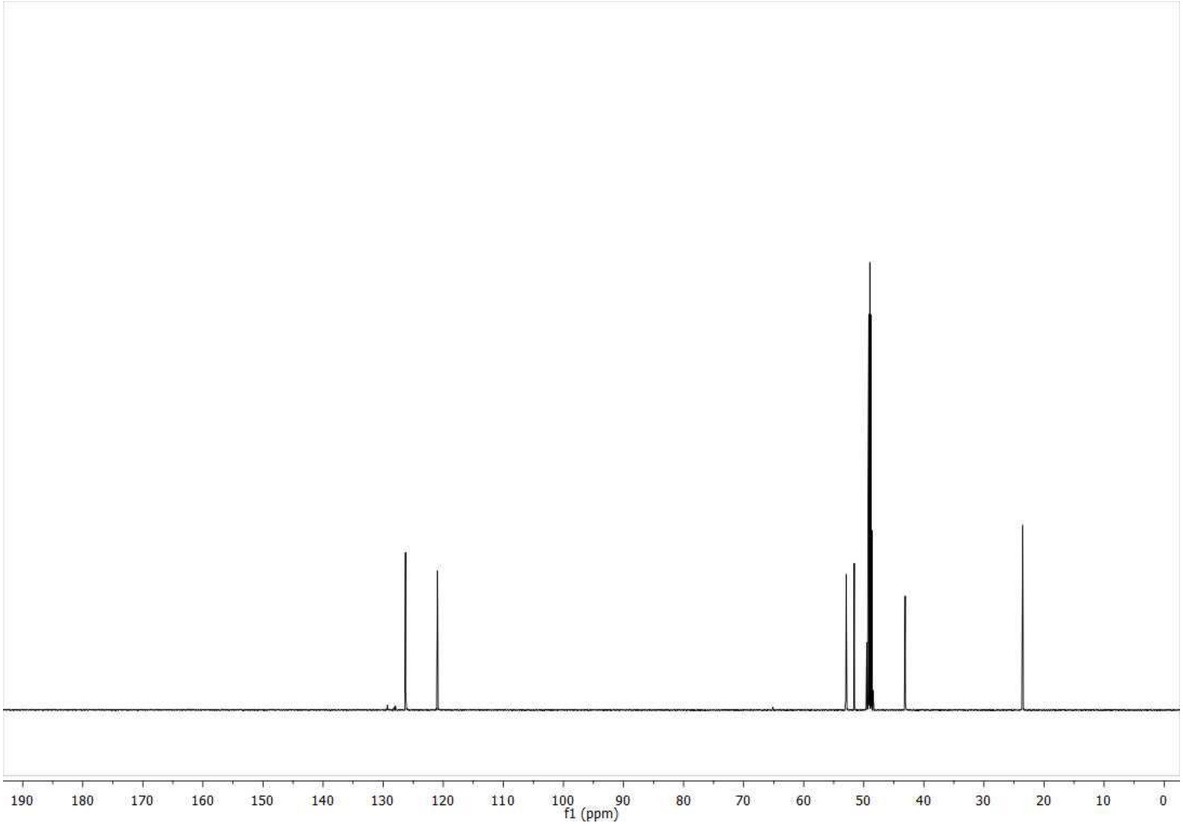
190 180 170 160 150 140 130 120 110 100 90 80 70 60 50 40 30 20 10 0
f1 (ppm)

Oxazole **21**

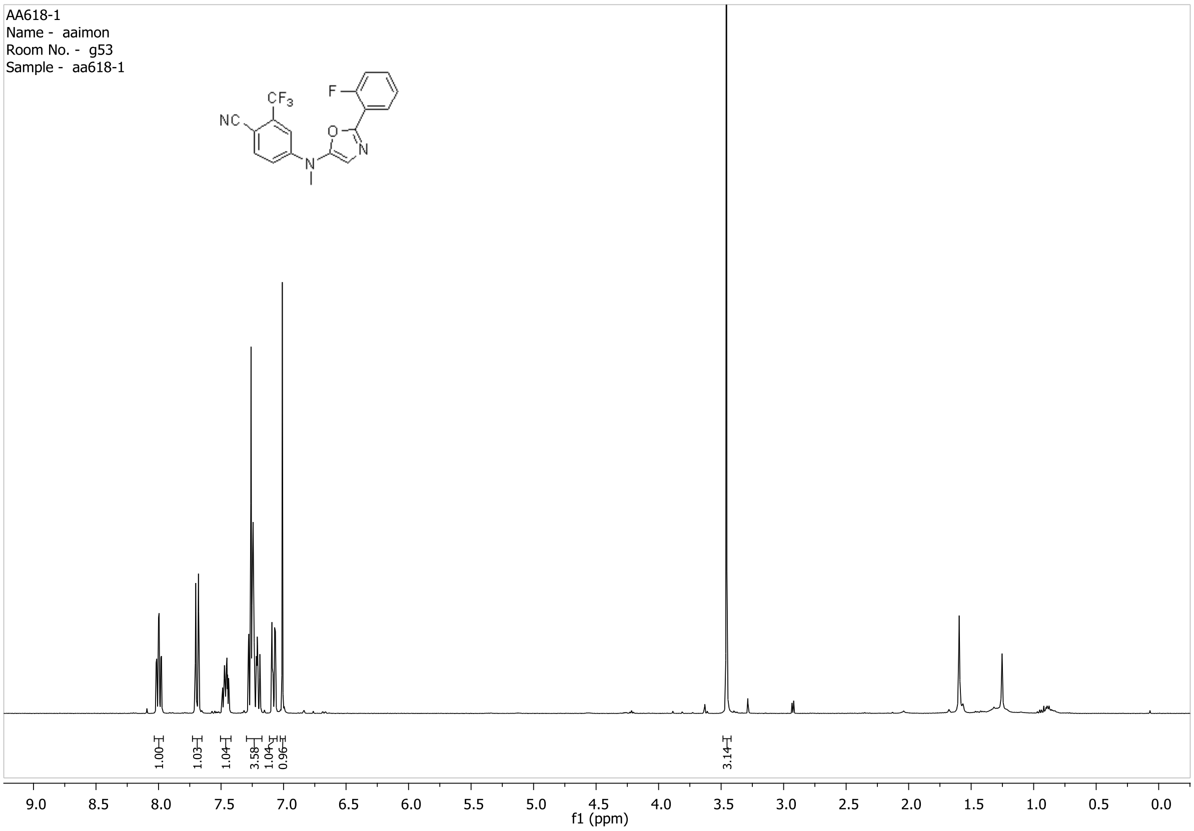
AA618-1
Name - aaimon
Room No. - g53
Sample - aa618-1
NC
CF3
F
O
N
N
1.00
1.03
1.04
3.58
1.04
0.96
3.14
9.0 8.5 8.0 7.5 7.0 6.5 6.0 5.5 5.0 4.5 4.0 3.5 3.0 2.5 2.0 1.5 1.0 0.5 0.0
f1 (ppm)

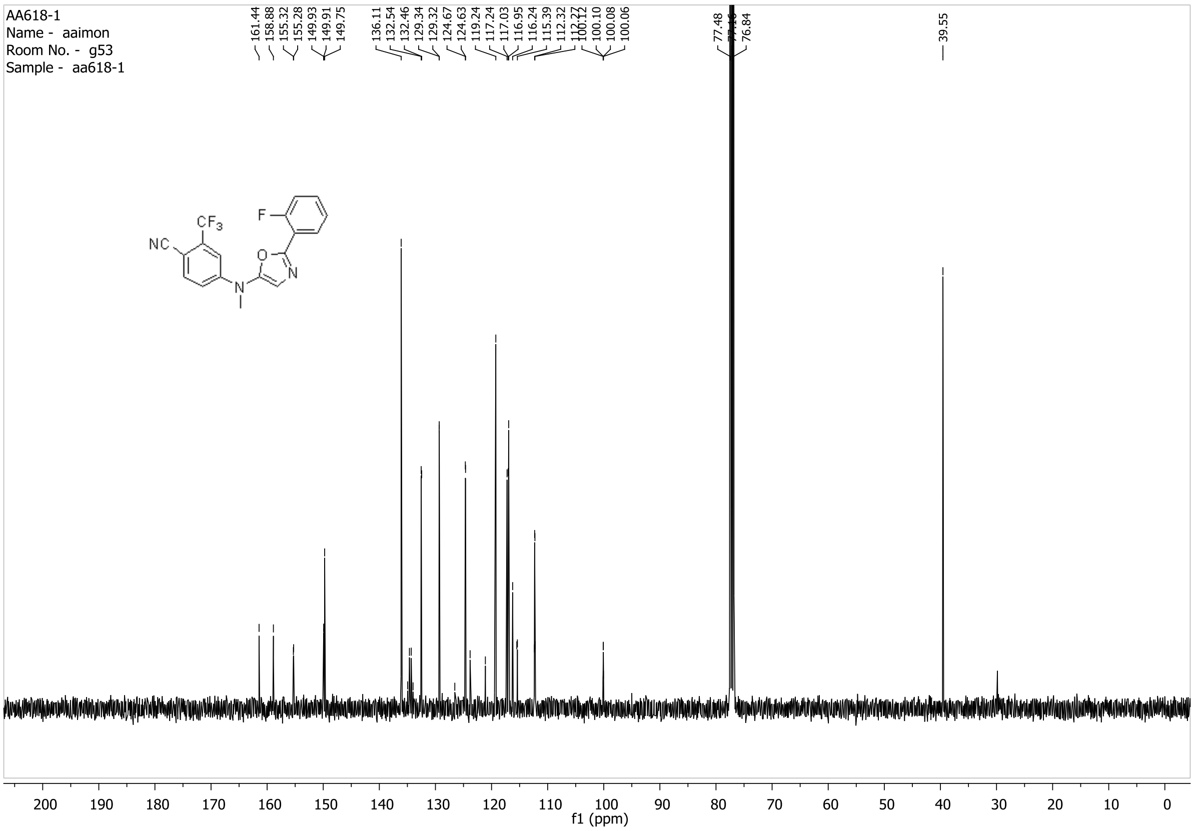
AA618-1
Name - aaimon
Room No. - g53
Sample - aa618-1
161.44
158.88
155.32
155.28
149.93
149.91
149.75
136.11
132.54
132.46
129.34
129.32
124.67
124.63
119.24
117.24
117.03
116.95
116.24
115.39
112.32
112.27
100.12
100.10
100.08
100.06
77.48
76.84
39.55
NC
CF3
F
O
N
N
200 190 180 170 160 150 140 130 120 110 100 90 80 70 60 50 40 30 20 10 0
f1 (ppm)

Oxazole **22**

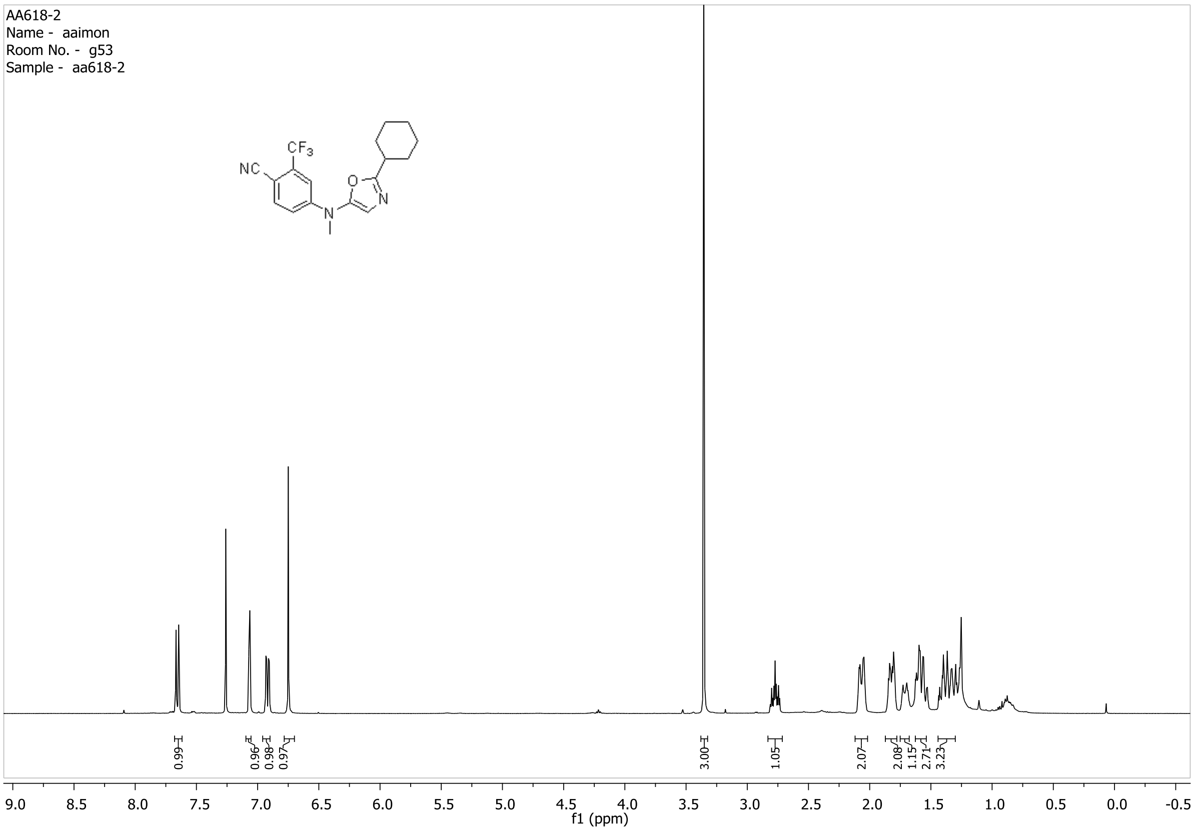
AA618-2
Name - aaimon
Room No. - g53
Sample - aa618-2
0.99
0.96
0.98
0.97
3.00
1.05
2.07
2.08
1.15
2.71
3.23
9.0 8.5 8.0 7.5 7.0 6.5 6.0 5.5 5.0 4.5 4.0 3.5 3.0 2.5 2.0 1.5 1.0 0.5 0.0 -0.5
f1 (ppm)

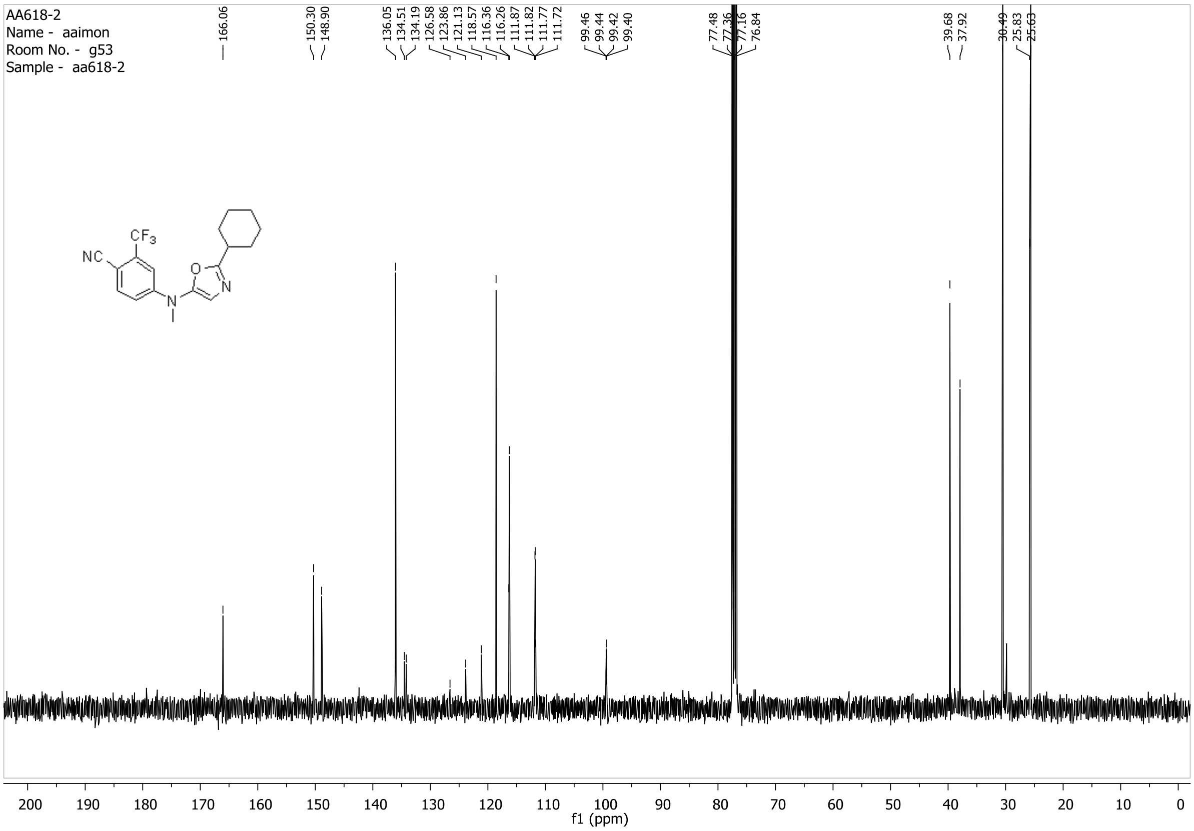
AA618-2
Name - aaimon
Room No. - g53
Sample - aa618-2
166.06
150.30
148.90
136.05
134.51
134.19
126.58
123.86
121.13
118.57
116.36
116.26
111.87
111.82
111.77
111.72
99.46
99.44
99.42
99.40
77.48
77.36
77.16
76.84
39.68
37.92
30.49
25.83
25.63
200 190 180 170 160 150 140 130 120 110 100 90 80 70 60 50 40 30 20 10 0
f1 (ppm)

Oxazole **23**

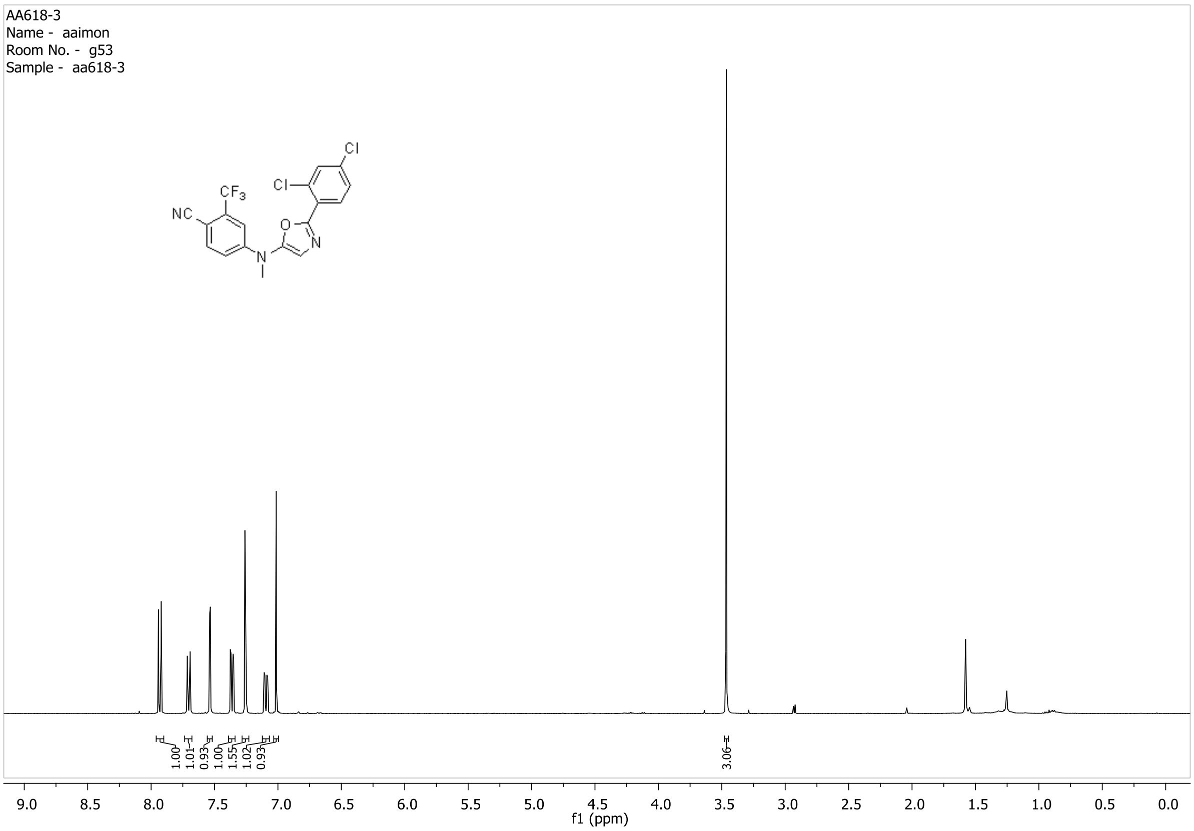

AA618-3
Name - aaimon
Room No. - g53
Sample - aa618-3
1.00
1.01
0.93
1.00
1.55
1.02
0.93
3.06
f1 (ppm)

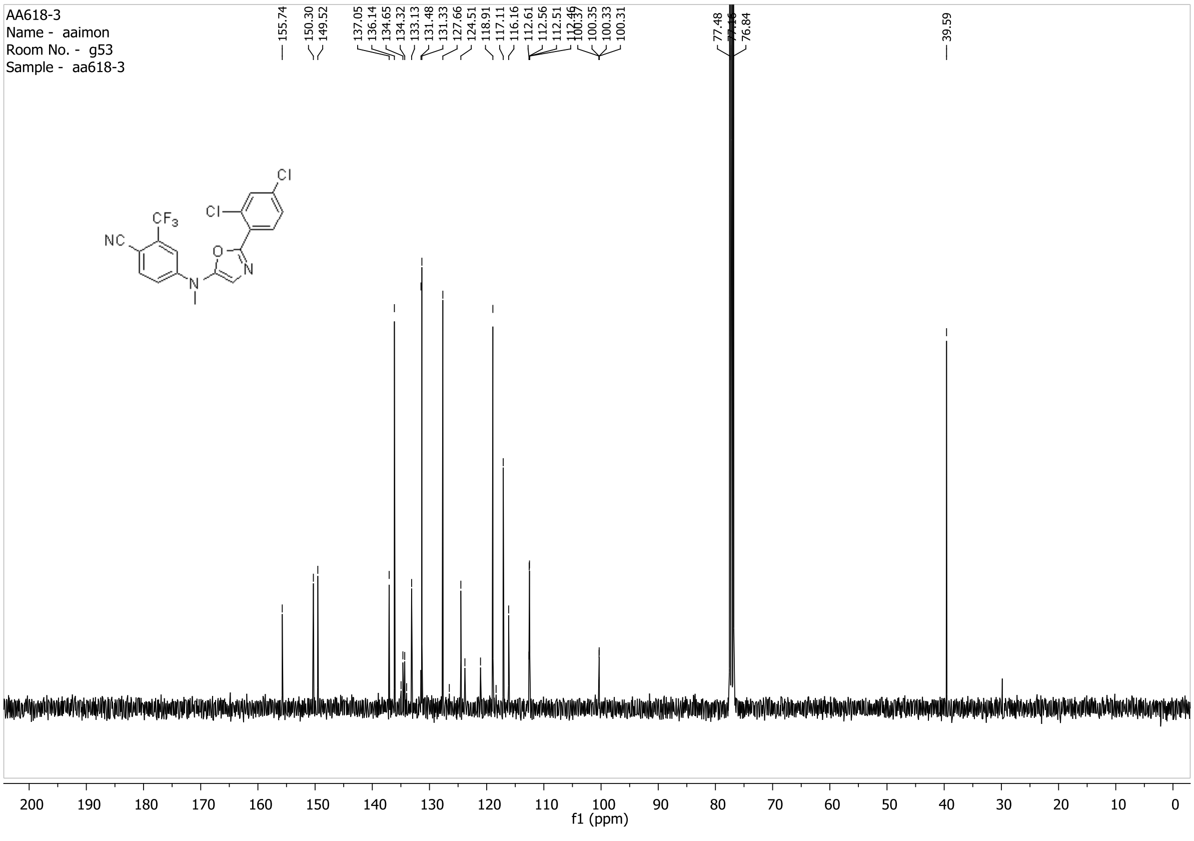

AA618-3
Name - aaimon
Room No. - g53
Sample - aa618-3
155.74
150.30
149.52
137.05
136.14
134.65
134.32
133.13
131.48
131.33
127.66
124.51
118.91
117.11
116.16
112.61
112.56
112.51
100.35
100.33
100.31
77.48
76.84
39.59
f1 (ppm)

Oxazole **24**

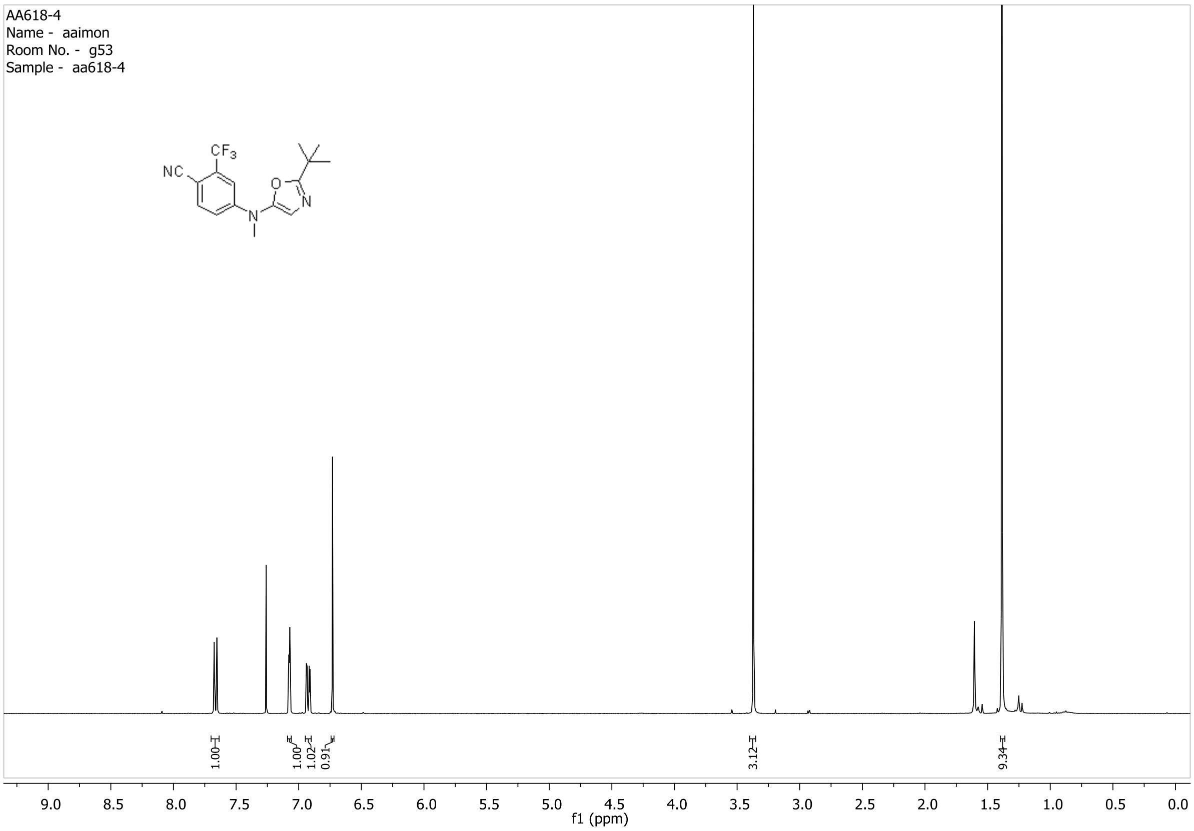
AA618-4
Name - aaimon
Room No. - g53
Sample - aa618-4
NC
CF3
N
O
N
1.00
1.00
1.02
0.91
3.12
9.34
9.0
8.5
8.0
7.5
7.0
6.5
6.0
5.5
5.0
4.5
4.0
3.5
3.0
2.5
2.0
1.5
1.0
0.5
0.0
f1 (ppm)

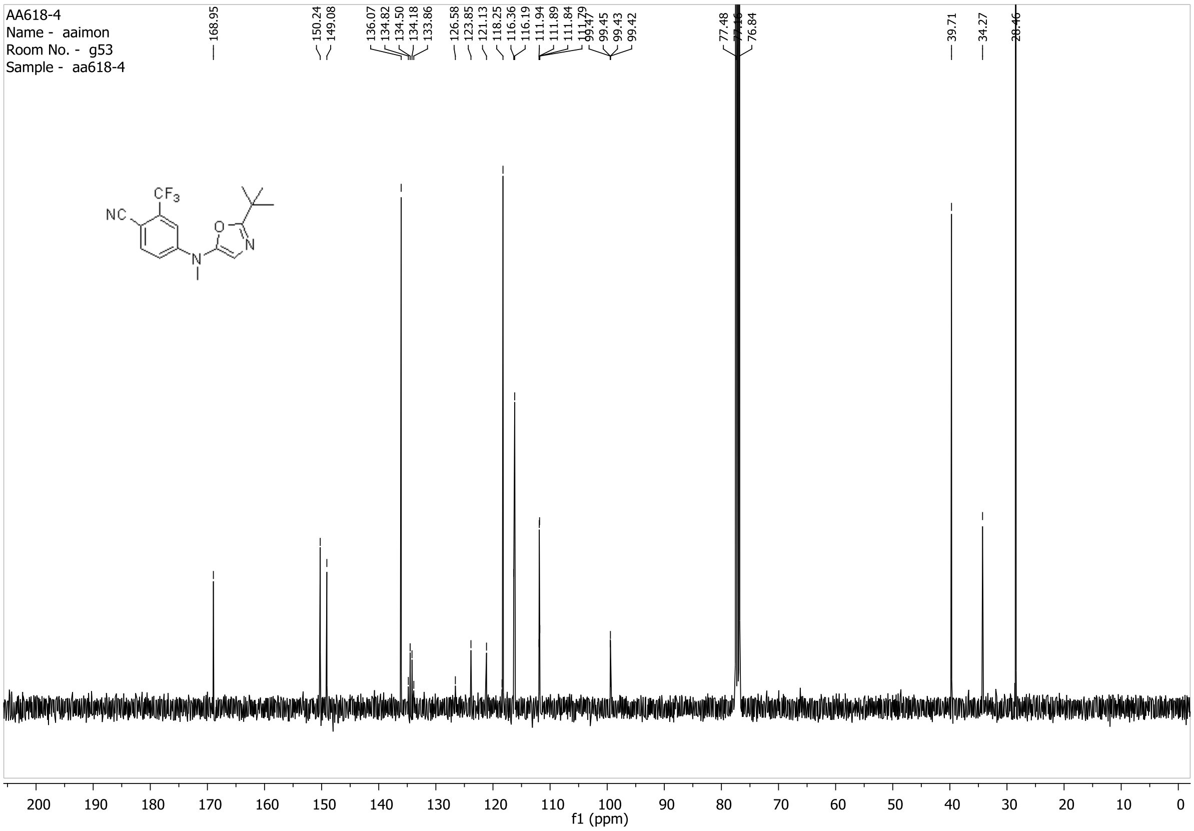
AA618-4
Name - aaimon
Room No. - g53
Sample - aa618-4
168.95
150.24
149.08
136.07
134.82
134.50
134.18
133.86
126.58
123.85
121.13
118.25
116.36
116.19
111.94
111.89
111.84
99.47
99.45
99.43
99.42
77.48
76.84
39.71
34.27
NC
CF3
N
O
N
200
190
180
170
160
150
140
130
120
110
100
90
80
70
60
50
40
30
20
10
0
f1 (ppm)

Oxazole **25**

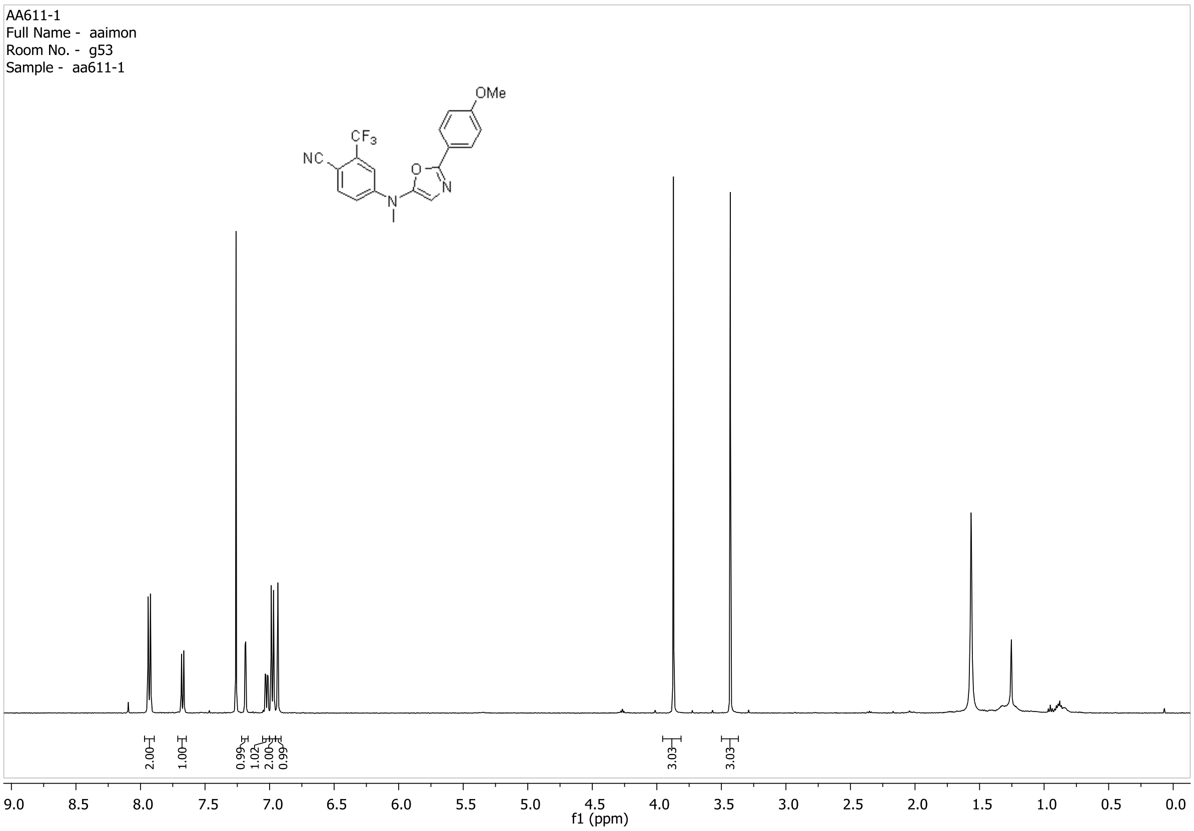

AA611-1
Full Name - aaimon
Room No. - g53
Sample - aa611-1
OMe
CF3
NC
O
N
N
2.00
1.00
0.99
1.02
2.00
0.99
3.03
3.03
9.0 8.5 8.0 7.5 7.0 6.5 6.0 5.5 5.0 4.5 4.0 3.5 3.0 2.5 2.0 1.5 1.0 0.5 0.0
f1 (ppm)

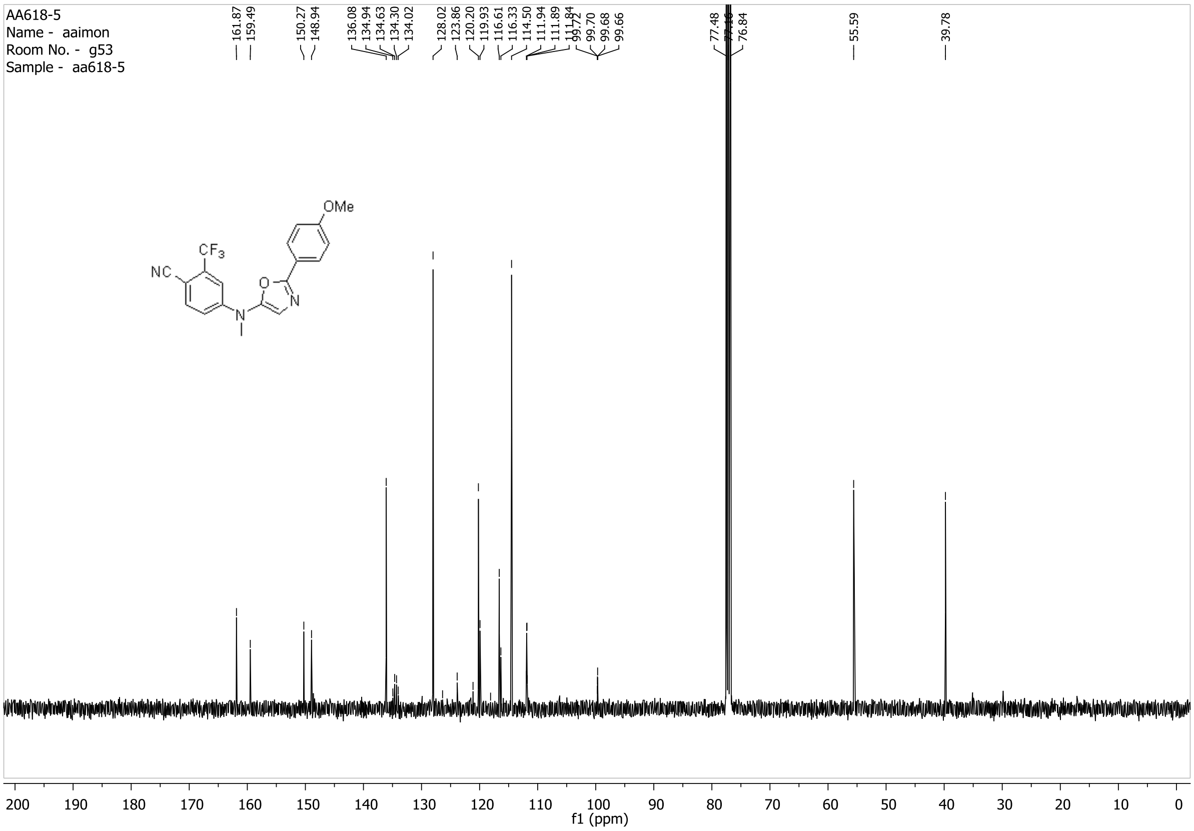

AA618-5
Name - aaimon
Room No. - g53
Sample - aa618-5
161.87
159.49
150.27
148.94
136.08
134.94
134.63
134.30
134.02
128.02
123.86
120.20
119.93
116.61
116.33
114.50
111.94
111.89
77.48
76.84
55.59
39.78
99.70
99.68
99.66
OMe
CF3
NC
O
N
N
200 190 180 170 160 150 140 130 120 110 100 90 80 70 60 50 40 30 20 10 0
f1 (ppm)

Oxazole **26**

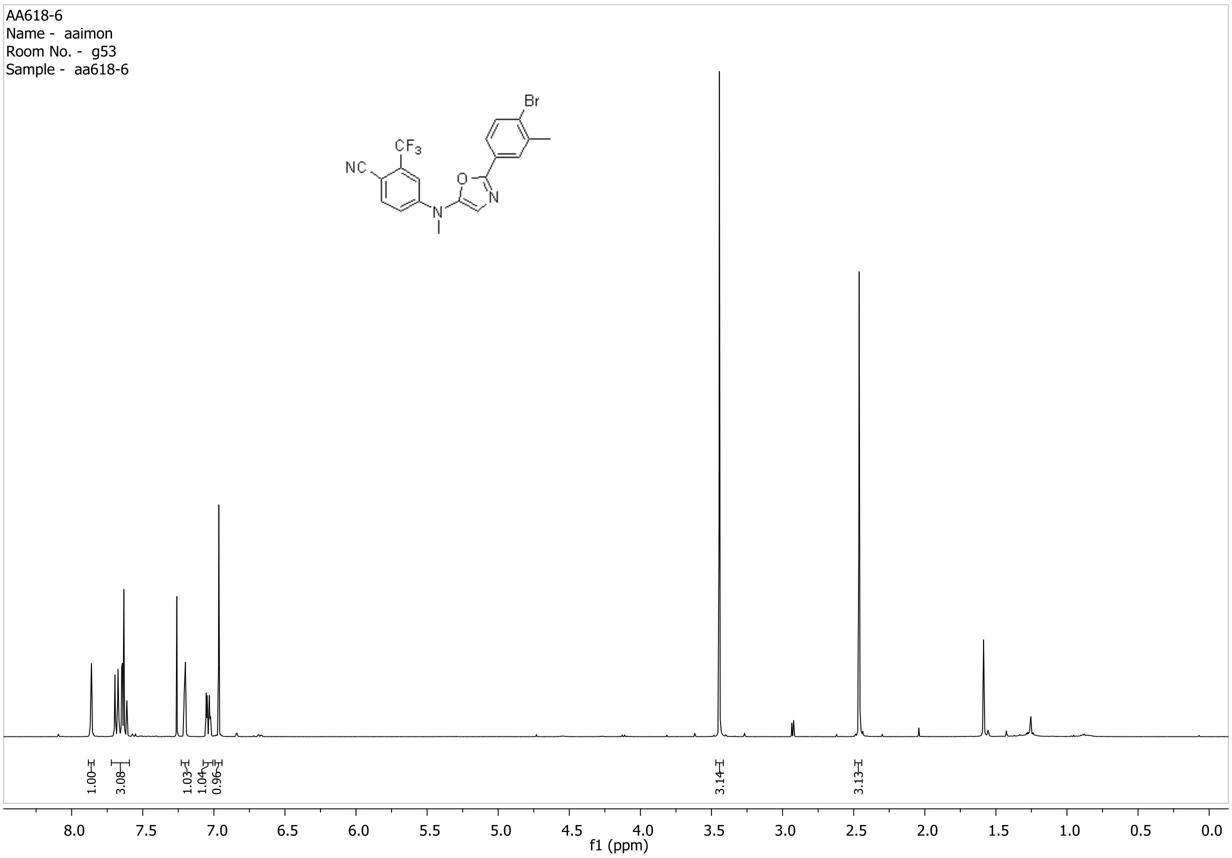

AA618-6
Name - aaimon
Room No. - g53
Sample - aa618-6
Br
CF3
NC
O
N
N
1.00
3.08
1.03
1.04
0.96
3.14
3.13
8.0
7.5
7.0
6.5
6.0
5.5
5.0
4.5
4.0
3.5
3.0
2.5
2.0
1.5
1.0
0.5
0.0
f1 (ppm)

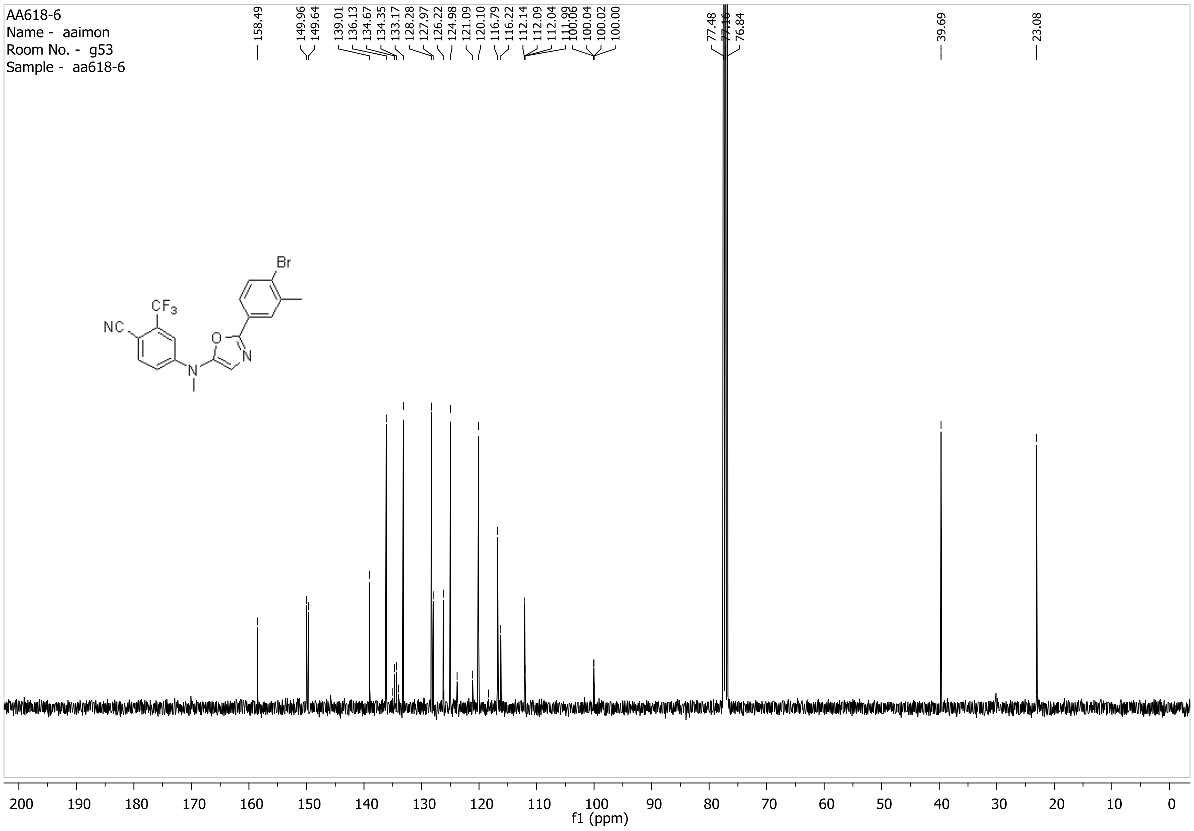

AA618-6
Name - aaimon
Room No. - g53
Sample - aa618-6
158.49
149.96
149.64
139.01
136.13
134.67
134.35
133.17
128.28
127.97
126.22
124.98
121.09
120.10
116.79
116.22
112.14
112.09
112.04
100.04
100.02
100.00
77.48
76.84
39.69
23.08
Br
CF3
NC
O
N
N
200
190
180
170
160
150
140
130
120
110
100
90
80
70
60
50
40
30
20
10
0
f1 (ppm)

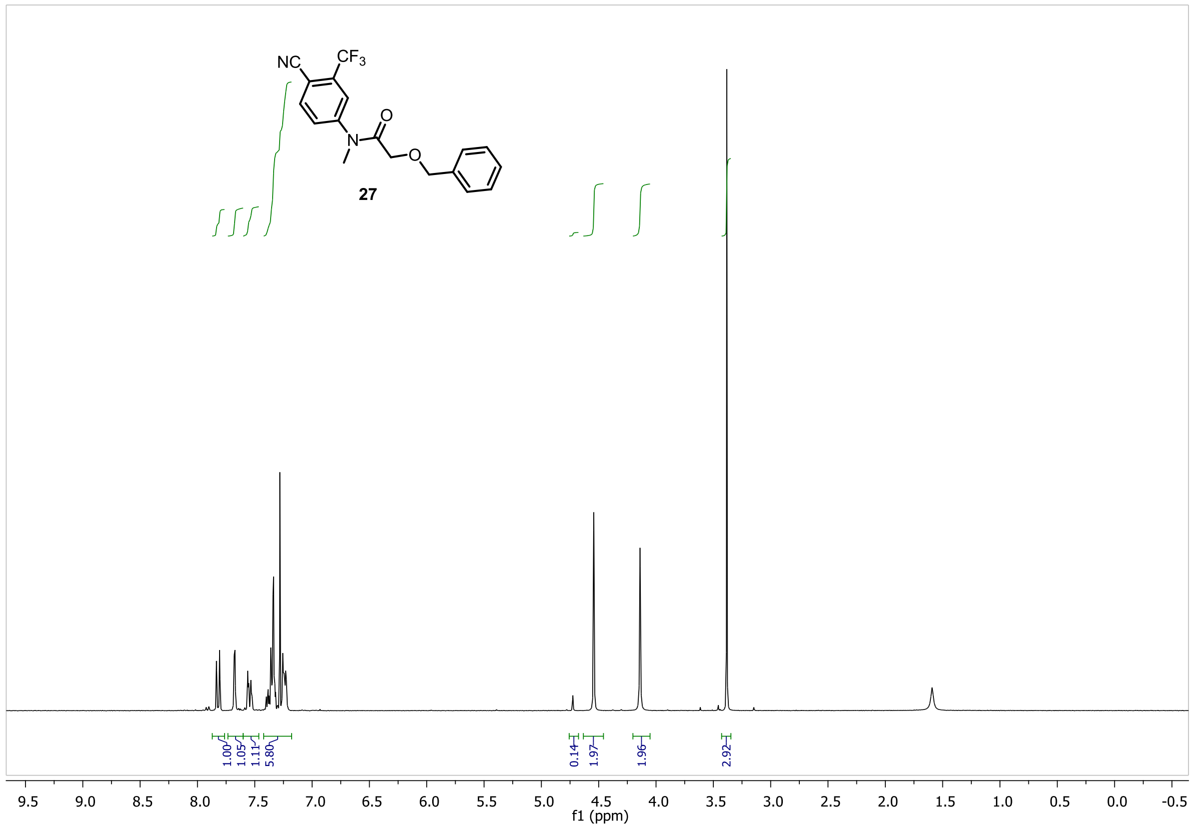
NC
CF3
N
O
O
27
1.00
1.05
1.11
5.80
0.14
1.97
1.96
2.92
9.5 9.0 8.5 8.0 7.5 7.0 6.5 6.0 5.5 5.0 4.5 4.0 3.5 3.0 2.5 2.0 1.5 1.0 0.5 0.0 -0.5
f1 (ppm)

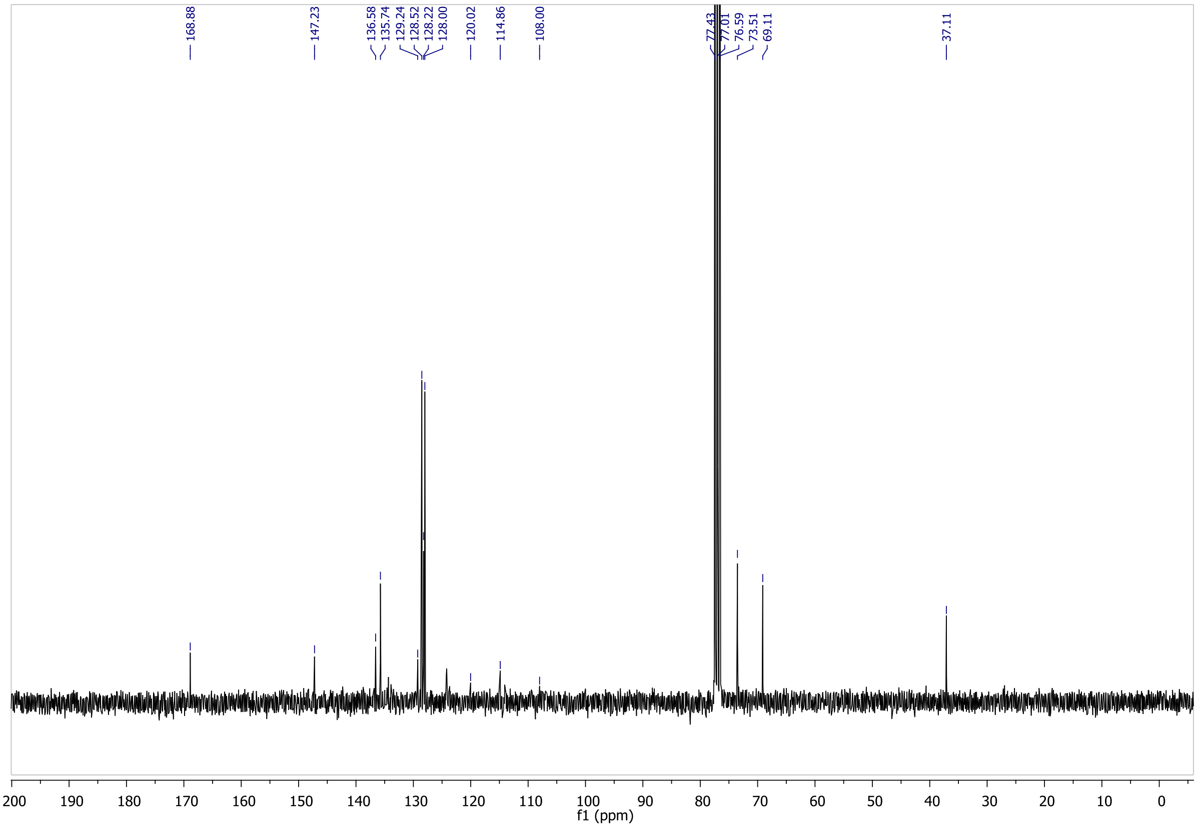
168.88
147.23
136.58
135.74
129.24
128.52
128.22
128.00
120.02
114.86
108.00
77.43
77.01
76.59
73.51
69.11
37.11
200 190 180 170 160 150 140 130 120 110 100 90 80 70 60 50 40 30 20 10 0
f1 (ppm)

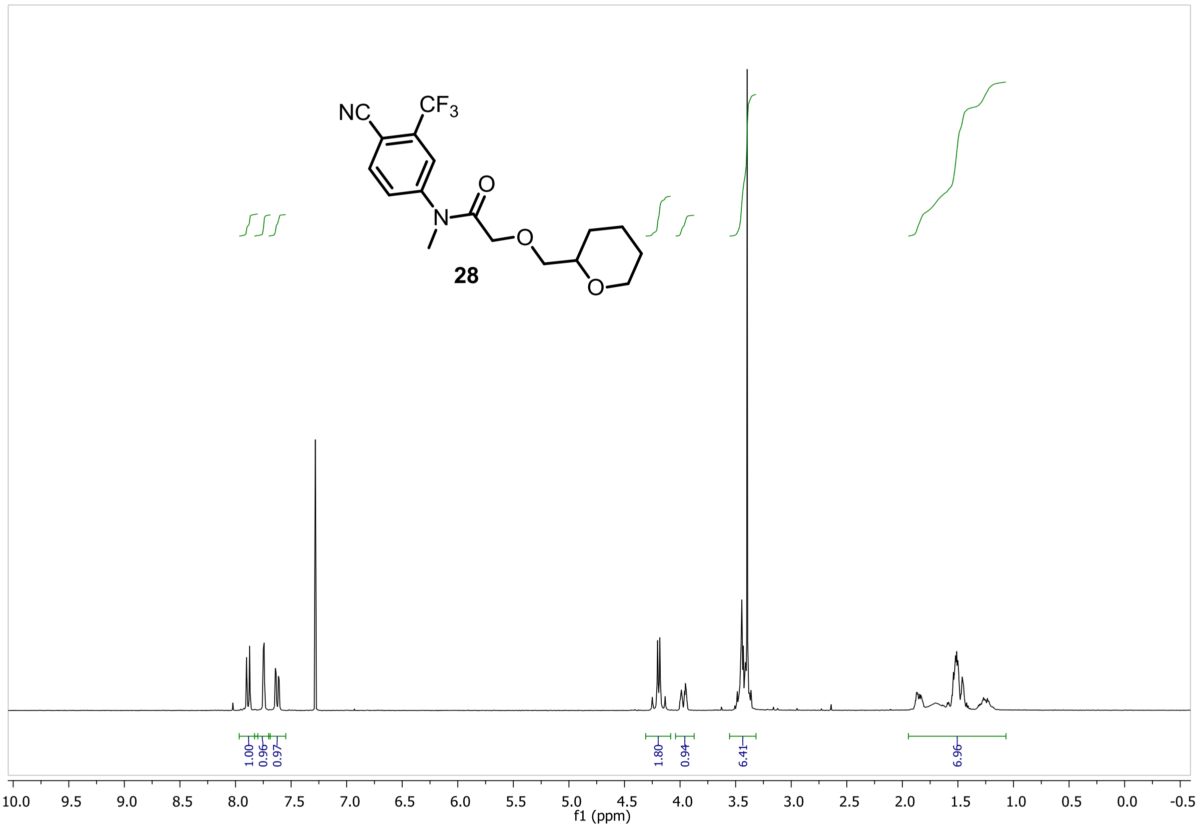

NC
CF3
N
O
O
O
28
1.00
0.96
0.97
1.80
0.94
6.41
6.96
f1 (ppm)

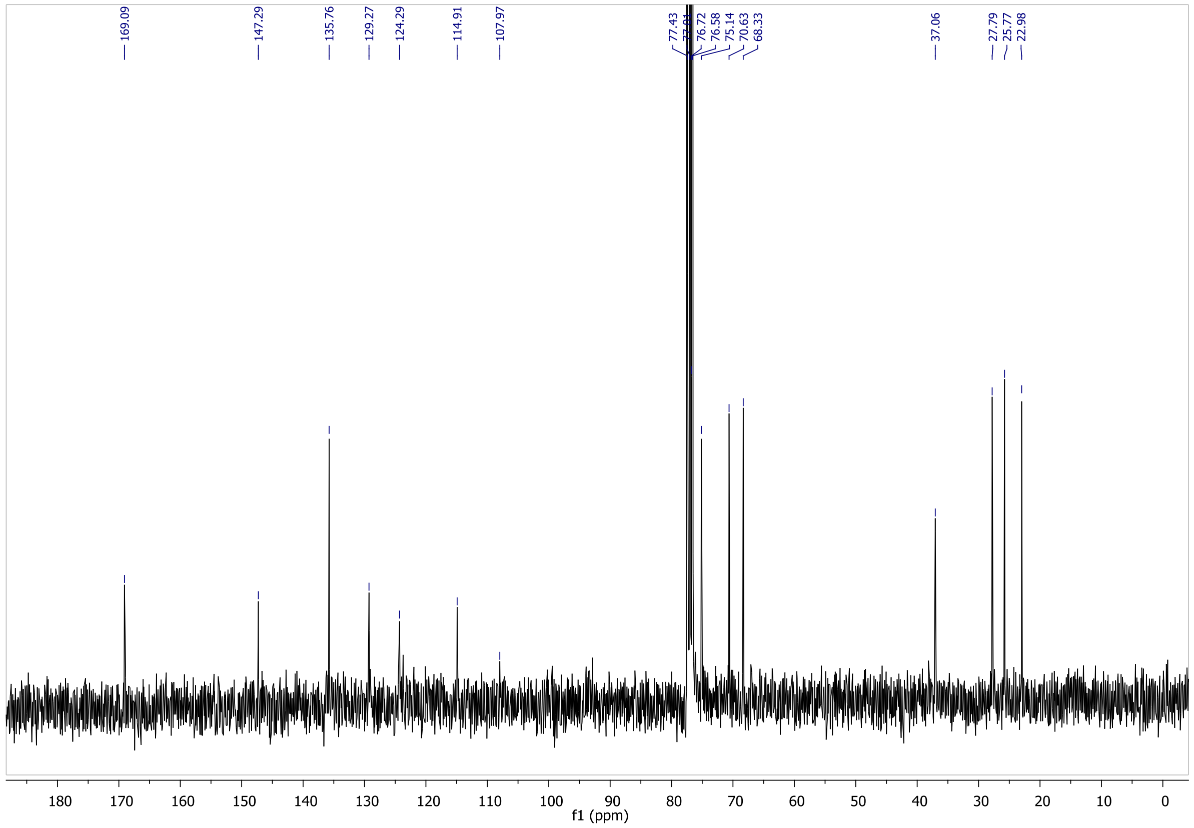

169.09
147.29
135.76
129.27
124.29
114.91
107.97
77.43
77.01
76.72
76.58
75.14
70.63
68.33
37.06
27.79
25.77
22.98
f1 (ppm)

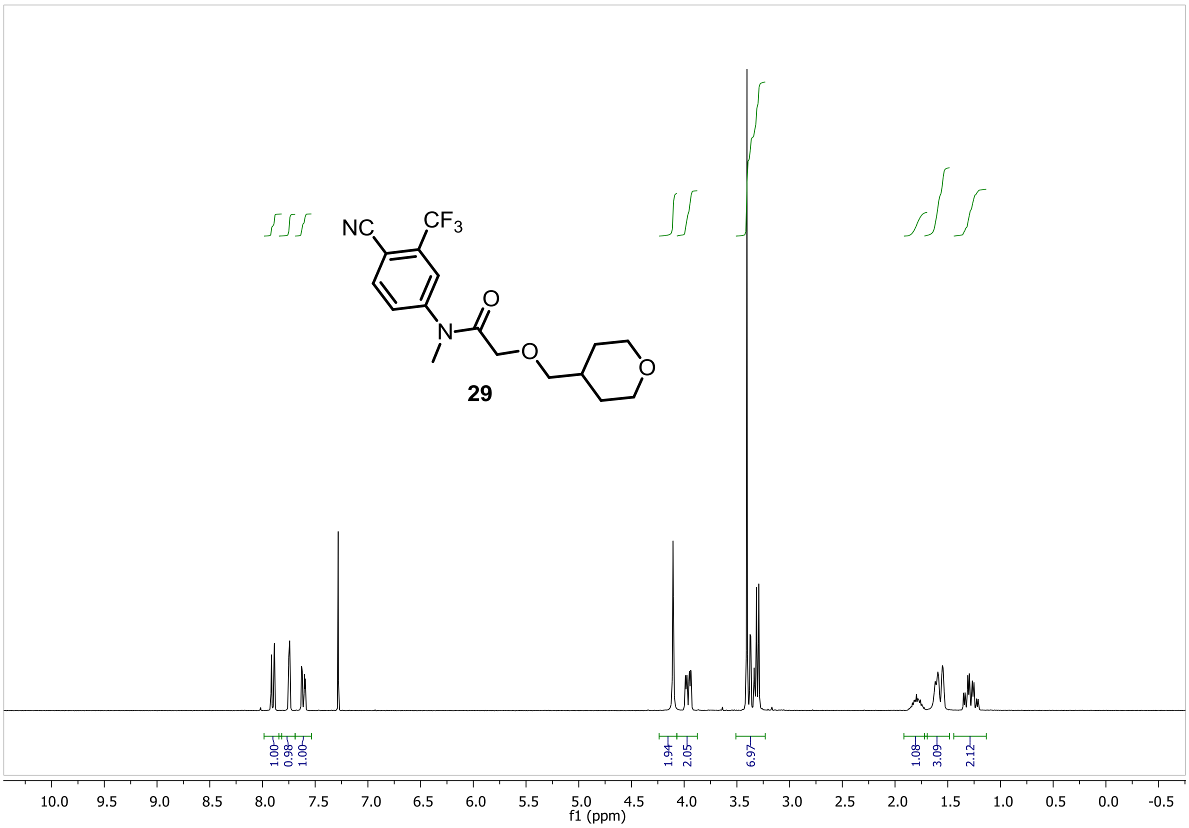
NC
CF3
O
N
O
O
29
1.00
0.98
1.00
1.94
2.05
6.97
1.08
3.09
2.12
10.0 9.5 9.0 8.5 8.0 7.5 7.0 6.5 6.0 5.5 5.0 4.5 4.0 3.5 3.0 2.5 2.0 1.5 1.0 0.5 0.0 -0.5
f1 (ppm)

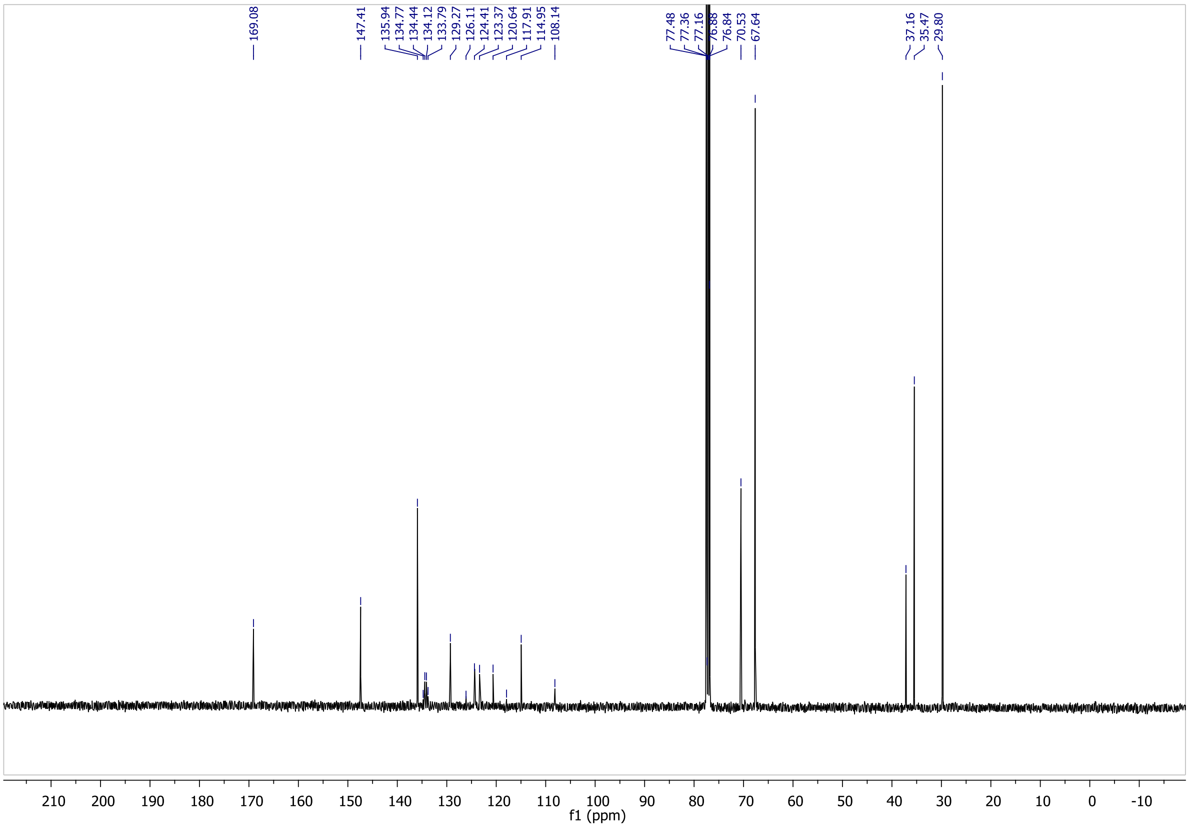
169.08
147.41
135.94
134.77
134.44
134.12
133.79
129.27
126.11
124.41
123.37
120.64
117.91
114.95
108.14
77.48
77.36
77.16
76.84
70.53
67.64
37.16
35.47
29.80
210 200 190 180 170 160 150 140 130 120 110 100 90 80 70 60 50 40 30 20 10 0 -10
f1 (ppm)

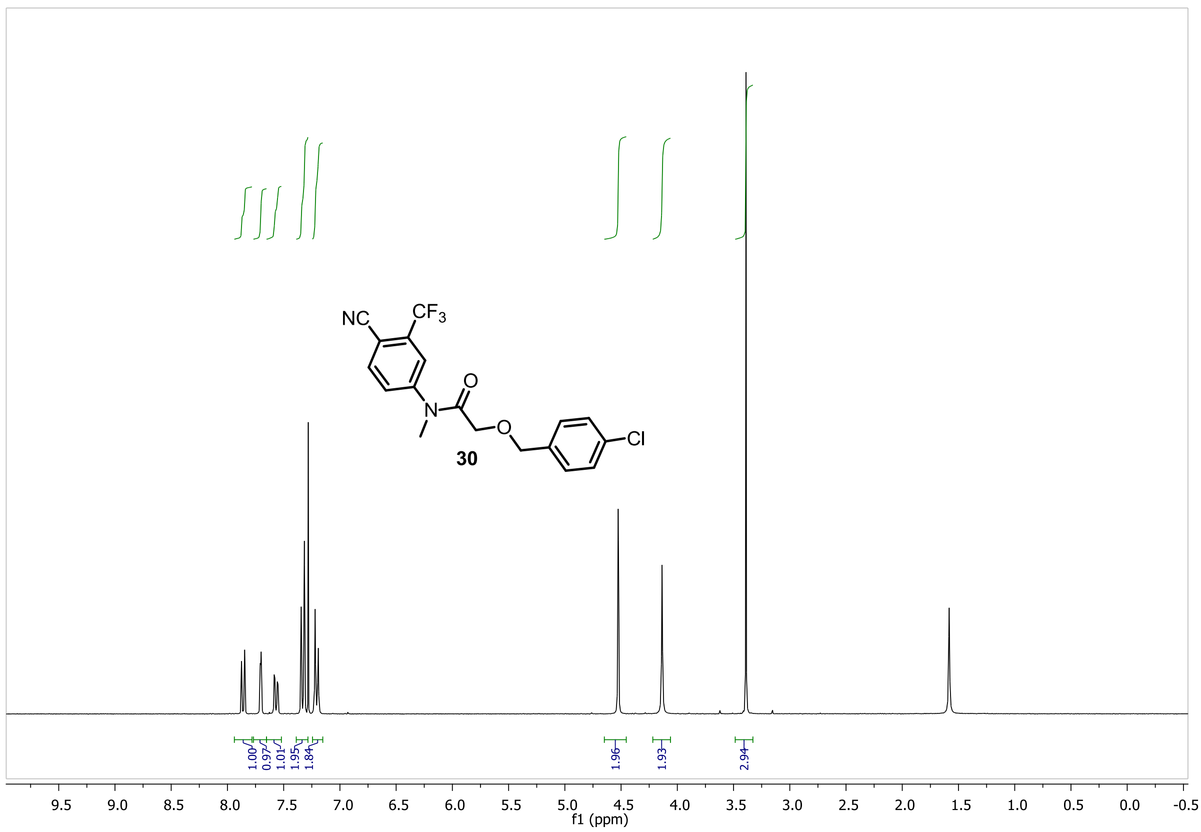
NC
CF3
O
N
O
Cl
30
1.00
0.97
1.01
1.95
1.84
1.96
1.93
2.94
9.5 9.0 8.5 8.0 7.5 7.0 6.5 6.0 5.5 5.0 4.5 4.0 3.5 3.0 2.5 2.0 1.5 1.0 0.5 0.0 -0.5
f1 (ppm)

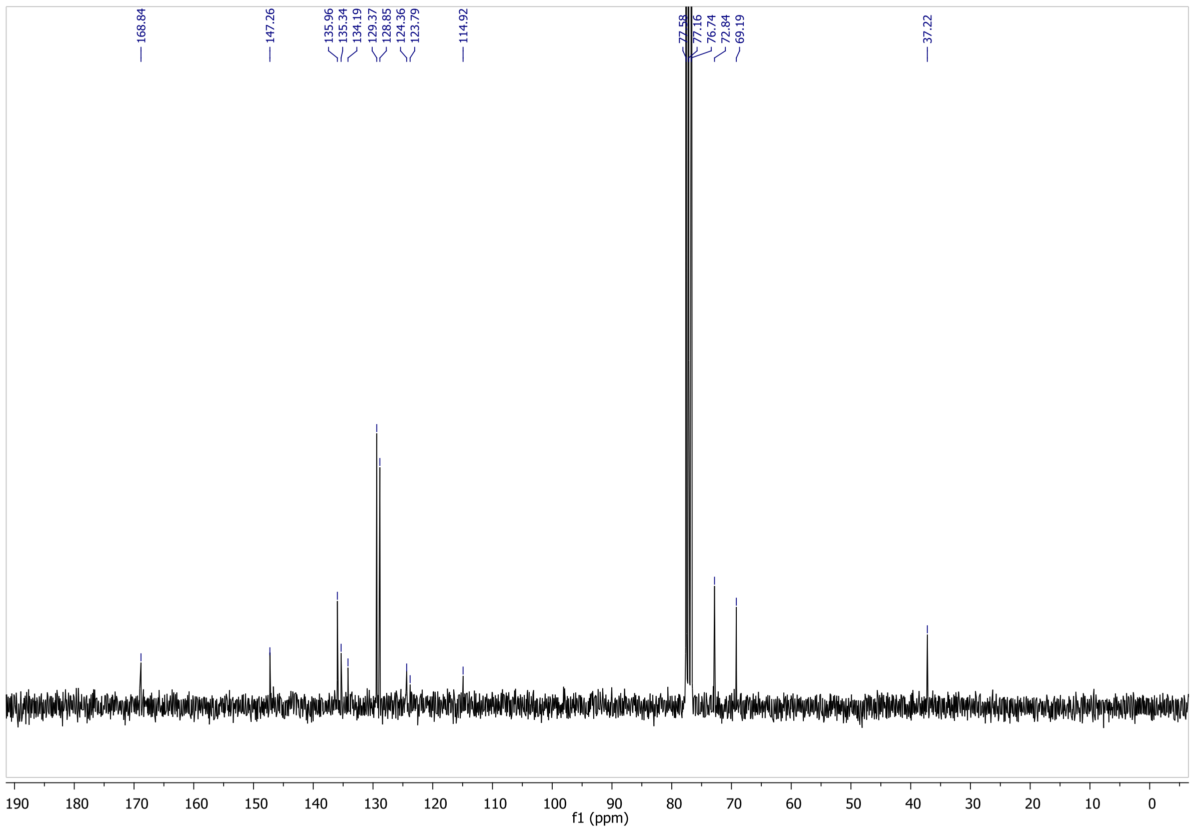
168.84
147.26
135.96
135.34
134.19
129.37
128.85
124.36
123.79
114.92
77.58
77.16
76.74
72.84
69.19
37.22
190 180 170 160 150 140 130 120 110 100 90 80 70 60 50 40 30 20 10 0
f1 (ppm)

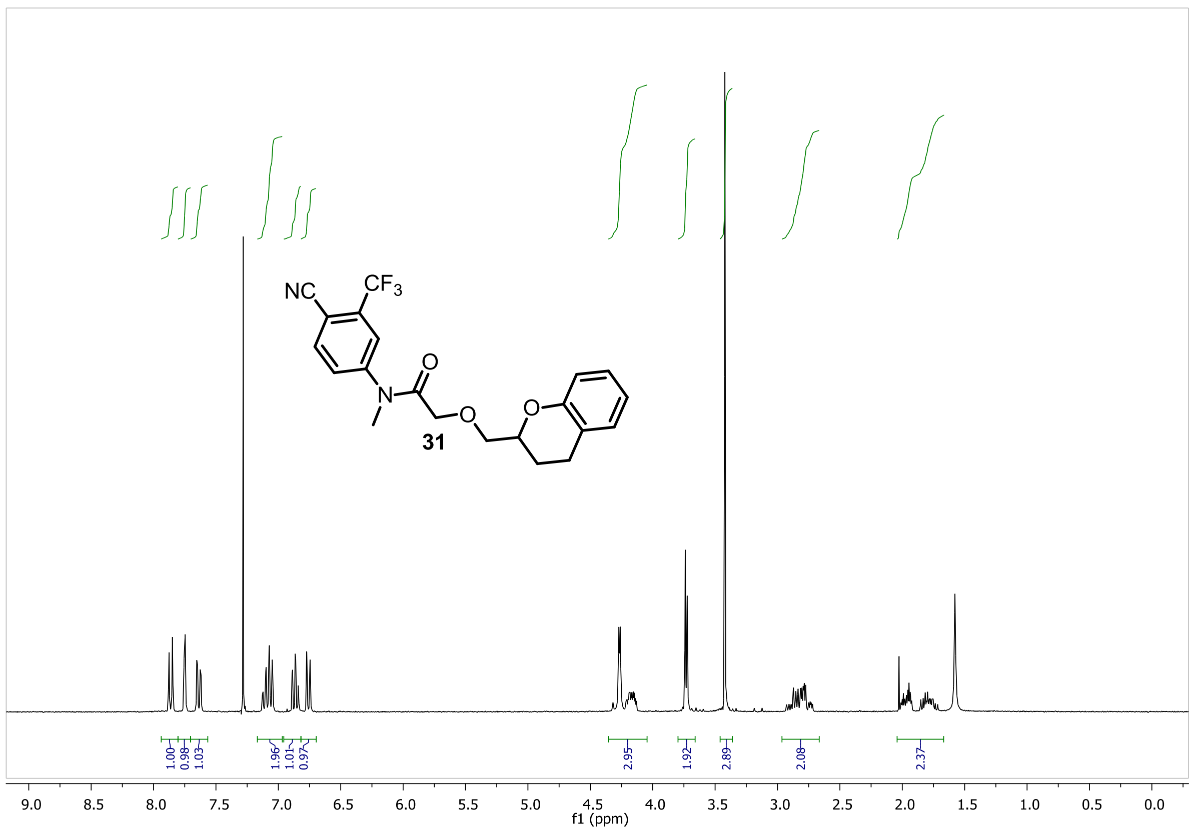
NC
CF3
O
N
O
O
31
1.00
0.98
1.03
1.96
1.01
0.97
2.95
1.92
2.89
2.08
2.37
9.0 8.5 8.0 7.5 7.0 6.5 6.0 5.5 5.0 4.5 4.0 3.5 3.0 2.5 2.0 1.5 1.0 0.5 0.0
f1 (ppm)

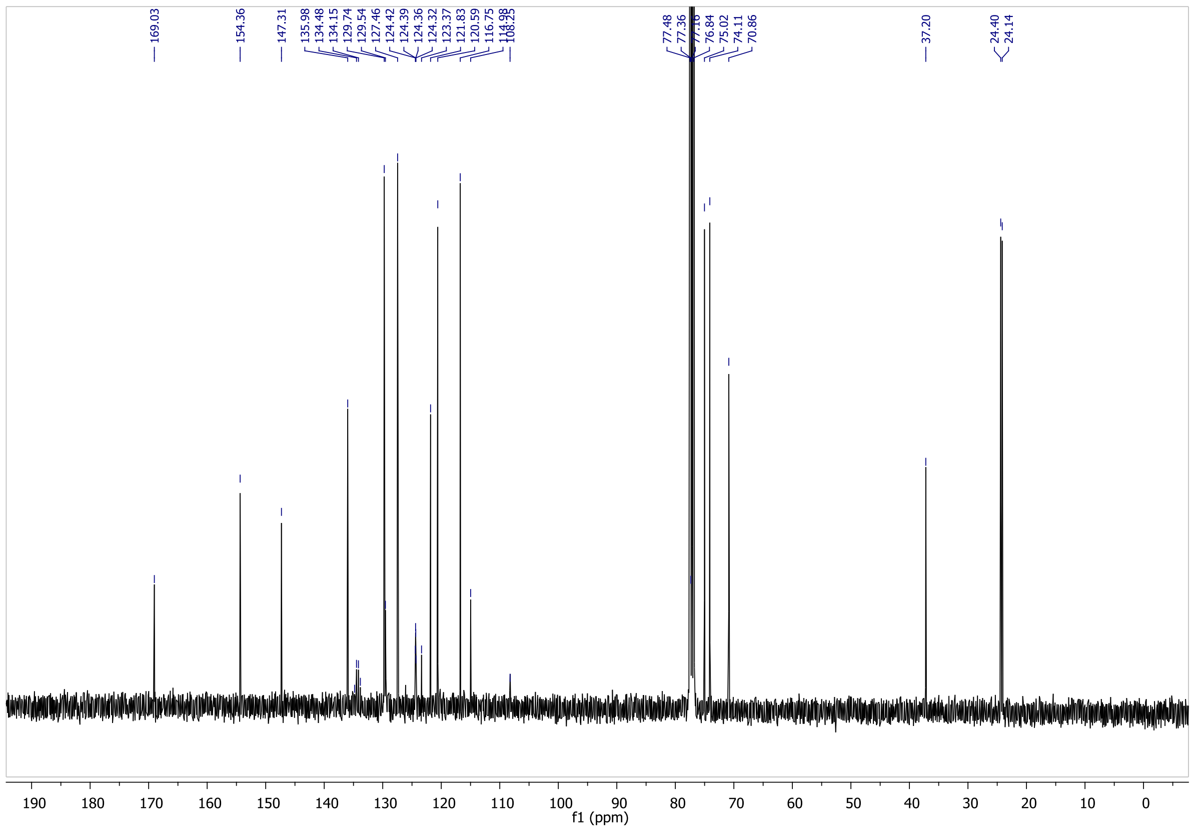
169.03
154.36
147.31
135.98
134.48
134.15
129.74
129.54
127.46
124.42
124.39
124.36
124.32
123.37
121.83
120.59
116.75
77.48
77.36
76.84
75.02
74.11
70.86
37.20
24.40
24.14
190 180 170 160 150 140 130 120 110 100 90 80 70 60 50 40 30 20 10 0
f1 (ppm)

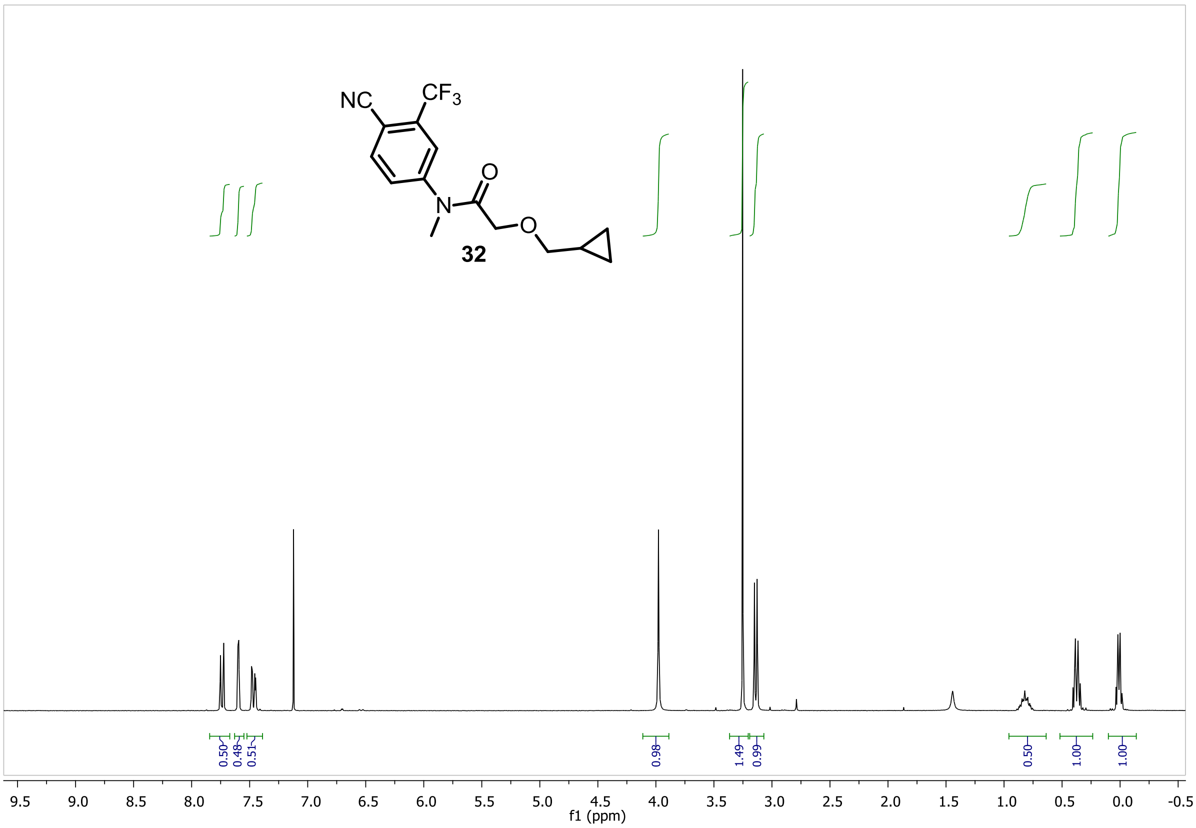
NC
CF3
O
N
O
32
0.50
0.48
0.51
0.98
1.49
0.99
0.50
1.00
1.00
f1 (ppm)

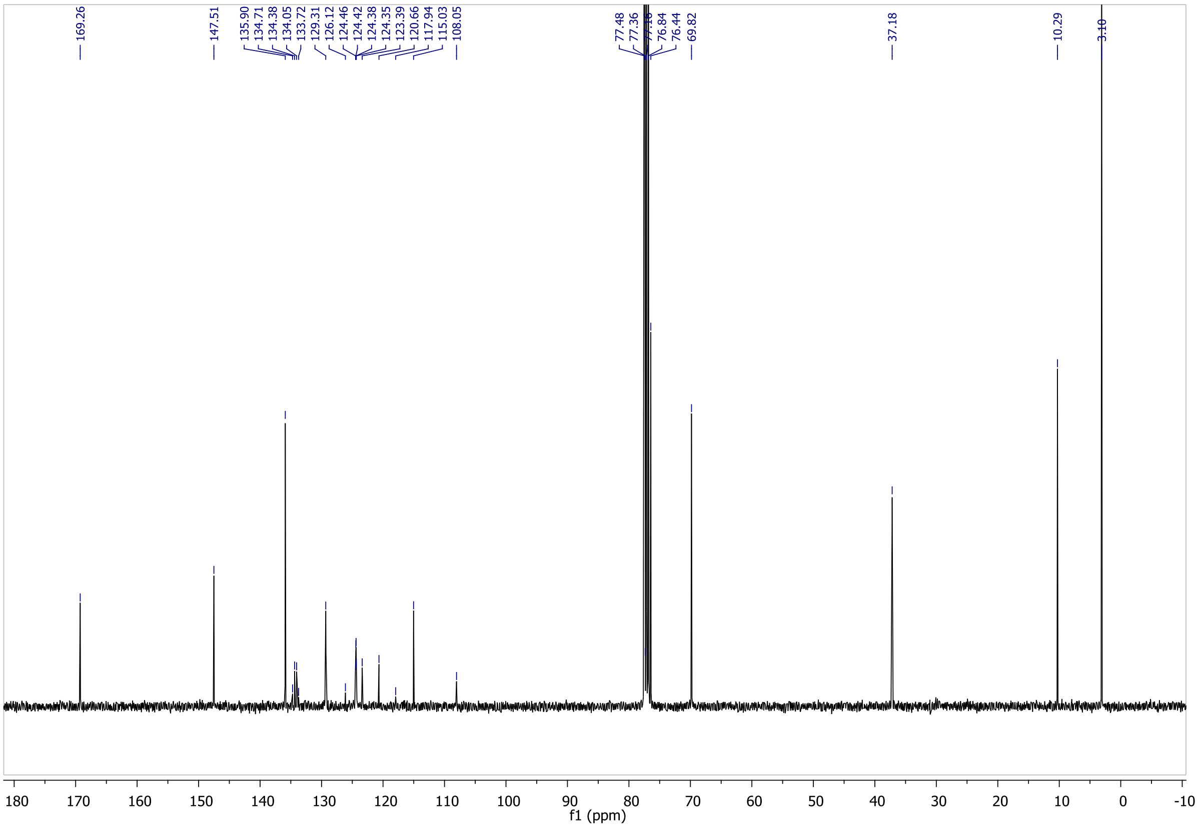
169.26
147.51
135.90
134.71
134.38
134.05
133.72
129.31
126.12
124.46
124.42
124.38
124.35
123.39
120.66
117.94
115.03
108.05
77.48
77.36
76.84
76.44
69.82
37.18
10.29
3.10
f1 (ppm)

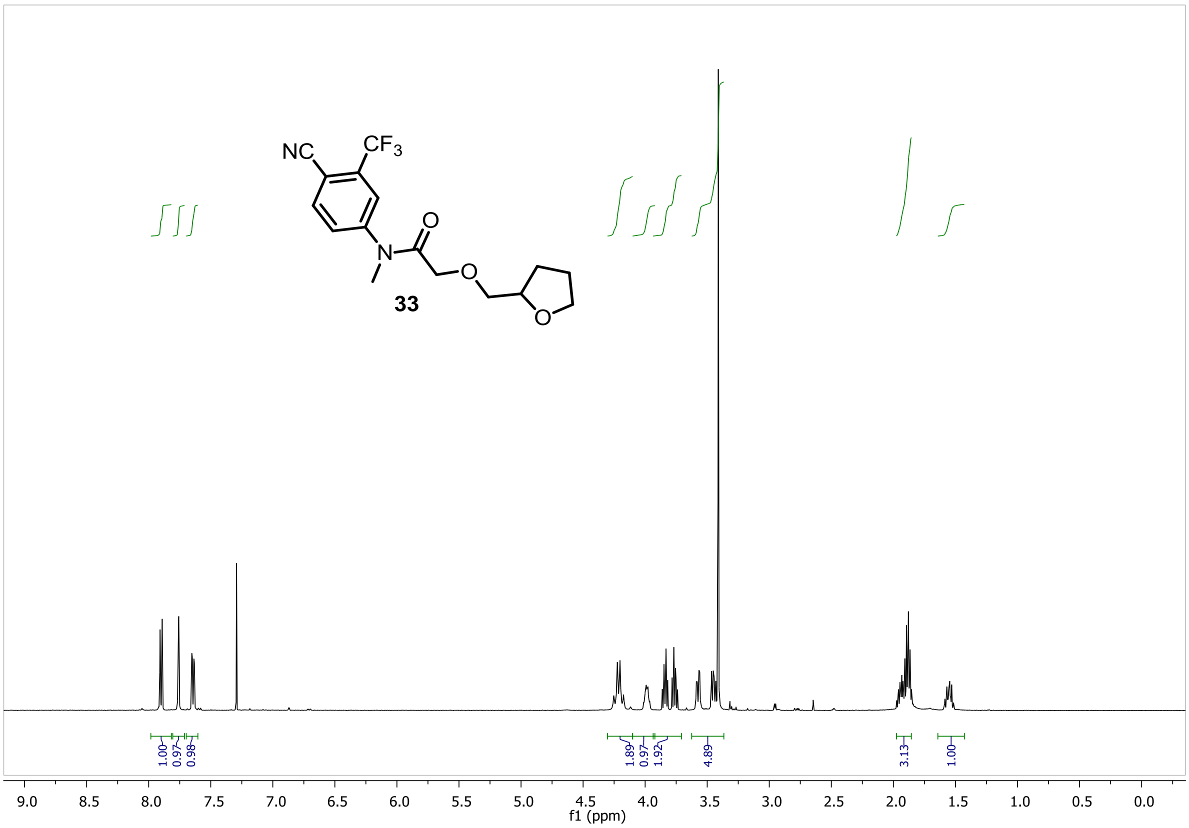

NC
CF3
O
N
O
O
33
1.00
0.97
0.98
1.89
0.97
1.92
4.89
3.13
1.00
9.0 8.5 8.0 7.5 7.0 6.5 6.0 5.5 5.0 4.5 4.0 3.5 3.0 2.5 2.0 1.5 1.0 0.5 0.0
f1 (ppm)

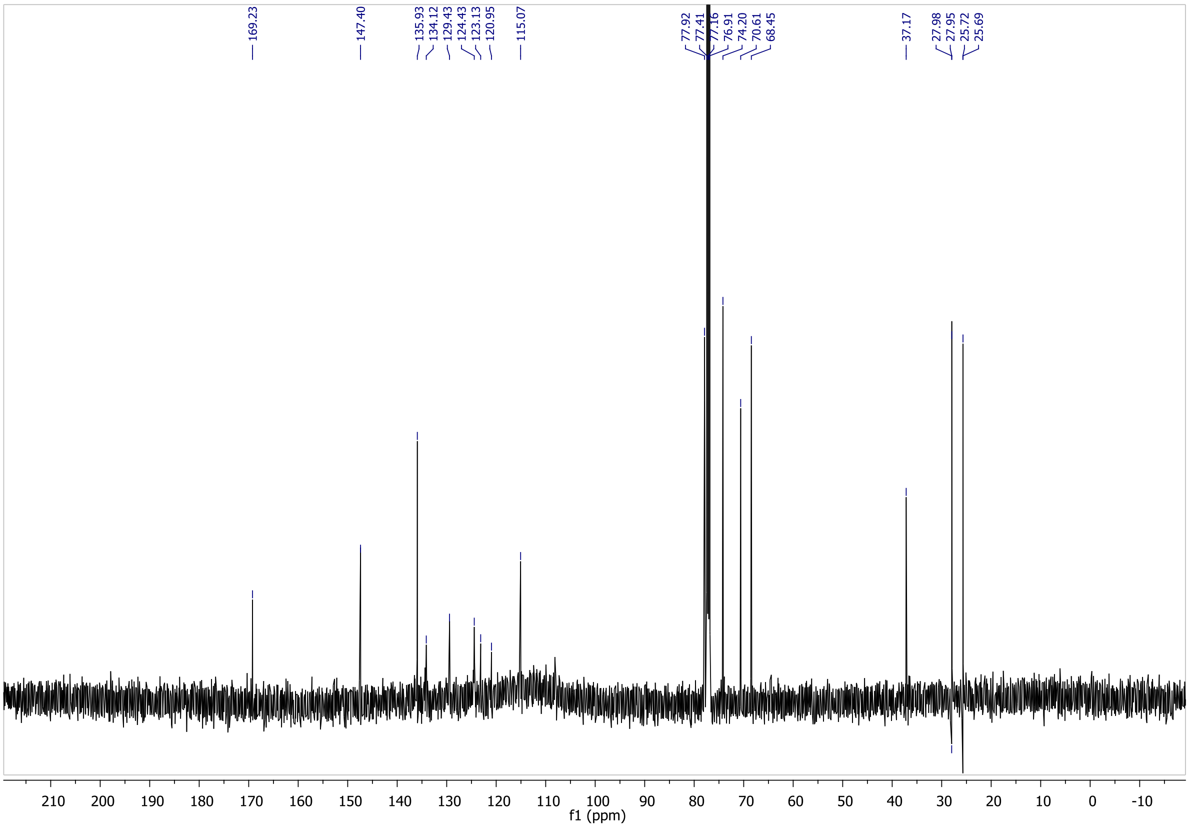

169.23
147.40
135.93
134.12
129.43
124.43
123.13
120.95
115.07
77.92
77.41
77.16
76.91
74.20
70.61
68.45
37.17
27.98
27.95
25.72
25.69
210 200 190 180 170 160 150 140 130 120 110 100 90 80 70 60 50 40 30 20 10 0 -10
f1 (ppm)

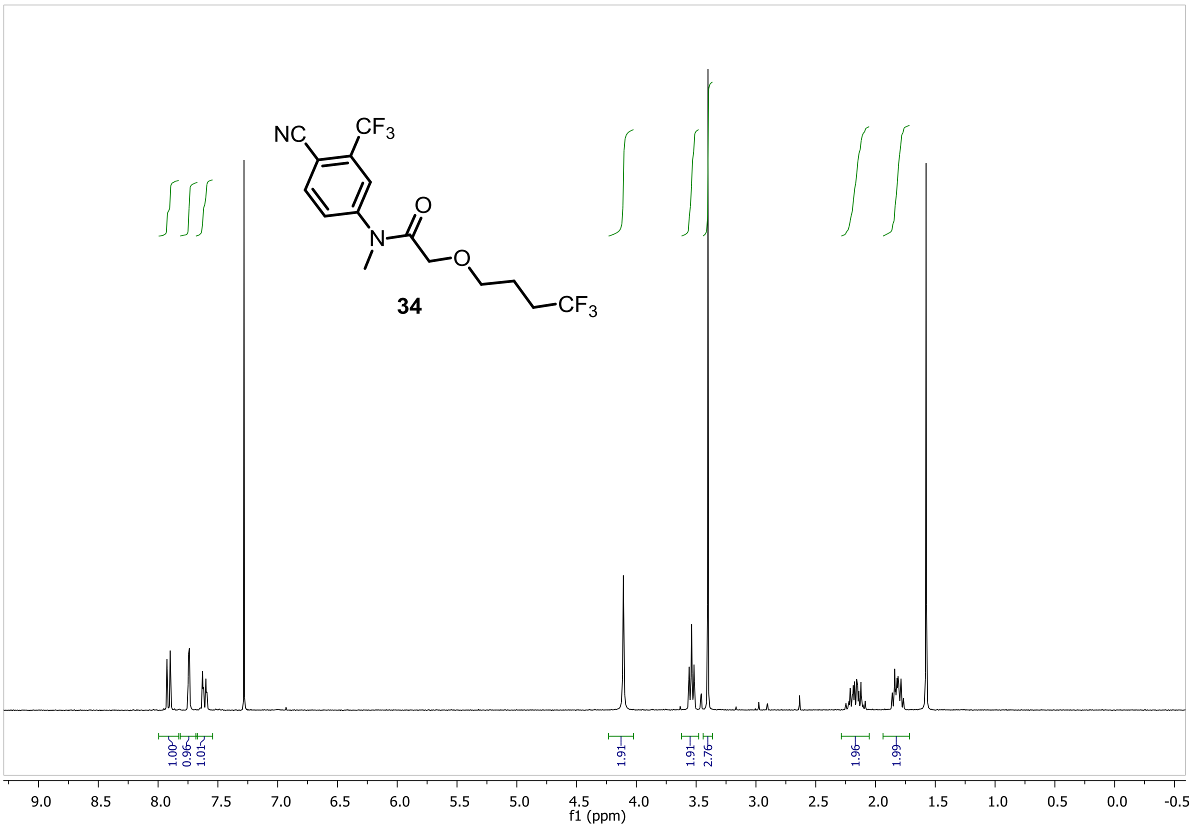

NC
CF3
N
O
O
CF3
34
1.00
0.96
1.01
1.91
1.91
2.76
1.96
1.99
9.0 8.5 8.0 7.5 7.0 6.5 6.0 5.5 5.0 4.5 4.0 3.5 3.0 2.5 2.0 1.5 1.0 0.5 0.0 -0.5
f1 (ppm)

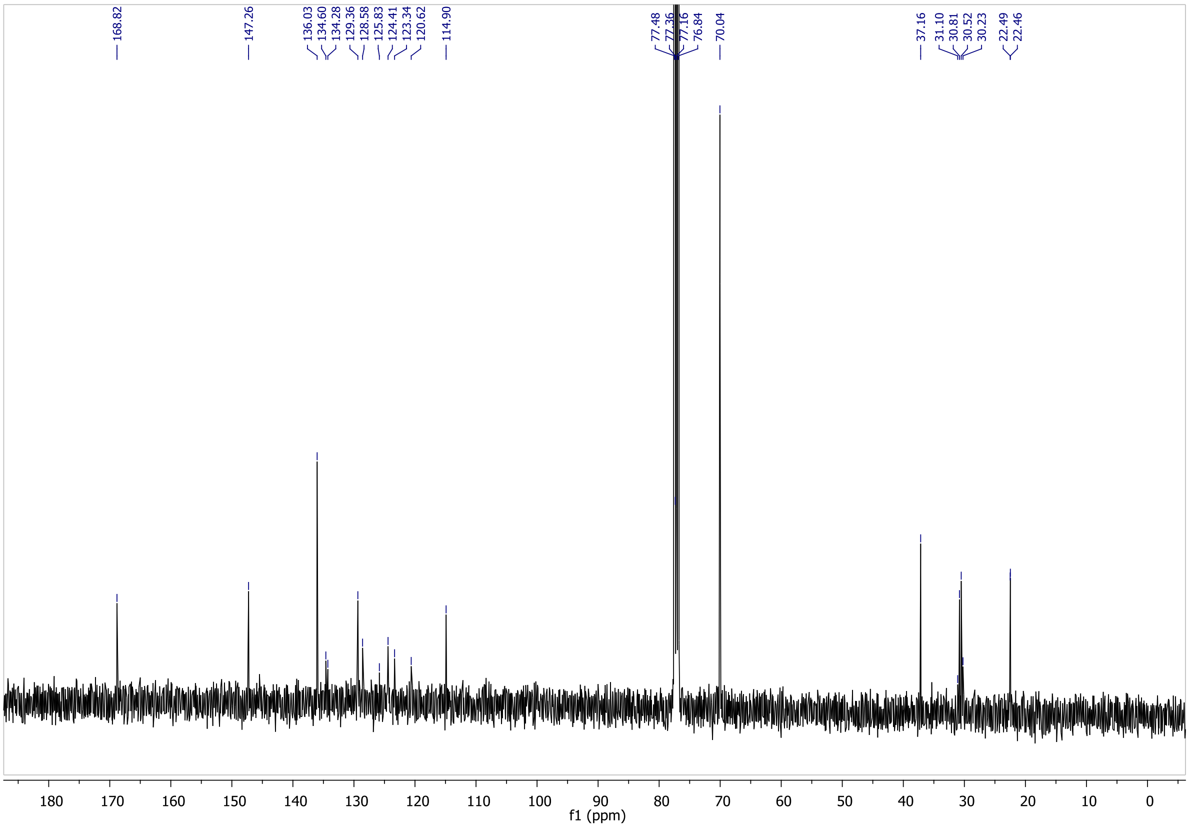

168.82
147.26
136.03
134.60
134.28
129.36
128.58
125.83
124.41
123.34
120.62
114.90
77.48
77.36
77.16
76.84
70.04
37.16
31.10
30.81
30.52
30.23
22.49
22.46
180 170 160 150 140 130 120 110 100 90 80 70 60 50 40 30 20 10 0
f1 (ppm)

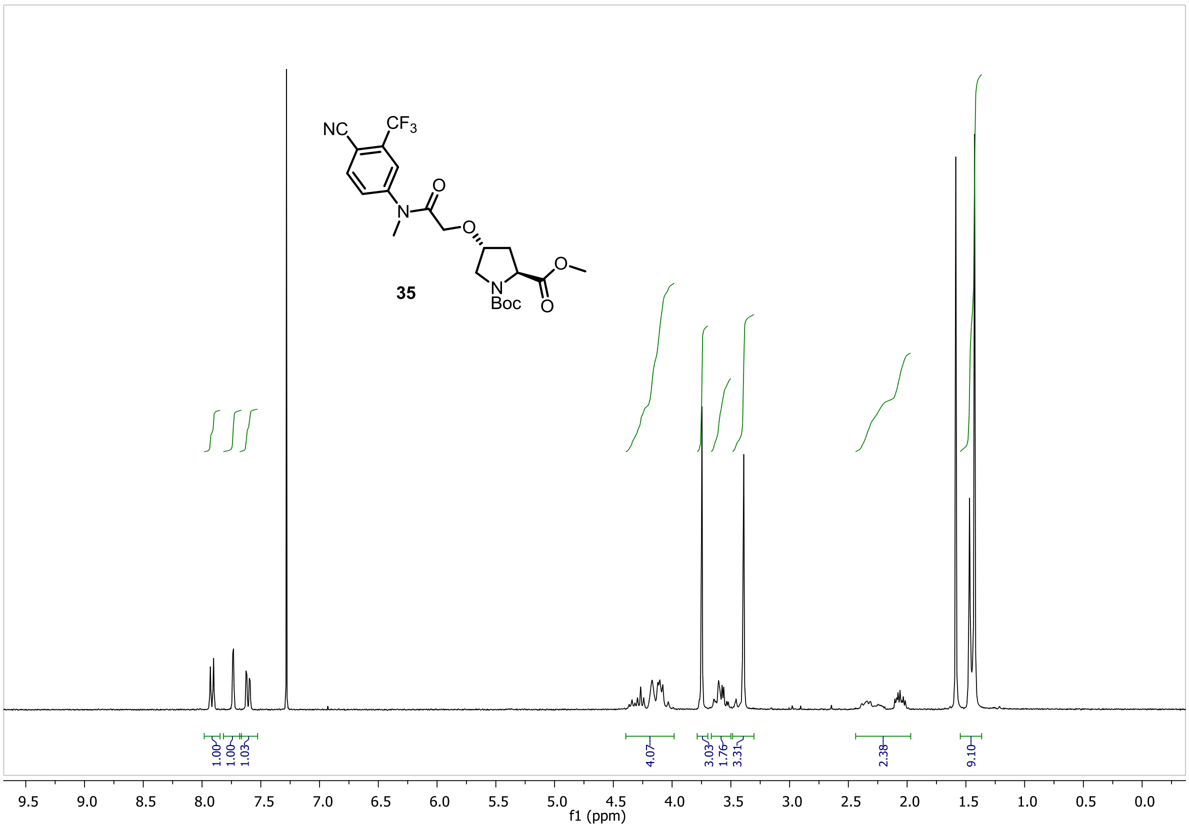
NC
CF3
O
N
O
O
N
Boc
O
35
1.00
1.00
1.03
4.07
3.03
1.76
3.31
2.38
9.10
9.5 9.0 8.5 8.0 7.5 7.0 6.5 6.0 5.5 5.0 4.5 4.0 3.5 3.0 2.5 2.0 1.5 1.0 0.5 0.0
f1 (ppm)

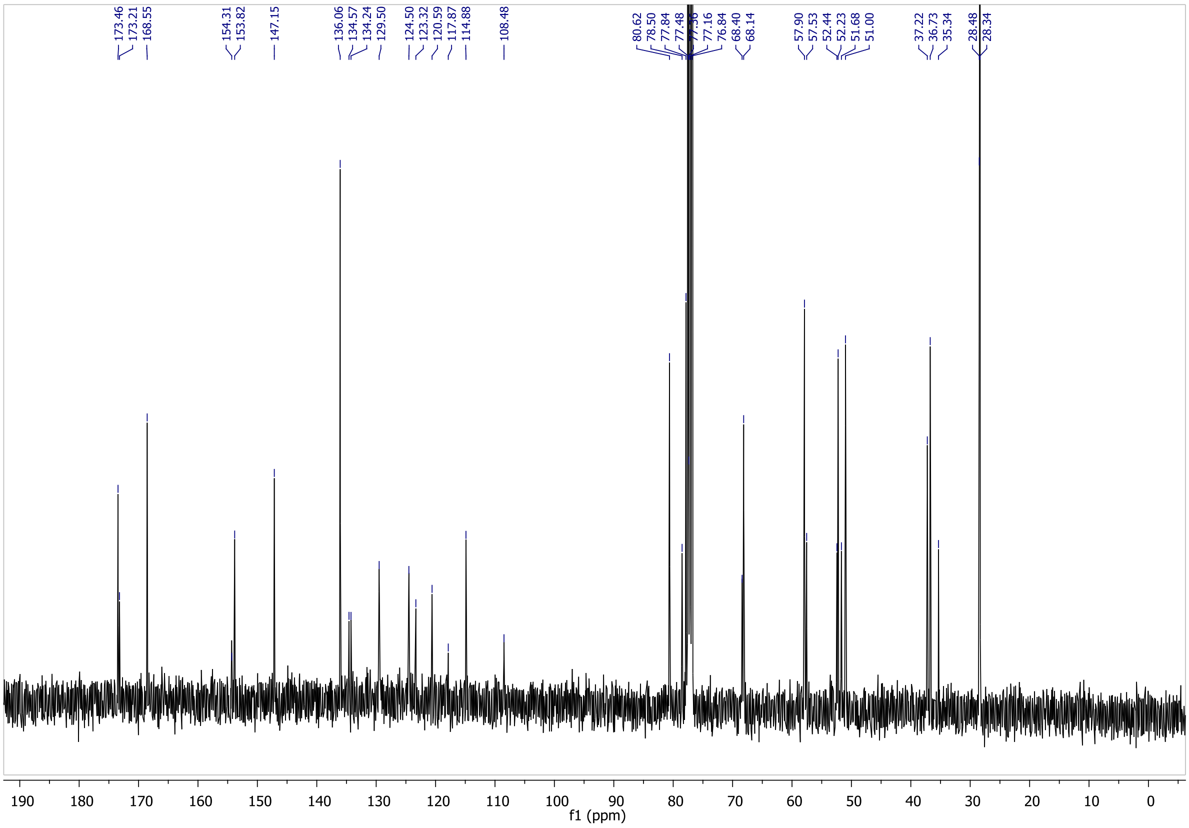
173.46
173.21
168.55
154.31
153.82
147.15
136.06
134.57
134.24
129.50
124.50
123.32
120.59
117.87
114.88
108.48
80.62
78.50
77.84
77.48
77.16
76.84
68.40
68.14
57.90
57.53
52.44
52.23
51.68
51.00
37.22
36.73
35.34
28.48
28.34
190 180 170 160 150 140 130 120 110 100 90 80 70 60 50 40 30 20 10 0
f1 (ppm)

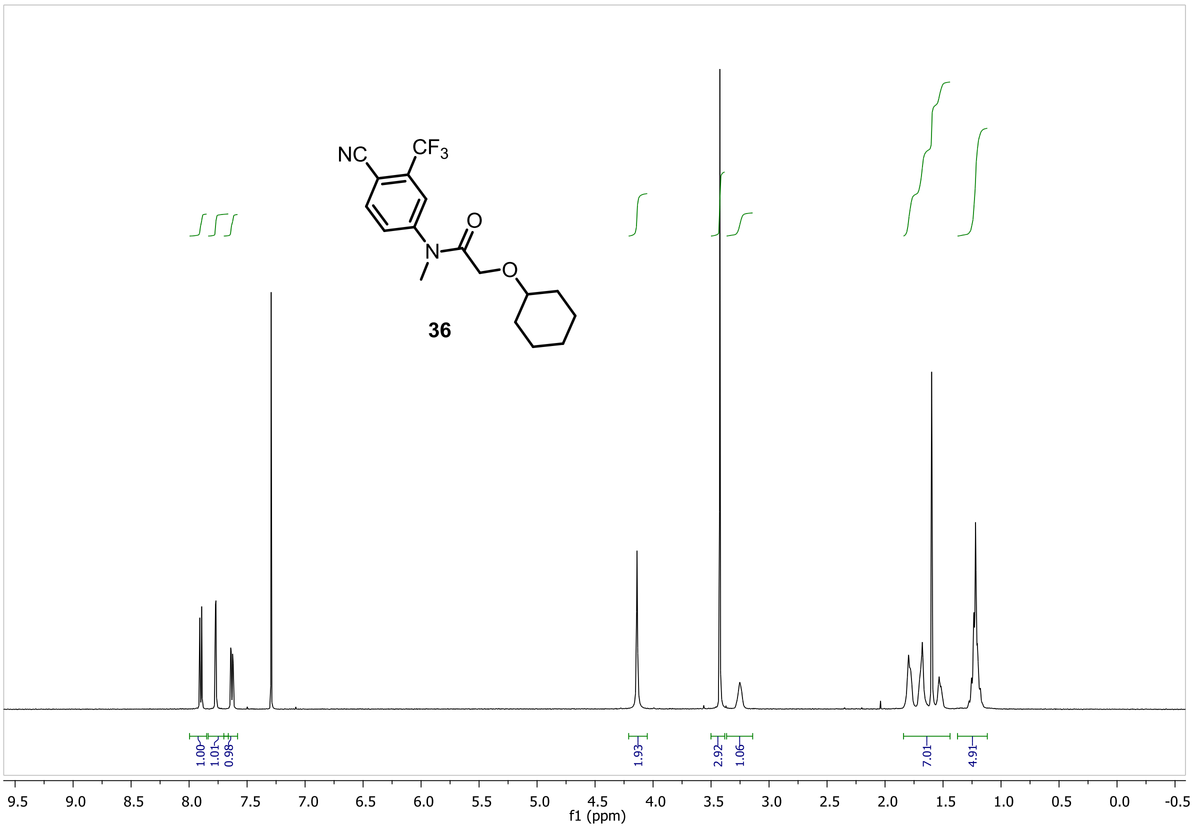
NC
CF3
N
O
O
36
1.00
1.01
0.98
1.93
2.92
1.06
7.01
4.91
9.5 9.0 8.5 8.0 7.5 7.0 6.5 6.0 5.5 5.0 4.5 4.0 3.5 3.0 2.5 2.0 1.5 1.0 0.5 0.0 -0.5
f1 (ppm)

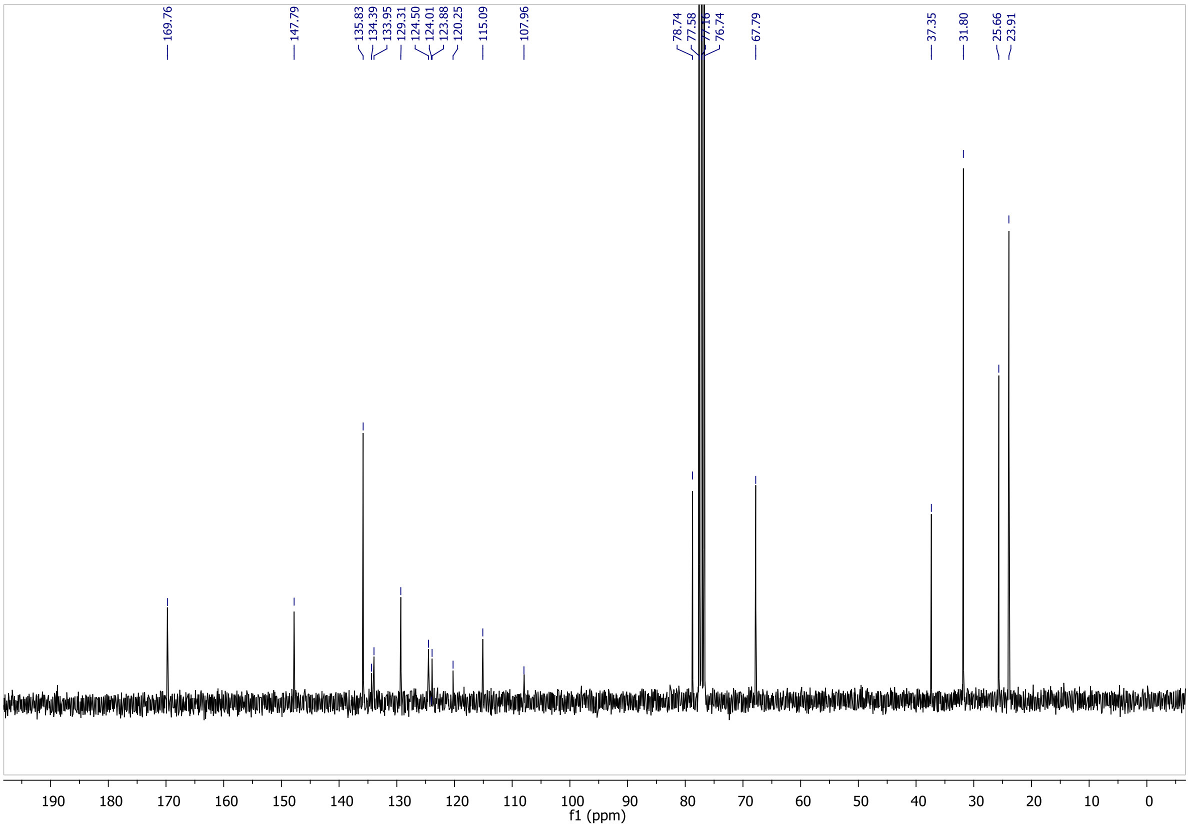
169.76
147.79
135.83
134.39
133.95
129.31
124.50
124.01
123.88
120.25
115.09
107.96
78.74
77.58
77.16
76.74
67.79
37.35
31.80
25.66
23.91
190 180 170 160 150 140 130 120 110 100 90 80 70 60 50 40 30 20 10 0
f1 (ppm)

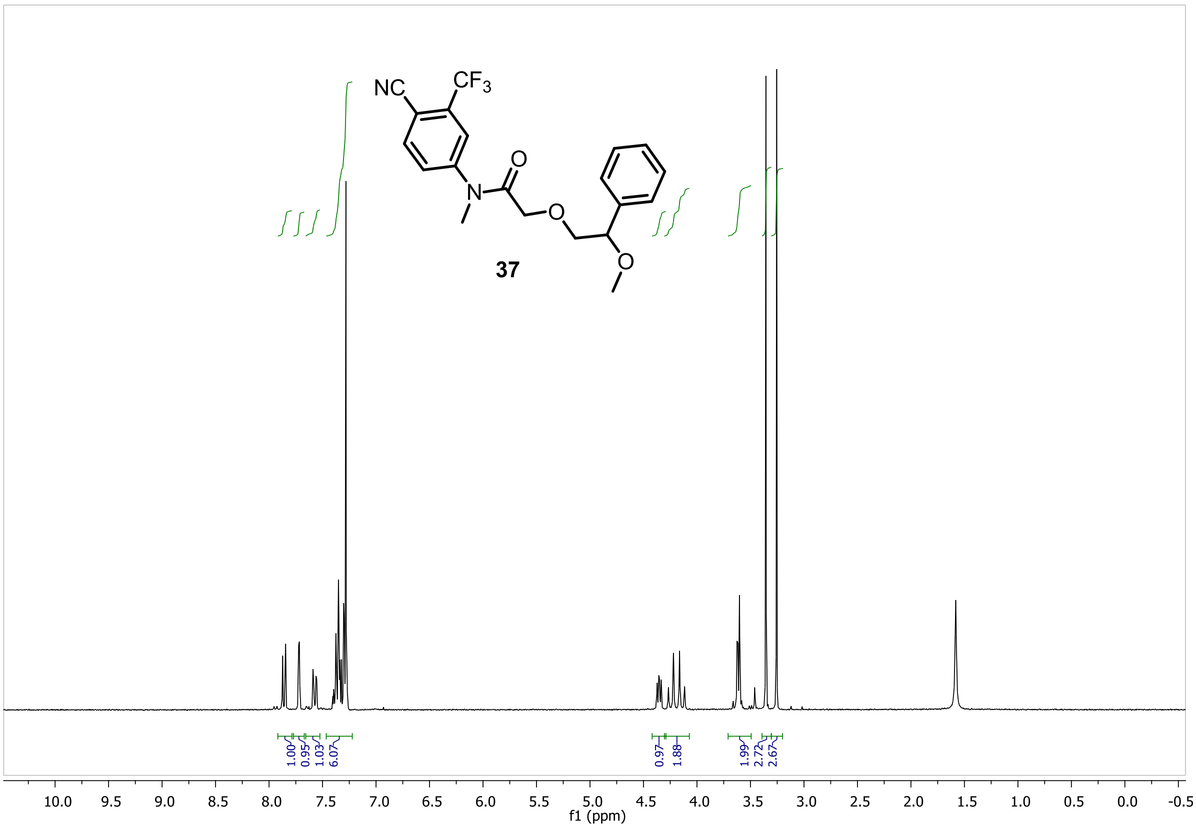
NC
CF3
O
N
O
O
37
1.00
0.95
1.03
6.07
0.97
1.88
1.99
2.72
2.67
10.0 9.5 9.0 8.5 8.0 7.5 7.0 6.5 6.0 5.5 5.0 4.5 4.0 3.5 3.0 2.5 2.0 1.5 1.0 0.5 0.0 -0.5
f1 (ppm)

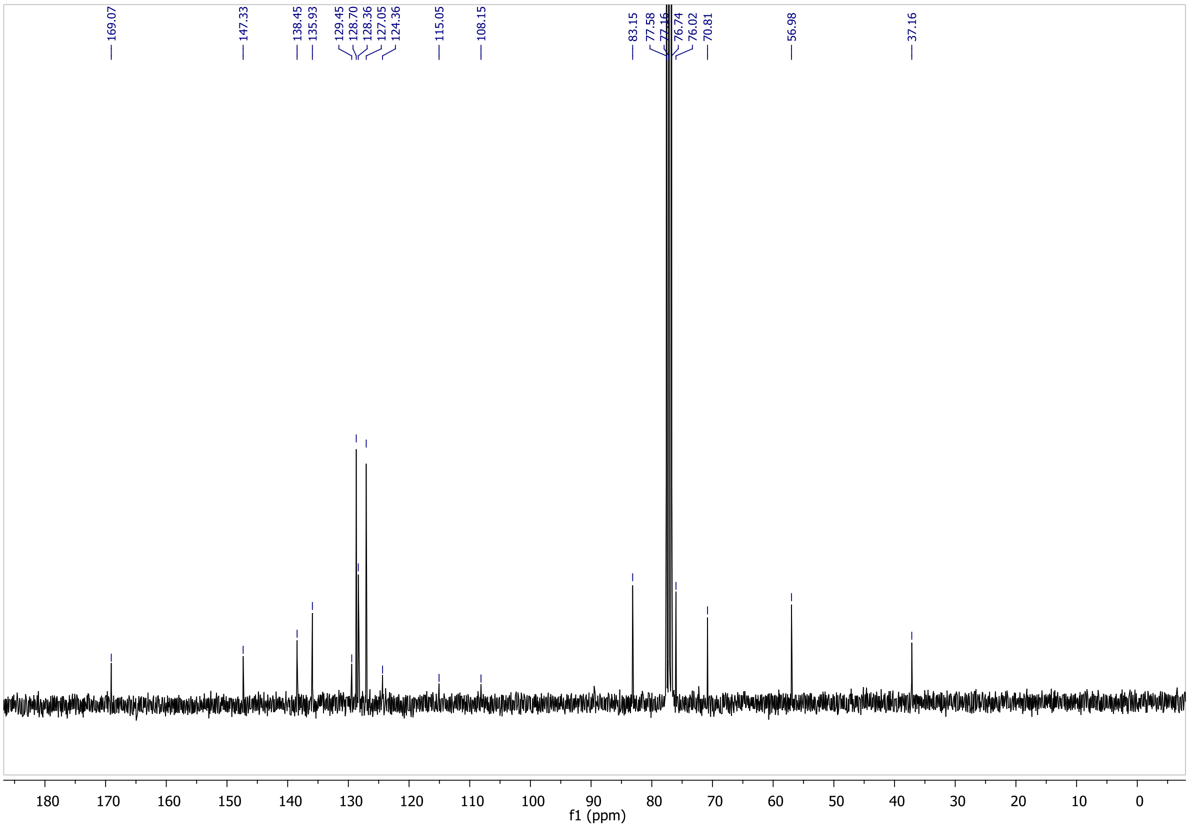
169.07
147.33
138.45
135.93
129.45
128.70
128.36
127.05
124.36
115.05
108.15
83.15
77.58
77.16
76.74
76.02
70.81
56.98
37.16
180 170 160 150 140 130 120 110 100 90 80 70 60 50 40 30 20 10 0
f1 (ppm)

12 References:

[1] J. K. Stille, Y. Becker, *J. Org. Chem.* **1980**, *45*, 2139–2145.

[2] R. C. Larock, Y. Wang, *Tetrahedron Lett.* **2002**, *43*, 21–23.

[3] T. Assaad, S. Mavel, S. M. Parsons, S. Kruse, L. Galineau, H. Allouchi, M. Kassiou, S. Chalon, D. Guilloteau, P. Emond, *Bioorg. Med. Chem. Lett.* **2006**, *16*, 2654–7.

[4] P. G. Jagtap, Z. Chen, K. Koppetsch, E. Piro, P. Fronce, G. J. Southan, K.-N. Klotz, *Tetrahedron Lett.* **2009**, *50*, 2693–2696.

[5] S.-K. Kang, J.-H. Jeon, T. Yamaguchi, R.-K. Hong, B.-S. Ko, *Tetrahedron: Asymmetry*.**1995**, *6*, 97–100.
